# Increasing Maternal or Post-Weaning Folic Acid Alters Gene Expression and Moderately Changes Behavior in the Offspring

**DOI:** 10.1371/journal.pone.0101674

**Published:** 2014-07-09

**Authors:** Subit Barua, Kathryn K. Chadman, Salomon Kuizon, Diego Buenaventura, Nathan W. Stapley, Felicia Ruocco, Umme Begum, Sara R. Guariglia, W. Ted Brown, Mohammed A. Junaid

**Affiliations:** 1 Department of Developmental Biochemistry, New York State Institute for Basic Research in Developmental Disabilities, Staten Island, New York, United States of America; 2 Department of Developmental Neurobiology, New York State Institute for Basic Research in Developmental Disabilities, Staten Island, New York, United States of America; 3 Department of Environmental Health Sciences, Columbia University, New York, United States of America; 4 Department of Human Genetics, New York State Institute for Basic Research in Developmental Disabilities, Staten Island, New York, United States of America; 5 Graduate Center and College of Staten Island, City University of New York, Staten Island, New York, United States of America; University of Missouri, United States of America

## Abstract

**Background:**

Studies have indicated that altered maternal micronutrients and vitamins influence the development of newborns and altered nutrient exposure throughout the lifetime may have potential health effects and increased susceptibility to chronic diseases. In recent years, folic acid (FA) exposure has significantly increased as a result of mandatory FA fortification and supplementation during pregnancy. Since FA modulates DNA methylation and affects gene expression, we investigated whether the amount of FA ingested during gestation alters gene expression in the newborn cerebral hemisphere, and if the increased exposure to FA during gestation and throughout the lifetime alters behavior in C57BL/6J mice.

**Methods:**

Dams were fed FA either at 0.4 mg or 4 mg/kg diet throughout the pregnancy and the resulting pups were maintained on the diet throughout experimentation. Newborn pups brain cerebral hemispheres were used for microarray analysis. To confirm alteration of several genes, quantitative RT-PCR (qRT-PCR) and Western blot analyses were performed. In addition, various behavior assessments were conducted on neonatal and adult offspring.

**Results:**

Results from microarray analysis suggest that the higher dose of FA supplementation during gestation alters the expression of a number of genes in the newborns’ cerebral hemispheres, including many involved in development. QRT-PCR confirmed alterations of nine genes including down-regulation of *Cpn2*, *Htr4*, *Zfp353*, *Vgll2* and up-regulation of *Xist*, *Nkx6-3*, *Leprel1*, *Nfix, Slc17a7*. The alterations in the expression of *Slc17a7* and *Vgll2* were confirmed at the protein level. Pups exposed to the higher dose of FA exhibited increased ultrasonic vocalizations, greater anxiety-like behavior and hyperactivity. These findings suggest that although FA plays a significant role in mammalian cellular machinery, there may be a loss of benefit from higher amounts of FA. Unregulated high FA supplementation during pregnancy and throughout the life course may have lasting effects, with alterations in brain development resulting in changes in behavior.

## Introduction

Maternal nutrition during the peri- or post-conception period is strongly related to fetal development and the risk of non-communicable diseases. Micronutrients including vitamins and minerals are major intrauterine environmental factors that regulate the fetal genome machinery and human reproductive health [1], [2]. Since the early 1970’s, the vitamin folic acid (FA) has received considerable attention because of its important role in alleviating neural tube defects (NTDs) [3]. In 1998, mandatory FA fortification of breakfast cereals and grains was introduced by several countries [4]. These guidelines are credited with a significant reduction in NTDs in infants [5]. Intrauterine and early life exposure to FA has increased well beyond the minimum necessary requirements as a result of the additive levels from fortified foodstuffs, prescriptions by physicians and over-the-counter prenatal vitamins, as well as energy drinks [6]. Studies suggest that folate concentrations in the serum (57%) and red blood cells (136%) of pregnant women were much higher than the reported dietary folate intake (28%) [7], and concerns have been raised about the interference of excess unmetabolized FA [8], [9].

Several epidemiological reports have suggested that excess FA increases the risk of asthma and type 2 diabetes in newborns [10]–[12], and evidence of unmetabolized FA has been found in fetal cord blood [13]. While studies have found an important role of maternal FA in the proper closure of the neural tube, there have been no published reports yet on whether higher FA supplementation during pregnancy and throughout life may impact brain development. A previous studies from our laboratory has showed that FA supplementation significantly dysregulates gene expression in human lymphoblastoid cell lines [14], and our recent study in a mouse model has shown that gestational FA induces substantial alterations in methylation patterns of several genes in the cerebral hemispheres of the offspring [15]. Studies in laboratory animals have shown adverse effects including tumors and birth defects in offspring exposed to higher FA concentrations, and suppression of thyroid function with motivational and spatial memory deficits in adolescence [6], [16]–[22].

The current study had two objectives: first, to determine if the amount of maternal FA supplementation during gestation modulates gene expression in the developing cerebral hemispheres and consequently affects neonatal behavior, and second, to explore if gestational and lifelong exposure to excess FA alters the behavior of the adult. We found that higher maternal FA supplementation caused large-scale alterations in gene expression in the newborn’s cerebral hemispheres. Additionally we found moderate alterations in behavior in both the newborn and the adult as a result of higher FA exposure. To our knowledge, this is the first study of its kind to evaluate the effect of FA supplementation on global gene expression and its correlation with behavior.

## Materials and Methods

### Mice Strain and Feeding

This study was carried out in strict accordance with the recommendations in the Guide for the Care and Use of Laboratory Animals of the National Institutes of Health. The animal use protocol was approved by the Institute for Basic Research Institutional Animal Care and Use Committee (IBR IACUC), which oversees the use of laboratory animals for research purposes (IBR IACUC approval number 401). One week prior to mating, female mice were fed a custom AIN-93G amino acid–based diet (Research Diet, Inc. New-Brunswick, NJ, USA), receiving FA at 0.4 mg/kg (n = 20), while the test group received FA at 4 mg/kg (n = 20). The 0.4 mg/kg diet level of FA is necessary for normal healthy litter size [23], where as FA at the level of the 4 mg/kg diet is ten times higher. These amounts were chosen because in humans FA is prescribed at a dose of 400−800 µg/day to every pregnant woman, and to those planning to achieve pregnancy, and the recommended dose of FA is 10-fold higher for women with a previous history of NTD pregnancy (4 mg) [5].

**Figure 1:**
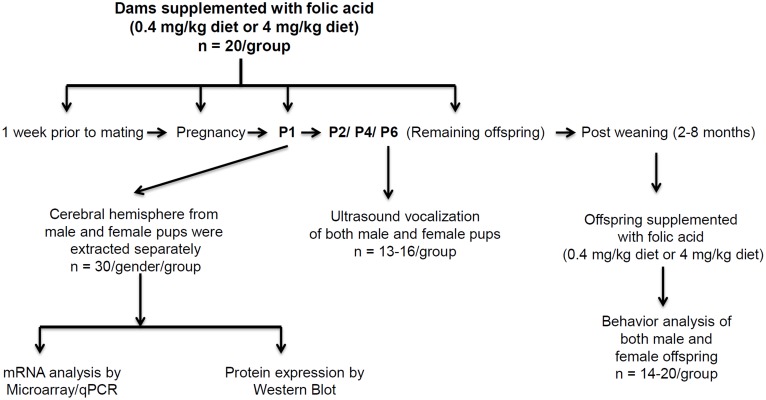
The schematic diagram of the experimental design for this study.

After mating, the females were separated and housed individually until the birth of their pups. At postnatal day one (P1), newborn pups from different dams were sacrificed, and cerebral hemisphere tissues were collected. For the 0.4 mg group, male pups’ n = 30 and female pups’ n = 30. Tissues from the 4 mg group (n = 30/gender) were similarly processed. All tissues were frozen in liquid nitrogen and from these, tissues were distributed for RNA and protein analyses. For the remaining offspring, FA diet was continued throughout the lifespan of the mice, and mice were either tested for ultrasonic vocalizations (USVs) at P2, P4 and P6 (n for 0.4 mg = 13; 4 mg = 16) or allowed to age with minimal interference for later behavioral testing. Adult male and female offspring were behaviorally tested between 2 and 7 months of age. When more than one male or female mouse was tested per litter, the data were pooled so that each litter is only represented by one male and one female score. Mice were housed 2−6/cage with *ad libitum* food (FA diets) and water, and 12-h light/dark cycle. The pups with the perinatal high FA treatment continued to receive 4 mg/kg FA diet after weaning till performance of the specified behavior tests. All experiments were conducted during the light phase between 10 am and 5 pm. These experiments were done throughout this time window, none were done only in the morning or the afternoon. The schematic diagram of the experimental design for this study is shown in Figure 1.

### RNA Preparation

Total RNA from P1 pups (n = 9/group/gender) was extracted with Trizol reagent (Life Technologies, Carlsbad, CA, USA) and further purified by Qiagen RNeasy kit (Qiagen, Valencia, CA, USA), according to the manufacturer’s instructions. Cerebral hemisphere tissues were pooled (n = 3/gender) for the 0.4 mg group: three male newborn pups (each from an independent dam) and three female newborn pups (each from an independent dam), and tissues from the 4 mg group were similarly processed. Considering the degree of inter-variability, RNA extractions were further repeated twice from a different batch (pooled samples, n = 3 for each group/gender, with each mouse from a different dam). RNA quality was assessed by measuring the absorbance ratio at 260/280 nm (Nanodrop 2000 spectrophotometer, Thermo Fisher Scientific, Waltham, MA, USA), and the integrity was assessed by formaldehyde-gel electrophoresis.

### Microarrays

Microarray analysis was carried out using pooled RNA from P1 pups as previously described [14]. Total RNA was converted to cDNA, followed by conversion to cyanine-3-labelled cRNA, using the one-color, low-input QuickAmp labelling kit (Agilent Technologies, Santa Clara, CA, USA) by following the manufacturer’s instructions. Purification of the cRNA was achieved with RNeasy kit (Qiagen,), and the incorporation of cyanine-3 was assessed by a Nano Drop spectrometer (Thermo Fisher Scientific, Waltham, MA, USA). A total of 600 ng of cRNA from each sample was fragmented and hybridized to the SurePrint G3 mouse gene expression 8×60 k arrays (G4852A) (Agilent Technologies). The data were processed by Feature Extraction software v10.7.3.1 for image analysis and initial quality control. Our Minimum Information About a Microarray Experiment (MIAME)−compliant data has been deposited in the Gene Expression Omnibus database of the National Center for Bioinformatics, with the accession number GSE45607 (http://www.ncbi.nlm.nih.gov/geo/query/acc.cgi?acc=GSE45607).

### Data Analysis

To increase statistical power and minimize differences of individual variability, samples were pooled from postnatal day one pups segregated by gender for each group [24]. A total of three microarray gene expression studies were performed for each group per gender, and each array represents expression of pooled n = 3/gender under the same dose of FA. The publicly available BRB ArrayTools 4.2.1−stable release was used for analyzing microarray data [25]. The expression data were normalized (quantile) and log-transformed. To select genes that changed significantly between the two doses of FA, the data sets were passed through a number of filtering steps based on intensity filter, *P* value of log intensity variation, and fold changes. For each spot, the intensity filter of threshold to minimum 10 was used if the intensity was below the minimum, and was further filtered by excluding the corresponding spots for an entire gene from all arrays having log intensity variations with a cut-off set at *P*>0.05. From these selected genes, a transcript was considered differentially expressed when the difference in the mean expression from three arrays was ≥ two-fold.

### Quantitative RT-PCR (qRT-PCR) analysis

Validation of microarray expression data was carried out by qRT-PCR for several randomly selected transcripts that were shown to have greater than two-fold change in expression. RT-PCR amplification was performed with iScript kit (BioRad, Hercules, CA, USA) by using 60−100 ng of total RNA from postnatal day one pups, by following the manufacturer’s instructions. The following RT-PCR cycle parameters were used: 50°C for 10 min, and 95°C for 5 min followed by 40 cycles of 95°C for 15 sec and 55°C for 1 min. Relative expression was calculated using the Pfaffl method [26]. Either *Hprt1* or *Gapdh* were used as an endogenous housekeeping control. The expression of these housekeeping genes was not changed as a result of FA treatment. The primers used in this study are listed in File S1, under Results and Table S1.

### Statistical Analysis

Each reaction was performed in triplicates and repeated twice, each from different batches of pooled samples (n = 3 pooled samples for each group/gender, each from different dams). Values are presented as means ± SD, and numerical results are presented considering *P*<0.05 as significant. Data were analyzed with Student t-test by using Prism software (GraphPad, La Jolla, CA, USA).

### Western Blot Analyses

Total cellular proteins from individual newborn pups' whole cerebral hemispheres (n = 2/gender/group) were used, and the detection of targeted proteins were accomplished by incubating with primary antibodies overnight at 4°C (anti-Slc17a7 1∶500, anti-Vgll2 1∶250) followed by horseradish peroxidase (HRP)−conjugated secondary antibody. The signal of the targeted protein was captured using the SuperSignal West Pico chemiluminescent substrate (Thermo Fisher Scientific, Waltham, MA, USA). Images were captured by using a Bioimager (UVP system, Upland, CA, USA), and analyzed by using ImageJ software (NIH).

### Behavior Analyses

Behavioral experimenters were blinded to the treatments and diet compositions that mice received. The researchers were provided with two groups of mice and two different diet pellets without disclosing the dietary content. Data generated as separate groups were again analyzed without prior knowledge of the diet composition. The experiments were run in the following order: postnatal day 2−6: USVs, after which the mice were allowed to age with minimal interference. Experiments started at 8 weeks of age and were run in the following order: social approach, elevated plus maze, marble-burying, self-grooming, buried food, trace fear conditioning, and open field. These experiments were conducted between 2 and 6 months of age. For each behavior test performed, all the mice were of similar age in all the groups tested. This behavioral phenotyping battery was chosen to assess the mice for aberrant behaviors in several psychological domains.

#### USV

For the USV experiments the data represent mice in the 0.4 mg group, n = 13 from 10 litters with 6 males and 7 females, and in the 4 mg group, n = 16 from 8 litters with 9 males and 7 females. Data were pooled so that no more than one female and male represents each litter. Individual P2, P4, and P6 pups were placed in a beaker inside of a Styrofoam box, which also contained an ultrasonic frequency detector set to 60 kHz. The box was closed to minimize outside noise. The frequency detectors were attached to a computer equipped with Ultravox software (Noldus, Attleboro, MA, USA) to analyze the number and duration of calls. Criterion for the number of calls was minimum call duration of 10 ms, with 5 ms of silence between vocalizations for each call to be considered independent. The pups were separated from their mothers and littermates for 5 minutes, with vocalization data recorded for the entire period of separation.

#### Social Approach

For the social approach experiments the data represent mice in the 0.4 mg group, n = 14 from 9 litters with 6 males and 8 females, and the 4 mg group, n = 16 from 9 litters with 8 males and 8 females. Data were pooled so that no more than one female and male represents each litter. Social approach behaviors were tested in an apparatus with three chambers in a single session, divided into three phases, as previously described [27], [28]. An upright plastic drinking cup weighed down with a lead weight was placed on top of each of the inverted wire cups to prevent the subject mouse from climbing on top. Both end chambers maintained a lighting level of 43−44 lux with two desk lamps angled away from the maze. More details are available in Methods S1 in File S1.

#### Trace Fear Conditioning

For the trace fear conditioning experiments, the data represent mice in the 0.4 mg group, n = 16 from 10 litters with 7 males and 9 females, and the 4 mg group, n = 16 from 10 litters with 9 males and 7 females. Data were pooled so that no more than one female and male represents each litter. Learning and memory abilities were assessed using a fear conditioning procedure as previously described [29]. A fear conditioning apparatus (ANY-maze, Stoelting, Inc., Wood Dale, IL, USA) equipped with a digital camera interfaced to a PC with ANY-maze software was used to score freezing. The fear conditioning experiment was conducted in three phases on three consecutive days: (1) trace fear training, (2) contextual fear conditioning, and (3) cued fear conditioning.

#### Open Field

For the open field experiments, the data represent mice in the 0.4 mg group, n = 15 from 9 litters with 6 males and 9 females, and the 4 mg group, n = 20 from 13 litters with 11 males and 9 females. Data were pooled so that no more than one female and male represents each litter. General exploratory locomotion in a novel environment was tested by placing mice in an open field for a 15-min test session. The open field was a transparent plexiglass apparatus (40 cm×40 cm×34.5 cm) with a grey metal floor. A camera was positioned above the apparatus to record the mice, and the videos were scored using video tracking software (ANY-maze). The testing room was illuminated to 45 lux and kept at a similar temperature as the colony room.

### Statistical Analysis for Behavioral Experiments

For the social approach task, repeated measures analysis of variance (ANOVA) was used to compare the outer chamber times. Time spent in the center chamber is shown graphically to illustrate where the subject mouse spent time during the entire 10-min phase. Chamber time, time spent sniffing the stranger mouse versus the novel object, and the number of entries to side chambers were recorded. Newman Keuls *post-hoc* analysis was run when the repeated measure (stranger mouse or novel object) was significant to determine the group differences. Groom time and time spent immobile were analyzed using factorial ANOVA and Newman Keuls *post-hoc* test. All other experiments were analyzed using factorial ANOVA, with the main factor being FA dose and Newman Keuls *post-hoc* tests. For all experiments, α was set at 0.05. The numbers of all animals (n’s) used for all experiments are in the figure legends.

## Results

### Microarray Analysis

We analyzed and compared the global gene expression pattern of the transcripts from the cerebral hemisphere of P1 pups whose mothers had received FA at two different concentrations throughout the pregnancy. Comparison of the expression data revealed that significant numbers of genes were altered in the pups from whose mothers had received FA at 4 mg/kg. The extent of extreme gene expression alteration was between 62-fold up-regulation in females and 18-fold down-regulation in males. MIAME−compliant data is available in the Gene Expression Omnibus database of the National Center for Bioinformatics, with the accession number GSE45607 (http://www.ncbi.nlm.nih.gov/geo/query/acc.cgi?acc=GSE45607).

The results showed that transcripts from 68 genes were down-regulated (Results Table S2 in File S1) and from 56 genes were up-regulated by more than two-fold (Results Table S3 in File S1) were common in both male and female pups from the 4 mg maternal FA group. Not only were genes altered by the amount of maternal FA, but there was also an interaction with FA and sexes. In male pups from the 4 mg maternal FA group, the expression patterns of over 1000 genes were down-regulated (Results Table S4 in File S1), and of over 900 genes were up-regulated (Results Table S5 in File S1) more than two-fold in comparison to the 0.4 mg maternal FA group. In female pups from the 4 mg maternal FA group, the expression patterns of over 900 genes were down-regulated (Results Table S6 in File S1) and of more than 1200 genes were up-regulated (Results Table S7 in File S1) more than two-fold. Several transcriptional factors, imprinted genes, and developmental genes were differentially regulated in the cerebral hemisphere of both male and female pups as a result of higher FA during gestational development. Thus, the results of this microarray analysis indicate that depending on the amount of maternal FA, the pattern of gene expression varies in the offspring’s cerebral hemisphere, and higher maternal FA could induce significant changes.

### FA-Mediated Differential Neural Gene Expression Profile in Newborns’ Cerebral Hemispheres

Because early brain development can influence long-term susceptibility to neuropsychiatric disorders, we analyzed the differential expression of neural genes (Tables 1 & 2). We found that expression of several genes involved in neural function were altered in the cerebral hemispheres of both male and female newborn pups born to females in the 4 mg FA group. Neurotransmitter genes (*Chrm4*, *Htr3a* and *Htr4*), genes related to glutamatergic synapses (*Avp*, *P2rx7*), dopaminergic and serotoninergic pathway genes (*Cyp2d22*, *Nr4a3*) and neurogenesis related genes (*S100b*, *Shh*) were down-regulated by more than two-fold in the cerebral hemispheres of male newborn pups receiving the 4 mg FA dose (Table 1). In female newborn pups from the 4 mg FA group, genes related to the dopamine-serotonin pathways (*Slc6a4*, *Slc18a2*), synaptic plasticity (*Srf*), GAP-junctions (*Tuba1b*), and the neurotrophin receptor (*Nrg4*) were down-regulated by more than two-fold (Table 2). The synaptic motor protein kinesin 17 (*Kif17*) in male newborn pups and nitric oxide synthase (*Nos1*) transcripts in female newborn pups were significantly up-regulated (Tables 1 & 2). Additionally, 4 mg FA caused a significant up-regulation of the neurotransmitter *Gabra6* in the cerebral hemispheres of both male and female newborn pups (Tables 1 & 2). These results indicate that the differences in the amount of maternal FA during gestation can modulate expression of neural genes in the offsprings’ cerebral hemispheres.

**Table 1:** Neural genes altered ≥2 fold in the cerebral hemisphere of male pups from mothers having FA supplementation during gestation at 4 mg/kg in comparison to mothers at 0.4 mg/kg diet.

Accession	Symbol	Fold Change	Direction of Change	Pathway
ref|NM_008313	Htr4	−3.70	↓	Dopa-Seretonin
ref|NM_013561	Htr3a	−3.67	↓	Dopa-Seretonin
ref|NM_019823	Cyp2d22	−3.04	↓	Dopa-Seretonin
ref|NM_015743	Nr4a3	−2.60	↓	Dopa-Seretonin
ref|NM_001038845	P2rx7	−3.96	↓	GABA-Glutamate
ref|NM_009732	Avp	−3.77	↓	GABA-Glutamate
ref|NM_007699	Chrm4	−3.12	↓	Neuro-transmitter
ref|NM_009170	Shh	−3.49	↓	Neurogenesis
ref|NM_009115	S100b	−2.99	↓	Neurogenesis
ref|NM_010291	Gjb5	−2.50	↓	Gap junctions
ref|NM_008123	Gja8	−2.07	↓	Gap junctions
ref|NM_019827	Gsk3b	3.27	↑	Dopa-Seretonin
ref|NM_009414	Tph1	2.48	↑	Dopa-Seretonin
ref|NM_173391	Tph2	2.14	↑	Dopa-Seretonin
ref|NM_013761	Srr	2.35	↑	GABA-Glutamate
ref|NM_182959	Slc17a8	2.30	↑	GABA-Glutamate
ref|NM_008068	Gabra6	2.15	↑	GABA-Glutamate
ref|NM_010623	Kif17	2.43	↑	Synaptic plasticity
ref|NM_008689	Nfkb1	2.25	↑	Synaptic plasticity
ref|NM_001042528	Cacna1b	2.42	↑	Neuronal-Ion Channels
ref|NM_145983	Kcna5	2.11	↑	Neuronal-Ion Channels
ref|NM_008426	Kcnj3	2.09	↑	Neuronal-Ion Channels
ref|NM_018732	Scn3a	2.07	↑	Neuronal-Ion Channels
ref|NM_177781	Trpa1	2.00	↑	Neuronal-Ion Channels
ref|NM_021382	Tacr3	2.20	↑	Neurotransmitter
ref|NM_009216	Sstr1	2.17	↑	Neurotransmitter
ref|NM_008312	Htr2c	2.12	↑	Neurotransmitter
ref|NM_001001496	Gja6	2.07	↑	Gap Junctions
ref|NM_172989	Lpar1	2.03	↑	Gap Junctions
ref|NM_008737	Nrp1	2.30	↑	Neurogenesis

↑ = Up regulated.

↓ = Down regulated.

**Table 2:** Neural genes altered ≥2 fold in the cerebral hemisphere of female pups from mothers having FA supplementation during gestation at 4 mg/kg in comparison to mothers at 0.4 mg/kg diet.

Accession	Symbol	Fold Change	Direction of Change	Pathway
ref|NM_172523	Slc18a2	−3.14	↓	Dopa-Seretonin
ref|NM_010484	Slc6a4	−2.84	↓	Dopa-Seretonin
ref|NM_198190	Ntf5	−3.30	↓	Neurotrophin receptor
ref|NM_032002	Nrg4	−2.28	↓	Neruotrophin receptor
ref|NM_145129	Chrna3	−2.13	↓	Neurotransmitter
ref|NM_001037712	Kcnh6	−2.09	↓	Neuronal-Ion Channels
ref|NM_020493	Srf	−2.98	↓	Synaptic plasticity
ref|NM_011654	Tuba1b	−3.32	↓	Gap junctions
ref|NM_011198	Ptgs2	3.83	↑	Dopa-Seretonin
ref|NM_138942	Dbh	2.40	↑	Dopa-Seretonin
ref|NM_019911	Tdo2	2.12	↑	Dopa-Seretonin
ref|NM_008068	Gabra6	12.82	↑	GABA-Glutamate
ref|NM_182993	Slc17a7	4.81	↑	GABA-Glutamate
ref|NM_008066	Gabra2	4.02	↑	GABA-Glutamate
ref|NM_205783	Chrm5	4.29	↑	Neuro-transmitter
ref|NM_007726	Cnr1	3.30	↑	Neuro-transmitter
ref|NM_009217	Sstr2	2.39	↑	Neuro-transmitter
ref|NM_007578	Cacna1a	2.34	↑	Neuronal-Ion Channels
ref|NM_008427	Kcnj4	2.20	↑	Neuronal-Ion Channels
ref|NM_013627	Pax6	2.25	↑	Neurogenesis
ref|NM_144939	Frs3	2.60	↑	Neruotrophin receptor
ref|NM_008712	Nos1	2.17	↑	Synaptic plasticity
ref|NM_080450	Gjc3	2.07	↑	Gap Junctions

↑ = Up regulated.

↓ = Down regulated.

### Quantitative RT-PCR Confirmed Alteration of Gene Expression as a Result of High Maternal FA

**Figure 2:**
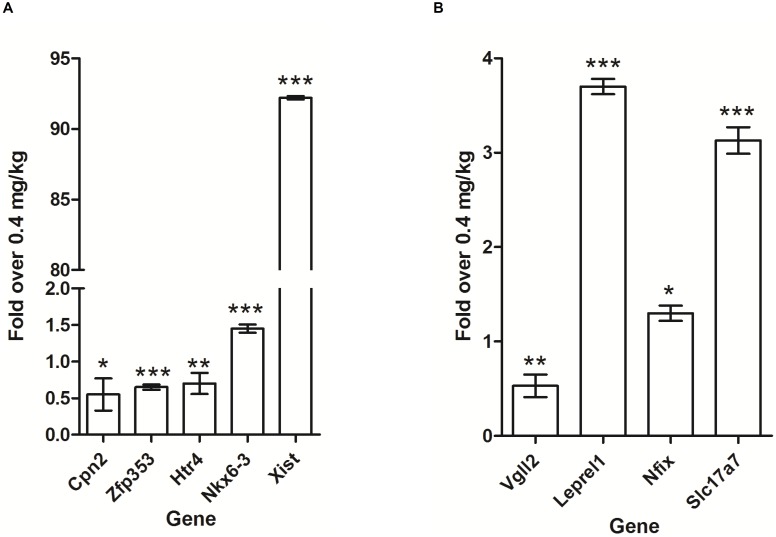
A: Quantitative RT-PCR confirmation of microarray data. Relative expression of the *Cpn2*, *Zfp353*, *Htr4*, *Nkx6-3* and *Xist* transcripts in the cerebral hemispheres of male newborn pups, from mothers supplemented with FA at 4 mg/kg of diet. The results were normalized to *Hprt* transcript expression, and expressed as relative values in comparison to corresponding transcripts from newborn pups with FA at 0.4 mg/kg diet. Results represent means ± S.D; asterisks denote statistically significant change (***P<0.001, **P<0.01 and *P<0.05). **B**): **Quantitative RT PCR confirmation of microarray data**. Relative expression of the *Vgll2*, *Leprel1*, *Nfix* and *Slc17a7* transcripts from the cerebral hemispheres of female newborn pups, from mothers supplemented with FA at 4 mg/kg of diet. The results were normalized to *Gapdh* transcript expression, and were expressed as relative values in comparison to corresponding transcripts from newborn pups with FA at 0.4 mg/kg diet. Results represent means ± S.D; asterisks denote statistically significant change (***P<0.001, **P<0.01 and *P<0.05).

To validate the microarray results, qRT-PCR was performed for nine randomly selected genes (*Xist*, *Nkx6-3*, *Leprel1*, *Nfix*, *Slc17a7, Cpn2, Htr4, Zfp353, and Vgll2*) whose expressions were altered in male or female newborn pups from 4 mg of maternal FA. The housekeeping gene *Hprt* was used as an internal control in male newborn pups, and *Gapdh* was used as an internal control in female newborn pups. Expression of both these genes did not show differences between the two diet groups. The results of qRT-PCR (Figures 2A & 2B) showed a similar expression directional pattern and agreed well with the results of the microarray experiments. In male newborn pups, the carboxypeptidase N, *Cpn2*; the zinc finger protein, *Zfp353*; and the serotonin receptor, *Htr4*; and in female newborn pups, the vestigial-like 2 gene, *Vgll2*, were significantly (*P*<0.05) down-regulated (Figures 2A & 2B). The imprinted gene, *Xist*, and the homeobox gene, *Nkx6-3*, were up-regulated significantly (*P*<0.001), in the cerebral hemispheres of male newborn pups. Moreover, in female newborn pups from 4 mg maternal FA supplementation, *Leprel1* that encodes an enzyme critical for collagen chain assembly; *Nfix* that encode transcriptional factor; and sodium-dependent phosphate transporter, *Slc17a7*, were significantly (*P*<0.05) up-regulated (Figure 2A & 2B). These qRT-PCR results provided further evidence that the high amounts of maternal FA results in alterations in the gene expression in newborn’s cerebral hemispheres.

### Western Blot Analysis Confirmed High Maternal FA Alters the Expression of Slc17a7p and Vgll2p in Newborn Pups Cerebral Hemisphere

**Figure 3:**
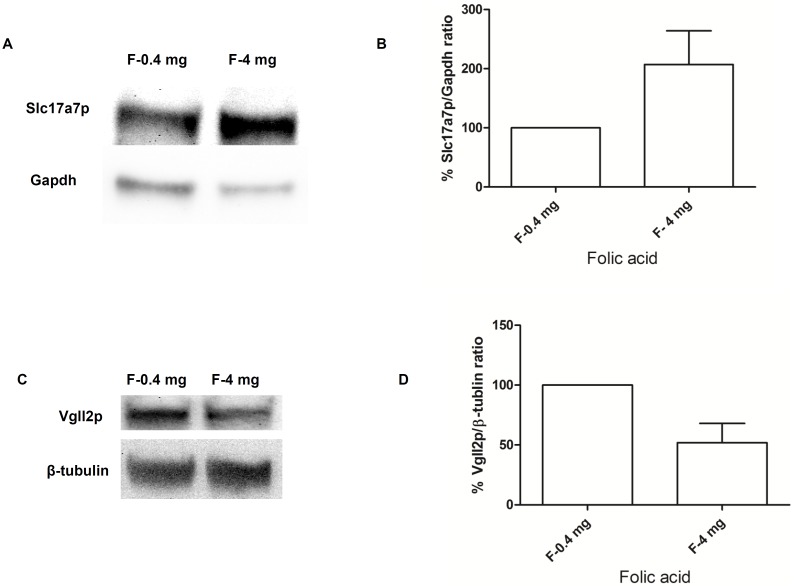
High maternal FA alters the expression of Slc17a7p and Vgll2p in the cerebral hemispheres of female newborn pups. Total cell lysates from the cerebral hemispheres of female (F) newborn pups were prepared from both 0.4 mg/kg and 4 mg/kg FA diet groups and proteins were resolved on 10% Tris/HEPES/SDS-polyacrylamide gels. The blot was probed with Slc17a7p antibody (**A**, **B**) or with Vgll2p antibody (**C**, **D**). To confirm equal protein loading, the membranes were re-probed with GAPDH/β-tubulin antibodies. The left panel represents one representative blot and the right panel shows densitometric evaluation of two independent experiments. Densitometric evaluation of the bands were calculated by using the Image J software and normalized to the densities of GAPDH//β-tubulin. The values corresponding to 0.4 mg/kg FA diet were arbitrarily set to 100%, and the 4 mg/kg values are presented relative to this. The densitometric values represent the mean, and the error bar represents the inter-variability among independent experiments.

In order to confirm that the gene expression changes observed in microarray analysis and qRT-PCR are translated to protein functions, we examined whether the alterations in the expression of *Slc17a7* and *Vgll2* transcript correlate with the expression of protein level. Western blot analysis and quantification of the protein levels using ImageJ software (NIH) revealed a significant up-regulation in the expression of Slc17a7p (Figures 3A & 3B) and reduction in the expression of Vgll2p (Figures 3C & 3D) in female newborn pups whose mothers received high maternal FA. These results exhibited that the high maternal FA results in alterations in gene expression in newborns’ cerebral hemispheres. Moreover, they further confirmed that the differential gene expression observed in microarray analysis and qRT-PCR as a result of different amounts of maternal FA are similarly reproduced at the protein level.

### Higher FA Supplementation during Pregnancy and throughout Life Moderately Modifies Behavior

In order to test whether the dose of maternal FA may lead to behavior modifications, we first studied the behavior of mice pups whose mothers were fed FA at 0.4 or 4 mg/kg diets for USVs. Then pups continued receiving FA diets throughout the lifespan and were tested for sociability, anxiety, obsessive-compulsiveness, and learning and memory during adulthood (starting at 8 weeks of age) in order to assess the behavioral outcome as a result of the cumulative exposure of gestational and lifelong FA (Results Table S8 in File S1).

#### USVs

**Figure 4:**
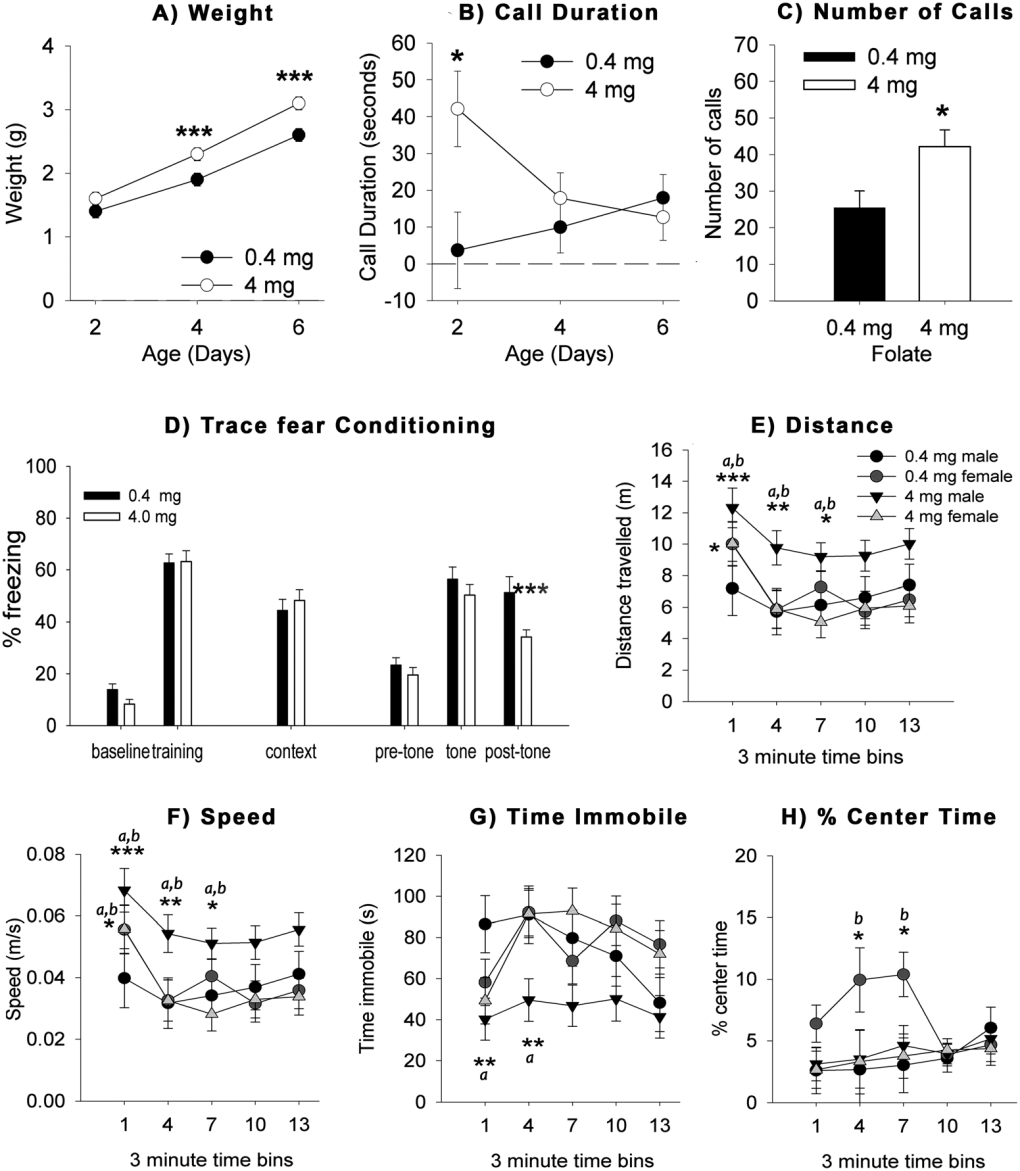
FA supplementation during gestational development and post-weaning period modifies behavior. *Ultrasonic vocalizations (panels* ***A–C*** *)*. Juvenile mice were recorded for 5 min upon separation from their dams. There were no differences between the sexes in any of these measures. **A**) The mice exposed to FA at 4 mg/kg weighed more on P4 and P6 than the 0.4 mg/kg-exposed mice. **B**) The mice exposed to FA at 4 mg/kg made longer calls on P2, but not on P4 and P6. **C**) The mice exposed to FA at 4 mg/kg made significantly more calls than the 0.4 mg/kg group. **D**) *Trace fear conditioning*. FA did not affect learning and memory in mice. The graph presents the group averages for the percentage of time spent freezing from the different stages of the experiment. The 4 mg/kg group froze less after the tone ended during the cued fear conditioning (0.4 mg/kg n = 16; 4 mg/kg n = 16). *Open field*. **E**) The 4 mg/kg males travelled the longest distance in each 3 min bin across the 15 min compared to the other sexes and treatment groups. The 0.4 mg/kg males travelled the least distance at the beginning of the test and became less mobile as the test progressed. Both the 0.4 and 4 mg/kg females were more active at the beginning and spent more time immobile later in the test period. a: significantly (*P*<0.05) different than the 0.4 mg/kg males; b: significantly different (*P*<0.05) than the 4 mg/kg males. **F**) The total distance travelled by the 4 mg/kg males over the entire 15 min was significantly greater than the 0.4 and 4 mg/kg females, but not the 0.4 mg/kg males (0.4 mg/kg, males n = 6; females n = 9; 4 mg/kg, males n = 11, females n = 9). **G**) Overall time immobile differed across the experiment. The 4 mg/kg males spent significantly less time immobile during the first two time bins compared to the 0.4 mg/kg males. There were no differences between the female FA groups. **H**) There were no effects of FA or time bin on percent center time; however, the 0.4 mg/kg females spent a significantly greater percentage of time in the center compared to the 0.4 mg/kg males in the second and third time bins. Open field asterisks denote significant differences from the ^a^low versus high FA groups, and between ^b^males versus females. For all graphs: *P<0.05; **P<0.01; ***P<0.001 was used.

All of the pups had increases in weight across P2 to P6, and the males weighed significantly more than the female pups overall, but sex did not interact with the FA dose or age. The 4 mg/kg pups weighed significantly more than the 0.4 mg/kg pups overall, which interacted with age. *Post-hoc* analysis revealed that the 4 mg/kg pups weighed significantly more on P4 and P6 but not P2 (Figure 4A). The offspring were tested for USVs in response to maternal separation on P2, P4, and P6. The 4 mg/kg pups had a longer total duration of calls than the 0.4 mg/kg pups (Figure 4B). There was no difference between the sexes or age of the pups. There was an interaction between the dose of FA and the age of testing as the 0.4 mg/kg pups' call duration was the longest at P2 and decreased across age, while the 4 mg/kg pups had their shortest call duration on P2, and the call duration gradually increased until P6. This finding was confirmed by *post-hoc* analysis of the interaction between age and FA dose, which revealed a difference between the FA groups on P2, where the 4 mg/kg pups made longer calls than the 0.4 mg/kg pups, but not on P4 and P6. The mice exposed to FA at 4 mg/kg diet *in utero* and throughout the experiment emitted more USVs than did the 0.4 mg/kg group (Figure 4C). This finding did not differ by the sex or the age of the pups. There were no interactions of FA dose, age of testing, or sex.

#### Trace Fear Conditioning

Figure 4D shows the percent freezing data from the trace fear conditioning test. FA dose did not affect freezing behavior during training or contextual fear conditioning, but there was an increase in freezing after the cue was presented in the 0.4 mg/kg mice. All of the mice froze significantly more following training than during baseline. There were no differences in freezing on the training day between the 0.4 and 4 mg/kg FA groups. On the next day when the animals were placed in the same context without the tone or shock, both FA groups froze to a similar extent. On day 3, the mice were placed into a novel context and exposed to the tone. All of the mice froze more when the tone was on than during the pre-tone or post-tone time periods. FA dose did affect freezing levels overall. There were no differences between the FA groups during the baseline or when the tone was on. However, the 4 mg/kg FA mice froze significantly less in the post-tone period than did the 0.4 mg/kg mice. There was no effect of sex in any of the measures (details in Results S1 in File S1).

#### Open Field

Figure 4, panels' E-H illustrate the data from the open field exploration test. Mice were placed into the open field, and time immobile, distance travelled, speed and center time were recorded across 15 min. Male mice from the dams fed the 4 mg/kg diet showed increased hyperactivity at the beginning of the test. The distance traveled was highest at the beginning of the test and dropped as shown by a significant effect of time bin (Figure 4E). *Post-hoc* analysis revealed that the 4 mg/kg males travelled a significantly greater distance than the 0.4 mg/kg males during the first three time bins. There were no differences between the female mice on distance traveled. The data for the speed of the mice were very similar, as the mice travelled at a greater speed at the beginning of the test (Figure 4F). This was shown by an effect of time bin. *Post-hoc* analysis revealed that the 4 mg/kg males moved at a greater speed than the 0.4 mg/kg males during the first three time bins. There was no difference between the females in the speed measure. Overall time immobile differed across the experiment (Figure 4G). *Post-hoc* analysis of the treatment groups by sex showed that the 4 mg/kg males spent significantly less time immobile during the first two time bins compared to the 0.4 mg/kg males. There were no differences between the female FA groups, nor there were any effects of FA or time bin on percent center time (Figure 4H). This was shown by lack of a significant effect of time bin. *Post-hoc* analysis revealed that the 0.4 mg/kg females spent a significantly greater percentage of time in the center compared to the 0.4 mg/kg males in the second and third time bins. There were no differences within the females or males due to FA dose.

#### Social Approach, Elevated Plus Maze, Spontaneous Grooming, Marble Burying and Buried Food

In the social approach test, offspring from both doses of FA demonstrated social behavior with a significant preference for the chamber with the stranger mouse and time spent sniffing the stranger mouse (File S1). Neither treatment elicited greater levels of anxiety-like behavior, as shown by no difference in percent open arm time or percent open arm entries. There was no difference in repetitive grooming between the 0.4 mg/kg and 4 mg/kg groups, or between the sexes, or interaction between treatment and sex. There was no difference between the FA groups on obsessive-compulsive like behavior, as shown by the number of marbles buried (File S1, Table S8), which was not affected by sex and FA. Neither group showed an olfactory deficit in finding the buried food (File S1, Table S8). There was no difference between the sexes and sex did not interact with the FA dose.

## Discussion

Fetal gestational development is controlled by precise regulation of gene expression, and epigenetics plays a crucial role in regulating gene expression during development. Recently we reported that maternal FA during gestation induces a substantial alteration in methylation patterns (both hypo- and hyper-) in a site-specific manner both in CpG and non-CpG contexts throughout the genome of offspring cerebral hemispheres [15]. In the current study, we have found that changes in the amount of FA in the maternal diet during gestation dysregulates gene expression in the newborns' cerebral hemispheres, and gestational and lifelong exposure to higher levels of FA moderately modified the pups' behavior. Key developmental genes including those involved in neural pathways, neurotransmitters, neuronal-ion channels, GABA and glutamatergic-synapse, dopamine-serotonin and synaptic plasticity which may modulate the behavior of offspring, exhibited alterations in expression due to the higher level of FA supplementation. For example, *Kif17*, which plays an important role in the transport of vesicles containing the *N*-methyl-D-aspartate receptor and the K+ channel subunit Kv4.2 to dendrites in mammals [30], [31], was up-regulated by more than two-fold in the cerebral hemispheres of male newborn pups from mothers that received higher FA diets. Because the kinesin superfamily of proteins play important roles in receptor transportation, and *Kif17* contributes to neuronal development, such alteration of *Kif17* expression may modulate synaptic plasticity in the offspring brain. Likewise, female newborn pups from higher maternal FA supplementation exhibited an up-regulation of the *Nos1*. In this regard, a recent study has shown that schizophrenic patients show significant up-regulation of *Nos1* in their dorsolateral-precerebral cortices [32]. Consequent to changes in gene expression, we found a moderate alteration in behavioral outcome when offspring were assessed behaviorally. Pups from dams exposed to a higher dose of FA during gestation and lactation made increased USVs as neonates; these behaviors may have originated from FA induced alteration of gene expression during the gestational period. Increased USVs have been found in mouse models for autism [33], [34]. Anxiety symptoms are highly prevalent in children with developmental disorders, including autism [35]. Of note, our result of microarray and qRT-PCR shows significant down-regulation in the expression of *Htr4* in the cerebral hemispheres of male newborn pups of higher maternal FA. *Htr4* is considered as a putative candidate gene for autism and was found to be differentially expressed in lymphoblastoid cell lines from individuals with autism spectrum disorders [36], [37]. *Htr4* is known to modulate the release of neurotransmitters, and irregularities in serotonergic neurotransmission have been linked to a variety of neuropsychiatric diseases [38]. Alterations in *Htr4* expression as a result of higher FA during gestational development may play a role in the genetic predisposition to psychiatric diseases. Similarly, the expression of *Slc17a7* was up-regulated significantly both at the mRNA and the protein levels in female newborn pups born to mothers that received a high FA diet. *Slc17a7* is known to be expressed mainly in the axon terminals of neocortical neurons, and to critically modulate the efficacy of glutamatergic synaptic transmission [39]–[43]. Several studies have shown that alterations in expression of vesicular glutamate transporters impact various brain disorders including schizophrenia, Parkinson's disease and Alzheimer's disease; moreover, a recent study has shown increased expression of *Slc17a7* in the white matter of schizophrenic patients [44], [45]. Considering the functional role of *Slc17a7* in synaptic plasticity, the up-regulation in the expression of *Slc17a7* in cerebral hemispheres of female newborn pups as a result of high maternal FA may cause consequent changes in behavior as observed in this study. However, behavior modification may result from a cumulative effect of high FA during gestational development as well as post-weaning period.

Furthermore, the qRT-PCR data also showed alterations in the expression of *Nfix* and *Vgll2* in female newborn pups and *Nkx6–3* in male newborn pups from high maternal FA. The expression of *Nfix* that was shown to play a central role in hippocampal morphogenesis [46], was significantly up-regulated, and the expression of *Vgll2* was down-regulated in female newborn pups from mothers fed higher FA diets. The biological consequences of such down-regulation in the expression of *Vgll2* may have far-reaching implications, as vestigial-like genes are reported to modulate cell fate and embryonic patterning [47]. *Nkx6-3*, which controls numerous developmental processes including the central nervous system, was also up-regulated in male newborn pups from mothers fed higher FA diets. We speculate that mechanistically such changes in gene expression may be modulated more in a site- and gene-specific manner with the methylation of individual CpGs due to high maternal FA. These gene expression changes could be the direct result of methylation of specific cytosine residues, both in CpG and other contexts, as we have recently reported [15]. Methylation data is available in the NCBI SRA with accession number SRX482108. Cytosine methylation may affect the gene expression either in cis-configuration affecting expression of the same gene and/or reading frame, or methylation of one gene may modulate expression of another gene elsewhere in the genome in a trans-configuration. While it is a logical conclusion that gene expression changes during gestational development may have contributed to the changes in behaviors, more direct evidence is necessary. These conclusions, however, will have to be confirmed by direct sequencing methods. We also cannot exclude alternative mechanisms, as DNA methylation is also modulated by other factors such as methylenetetrahydrofolate reductase (MTHFR) activity, and is not restricted on folate status alone [48], [49].

Next, we analyzed whether the amount of FA received during gestation and throughout the lifespan impacts the behavior of offspring. The results of our behavioral studies have shown that higher FA exposure resulted in males being more active in the open field during adulthood. This finding is reflected in the open field test, which measures the general exploratory behavior of mice in a novel environment in which the males in the higher FA group travelled a greater distance. In this context, we found that in male newborn pups, the expression of the imprinted gene *Xist* was up-regulated by 90 fold. *Xist* is well known for its actions in transcriptional silencing and plays an important role in developmental components by modulating dosage compensation [50], [51]. Moreover, previous studies have shown that *XIST* is over-expressed in males diagnosed with Klinefelter syndrome compared to XY controls, suggesting a silencing of many genes on the X chromosome, resulting in dysregulation of X-linked gene expression [52]. Gender differences observed in gene expression alteration could be the result of genomic imprinting and X-inactivation. Some of the gender-related differences in gene alteration may explain the differences in behaviors between the males from the higher-dose group being more active than females. Of note, some of the behavior resulting from higher FA that we found in this study may have been caused by alterations in methylation levels, as aberrant DNA methylation is associated with reduced neurogenesis and increased genomic instability, and has been linked to cognitive impairments, including fragile X and Rett syndromes [53]–[55]. However, this study found no differences in social behavior or learning and memory between the two groups. Thus, the result of the behavioral study indicates that gestational and lifelong exposure to higher levels of FA may impact some behavioral changes, if not drastic alterations. One caveat of our experimental design is that it continued a FA-rich diet during the post-weaning period. Therefore, it cannot be determined whether the changes in behavior observed in this study are due to excess FA during gestational/lactational development or to continued postnatal supplementation. In the future, studies of FA-rich diets restricted to during gestational development or stopped upon closure of the neural tube should clarify this aspect, as well as ascertain whether excess FA-induced unintended changes can be minimized or eliminated.

## Conclusions

In view of a recent report that FA consumed by pregnant women four weeks prior to and 8 weeks after conception in Norway contributes to a lower risk of autism [56], it is clear that the timing of FA supplementation is critical. There is also the possibility of genetic predisposition that would make certain at-risk populations more susceptible to changes in dietary epigenetic factors. Thus, autism may have genetic predisposition, which is exacerbated with environmental contributors such as epigenetic factors. Additionally, a recent study in rodents has shown that FA may be useful in clinical interventions to promote brain and spinal cord healing; however higher dose-induced decreased axonal regeneration, which may be associated with reduced *de novo* methyltransferase levels, indicates a ceiling on the regenerative potential provided by FA supplementation [57]. We suggest that moderation of FA supplementation be exercised during gestation and during the life span, as alterations of nutrient intake may modulate susceptibility to various diseases. Our findings demonstrate both that FA is an important contributing factor that can A) alter global gene expression, B) epigenetically reprogram brain structure, and C) affect its function, and that there may be a loss of benefit from higher amounts of FA. Even though numerous genes were identified as being altered in our study, it is unlikely that all these genes will have adverse consequences as shown by the behavior analysis of our data (no differences in social behavior or learning and memory). In the future, it will be of interest to determine whether the alteration of gene expression has a combined, cumulative influence in reprogramming of the normal development of the brain.

## Supporting Information

File S1 File contains additional Methods S1 and Results S1 associated with the behavioral study, as well as several large microarray data Tables that are referenced in the text.
(DOCX)
