# Supplementary material for: Increasing Maternal or Post-Weaning Folic Acid Alters Gene Expression and Moderately Changes Behavior in the Offspring
Source: PLoS One. 2014 Jul 9;9(7):e101674. doi: 10.1371/journal.pone.0101674 (PMC4090150; doi:10.1371/journal.pone.0101674)
Supplement: File S1 — File contains additional Methods S1 and Results S1 associated with the behavioral study, as well as several large microarray data Tables that are referenced in the text. (DOCX) [file pone.0101674.s001.docx]

**METHODS S1**

Social Approach: Social approach behaviors were tested in an apparatus with 3 chambers in a single 25 minute session, divided into 3 phases, as previously described [1,2]. This experiment had two habituation phases (center and all 3 chambers) followed by one testing phase (sociability). The sociability test compared the preference for a social stimulus versus an inanimate object. The subject mouse was acclimated to the apparatus for 5 minutes in the center chamber only, and then for an additional 10 minutes with access to all 3 empty chambers. The subject was then confined to the middle chamber, while the novel object (an inverted wire cup, Galaxy Cup, Kitchen-Plus, Streetsboro, OH) was placed into one of the side chambers, and the stranger mouse, inside an identical inverted wire cup, was placed in the opposite side chamber. The location (left or right) of the novel object and stranger mouse alternated across subjects. The chamber doors were opened simultaneously, and the subject had access to all 3 chambers for 10 min. Video tracking with ANY-maze (Stoelting, Inc.; Wood Dale, IL) automatically scored the time spent in each of the 3 chambers, time spent sniffing the novel object or stranger mouse, and number of entries into each chamber during each 10-minute phase of the test.

Animals used as strangers were male or female C57BL/6J mice habituated to the testing chamber for 30-minutes sessions on 3 consecutive days and were enclosed in the wire cup to ensure that all social approach was initiated by the subject mouse. An upright plastic drinking cup weighed down with a lead weight was placed on top of each of the inverted wire cups to prevent the subject mouse from climbing on top. Both end chambers were maintained at a lighting level of 43-44 lux with 2 desk lamps angled away from the maze.

Marble Burying: Obsessive compulsive-like behavior was tested using the marble burying procedure described by [3]. Mice were placed individually in housing cages (28 x 28 x 13 cm) filled with 5 cm of cob bedding and covered with a filter top for 30 min. Each cage contained 20 black glass marbles that were evenly positioned in 4 x 5 rows in a grid. At the end, the mice were returned to their home cage and the number of buried marbles, defined by being more than 2/3 covered, were counted and photographed.

Elevated Plus Maze: Anxiety-like behavior was tested in the elevated plus maze as previously described [1,2]. The elevated (95 cm) plus maze consists of 2 open arms (30 x 5 cm x 0.25 cm) and 2 closed arms (30 x 5 x 15 cm) extending from a central (5 x 5 cm) area. Mice were placed in the central area facing an open arm and allowed to traverse the maze freely for 5 min. Arm entries (70% of mouse in the arm) and time spent in the open and closed arms were tracked and scored using ANY-maze software (Stoelting, Inc.). The open arms of the maze were lighted to 78-79 lux and the closed arms to 58-60 lux with 2 desk lamps angled away from the maze.

Self-grooming: Mice were scored for spontaneous grooming behavior. Each mouse was placed individually into a standard mouse cage. After a 10-minute habituation period in the test cage, each mouse was scored with a stopwatch for 10 minutes for cumulative time spent grooming all body regions.

Buried Food: Olfactory sensitivity was measured using the buried food test as previously described [4]. Several days before the test, an unfamiliar food (Teddy Grahams, Kraft Foods, Northfield, IL) was placed overnight in the home cages of the subject mice to avoid food neophobia on the day of testing. Cages were checked for uneaten cookie the following day. Most of the mice began eating the cookie in a short amount of time, and cookies were consumed in all home cages. Mice were food deprived for 20 hours prior to testing. Testing was conducted in a clean empty mouse cage containing clean home cage bedding 3 cm deep. Mice were allowed to explore for 5 minutes and then removed from the cage. A cookie was then buried 1 cm deep in the cage bedding. The subject mouse was returned to the cage and the latency to dig for the buried cookie was measured with a 15 minute cutoff.

**RESULTS S1**

Social Approach. Table S8 illustrates the sociability scores from the three chambered social approach task from the 0.4 or 4 mg FA groups. Sociability, defined as more time spent in the chamber with the stranger mouse than the chamber with the novel object, and did not differ by amount of FA in the diet. Both the 0.4 mg and 4 mg groups demonstrated a significant preference for spending time with a stranger mouse over a novel object (within subjects chamber time, F_1,26_ = 132.43, *P*<0.001) regardless of sex (within subjects chamber time by sex, F_1,26_ = 0.15, NS). Post-hoc analysis revealed that mice in both conditions spent significantly more time in the chamber with the stranger mouse compared to the novel object (*P*’s <0.001). Similarly, the time spent sniffing the stranger mouse was significantly greater than the time sniffing the novel object overall (within subjects sniff time in either chamber, F_1,26_ = 142.41, *P* <0.001) and with each folate dose group (*P*’s < 0.001) regardless of sex (within subjects sniff time in either chamber by sex, F_1,26_ = 0.43, NS). The mice made significantly more entries into the chamber with the stranger mouse (within subjects entries in either chamber, F_1,26_= 31.49, *P* < 0.001). FA dose did affect the overall number of entries (within subjects entries by folate dose, F_1,26_= 7.90 , *P* < 0.01) but both the mice treated with both doses made more entries into the chamber with the strange mouse (*P*’s < 0.05). Sex did not interact with FA dose (within subjects entries by sex, F_1,26_ = 1.21, NS.

Elevated Plus Maze. Anxiety-like behavior in the mice is shown by their performance on the elevated plus maze. There were no differences in anxiety-like behavior as shown by no difference in percent open arm time (F _1,18_ = 0.10; NS) or percent open arm entries (F _1,18_ = 0.03; NS). There was no effect of sex (F_1,18_ = 0.19, NS) or interaction between FA and sex (F_1,18_ = 0.00, NS) on percent open arm time, or sex on percent open arm entries (F_1,18_ = 0.06, NS) and sex did not interact with FA (F_1,18_ = 0.26, NS). The 4 mg FA mice almost made significantly more total entries arms of the maze (F_1,18_ = 4.41, *P* = 0.0501) indicating more locomotor activity independent of anxiety-like behavior. There was no effect of sex (F_1,18_ = 0.69, NS) or interaction between FA and sex (F_1,18_ = 2.40, NS).

Trace Fear Conditioning. There was no difference during baseline and training between the sexes (F_1,29_= 0.06, NS), or interaction between sex and FA dose (F_1,46_= 0.86, NS). During contextual fear conditioning there was no difference between the sexes (F_1,29_= 0.40, NS) and sex did not interact with FA dose (F_1,29_= 4.11, NS). During cued fear conditioning there was no difference between the sexes (F_1,29_= 0.10, NS) and sex did not interact with FA dose (F_1,29_= 2.14, NS).

Reference List

1. Chadman KK, Gong S, Scattoni ML, Boltuck SE, Gandhy SU, et al. (2008) Minimal aberrant behavioral phenotypes of neuroligin-3 R451C knockin mice. Autism Res 1: 147−158.

2. Chadman KK (2011) Fluoxetine but not risperidone increases sociability in the BTBR mouse model of autism. Pharmacol Biochem Behav 97: 586−594.

3. Bruins Slot LA, Bardin L, Auclair AL, Depoortere R, Newman-Tancredi A (2008) Effects of antipsychotics and reference monoaminergic ligands on marble burying behavior in mice. Behav Pharmacol 19: 145−152.

4. Yang M, Crawley JN (2009) Simple behavioral assessment of mouse olfaction. Curr Protoc Neurosci Chapter 8: Unit 8.24. doi: 10.1002/0471142301.ns0824s48.

**Table S1**: List of primers used for qRT-PCR in this study to validate the microarray data.

| **Name** | **Sequences** |
| --- | --- |
| Cpn2-Forward | GGAGTGAGACCCAGCACAAC |
| Cpn2-Reverse | TGTTTCCGAGGAAAAACCAG |
| NK6-3-Forward | CAGATCCCACTGTACCAAGG |
| NK6-3-Reverse | CCAGTCTGGTGTCCCTATTG |
| Vgll2-Forward | TGAGCTTCCTGCATTTTTGA |
| Vgll2-Reverse | GTACCAGGTCTACGGTCCC |
| Leprel1 Forward | AGAACGTGTGAGCTGCTTCC |
| Leprel1 Reverse | TCCAACGCAGAGTTCCCTAC |
| Nfix-Forward | CACTGGGGCGACTTGTAGAG |
| Nfix-Reverse | AGGCTGACAAGGTGTGGC |
| Slc17a7-Forward | CGAAGCAAAGACCCCATAGA |
| Slc17a7-Reverse | GTGCAATGACCAAGCACAAG |
| Htr4, Zfp353, Xist, Hprt1, Gapdh | Source SA Biosciences (Frederick, MD, a Qiagen Co) |

**Table S2**: Genes down regulated ≥ 2 fold in the cerebral hemisphere of both male and female pups from mothers having FA supplementation at 4 mg/kg during gestation in comparison to mothers at 0.4 mg/kg diet.

| **Accession** | **Symbol** | **Fold Change Male** | | **Fold Change Female** |
| --- | --- | --- | --- | --- |
| gb|CA491940 | 0610008F07Rik | -2.289 | -2.288 | |
| gb|AK012274 | 2700022O18Rik | -7.438 | -2.283 | |
| gb|AK132490 | 4732463B04Rik | -7.563 | -2.208 | |
| gb|AK015045 | 4930402D18Rik | -3.866 | -2.922 | |
| gb|AK015599 | 4930480G23Rik | -2.916 | -2.435 | |
| gb|AK015708 | 4930505N22Rik | -2.107 | -2.467 | |
| ref|NM_001077684 | 4930578N16Rik | -6.333 | -2.045 | |
| gb|AK016745 | 4933408N05Rik | -3.061 | -2.43 | |
| gb|AK029486 | A730032A03Rik | -3.408 | -2.218 | |
| gb|BG069650 | AU020147 | -2.19 | -5.529 | |
| Unknown | A_55_P2009037 | -2.495 | -2.702 | |
| ref|NM_053156 | Allc | -3.603 | -2.12 | |
| ref|NM_025778 | Bcl2l14 | -2.263 | -2.155 | |
| gb|BG079244 | D15Ertd50e | -2.224 | -2.252 | |
| ens|ENSMUST00000038776 | ENSMUST00000038776 | -2.447 | -3.487 | |
| ens|ENSMUST00000041220 | ENSMUST00000041220 | -4.925 | -2.315 | |
| ens|ENSMUST00000101255 | ENSMUST00000101255 | -2.304 | -3.002 | |
| ens|ENSMUST00000103492 | ENSMUST00000103492 | -3.224 | -3.015 | |
| ens|ENSMUST00000106832 | ENSMUST00000106832 | -2.102 | -2.269 | |
| ens|ENSMUST00000108880 | ENSMUST00000108880 | -2.427 | -2.359 | |
| ref|NM_177350 | Gldn | -8.688 | -2.334 | |
| ref|XM_001475143 | Gm13017 | -2.004 | -2.402 | |
| ref|XM_001472979 | Gm2582 | -2.632 | -5.042 | |
| ref|XM_001474354 | Gm2678 | -2.815 | -2.525 | |
| ref|XM_001474560 | Gm2739 | -2.572 | -2.639 | |
| ref|XM_001474628 | Gm2753 | -2.383 | -2.055 | |
| ref|XM_136686 | Gm4958 | -3.042 | -2.249 | |
| ref|NM_001013750 | Gm597 | -2.148 | -2.413 | |
| ref|XM_897430 | Gm6282 | -5.618 | -2.768 | |
| ref|NM_010951 | Gpr143 | -2.766 | -2.743 | |
| ref|NM_009330 | Hnf1b | -2.228 | -2.442 | |
| ref|NM_008357 | Il15 | -3.057 | -2.001 | |
| ref|NM_010583 | Itk | -8.877 | -2.029 | |
| ref|XM_001472790 | LOC100039380 | -2.082 | -2.407 | |
| ref|XM_001479063 | LOC100047654 | -5.606 | -5.245 | |
| ref|XM_001480007 | LOC100048027 | -4.332 | -6.378 | |
| ref|XM_911832 | LOC636441 | -3.665 | -3.05 | |
| ref|NM_010775 | Mbl1 | -2.493 | -2.648 | |
| ref|NM_008571 | Mcpt2 | -2.02 | -3.735 | |
| ref|NM_207574 | Olfr1383 | -2.5 | -2.516 | |
| ref|NM_146471 | Olfr1393 | -3.456 | -2.689 | |
| ref|NM_146706 | Olfr401 | -2.055 | -2.304 | |
| ens|ENSMUST00000045546 | Olfr607 | -2.107 | -2.228 | |
| ref|NM_053127 | Pcdhb2 | -2.423 | -2.045 | |
| ref|NM_177568 | Plcb2 | -2.433 | -2.029 | |
| ref|XM_905487 | Rpl19-ps4 | -2.138 | -3.169 | |
| ref|NM_027301 | Sdr9c7 | -2.615 | -2.878 | |
| ref|NM_173070 | Sprr4 | -2.597 | -2.099 | |
| ref|NM_019992 | Stap1 | -9.613 | -2.149 | |
| ref|NM_018803 | Syt10 | -3.74 | -2.116 | |
| ref|NM_203489 | Vmn1r183 | -4.061 | -2.127 | |
| ref|NM_001103368 | Vmn2r80 | -2.819 | -2.141 | |
| ref|NM_001012725 | Wfdc6b | -7.787 | -2.108 | |
| ref|NM_153096 | Zfp353 | -3.776 | -2.39 | |
| Unknown | chr10:126151060-126156450_R | -2.805 | -2.743 | |
| Unknown | chr10:126151060-126156450_R | -2.805 | -2.743 | |
| Unknown | chr10:84005221-84022896_F | -2.186 | -2.717 | |
| Unknown | chr15:66682293-66722977_F | -2.736 | -2.117 | |
| Unknown | chr18:3003075-3016150_F | -2.586 | -2.056 | |
| Unknown | chr1:167218236-167218418_F | -2.407 | -2.656 | |
| Unknown | chr1:77819988-77820546_F | -2.127 | -2.061 | |
| Unknown | chr8:124364048-124365705_F | -2.075 | -2.189 | |
| Unknown | chr8:49911596-49912196_R | -2.173 | -2.198 | |
| Unknown | chr8:59994074-60002058_R | -2.183 | -2.537 | |
| Unknown | chr9:27153416-27153677_F | -2.65 | -2.626 | |
| Unknown | chr9:7214753-7217212_F | -2.28 | -3.169 | |
| Unknown | chrX:146832315-146875680_R | -3.714 | -2.236 | |
| Unknown | chrX:6982891-7008528_R | -2.145 | -2.116 | |

**Table S3**: Genes up regulated ≥ 2 fold in the cerebral hemisphere of both male and female pups from mothers having FA supplementation at 4 mg/kg during gestation in comparison to mothers at 0.4 mg/kg diet.

| **Accession** | **Symbol** | **Fold Change Male** | **Fold Change Female** |
| --- | --- | --- | --- |
| gb|AK005727 | 1700007J24Rik | 2.398 | 2.252 |
| ref|NM_028014 | 2310067B10Rik | 2.403 | 2.114 |
| ref|NM_029107 | 4930417G10Rik | 2.358 | 2.506 |
| ref|XM_001479264 | 4930439D14Rik | 2.288 | 2.283 |
| ref|NR_033596 | 5730416F02Rik | 2.531 | 2.012 |
| ref|NM_029530 | 6330527O06Rik | 2.092 | 2.252 |
| ref|NR_015488 | A930003A15Rik | 2.136 | 2.087 |
| ref|NM_001012310 | AI132487 | 2.066 | 2.702 |
| gb|BB756663 | AI448005 | 2.439 | 3.311 |
| gb|BU848224 | AW047481 | 2.932 | 2.985 |
| ref|NM_133715 | Arhgap27 | 2.865 | 2.369 |
| ref|NM_001110506 | BC060267 | 2.421 | 4.098 |
| ref|NR_033527 | Ccl25 | 3.412 | 4.237 |
| ref|NM_001136055 | Cd82 | 2.475 | 2.123 |
| ref|NM_027218 | Clec4b1 | 2.624 | 6.493 |
| ref|NM_172687 | Coq3 | 2.915 | 3.558 |
| ref|NM_030143 | Ddit4l | 2.631 | 2.232 |
| ens|ENSMUST00000044682 | ENSMUST00000044682 | 4.545 | 2.32 |
| ens|ENSMUST00000044964 | ENSMUST00000044964 | 2.267 | 2.212 |
| ens|ENSMUST00000084298 | ENSMUST00000084298 | 2.638 | 2.272 |
| ens|ENSMUST00000105022 | ENSMUST00000105022 | 2.358 | 2.84 |
| ens|ENSMUST00000107605 | ENSMUST00000107605 | 3.115 | 8.474 |
| ens|ENSMUST00000108216 | ENSMUST00000108216 | 3.076 | 2.375 |
| ens|ENSMUST00000114775 | ENSMUST00000114775 | 3.448 | 2.192 |
| ens|ENSMUST00000119133 | ENSMUST00000119133 | 4.201 | 2.207 |
| ref|NM_028671 | Fam122c | 4.016 | 3.3 |
| ref|NM_008068 | Gabra6 | 2.145 | 12.82 |
| ref|XM_001475884 | Gm14717 | 2 | 2.061 |
| ref|XM_001475033 | Gm7902 | 2.785 | 3.546 |
| ref|NM_019545 | Hao2 | 4.273 | 2.347 |
| ref|NM_010452 | Hoxa3 | 4.201 | 2.583 |
| ref|NM_010453 | Hoxa5 | 2.032 | 3.134 |
| ref|NM_008268 | Hoxb5 | 5.524 | 2.793 |
| ref|NM_010460 | Hoxb7 | 2.164 | 3.891 |
| ref|NM_013553 | Hoxc4 | 2.232 | 5.494 |
| ref|NM_010661 | Krt12 | 2.702 | 2.427 |
| ref|XM_001472384 | LOC100048880 | 2.309 | 3.105 |
| ref|XM_135380 | LOC235882 | 2.04 | 2.564 |
| ref|NM_001037923 | Lekr1 | 2.427 | 2.222 |
| ref|NM_001170788 | Lrrc36 | 2.178 | 2.785 |
| ref|NM_153789 | Mylip | 2.493 | 2.493 |
| ref|NM_001011753 | Olfr115 | 2.036 | 14.285 |
| ref|NM_146958 | Olfr171 | 2.352 | 2.07 |
| ref|NM_145495 | Rin1 | 2.785 | 2.597 |
| ref|NM_027394 | Ube2cbp | 2.092 | 2.906 |
| ref|NM_134198 | Vmn1r234 | 2.074 | 2.463 |
| ref|NM_011737 | Ysk4 | 2.288 | 5.05 |
| ref|NM_175358 | Zdhhc15 | 2.631 | 2.352 |
| Unknown | chr15:60656586-60685333_R | 2.012 | 62.5 |
| Unknown | chr17:10517466-10522172_F | 2.421 | 2.816 |
| Unknown | chr18:6144775-6202232_R | 3.012 | 2.057 |
| Unknown | chr19:61177675-61219875_F | 2.164 | 6.578 |
| Unknown | chr3:9401052-9415202_F | 2.049 | 5.319 |
| Unknown | chr5:77188626-77219161_F | 2.008 | 2.07 |
| Unknown | chr6:31233512-31241062_F | 2.77 | 3.571 |
| Unknown | chr7:68849401-68958266_R | 2.288 | 2.358 |

**Table S4**: Genes down regulated ≥ 2 fold in the cerebral hemisphere of male pups from mothers having FA supplementation during gestation at 4 mg/kg in comparison to mothers at 0.4 mg/kg diet.

| **UniqueID** | **Accession** | **Symbol** | **Fold Change** |
| --- | --- | --- | --- |
| A_55_P2247021 | ref|NM_029317|ens|ENSMUST00000085422|ens|ENSMUST00000107897|gb|AK006268 | 1700023D19Rik | -18.23 |
| A_30_P01032406 | Unknown | chr14:25924039-25924372_F | -17.64 |
| A_30_P01023738 | Unknown | chr11:97457180-97487074_F | -15.79 |
| A_55_P1977279 | ref|NR_028438|gb|AK078991|tc|TC1656069|riken|9230102K24 | 9230102K24Rik | -14.61 |
| A_51_P490337 | ref|NM_030028|ens|ENSMUST00000013235|gb|AK019807|tc|TC1590202 | Tmem190 | -14.24 |
| A_52_P185162 | ens|ENSMUST00000110854|gb|BC037066|gb|AK018566|gb|BC022639 | ENSMUST00000110854 | -13.01 |
| A_55_P2129442 | ens|ENSMUST00000080872|gb|AK086953|tc|TC1613979|riken|E030015K02 | ENSMUST00000080872 | -12.86 |
| A_55_P2144508 | ref|XM_001474135|ref|XR_035752 | LOC100040235 | -12.71 |
| A_51_P336325 | ref|NM_008768|ens|ENSMUST00000030044|gb|M12567|gb|CT010399 | Orm1 | -12.44 |
| A_51_P140803 | ref|NM_020495|ens|ENSMUST00000042812|ens|ENSMUST00000087577|gb|AB031959 | Slco1b2 | -12.26 |
| A_30_P01024008 | Unknown | chr1:167802183-167805411_F | -12.12 |
| A_30_P01028629 | Unknown | chr15:83181291-83197750_F | -12.06 |
| A_55_P2365897 | gb|AK169782|riken|E430022E24|nap|NAP125336-1 | AU018505 | -10.94 |
| A_66_P130855 | ens|ENSMUST00000103671|nap|NAP018998-001 | ENSMUST00000103671 | -10.94 |
| A_30_P01029985 | Unknown | chr18:75131739-75144870_R | -10.88 |
| A_30_P01023749 | Unknown | chr17:32044823-32045974_R | -10.67 |
| A_55_P1993899 | ens|ENSMUST00000023160|ens|ENSMUST00000100211|gb|AK133365|gb|AK019778 | 4930562C15Rik | -10.48 |
| A_30_P01019466 | Unknown | chr18:61783548-61783935_F | -10.32 |
| A_30_P01025257 | Unknown | chr2:156654675-156665430_R | -10.23 |
| A_52_P388780 | ref|NM_001164166|gb|AK160151|tc|TC1669462|riken|1700054H16 | Pom121l12 | -10.18 |
| A_55_P1973688 | ref|XM_001474837|ref|XM_001478236 | Gm15482 | -9.91 |
| A_55_P2027416 | ref|XM_001476144|tc|TC1605312 | LOC100046065 | -9.90 |
| A_55_P1980308 | ref|NM_019992|ens|ENSMUST00000031171|ens|ENSMUST00000113389|gb|BC145550 | Stap1 | -9.61 |
| A_52_P341058 | ref|NM_177335|ens|ENSMUST00000120642|ens|ENSMUST00000132744|ens|ENSMUST00000140802 | D930020B18Rik | -9.59 |
| A_55_P2421222 | gb|AK016739|tc|TC1595998|riken|4933408J17|nap|NAP073520-1 | 4933408J17Rik | -9.58 |
| A_30_P01026703 | Unknown | chr1:161064402-161096518_F | -9.53 |
| A_30_P01027648 | Unknown | chr3:83089315-83108396_F | -9.52 |
| A_55_P2143406 | ens|ENSMUST00000068033 | ENSMUST00000068033 | -9.50 |
| A_30_P01022620 | Unknown | chr3:27837825-27880925_R | -9.33 |
| A_55_P2322660 | gb|BC051537|gb|AK136114|riken|7420458H03 | BC051537 | -9.32 |
| A_66_P117201 | ref|NM_010202|ens|ENSMUST00000060336|gb|AK082814|tc|TC1588591 | Fgf4 | -9.28 |
| A_55_P2264762 | gb|AK163097|tc|TC1724359|tc|TC1779535|riken|A530069H21 | 4933407K13Rik | -9.24 |
| A_55_P2179151 | ens|ENSMUST00000100658|gb|AK157412|riken|F830215A15 | ENSMUST00000100658 | -9.24 |
| A_55_P2005999 | ref|XM_896050|gb|CF198073|tc|TC1763344|nap|NAP100825-001 | Gm6185 | -9.20 |
| A_55_P2032478 | ens|ENSMUST00000113788|gb|BC089588|gb|AK164150|tc|TC1643804 | ENSMUST00000113788 | -9.20 |
| A_51_P173484 | ens|ENSMUST00000080298|gb|AK016353|tc|TC1594119|riken|4930585L22 | ENSMUST00000080298 | -9.19 |
| A_30_P01019073 | Unknown | chr2:173420987-173480087_F | -9.12 |
| A_51_P433083 | ens|ENSMUST00000034550|gb|AK015742|tc|TC1595867|riken|4930510E17 | ENSMUST00000034550 | -9.11 |
| A_52_P676255 | ref|NM_001163527|ref|NM_001163528|ens|ENSMUST00000154021|ens|ENSMUST00000110386 | Itpripl1 | -9.11 |
| A_55_P2137726 | ref|XM_001479883|ref|XM_001480002|gb|AK015516|riken|4930466O16 | Gm10620 | -8.93 |
| A_55_P1953788 | ref|NM_010583|ens|ENSMUST00000020664|ens|ENSMUST00000109237|gb|AK153613 | Itk | -8.88 |
| A_55_P2114959 | ref|NM_001111094|ens|ENSMUST00000114181|gb|BC052925|tc|TC1589587 | Btnl1 | -8.86 |
| A_55_P1976859 | ens|ENSMUST00000107551|ens|ENSMUST00000147971|tc|TC1768226|tc|TC1623448 | ENSMUST00000107551 | -8.85 |
| A_52_P346987 | ref|NM_008272|ens|ENSMUST00000001706|gb|AK012173|gb|X55318 | Hoxc9 | -8.82 |
| A_30_P01018418 | Unknown | chr4:46583780-46614788_R | -8.72 |
| A_51_P284503 | ref|XM_897249|ref|XM_925070|gb|AK004025|tc|TC1694027 | Krtap22-2 | -8.71 |
| A_55_P1982499 | ref|NM_177350|ens|ENSMUST00000056740|gb|AK031523|gb|AF548022 | Gldn | -8.69 |
| A_30_P01031472 | Unknown | chr19:29582018-29596693_F | -8.68 |
| A_55_P2028680 | ref|NM_029367|ens|ENSMUST00000103223|ens|ENSMUST00000065468|ens|ENSMUST00000103222 | Spaca3 | -8.68 |
| A_66_P126877 | ref|NM_134214|ens|ENSMUST00000074252|gb|BC145835|tc|NP508424 | Vmn1r214 | -8.67 |
| A_52_P321244 | ens|ENSMUST00000063230|gb|AK076988|tc|TC1593019|riken|4930595D20 | ENSMUST00000063230 | -8.66 |
| A_55_P2339399 | gb|C78361 | D14Ertd170e | -8.63 |
| A_55_P2229098 | gb|AK090054|tc|TC1610310|riken|G430080K10|nap|NAP072423-1 | LOC629206 | -8.49 |
| A_30_P01019793 | Unknown | chr14:99753319-99753569_R | -8.49 |
| A_51_P333274 | ref|NM_013542|ens|ENSMUST00000015581|gb|X04072|gb|M12302 | Gzmb | -8.48 |
| A_55_P1963727 | ref|NM_007947|ref|NM_001042630|ens|ENSMUST00000100312|ens|ENSMUST00000100313 | Lcn5 | -8.48 |
| A_52_P302371 | ref|NM_153512|ens|ENSMUST00000051482|gb|AF454552|gb|AF454551 | Kcng3 | -8.45 |
| A_51_P504735 | ref|NM_146791|ens|ENSMUST00000099770|gb|BC150718|nap|NAP110779-1 | Olfr1248 | -8.42 |
| A_55_P2166232 | ref|XM_911001|ref|XM_976347 | Kdm4dl | -8.40 |
| A_55_P1969506 | ref|NM_009198|ref|NM_001170638|ens|ENSMUST00000110413|ens|ENSMUST00000006785 | Slc17a1 | -8.39 |
| A_55_P1966634 | ref|NM_001102578|ref|NM_001105187|nap|NAP092187-001|nap|NAP113640-1 | Vmn2r75 | -8.34 |
| A_55_P2044932 | ref|NM_030720|ens|ENSMUST00000079824|gb|BC023249|gb|AF272948 | Gpr84 | -8.32 |
| A_55_P2185618 | ref|NM_175470|ens|ENSMUST00000062028|ref|XM_001478540|ref|XM_001480906 | Gpr61 | -8.17 |
| A_55_P2068184 | ref|XM_001473044|ref|XM_001473253|tc|TC1615311 | XM_001473044 | -8.16 |
| A_55_P2065601 | ref|NM_177704|ens|ENSMUST00000086165|ens|ENSMUST00000067529|gb|BC150158 | Sytl5 | -8.09 |
| A_55_P2075364 | ref|XM_001472141|ref|XM_001472908|ref|XM_001473097|ref|XM_001475486 | LOC100039041 | -8.07 |
| A_30_P01032062 | Unknown | chr3:96206303-96206871_F | -8.06 |
| A_30_P01028178 | Unknown | chr2:155667801-155668175_R | -8.04 |
| A_55_P2026720 | ref|XM_001473850|ref|XM_001478271|gb|AK016834|tc|TC1600323 | 4933416M06Rik | -8.04 |
| A_30_P01022436 | Unknown | chr9:88577769-88614651_R | -8.03 |
| A_55_P2025630 | ref|XM_918521 | LOC641177 | -7.98 |
| A_52_P374983 | ens|ENSMUST00000098492|gb|AK080381|tc|TC1595732|riken|A630085K21 | ENSMUST00000098492 | -7.97 |
| A_30_P01032944 | Unknown | chr1:195332659-195335997_R | -7.94 |
| A_51_P438990 | ref|NM_146873|ens|ENSMUST00000074987|gb|BC127963|tc|TC1637240 | Olfr909 | -7.92 |
| A_66_P132491 | ens|ENSMUST00000100683|gb|AK148391|tc|TC1645642|riken|G370126I09 | ENSMUST00000100683 | -7.89 |
| A_55_P1979335 | ens|ENSMUST00000029268|ref|XM_001475130|ref|XM_001476270|gb|AK007933 | 1810062G17Rik | -7.80 |
| A_55_P1966155 | ref|NM_001012725|ens|ENSMUST00000094346|gb|BC141216|gb|AY541526 | Wfdc6b | -7.79 |
| A_66_P103316 | ref|XR_035266|ref|XR_035359|gb|AK039704|riken|A330091H04 | Gm6521 | -7.72 |
| A_55_P2037568 | ref|XR_032672 | LOC100046502 | -7.72 |
| A_52_P273865 | ens|ENSMUST00000088858|gb|AK016370|tc|TC1596250|riken|4930588P12 | ENSMUST00000088858 | -7.65 |
| A_55_P2414707 | gb|AK132490|riken|4732463B04 | 4732463B04Rik | -7.56 |
| A_55_P2072671 | ref|NM_146836|ens|ENSMUST00000111571|ens|ENSMUST00000090711|tc|NP646759 | Olfr1132 | -7.54 |
| A_52_P428801 | ref|XR_002346|ref|XR_005168|gb|AK076825|gb|BC120772 | Gm5108 | -7.50 |
| A_55_P2002460 | ref|XM_001473424 | Gm2399 | -7.47 |
| A_55_P2135730 | ref|NM_175436|ens|ENSMUST00000092652|ens|ENSMUST00000055604|gb|BC096589 | Zfp526 | -7.45 |
| A_55_P2233462 | gb|AK012274|tc|TC1604045|riken|2700022O18|nap|NAP091368-1 | 2700022O18Rik | -7.44 |
| A_51_P431543 | ens|ENSMUST00000044533|ref|XM_487606|ref|XM_906114|gb|AK006434 | 1700027L20Rik | -7.40 |
| A_65_P13946 | ref|XM_001472183|ref|XM_910788|tc|TC1645007 | Fbxw23 | -7.40 |
| A_55_P1972772 | ref|NM_010352|ref|NM_001080553|ref|NM_001080552|ens|ENSMUST00000111911 | Gsg1 | -7.39 |
| A_55_P2096174 | ref|XM_974412|ref|XM_886735|nap|NAP062174-1 | 5830473C10Rik | -7.36 |
| A_66_P122860 | ref|NM_001161433|ens|ENSMUST00000113832|gb|BC106845|gb|AK049134 | Eda2r | -7.31 |
| A_30_P01022892 | Unknown | chrX:120819096-120820227_R | -7.30 |
| A_55_P2172545 | ref|NM_175938|gb|BB489469 | Btn2a2 | -7.28 |
| A_30_P01024079 | Unknown | chr5:92556322-92558910_F | -7.19 |
| A_30_P01027114 | Unknown | chrX:120353972-120361170_R | -7.16 |
| A_55_P2067787 | ref|NM_054064|ens|ENSMUST00000075934|gb|BC114207|nap|NAP032032-1 | Psg29 | -7.15 |
| A_51_P244950 | ref|NM_001164466|ens|ENSMUST00000110306|gb|AK004822|gb|AF249296 | Dpys | -7.15 |
| A_55_P1967099 | ref|XM_001478921 | LOC100047816 | -7.10 |
| A_66_P109692 | ens|ENSMUST00000103332|tc|TC1706422 | ENSMUST00000103332 | -7.09 |
| A_55_P2061049 | ref|XM_001476580 | Gm3406 | -7.07 |
| A_30_P01023002 | Unknown | chrX:120812236-121038407_R | -7.07 |
| A_55_P2169779 | ens|ENSMUST00000105713|ens|ENSMUST00000149129 | ENSMUST00000105713 | -7.03 |
| A_52_P629748 | ens|ENSMUST00000066154|gb|AK028718|gb|AK144387|gb|AV240823 | ENSMUST00000066154 | -6.93 |
| A_55_P2293013 | ref|NM_133960|ref|NM_001190330|ens|ENSMUST00000034346|gb|AK165784 | Ces6 | -6.88 |
| A_66_P123812 | ref|NM_146357|ens|ENSMUST00000078554|gb|BC127969|tc|TC1600884 | Olfr168 | -6.85 |
| A_55_P2081383 | ref|NM_001039595|ref|NM_001113735|ref|NM_001113736|ens|ENSMUST00000054977 | Gm13043 | -6.81 |
| A_65_P07768 | ref|NR_033458|ens|ENSMUST00000097404|gb|AK135822|riken|7420422E11 | Gm10512 | -6.58 |
| A_55_P1981030 | ens|ENSMUST00000114743|tc|TC1655950 | ENSMUST00000114743 | -6.57 |
| A_55_P2112315 | ref|XR_035433|nap|NAP017842-001 | 4930432E11Rik | -6.56 |
| A_30_P01025562 | Unknown | chr14:76912477-76931702_F | -6.55 |
| A_55_P1955602 | ref|XM_990840|tc|TC1682426 | 2010001A14Rik | -6.54 |
| A_55_P2401004 | gb|AK138270|riken|A230049L08 | B230110C06Rik | -6.51 |
| A_55_P2198946 | ref|NR_033472|gb|AK143931|tc|TC1681492|riken|F530001O12 | Gm10768 | -6.49 |
| A_55_P2082703 | gb|AK155468|tc|TC1587715|riken|F630230J21 | Usp-ps | -6.47 |
| A_55_P2004184 | tc|TC1629580 | TC1629580 | -6.46 |
| A_55_P2163413 | ref|XM_001476819 | Gm3465 | -6.44 |
| A_55_P1989599 | ref|NM_029048|ens|ENSMUST00000085733|ens|ENSMUST00000110743|gb|AK015441 | Spdye4 | -6.42 |
| A_55_P2021014 | ref|NM_207278|ens|ENSMUST00000062623|gb|AK081667|tc|TC1593446 | Tigd4 | -6.42 |
| A_30_P01025443 | Unknown | chr17:26613904-26614277_F | -6.37 |
| A_52_P174721 | ref|NM_176950|ens|ENSMUST00000061584|ens|ENSMUST00000109836|gb|BC141487 | Defb20 | -6.34 |
| A_52_P501875 | ref|NM_001077684|ens|ENSMUST00000094942|gb|AK016309|gb|AK015894 | 4930578N16Rik | -6.33 |
| A_51_P368123 | ref|NM_008699|ens|ENSMUST00000057178|gb|AF202036|gb|AK044871 | Nkx2-3 | -6.27 |
| A_55_P2025552 | ref|XM_137324|ref|XM_905568|nap|NAP023780-001 | Gm4801 | -6.26 |
| A_55_P2046493 | ens|ENSMUST00000099803 | ENSMUST00000099803 | -6.23 |
| A_66_P129347 | ref|NM_001033793|ens|ENSMUST00000098973|gb|AK163257|gb|AK139937 | Ubtfl1 | -6.10 |
| A_55_P2080658 | ens|ENSMUST00000105252|gb|AK157048|gb|BC131649|riken|F830115E19 | ENSMUST00000105252 | -6.09 |
| A_55_P2281733 | gb|BG063235 | AA408396 | -6.05 |
| A_66_P140433 | ens|ENSMUST00000014133|ref|XR_035135|ref|XR_035132|gb|AK084946 | ENSMUST00000014133 | -6.05 |
| A_55_P2048467 | ens|ENSMUST00000081144|ref|XM_136255|ref|XM_915762|nap|NAP017872-001 | Gm4845 | -6.01 |
| A_55_P2083386 | ref|NR_002869|nap|NAP060183-1 | Gm5477 | -6.01 |
| A_30_P01024554 | Unknown | chr2:172343878-172344212_R | -5.99 |
| A_55_P2122614 | ref|NM_001104566|nap|NAP101499-1 | Vmn2r104 | -5.99 |
| A_30_P01032571 | Unknown | chr18:23910833-23933185_F | -5.97 |
| A_51_P351896 | ref|NM_133187|ens|ENSMUST00000029567|ens|ENSMUST00000118853|gb|AK143522 | Fam198b | -5.96 |
| A_30_P01031348 | Unknown | chr3:51804655-51805883_R | -5.94 |
| A_55_P2080183 | ens|ENSMUST00000098988|tc|TC1741071 | ENSMUST00000098988 | -5.91 |
| A_52_P562624 | ref|NM_138674|ens|ENSMUST00000038336|gb|AY219182|tc|TC1645353 | Pkhd1l1 | -5.89 |
| A_55_P2147315 | ref|NM_146683|ens|ENSMUST00000059033|gb|BC145847|tc|NP830820 | Olfr1441 | -5.88 |
| A_55_P1992218 | ref|XM_001476624 | LOC100046688 | -5.85 |
| A_55_P1983338 | ref|NM_146661|ens|ENSMUST00000053050|tc|NP830907|nap|NAP030033-1 | Olfr1112 | -5.84 |
| A_30_P01022731 | Unknown | chr8:60132300-60146830_R | -5.82 |
| A_51_P472932 | ref|NM_001001488|ens|ENSMUST00000025482|gb|AK149470|gb|AK157316 | Atp8b1 | -5.79 |
| A_66_P126005 | ref|XM_001475704|gb|AK006948|tc|TC1637889|riken|1700074I03 | 1700074I03Rik | -5.69 |
| A_55_P2003388 | ref|NM_181317|ens|ENSMUST00000049742|ens|ENSMUST00000072868|gb|AK039387 | Kcns2 | -5.67 |
| A_66_P103052 | ref|XM_897430|ref|XM_921604|nap|NAP058187-1 | Gm6282 | -5.62 |
| A_55_P2097284 | ref|XM_001479063 | LOC100047654 | -5.61 |
| A_55_P1981231 | ref|XM_001473439 | LOC100044978 | -5.59 |
| A_52_P84347 | ref|XM_001473883|ref|XM_001477957|gb|AK016464|tc|TC1600269 | 4931419H13Rik | -5.57 |
| A_55_P2029116 | ens|ENSMUST00000118194|gb|AK165461|riken|F530008I07 | ENSMUST00000118194 | -5.54 |
| A_30_P01021439 | Unknown | chr5:53931541-53945241_R | -5.50 |
| A_55_P2063486 | ref|XM_001473418|ref|XM_001473653|ref|XM_001474607|ref|XM_001473396 | LOC100040022 | -5.46 |
| A_51_P306933 | ref|NM_019404|ens|ENSMUST00000033765|gb|AJ310753|tc|TC1590212 | Avpr2 | -5.44 |
| A_30_P01019775 | Unknown | chr5:106351376-106352151_F | -5.44 |
| A_55_P2317730 | gb|AK010100|tc|TC1599812|riken|2310067P03|nap|NAP091270-1 | 2310067P03Rik | -5.43 |
| A_55_P2301713 | gb|AK034922|tc|TC1615133|riken|9430062P05|nap|NAP078508-1 | 9430062P05Rik | -5.42 |
| A_55_P2053564 | ref|NM_001034898|ens|ENSMUST00000087923|gb|AK143446|tc|TC1592161 | Ms4a15 | -5.41 |
| A_30_P01032961 | Unknown | chrX:122184810-122500048_F | -5.33 |
| A_52_P567228 | ref|NM_001160265|ens|ENSMUST00000031521|nap|NAP026994-1|nap|NAP026995-1 | Cyp2w1 | -5.32 |
| A_55_P1962049 | ref|NM_001037166|ens|ENSMUST00000092231|gb|BC100581|tc|TC1593418 | Gm4925 | -5.26 |
| A_55_P2040951 | ref|NM_009608|ens|ENSMUST00000116448|ens|ENSMUST00000090269|ref|XR_034583 | Actc1 | -5.25 |
| A_55_P1970686 | ref|XR_031715 | LOC100045460 | -5.23 |
| A_30_P01027505 | Unknown | chr13:62152594-62153041_R | -5.18 |
| A_55_P2019896 | ref|NR_030675|ens|ENSMUST00000100428|gb|AK035732|gb|AK037689 | D730005E14Rik | -5.18 |
| A_30_P01025773 | Unknown | chr12:4133875-4243375_R | -5.16 |
| A_55_P2000964 | ref|NM_029901|ens|ENSMUST00000021628|ens|ENSMUST00000074985|gb|AK020439 | Akr1c21 | -5.16 |
| A_55_P1978920 | ref|XM_001476569 | LOC100041553 | -5.12 |
| A_55_P2340613 | gb|AK020962|tc|TC1611215|riken|B230110G15|nap|NAP076540-1 | B230110G15Rik | -5.10 |
| A_30_P01027021 | Unknown | chr5:104936417-104984892_F | -5.10 |
| A_51_P297069 | ref|NM_021883|ens|ENSMUST00000030012|ens|ENSMUST00000107773|gb|S76831 | Tmod1 | -5.10 |
| A_55_P2048867 | ref|XM_001475122|ref|XM_001476820 | Gm2922 | -5.01 |
| A_30_P01033258 | Unknown | chr14:80080060-80080597_R | -4.98 |
| A_30_P01027995 | Unknown | chr12:33606225-33638225_R | -4.98 |
| A_55_P1977528 | ref|NM_001128103|tc|TC1653751|nap|NAP030012-1 | Ano3 | -4.97 |
| A_55_P1958906 | ref|NM_007732|ens|ENSMUST00000026045|ens|ENSMUST00000086923|gb|L08407 | Col17a1 | -4.97 |
| A_55_P2145164 | ref|NR_015385|gb|AY589792 | Six3os1 | -4.95 |
| A_30_P01022565 | Unknown | chr4:3752496-3752901_R | -4.93 |
| A_55_P2101577 | ens|ENSMUST00000041220|gb|AK029603|riken|4930412E20|nap|NAP073993-1 | ENSMUST00000041220 | -4.93 |
| A_52_P381484 | ref|NM_133903|ens|ENSMUST00000046186|gb|AK042840|gb|AK131131 | Spon2 | -4.91 |
| A_30_P01018147 | Unknown | chr16:91198576-91199149_F | -4.89 |
| A_55_P2087719 | ref|XM_979107 | Gm7762 | -4.87 |
| A_55_P2067632 | ref|NM_001163733|ref|NM_153677|ref|NM_023649|ens|ENSMUST00000154292 | Ush1c | -4.84 |
| A_51_P160713 | ref|NM_009654|ens|ENSMUST00000031314|gb|AJ457860|gb|AK050644 | Alb | -4.82 |
| A_52_P223571 | ref|NM_175403|ens|ENSMUST00000053271|ens|ENSMUST00000112121|gb|AK075940 | Mlec | -4.80 |
| A_30_P01027027 | Unknown | chr2:77146893-77155643_F | -4.79 |
| A_52_P554594 | ref|NM_198960|ens|ENSMUST00000048194|gb|AY325902|tc|TC1584194 | Tcfap2e | -4.78 |
| A_66_P107483 | ref|NM_001033405|ens|ENSMUST00000095248|ref|XM_001479405|ref|XM_001479411 | Treml2 | -4.78 |
| A_55_P2142211 | ref|XM_001475551 | Gm15246 | -4.77 |
| A_51_P249335 | ref|NM_145565|ens|ENSMUST00000066540|gb|BC021605|gb|AK149438 | Sds | -4.76 |
| A_55_P2084348 | ref|NM_001042503|ens|ENSMUST00000111816|ref|XM_001476361|gb|DQ005956 | Trim71 | -4.74 |
| A_55_P1981150 | ens|ENSMUST00000113803 | ENSMUST00000113803 | -4.74 |
| A_55_P2077368 | ref|NM_176913|ens|ENSMUST00000081998|gb|AF488552|gb|BC156797 | Dpep2 | -4.73 |
| A_55_P2089665 | ref|XM_001472555|gb|AK144919|riken|G830003D14 | LOC669153 | -4.73 |
| A_66_P131662 | ref|NM_146975|gb|BC104266|gb|BC104267|gb|BC126917 | Olfr1273-ps | -4.72 |
| A_55_P1985067 | ens|ENSMUST00000114274 | ENSMUST00000114274 | -4.71 |
| A_55_P2221186 | gb|AK161464|riken|4932418F08 | A930030B08Rik | -4.68 |
| A_55_P2086585 | ref|XM_001474321 | LOC100045455 | -4.66 |
| A_30_P01022022 | Unknown | chr1:59775022-59780348_R | -4.66 |
| A_51_P102438 | ref|NM_001029867|ref|NM_053215|ens|ENSMUST00000094649|ens|ENSMUST00000075858 | Ugt2b36 | -4.61 |
| A_55_P2340443 | gb|AK028725|tc|TC1612111|riken|4732446E07 | BC042782 | -4.59 |
| A_55_P2379360 | ref|NR_027507|gb|AJ001379|gb|AJ001378|tc|NP1217907 | Tspy-ps | -4.59 |
| A_30_P01017979 | Unknown | chr9:51905799-51906277_F | -4.57 |
| A_51_P122660 | ref|NM_153404|gb|AK035498|gb|AK048150|gb|BC037489 | Liph | -4.56 |
| A_30_P01032531 | Unknown | chr18:77968421-77972209_R | -4.55 |
| A_51_P302738 | ref|NM_009477|ens|ENSMUST00000020677|gb|D44464|tc|TC1581211 | Upp1 | -4.54 |
| A_55_P2012246 | ref|XM_001476761|ref|XM_001479344 | Gm4174 | -4.52 |
| A_55_P2094632 | ref|XM_001478150|ref|XM_001479350 | Gm3356 | -4.50 |
| A_55_P2178723 | ens|ENSMUST00000046398|gb|AB095736|tc|NP798438 | ENSMUST00000046398 | -4.49 |
| A_30_P01029469 | Unknown | chrX:91668178-91668700_R | -4.49 |
| A_55_P1976849 | ref|NR_015593|gb|AK051169|gb|AK051771|gb|AK051495 | D130009I18Rik | -4.49 |
| A_55_P2064989 | ref|NM_001101472|ens|ENSMUST00000109957 | Serpina3j | -4.46 |
| A_55_P1963495 | ens|ENSMUST00000023471|ref|XR_035261|ref|XR_035436|gb|AK016649 | ENSMUST00000023471 | -4.45 |
| A_55_P1976310 | ref|NM_020496|gb|AF260557|tc|TC1591721 | Tbx20 | -4.42 |
| A_30_P01024419 | Unknown | chr13:62771112-62786278_R | -4.42 |
| A_55_P2153116 | gb|AF138745|tc|TC1643100 | Tsix | -4.42 |
| A_55_P2039719 | ref|XM_001006365 | LOC677629 | -4.42 |
| A_52_P434055 | ens|ENSMUST00000115672|gb|BC011338|tc|TC1589149 | ENSMUST00000115672 | -4.42 |
| A_30_P01031015 | Unknown | chr13:29106280-29120376_F | -4.41 |
| A_55_P2066173 | ens|ENSMUST00000103421|ens|ENSMUST00000103420|ens|ENSMUST00000103419|ens|ENSMUST00000103416 | ENSMUST00000103421 | -4.39 |
| A_51_P386802 | ens|ENSMUST00000035860|gb|AK006064|tc|TC1589204|riken|1700017G19 | ENSMUST00000035860 | -4.39 |
| A_55_P2293434 | gb|AK134048|riken|5830419E12 | 5830419E12Rik | -4.39 |
| A_52_P57205 | ref|NM_010572|ens|ENSMUST00000067841|gb|AK045172|gb|BC060235 | Irs4 | -4.38 |
| A_55_P2306453 | gb|AK018247|tc|TC1600769|riken|6330571C24 | 6330571C24Rik | -4.34 |
| A_55_P2174736 | ref|XM_001480007 | LOC100048027 | -4.33 |
| A_55_P2419116 | gb|CA480049 | Snhg9 | -4.33 |
| A_52_P554536 | ref|NM_175649|ens|ENSMUST00000055723|gb|BC138820|gb|BC138819 | Tnfrsf26 | -4.33 |
| A_66_P136582 | ens|ENSMUST00000082150|ref|XM_890347|ref|XM_914658|tc|NP589866 | ENSMUST00000082150 | -4.32 |
| A_55_P2142321 | ref|XM_001478620|gb|AK020250|riken|9030217H17|nap|NAP075831-1 | Gm3957 | -4.31 |
| A_55_P2062329 | ref|XM_993854|ref|XM_990959 | Gm8674 | -4.29 |
| A_55_P1998947 | ref|NM_053169|ens|ENSMUST00000108703|ens|ENSMUST00000055006|gb|AK053139 | Trim16 | -4.28 |
| A_30_P01021601 | Unknown | chr3:41047600-41047808_F | -4.25 |
| A_30_P01025259 | Unknown | chr5:29778934-29795378_F | -4.24 |
| A_55_P2093373 | ref|NM_177330|ens|ENSMUST00000057186|gb|AK049671|tc|TC1669087 | Ghsr | -4.23 |
| A_55_P2357676 | gb|BC064107|gb|BC020449 | BC064107 | -4.22 |
| A_55_P1982926 | ref|NM_146691|ens|ENSMUST00000054687|tc|NP830790|nap|NAP060697-1 | Olfr1467 | -4.22 |
| A_51_P109097 | ref|NM_146835|ens|ENSMUST00000031086|gb|BC130246|tc|NP646758 | Olfr109 | -4.21 |
| A_30_P01031288 | Unknown | chr10:78969674-78981039_F | -4.19 |
| A_55_P2223710 | gb|BC068229|tc|TC1600661 | 2700054A10Rik | -4.17 |
| A_55_P2091022 | ref|XR_030641|ref|XR_031719 | Gm6119 | -4.14 |
| A_52_P404942 | ref|NM_144854|ens|ENSMUST00000026700|gb|BC115625|gb|BC115624 | ORF63 | -4.12 |
| A_30_P01032836 | Unknown | chr6:140557582-140557917_F | -4.12 |
| A_55_P2186282 | ref|NM_001031851|ens|ENSMUST00000022858|ens|ENSMUST00000110542|gb|BC141248 | Agxt2 | -4.11 |
| A_55_P2019799 | ref|NM_146543|ref|NM_001011820|ens|ENSMUST00000077843|ens|ENSMUST00000079135 | Olfr1360 | -4.09 |
| A_51_P400054 | ref|NM_026296|ens|ENSMUST00000031020|gb|AK016690|gb|AK016070 | 4930548H24Rik | -4.09 |
| A_55_P2060249 | ref|NM_173023|ens|ENSMUST00000055156|gb|EF199807|gb|BC132479 | Catsperb | -4.09 |
| A_55_P1988428 | ref|XM_001474311 | LOC100045508 | -4.07 |
| A_30_P01028447 | Unknown | chr17:33425193-33449090_F | -4.07 |
| A_55_P2166613 | ref|XM_001473057 | Gm2290 | -4.07 |
| A_55_P1978845 | ref|NM_203489|ref|NM_206868|ref|NM_001166712|ref|NM_001166713 | Vmn1r183 | -4.06 |
| A_55_P1973578 | ref|NM_007989|ens|ENSMUST00000037824|gb|AF177770|gb|AF069303 | Foxh1 | -4.06 |
| A_30_P01031006 | Unknown | chr5:35492530-35492959_R | -4.05 |
| A_55_P2131048 | ref|XR_031219|ref|XR_031631 | Gm2401 | -4.05 |
| A_55_P2149393 | ref|XM_001476731 | Gm3443 | -4.04 |
| A_55_P2096310 | ref|XM_001473663 | LOC100045096 | -4.04 |
| A_30_P01019130 | Unknown | chr9:118060025-118085050_R | -4.03 |
| A_30_P01025931 | Unknown | chr12:113434415-113453161_F | -4.01 |
| A_30_P01027976 | Unknown | chr18:35046847-35047423_R | -4.01 |
| A_30_P01032778 | Unknown | chr3:35782698-35789748_F | -3.99 |
| A_52_P420216 | ref|NM_030563|ens|ENSMUST00000080846|ens|ENSMUST00000098530|ens|ENSMUST00000034074 | N4bp1 | -3.99 |
| A_30_P01024194 | Unknown | chr16:64758224-64765674_F | -3.99 |
| A_55_P2034385 | ref|NM_001005788|ens|ENSMUST00000084303|ens|ENSMUST00000106280|ens|ENSMUST00000106281 | Zfp69 | -3.98 |
| A_30_P01030133 | Unknown | chr5:53910499-53913968_R | -3.96 |
| A_51_P506733 | ref|NM_001038845|ens|ENSMUST00000031425|gb|AK171132|gb|AK144585 | P2rx7 | -3.96 |
| A_55_P2069468 | ref|NM_027588|ens|ENSMUST00000147323|ens|ENSMUST00000002456|ens|ENSMUST00000118657 | Nt5c1b | -3.95 |
| A_51_P230298 | ref|NM_008232|ens|ENSMUST00000055915|gb|D63663|gb|AK133357 | Hdgfl1 | -3.94 |
| A_30_P01020728 | Unknown | TSIX | -3.94 |
| A_55_P2060639 | ref|NM_201611|ens|ENSMUST00000041398|gb|AY211083|tc|TC1596798 | H2-M10.6 | -3.94 |
| A_55_P2167537 | ref|NM_181549|gb|AK132888|tc|TC1626603|riken|4930485E17 | Clec18a | -3.93 |
| A_55_P2098067 | ens|ENSMUST00000057134|gb|BC125442 | ENSMUST00000057134 | -3.90 |
| A_51_P120356 | gb|BC150838|gb|AK011007|gb|AK011008|tc|TC1593706 | Rwdd3 | -3.90 |
| A_52_P147487 | ref|NM_007731|ens|ENSMUST00000105450|ens|ENSMUST00000048320|ens|ENSMUST00000092484 | Col13a1 | -3.90 |
| A_55_P2357342 | gb|AK005634|tc|TC1591746|riken|1700003F17|nap|NAP012180-001 | 1700003F17Rik | -3.88 |
| A_55_P2337500 | gb|AK085299|tc|TC1597826|riken|D630008C01|nap|NAP024772-001 | 9530052C20Rik | -3.87 |
| A_52_P439887 | ens|ENSMUST00000053799 | ENSMUST00000053799 | -3.87 |
| A_55_P2257306 | gb|AK015045|tc|TC1649531|riken|4930402D18|nap|NAP073968-1 | 4930402D18Rik | -3.87 |
| A_55_P2120016 | ref|NM_213728|ens|ENSMUST00000071104|gb|BC156168|gb|BC157004 | Krt72-ps | -3.86 |
| A_55_P1954468 | ref|NM_001114678|ens|ENSMUST00000104922|ens|ENSMUST00000063726|ens|ENSMUST00000101396 | Gm5072 | -3.86 |
| A_55_P2014144 | ref|NM_146566|ens|ENSMUST00000078861|tc|TC1599952|nap|NAP021176-001 | Olfr830 | -3.85 |
| A_55_P2003333 | ref|XM_001480938|ref|XM_001479967|gb|BB203186 | Gm4641 | -3.82 |
| A_51_P170562 | ref|NM_027904|gb|BC025836|gb|BC081550|gb|BC054470 | Cpn2 | -3.82 |
| A_55_P2171443 | ref|NM_001037718|gb|BC109354|gb|BC116728|tc|NP1482393 | 4932415M13Rik | -3.82 |
| A_55_P2393558 | Unknown | A_55_P2393558 | -3.82 |
| A_55_P2363170 | gb|BY714637 | 4930406D18Rik | -3.82 |
| A_55_P2147831 | ref|NM_174990|ref|NM_175048|ens|ENSMUST00000121957|ens|ENSMUST00000156770 | Gimap4 | -3.81 |
| A_55_P2008830 | ref|XM_001473236|gb|BM939002|tc|TC1674279 | Gm2294 | -3.80 |
| A_66_P135372 | ref|XM_001474200|gb|AK017057|gb|AV254140|tc|TC1720072 | LOC100045417 | -3.79 |
| A_66_P114333 | ref|NM_205823|ens|ENSMUST00000074829|gb|AK137624|gb|AK143385 | Tlr12 | -3.79 |
| A_55_P2067366 | ens|ENSMUST00000087927|gb|BC094552|tc|TC1587927 | ENSMUST00000087927 | -3.79 |
| A_30_P01017908 | Unknown | chr15:102926581-102932542_R | -3.79 |
| A_51_P379750 | ref|NM_021508|ens|ENSMUST00000090469|gb|AK041896|gb|AJ005620 | Myoz1 | -3.78 |
| A_55_P2114528 | ref|NM_016858|ens|ENSMUST00000054387|gb|AB004665|tc|TC1575030 | Rab33b | -3.78 |
| A_55_P2182056 | ref|NM_153096|ref|NM_153102|ens|ENSMUST00000080541|ens|ENSMUST00000094971 | Zfp353 | -3.78 |
| A_51_P136508 | ref|NM_009732|ens|ENSMUST00000046001|gb|AK138179|gb|BC051997 | Avp | -3.77 |
| A_51_P289828 | ref|NM_001162956|gb|AK080572|gb|BC042726|gb|EU234003 | Ccdc138 | -3.77 |
| A_55_P2016717 | ref|XM_001480447|gb|AK086576|tc|TC1619804|riken|D930038D03 | LOC100048441 | -3.77 |
| A_55_P2051239 | ref|NM_181749|ens|ENSMUST00000106584|ens|ENSMUST00000045319|gb|AY957501 | Gpr142 | -3.76 |
| A_66_P134265 | ref|NM_001170572|ref|NM_001033478|ref|NM_001170571|ens|ENSMUST00000149617 | Fam47e | -3.76 |
| A_30_P01032553 | Unknown | chr4:11102287-11118421_F | -3.76 |
| A_30_P01019774 | Unknown | chr4:45079022-45096897_R | -3.75 |
| A_55_P2200009 | gb|C79036 | LOC493582 | -3.75 |
| A_52_P681705 | ref|NM_001142957|gb|AK135959|gb|AK020746|tc|TC1641777 | Gm4455 | -3.75 |
| A_55_P2026614 | ref|XM_001477619|ref|XM_001477607 | LOC100042409 | -3.74 |
| A_51_P318830 | ref|NM_018803|ens|ENSMUST00000029441|gb|BC125634|gb|BC125632 | Syt10 | -3.74 |
| A_55_P2040497 | ref|NR_033523|gb|BY726472 | Gm11517 | -3.74 |
| A_55_P2415910 | gb|AK034487|riken|9330199F22 | 9330199F22Rik | -3.74 |
| A_30_P01018087 | Unknown | chr15:59966962-59972812_F | -3.73 |
| A_52_P357469 | ref|NM_010237|ref|NM_001159544|ens|ENSMUST00000019913|gb|AK052614 | Frk | -3.73 |
| A_51_P277836 | ref|NM_027048|ens|ENSMUST00000091364|gb|AK005786|gb|BY705940 | 1700008P02Rik | -3.73 |
| A_30_P01029477 | Unknown | chr6:126766225-126771309_F | -3.72 |
| A_55_P2415417 | ref|NR_033638|gb|X89690|tc|NP051724|nap|NAP103508-1 | Olfr29-ps1 | -3.72 |
| A_30_P01026313 | Unknown | chr11:95113972-95114267_F | -3.72 |
| A_30_P01022758 | Unknown | chrX:146832315-146875680_R | -3.71 |
| A_55_P2041614 | ref|NM_001048176|ens|ENSMUST00000143974|ens|ENSMUST00000156731|gb|BC046474 | Cerkl | -3.71 |
| A_65_P01834 | ref|NM_001113545|ref|NM_023063|ens|ENSMUST00000073691|ens|ENSMUST00000109024 | Lima1 | -3.71 |
| A_55_P1990400 | ref|NM_008313|ens|ENSMUST00000027560|gb|AK141625|gb|AK141587 | Htr4 | -3.70 |
| A_55_P2135296 | ens|ENSMUST00000097520|gb|BC147247|gb|BC147248|gb|AK158289 | ENSMUST00000097520 | -3.70 |
| A_30_P01020493 | Unknown | chr14:44151048-44156924_R | -3.69 |
| A_55_P2290931 | gb|BG067443 | C86371 | -3.69 |
| A_30_P01019363 | Unknown | chr5:150921318-150927422_F | -3.69 |
| A_51_P219160 | ens|ENSMUST00000113046|ref|XM_001473792|ref|XM_001475652|gb|AK016974 | 4933428M09Rik | -3.68 |
| A_55_P2001876 | ref|XM_001473461|ref|XM_001478979 | Gm6214 | -3.68 |
| A_55_P2093336 | ref|NM_013561|ref|NM_001099644|ens|ENSMUST00000003826|gb|AF513221 | Htr3a | -3.67 |
| A_66_P116597 | ref|XM_001480387|ref|XM_001480639|gb|AK019644|gb|BY715115 | 4930473D10Rik | -3.67 |
| A_55_P2011794 | ref|XM_911832 | LOC636441 | -3.67 |
| A_55_P2139231 | ref|NM_001085540|ens|ENSMUST00000105762 | Pramef17 | -3.66 |
| A_55_P2018862 | gb|AJ564870|gb|AJ564872|gb|AJ564879|gb|BC148704 | Defa-rs7 | -3.65 |
| A_30_P01030188 | Unknown | chr8:74333475-74384150_F | -3.65 |
| A_51_P109274 | ref|NM_134202|ens|ENSMUST00000056339|gb|BC115528|tc|NP496260 | Vmn1r233 | -3.65 |
| A_30_P01033125 | Unknown | chr8:67550064-67560987_R | -3.65 |
| A_51_P205028 | ref|NM_008007|ens|ENSMUST00000155320|gb|BC117061|gb|BC117059 | Fgf3 | -3.64 |
| A_55_P2127699 | ref|NM_011089|ref|NM_011093|ref|NM_008848|ens|ENSMUST00000071671 | Pira2 | -3.64 |
| A_55_P2098925 | ref|NM_145369|ens|ENSMUST00000045127|gb|AY669505|gb|AK131905 | Wfdc5 | -3.63 |
| A_55_P2042096 | ens|ENSMUST00000112869|ens|ENSMUST00000112867|gb|AK165913|tc|TC1682069 | ENSMUST00000112869 | -3.60 |
| A_55_P2049226 | ref|NM_053156|ens|ENSMUST00000020965|ens|ENSMUST00000110917|gb|AK005893 | Allc | -3.60 |
| A_55_P2166622 | ref|XR_030743 | LOC674362 | -3.60 |
| A_55_P2065260 | ref|XM_908200|nap|NAP112303-1 | LOC633720 | -3.60 |
| A_30_P01022603 | Unknown | chr17:56687541-56694616_F | -3.60 |
| A_55_P2113886 | ens|ENSMUST00000065540|tc|NP062288 | ENSMUST00000065540 | -3.59 |
| A_30_P01024337 | Unknown | chr1:196445304-196445818_R | -3.59 |
| A_55_P2185143 | ens|ENSMUST00000111181|ens|ENSMUST00000044972|ens|ENSMUST00000111182|gb|AK090036 | ENSMUST00000111181 | -3.58 |
| A_55_P2141978 | ref|NM_001083906|ens|ENSMUST00000034031|ens|ENSMUST00000148106|ens|ENSMUST00000109915 | Nr3c2 | -3.58 |
| A_66_P120380 | ref|NM_177820|ref|NM_001143686|ref|NM_001177533|ens|ENSMUST00000089465 | Apol10b | -3.58 |
| A_55_P2150013 | ref|XM_001481197 | LOC100048762 | -3.58 |
| A_55_P2057901 | ens|ENSMUST00000085981|gb|AK149266|riken|A930006O16 | ENSMUST00000085981 | -3.58 |
| A_30_P01028264 | Unknown | chr3:93159107-93159613_F | -3.57 |
| A_55_P2328082 | gb|AK142074|riken|D230021C23 | AI606473 | -3.57 |
| A_55_P2023057 | ref|NM_178055|ref|NM_001159885|ref|NM_001159884|ref|NM_020266 | Dnajb2 | -3.56 |
| A_55_P2169099 | ref|NM_010463|ens|ENSMUST00000055562|gb|AF448482|gb|BC120845 | Hoxc12 | -3.56 |
| A_52_P119350 | ref|XM_001478300|ref|XM_001478818|tc|TC1703548|nap|NAP036674-1 | 4732419C18Rik | -3.55 |
| A_30_P01030563 | Unknown | chr6:30127175-30128051_F | -3.54 |
| A_51_P490955 | ref|NM_001039532|ens|ENSMUST00000062428|gb|AK050452|tc|TC1582198 | Zfp784 | -3.53 |
| A_30_P01023693 | Unknown | chr10:66640530-66644032_F | -3.53 |
| A_52_P495372 | ref|NM_153796|ens|ENSMUST00000026227|gb|AK146244|gb|AK054159 | Peo1 | -3.53 |
| A_51_P225493 | ref|NM_027077|ens|ENSMUST00000016106|gb|AK006014|gb|BY706135 | 1700016C15Rik | -3.52 |
| A_51_P229676 | ref|NM_011636|ens|ENSMUST00000093801|ens|ENSMUST00000034930|gb|AF159593 | Plscr1 | -3.52 |
| A_30_P01025458 | Unknown | chr11:50994911-51050704_R | -3.52 |
| A_55_P2325568 | gb|AK164818|riken|D830045F20 | 1700060J05Rik | -3.52 |
| A_55_P1973120 | ref|NR_033566|ens|ENSMUST00000097812|gb|AK131719|gb|AV042767 | AI507597 | -3.51 |
| A_55_P2217747 | gb|AK016366|riken|4930588G17|nap|NAP023617-001 | 4930588G17Rik | -3.51 |
| A_55_P2003628 | ens|ENSMUST00000119995 | ENSMUST00000119995 | -3.51 |
| A_55_P2007185 | ref|NM_147010|ens|ENSMUST00000054746|gb|BC127979|tc|NP646622 | Olfr1052 | -3.50 |
| A_52_P233305 | ref|NM_175501|ens|ENSMUST00000061318|gb|AJ537452|gb|BC152338 | Adamts12 | -3.50 |
| A_51_P424854 | ref|NM_013465|ens|ENSMUST00000023583|gb|AK167709|gb|AK133523 | Ahsg | -3.50 |
| A_52_P49014 | ref|NM_009170|ens|ENSMUST00000002708|gb|AK077688|gb|AK052447 | Shh | -3.49 |
| A_52_P628212 | ens|ENSMUST00000110203|gb|AK044339|tc|TC1675974|riken|A930008D01 | ENSMUST00000110203 | -3.49 |
| A_55_P2404823 | gb|AK007074|tc|TC1694534|riken|1700095A21|nap|NAP120456-1 | 1700095A21Rik | -3.49 |
| A_51_P373728 | ref|NM_134219|ens|ENSMUST00000072972|tc|NP496246|nap|NAP057742-1 | Vmn1r215 | -3.48 |
| A_51_P257938 | ref|NM_020047|ens|ENSMUST00000058178|gb|BC117719|gb|BC117720 | Tacstd2 | -3.48 |
| A_30_P01031601 | Unknown | chr1:121971906-121988306_R | -3.48 |
| A_51_P108309 | ref|NM_146990|ens|ENSMUST00000051768|gb|BC104099|gb|BC104100 | Olfr1494 | -3.48 |
| A_55_P2293668 | gb|AI266897 | AI132709 | -3.47 |
| A_55_P2336078 | gb|AK016653|riken|4933404K13|nap|NAP073408-1 | 4933404K13Rik | -3.47 |
| A_55_P1953038 | ens|ENSMUST00000038090|gb|AK014905|tc|TC1577367|riken|4921517B04 | ENSMUST00000038090 | -3.47 |
| A_51_P333896 | ref|NM_146471|ens|ENSMUST00000078932|gb|BC104066|gb|BC104067 | Olfr1393 | -3.46 |
| A_51_P444822 | ref|NM_146360|ens|ENSMUST00000052997|gb|BC127050|tc|TC1599512 | Olfr574 | -3.45 |
| A_30_P01019567 | Unknown | chr9:36509939-36515389_F | -3.45 |
| A_30_P01033228 | Unknown | chr9:88575075-88611650_R | -3.45 |
| A_55_P1961466 | ref|NM_010024|ens|ENSMUST00000022725|gb|AK210881|gb|AK192007 | Dct | -3.45 |
| A_52_P590625 | ref|NM_175532|ens|ENSMUST00000055745|gb|AK028802|gb|BB003937 | Nlrp10 | -3.44 |
| A_52_P445360 | ref|NM_023256|ens|ENSMUST00000017743|gb|AK008079|gb|AK018567 | Krt20 | -3.44 |
| A_55_P1999725 | ref|XM_001472999|ref|XM_001475035 | 1700018P22Rik | -3.44 |
| A_55_P2040344 | ref|NM_177901|ens|ENSMUST00000093342|ref|XR_034804|gb|BC120779 | 4933402J07Rik | -3.44 |
| A_30_P01023741 | Unknown | chr5:107503800-107515575_F | -3.44 |
| A_55_P1957163 | ref|XM_001473071|ref|XM_001475011|tc|TC1643136 | Gm2297 | -3.43 |
| A_51_P310020 | ref|NM_146884|ens|ENSMUST00000111004|ens|ENSMUST00000099610|tc|TC1600228 | Olfr1299 | -3.43 |
| A_55_P2065953 | ref|NR_024078|gb|BB829614 | Btbd19 | -3.43 |
| A_55_P2012869 | ref|XR_034787 | LOC677636 | -3.41 |
| A_55_P2107711 | ref|NM_029838|ref|NM_198711|ens|ENSMUST00000080335|ens|ENSMUST00000106347 | Col25a1 | -3.41 |
| A_52_P641282 | ref|NM_027943|ens|ENSMUST00000033267|gb|AK005692|tc|TC1590165 | Pdilt | -3.41 |
| A_30_P01018136 | Unknown | chrX:11239288-11359919_R | -3.41 |
| A_55_P2240919 | gb|AK029486|tc|TC1626483|riken|4833447H21|nap|NAP088595-1 | A730032A03Rik | -3.41 |
| A_55_P2163541 | ref|NM_177872|ref|NM_001081401|ens|ENSMUST00000061427 | Adamts3 | -3.40 |
| A_55_P2098442 | ref|XM_001475331|ref|XM_001477206 | Gm3575 | -3.39 |
| A_55_P2033819 | ref|NM_001011527|ens|ENSMUST00000078162|nap|NAP098522-001 | Olfr503 | -3.39 |
| A_55_P2058388 | tc|TC1702193 | TC1702193 | -3.38 |
| A_55_P2070316 | ref|NM_023816|ens|ENSMUST00000020774|ens|ENSMUST00000109855|ens|ENSMUST00000020773 | Ankrd36 | -3.38 |
| A_55_P1983459 | ref|NM_207653|ens|ENSMUST00000097722|ens|ENSMUST00000069333|ens|ENSMUST00000114313 | Cflar | -3.38 |
| A_55_P2169989 | ens|ENSMUST00000054218|tc|TC1676117 | ENSMUST00000054218 | -3.38 |
| A_51_P388471 | ref|NM_027094|ens|ENSMUST00000022315|gb|AK006511|gb|BC049632 | Dydc1 | -3.37 |
| A_30_P01028407 | Unknown | chr9:53613193-53613871_F | -3.37 |
| A_30_P01027945 | Unknown | chr9:55786058-55797810_F | -3.36 |
| A_30_P01018124 | Unknown | chr14:103751075-103769725_F | -3.36 |
| A_55_P2006630 | ref|NM_001003671|ens|ENSMUST00000007584|ref|XM_908963|gb|BC160198 | Pcdhac1 | -3.36 |
| A_55_P2056595 | ref|NM_001034904|ens|ENSMUST00000098710|ens|ENSMUST00000098726|ens|ENSMUST00000108446 | Gm5891 | -3.36 |
| A_51_P324633 | ref|NM_007703|ens|ENSMUST00000043739|gb|U97107|tc|TC1584835 | Elovl3 | -3.36 |
| A_52_P573627 | ens|ENSMUST00000051236|gb|BC049804|tc|TC1701664 | ENSMUST00000051236 | -3.36 |
| A_55_P2331661 | gb|AK082631|gb|AK048631|riken|C130090I10|riken|C230071I02 | C230071I02Rik | -3.35 |
| A_55_P2075909 | ref|NM_001101579|nap|NAP062304-1 | Vmn1r12 | -3.35 |
| A_30_P01028945 | Unknown | chr13:114111261-114117957_F | -3.35 |
| A_55_P2086750 | ref|XM_001473468|ref|XM_001478820|gb|AK006860|tc|TC1678906 | 1700062C10Rik | -3.35 |
| A_55_P2068867 | ref|XM_001474724 | Gm2789 | -3.33 |
| A_55_P1971154 | ref|NM_199257|ens|ENSMUST00000068928|ens|ENSMUST00000033870|ens|ENSMUST00000077194 | Tpte | -3.33 |
| A_30_P01020830 | Unknown | chr8:60141721-60142223_F | -3.32 |
| A_55_P2226660 | gb|AK017178|tc|TC1716766|tc|TC1666651|riken|5033418A18 | 5033418A18Rik | -3.32 |
| A_66_P117519 | ens|ENSMUST00000099292|ref|XR_035472|ref|XR_035475|gb|AK144672 | ENSMUST00000099292 | -3.32 |
| A_55_P2111093 | ref|NM_207552|ens|ENSMUST00000052085|nap|NAP013710-001 | Olfr250 | -3.30 |
| A_55_P2353079 | gb|AK041809|gb|BB243423|riken|A630039O03|nap|NAP024874-001 | A630039O03Rik | -3.30 |
| A_55_P2114651 | ens|ENSMUST00000025390|ref|XM_128949|ref|XM_915395|gb|AK009098 | 2310002L13Rik | -3.30 |
| A_51_P521052 | ref|NM_029627|ens|ENSMUST00000060301|gb|AK010485|gb|BC049723 | Ly6k | -3.30 |
| A_51_P497240 | ref|NR_026942|ens|ENSMUST00000065383|gb|AK087735|gb|BC076607 | E330013P04Rik | -3.29 |
| A_30_P01023069 | Unknown | chr13:113205145-113207570_F | -3.29 |
| A_55_P1956147 | ref|NM_008081|ens|ENSMUST00000103158|gb|AK153938|tc|TC1651111 | B4galnt2 | -3.28 |
| A_30_P01026627 | Unknown | chr4:145593253-145594254_R | -3.27 |
| A_52_P382149 | ref|NM_007811|ens|ENSMUST00000025946|gb|BC012673|gb|Y12657 | Cyp26a1 | -3.27 |
| A_52_P70949 | ref|NM_001033904|gb|BC165941|gb|AK136166|gb|BC099381 | Khdc1c | -3.26 |
| A_55_P2158176 | ref|NM_139148|ens|ENSMUST00000029931|gb|BC156642|gb|AY008277 | Clca4 | -3.26 |
| A_55_P2040090 | ref|NM_001038699|ens|ENSMUST00000103014|gb|BC054374 | Fn3k | -3.26 |
| A_52_P214764 | ref|NR_027848|ref|NR_027849|ens|ENSMUST00000051495|gb|AK006932 | 4930479M11Rik | -3.26 |
| A_55_P2122075 | gb|AF142780 | Pdcd1lg2 | -3.26 |
| A_55_P2161485 | ref|NM_001083342|ens|ENSMUST00000116089|gb|AK160846|tc|TC1667852 | Ptchd2 | -3.25 |
| A_55_P2051476 | ref|NM_146148|ens|ENSMUST00000048947|ens|ENSMUST00000064873|ens|ENSMUST00000106808 | C8a | -3.24 |
| A_30_P01023551 | Unknown | chr4:89088538-89130181_F | -3.23 |
| A_30_P01025664 | Unknown | chr10:39607282-39619520_F | -3.23 |
| A_51_P314669 | ref|NM_011128|ens|ENSMUST00000026081|gb|M30687|gb|AK008021 | Pnliprp2 | -3.23 |
| A_30_P01021311 | Unknown | chr9:78107225-78118850_R | -3.23 |
| A_66_P113188 | ens|ENSMUST00000103492|gb|EU568202|gb|AF006586|gb|EF492992 | ENSMUST00000103492 | -3.22 |
| A_52_P149173 | ref|NM_001104644|nap|NAP026027-1|nap|NAP000154-001|nap|NAP000154-004 | Vmn2r53 | -3.22 |
| A_55_P2367350 | gb|AK016636|riken|4933403J19|nap|NAP073411-1 | 4933403J19Rik | -3.22 |
| A_55_P1993415 | ens|ENSMUST00000101355|ref|XR_035326|gb|AK134759|tc|TC1594514 | ENSMUST00000101355 | -3.22 |
| A_52_P547456 | ref|NM_026394|ens|ENSMUST00000047153|gb|AK003518|gb|BC060283 | Lce1f | -3.21 |
| A_55_P2359110 | gb|AK038413|riken|A230005M16|nap|NAP121480-001 | A230005M16Rik | -3.21 |
| A_55_P2126652 | ref|XR_032132 | LOC635773 | -3.21 |
| A_51_P257841 | ref|NM_028494|gb|AK006596|gb|AK077003|gb|AV209908 | 1700034I23Rik | -3.21 |
| A_55_P2024270 | ref|NM_146400|ens|ENSMUST00000053710|ens|ENSMUST00000104889|tc|TC1599953 | Olfr1288 | -3.21 |
| A_55_P2415069 | gb|AK015937|riken|4930529K09|nap|NAP074473-1 | 4930529K09Rik | -3.20 |
| A_55_P2175095 | ref|NM_011384|ens|ENSMUST00000021519|gb|AK029309|riken|4832418K20 | Six6 | -3.20 |
| A_55_P2102833 | ens|ENSMUST00000105709 | ENSMUST00000105709 | -3.20 |
| A_55_P1986586 | ref|NM_027598|ens|ENSMUST00000044829|ens|ENSMUST00000075174|gb|AK014961 | 4921524L21Rik | -3.20 |
| A_30_P01026959 | Unknown | chrX:93154123-93164481_F | -3.20 |
| A_30_P01032530 | Unknown | chr1:45569500-45580900_R | -3.20 |
| A_55_P2077505 | ref|XM_001474513|ref|XM_001476010 | Gm3021 | -3.19 |
| A_52_P198885 | ref|NM_207651|ref|NM_001110273|ens|ENSMUST00000025434|gb|AK032837 | Slc14a2 | -3.19 |
| A_55_P2147836 | ref|NM_001033427|ens|ENSMUST00000078307|gb|AK132442|tc|TC1589421 | Lyg2 | -3.18 |
| A_51_P446085 | ref|NM_028908|ens|ENSMUST00000053078|gb|AK080043|gb|AK220388 | 4933403G14Rik | -3.18 |
| A_55_P1997184 | ref|XM_001475088|ref|XM_001479447 | Gm4211 | -3.18 |
| A_55_P1971010 | ref|NM_010373|ref|NM_010372|ens|ENSMUST00000015588|ens|ENSMUST00000089549 | Gzme | -3.18 |
| A_30_P01022782 | Unknown | chr12:119836655-119984828_R | -3.18 |
| A_55_P2044212 | ref|NM_177787|ens|ENSMUST00000050132|ens|ENSMUST00000111873|gb|AK036413 | Slc15a5 | -3.17 |
| A_55_P1998319 | ref|XR_031396 | LOC100045098 | -3.17 |
| A_55_P2123185 | ens|ENSMUST00000060246|gb|AK003565|tc|TC1596041|riken|1110008E08 | ENSMUST00000060246 | -3.17 |
| A_55_P1964734 | ens|ENSMUST00000088320|tc|NP859915 | ENSMUST00000088320 | -3.16 |
| A_55_P2119932 | ref|XM_001479955 | LOC100048419 | -3.16 |
| A_55_P2132443 | ens|ENSMUST00000099208 | ENSMUST00000099208 | -3.16 |
| A_55_P2028375 | ref|XM_001474359 | LOC100045471 | -3.16 |
| A_52_P589550 | ref|NM_011629|ens|ENSMUST00000099343|ens|ENSMUST00000092213|ens|ENSMUST00000105290 | Nr2c1 | -3.15 |
| A_52_P447196 | ref|NM_053185|ens|ENSMUST00000033629|ens|ENSMUST00000101205|gb|AB041351 | Col4a6 | -3.15 |
| A_52_P121753 | ref|NM_207527|ens|ENSMUST00000064614|gb|AK076838|gb|BC048595 | 4930504O13Rik | -3.15 |
| A_55_P2056580 | ref|XM_001478837|ref|XM_001480922|gb|BF148553 | Gm4524 | -3.15 |
| A_52_P321831 | ref|NM_023511|ens|ENSMUST00000055502|gb|AK009119|gb|BC060278 | Krtap3-1 | -3.14 |
| A_55_P2164829 | ens|ENSMUST00000059648|ref|XM_354798|gb|AK087762|riken|E330017F02 | 4933413J09Rik | -3.14 |
| A_55_P2102400 | ens|ENSMUST00000027006 | ENSMUST00000027006 | -3.14 |
| A_55_P2052887 | ref|NM_146402|ens|ENSMUST00000099609|tc|TC1599988|nap|NAP101676-1 | Olfr1303 | -3.13 |
| A_55_P2424742 | gb|AK012975|tc|TC1589788|riken|2810403G11|nap|NAP120057-1 | LOC100036525 | -3.13 |
| A_55_P2113091 | ref|NM_201409|ens|ENSMUST00000076501|gb|BC137942|gb|BC145058 | Dub1a | -3.13 |
| A_55_P2029203 | ref|NM_026700|gb|AK002808|gb|AK164839|gb|AK156170 | Dopey2 | -3.13 |
| A_55_P2115876 | ref|XM_001478317|ref|XM_001479276|tc|TC1736506|nap|NAP114901-1 | LOC100047838 | -3.13 |
| A_55_P2120244 | ref|NM_010184|ens|ENSMUST00000049706|gb|J05018|tc|TC1587021 | Fcer1a | -3.13 |
| A_30_P01021151 | Unknown | chr6:100362389-100362920_R | -3.12 |
| A_52_P196632 | ref|NM_028974|gb|AK048611|gb|AK017385|tc|TC1586443 | Kbtbd13 | -3.12 |
| A_52_P88054 | ref|NM_007699|ens|ENSMUST00000045537|gb|S74916|gb|BC120504 | Chrm4 | -3.12 |
| A_55_P2040205 | ref|NM_213721|ref|NM_146629|ens|ENSMUST00000122036|ens|ENSMUST00000074555 | Olfr118 | -3.12 |
| A_30_P01022031 | Unknown | chr7:107226916-107227672_R | -3.11 |
| A_66_P114540 | ens|ENSMUST00000063891|gb|AK079848|tc|TC1618761|riken|A430092C21 | ENSMUST00000063891 | -3.11 |
| A_55_P2101696 | ref|NM_008141|ens|ENSMUST00000009252|ens|ENSMUST00000058669|gb|L10666 | Gnat2 | -3.11 |
| A_55_P2022048 | ref|NM_146795|ens|ENSMUST00000057775|gb|BC132194|gb|BC132196 | Olfr812 | -3.10 |
| A_51_P214998 | ens|ENSMUST00000036821|gb|AK019794|gb|BY715867|tc|TC1595925 | ENSMUST00000036821 | -3.10 |
| A_55_P2048808 | ens|ENSMUST00000100090|gb|AK133126|tc|TC1594428|riken|4930588K23 | ENSMUST00000100090 | -3.10 |
| A_51_P367752 | ref|NM_026150|ens|ENSMUST00000020712|gb|AK162162|gb|AK049235 | 4921536K21Rik | -3.10 |
| A_55_P2149535 | ens|ENSMUST00000085257|ref|XM_001473239|ref|XM_001473330|gb|AK077175 | ENSMUST00000085257 | -3.09 |
| A_66_P138053 | ens|ENSMUST00000066239|gb|AK044848|tc|TC1589242|riken|B130007O15 | ENSMUST00000066239 | -3.08 |
| A_30_P01032271 | Unknown | chr5:121669792-121732542_R | -3.08 |
| A_55_P2161903 | ref|NM_011025|ens|ENSMUST00000110265|ens|ENSMUST00000028764|gb|AK003042 | Oxt | -3.08 |
| A_52_P261496 | ref|NM_010249|ens|ENSMUST00000103226|gb|AY282805|gb|AY282802 | Gabpb1 | -3.08 |
| A_51_P185584 | ref|NM_001042714|ens|ENSMUST00000055607|gb|AK033960|gb|BC112417 | Ankdd1b | -3.08 |
| A_52_P1163890 | ref|NR_033556|ens|ENSMUST00000129643|ens|ENSMUST00000100165|ens|ENSMUST00000147024 | A630010A05Rik | -3.08 |
| A_55_P2168959 | ref|XM_001477497|ref|XM_001474135|ref|XR_033309|ref|XR_031374 | LOC100042366 | -3.08 |
| A_30_P01022988 | Unknown | chr3:90427029-90427674_F | -3.07 |
| A_55_P2061174 | ref|XM_001476006 | LOC100046317 | -3.07 |
| A_55_P2140255 | ref|NM_011709|ens|ENSMUST00000000391|ens|ENSMUST00000102910|gb|BC132280 | Wap | -3.07 |
| A_30_P01023660 | Unknown | chr3:85589154-85621215_R | -3.07 |
| A_55_P2111212 | ref|NR_033453|gb|AK020276|tc|TC1614970|riken|9130209A04 | 9130209A04Rik | -3.07 |
| A_55_P2376413 | gb|AK016745|riken|4933408N05|nap|NAP073507-1 | 4933408N05Rik | -3.06 |
| A_30_P01019291 | Unknown | chr14:78957377-78962977_F | -3.06 |
| A_55_P2005175 | ens|ENSMUST00000111875|gb|BC069904|tc|TC1644411 | ENSMUST00000111875 | -3.06 |
| A_52_P15461 | ref|NM_008357|ens|ENSMUST00000034148|gb|DQ083236|gb|AK155616 | Il15 | -3.06 |
| A_52_P592195 | ens|ENSMUST00000110481|gb|AK011044|gb|AK011096|tc|TC1630020 | ENSMUST00000110481 | -3.06 |
| A_51_P196695 | ref|NM_008372|ens|ENSMUST00000003981|gb|BC089571|tc|TC1574987 | Il7r | -3.05 |
| A_55_P2059200 | ref|NM_146818|ens|ENSMUST00000099798|tc|NP646776|nap|NAP022322-001 | Olfr1218 | -3.05 |
| A_30_P01030239 | Unknown | chr13:28506971-28511732_R | -3.04 |
| A_55_P2018691 | ref|NM_146744|gb|BC119336|gb|BC119338|tc|TC1600268 | Olfr1362 | -3.04 |
| A_51_P124126 | ref|NM_019823|ref|NM_001163472|ens|ENSMUST00000023083|gb|AF221525 | Cyp2d22 | -3.04 |
| A_55_P2158324 | ref|XM_136686|ref|XM_910074 | Gm4958 | -3.04 |
| A_55_P2132536 | ref|XM_001473400 | Gm2394 | -3.04 |
| A_55_P2069689 | ref|NM_146282|ens|ENSMUST00000078774|gb|BC134375|tc|NP647022 | Olfr846 | -3.04 |
| A_55_P2059035 | ref|XM_001471954|ref|XM_001478858 | Gm13403 | -3.04 |
| A_52_P302433 | ref|NM_008873|ens|ENSMUST00000022368|gb|X02389|tc|TC1587755 | Plau | -3.04 |
| A_55_P2106598 | ref|NM_001105254|ref|NM_178657|ens|ENSMUST00000110145|ens|ENSMUST00000095509 | Gm10436 | -3.03 |
| A_55_P1997305 | ref|XM_001478763|ref|XM_917116|nap|NAP063237-1 | Fam55a | -3.03 |
| A_55_P2014932 | ref|XM_001472658|ref|XM_001472812|gb|AK133228|riken|4932419C06 | Gm2245 | -3.03 |
| A_52_P409746 | ref|NM_080844|ens|ENSMUST00000064725|gb|BC011165|gb|S47225 | Serpinc1 | -3.03 |
| A_55_P2164492 | ens|ENSMUST00000099138|gb|AK152815|gb|AK150964|riken|I830086L09 | ENSMUST00000099138 | -3.02 |
| A_55_P2138281 | ref|XM_001477225 | LOC100047014 | -3.02 |
| A_52_P548377 | ref|NM_001033775|ens|ENSMUST00000093914|gb|BC147608|gb|BC157912 | 4933422H20Rik | -3.02 |
| A_51_P345995 | ref|NM_130857|gb|BC115545|gb|BC115546|gb|AF345295 | Krtap16-5 | -3.02 |
| A_55_P2171066 | ref|XM_001473735 | Gm2478 | -3.02 |
| A_30_P01018148 | Unknown | chr7:6296401-6311486_R | -3.02 |
| A_30_P01027861 | Unknown | chr5:125190100-125209625_R | -3.01 |
| A_55_P2051965 | ref|NM_001177508|ens|ENSMUST00000071294|ref|XM_993694|tc|NP1082806 | Olfr265 | -3.01 |
| A_55_P2369707 | gb|AK085082|tc|TC1594892|riken|D430034L15|nap|NAP086507-1 | Gm5919 | -3.01 |
| A_55_P2341368 | gb|AK136643|riken|9230016G08 | E030011O05Rik | -3.01 |
| A_66_P120309 | ens|ENSMUST00000118205|gb|AK042540|tc|TC1665740|tc|TC1593115 | ENSMUST00000118205 | -3.00 |
| A_30_P01027124 | Unknown | chr1:138477829-138478580_F | -3.00 |
| A_30_P01027573 | Unknown | chr1:85036677-85038530_R | -3.00 |
| A_30_P01021477 | Unknown | chr13:99465424-99465644_R | -3.00 |
| A_30_P01030285 | Unknown | chr16:90038086-90127636_F | -2.99 |
| A_55_P2022021 | ref|NM_001011819|ref|NM_146536|ens|ENSMUST00000078217|ens|ENSMUST00000082220 | Olfr312 | -2.99 |
| A_55_P2035286 | ref|NM_010931|ref|NM_001111079|ref|NM_001111078|ref|NM_001111080 | Uhrf1 | -2.99 |
| A_52_P627816 | ref|NM_019984|ref|NM_001161715|ref|NM_001161714|ens|ENSMUST00000002389 | Tgm1 | -2.99 |
| A_52_P223483 | ens|ENSMUST00000055404|ref|XR_035241|ref|XR_035312|gb|AK027987 | ENSMUST00000055404 | -2.99 |
| A_51_P468260 | ref|NM_009115|ens|ENSMUST00000036387|gb|AK135083|gb|AK134625 | S100b | -2.99 |
| A_55_P1960342 | ref|NM_009337|ens|ENSMUST00000041316|ens|ENSMUST00000101071|gb|AK149361 | Tcl1 | -2.99 |
| A_66_P112071 | ref|XM_001478594|tc|TC1682181|nap|NAP063521-1 | Gm3822 | -2.99 |
| A_55_P2009001 | ref|NM_053118|ens|ENSMUST00000032327|ens|ENSMUST00000111922|gb|AK137739 | Gprc5d | -2.98 |
| A_55_P2122884 | ref|NM_011195|ens|ENSMUST00000041012|gb|BC119062|gb|BC120746 | Ptcra | -2.98 |
| A_66_P126147 | ref|NM_001081180|ens|ENSMUST00000069245|gb|AK010042|gb|AK028727 | Spink5 | -2.97 |
| A_55_P2360356 | gb|AK016599|tc|NP741622|riken|4933401D09|nap|NAP073396-1 | 4933401D09Rik | -2.97 |
| A_55_P2081746 | ref|NM_146926|ens|ENSMUST00000091605|gb|BC140427|gb|BC156634 | Olfr477 | -2.97 |
| A_55_P2178882 | ens|ENSMUST00000050338|gb|AK003851|tc|NP359359|riken|1110020C17 | ENSMUST00000050338 | -2.97 |
| A_55_P2138956 | ens|ENSMUST00000150599|gb|AK039131|tc|TC1593426|riken|A230101D03 | ENSMUST00000150599 | -2.96 |
| A_55_P2139546 | ref|NM_028189|ens|ENSMUST00000034260|tc|TC1583281 | B3gnt3 | -2.96 |
| A_55_P2286951 | gb|AK015568|riken|4930473O22|nap|NAP074334-1 | 4930473O22Rik | -2.96 |
| A_55_P2157530 | ref|XM_001479304|ref|XM_001479970 | Gm4360 | -2.94 |
| A_55_P2161635 | ref|NM_001126325|ref|NM_177571|ens|ENSMUST00000105772|ens|ENSMUST00000105771 | Gm13088 | -2.94 |
| A_30_P01032848 | Unknown | chrX:31867450-32124664_F | -2.94 |
| A_55_P2048134 | ref|NM_030614 | Fgf16 | -2.94 |
| A_55_P2107382 | ref|XM_916585 | LOC639943 | -2.94 |
| A_30_P01021071 | Unknown | chr12:111617728-111665403_F | -2.94 |
| A_55_P2147126 | ref|NM_001166218 | Zfp712 | -2.94 |
| A_55_P2175885 | ref|NM_016960|ref|NM_001159738|ens|ENSMUST00000027351|ens|ENSMUST00000113437 | Ccl20 | -2.94 |
| A_30_P01026563 | Unknown | chr15:5975553-5994636_R | -2.93 |
| A_55_P2161987 | ens|ENSMUST00000078427|ref|XM_001475584|ref|XM_001480401 | ENSMUST00000078427 | -2.93 |
| A_55_P2029176 | ens|ENSMUST00000098471|gb|AK142244|riken|D330020L22 | ENSMUST00000098471 | -2.92 |
| A_55_P2361927 | gb|AK015599|tc|TC1600640|riken|4930480G23|nap|NAP074308-1 | 4930480G23Rik | -2.92 |
| A_55_P2067553 | ref|XM_484242|ref|XM_921970|gb|DN174638|tc|TC1679990 | Gm5448 | -2.91 |
| A_55_P2159139 | ref|NM_146608|ens|ENSMUST00000056795|tc|NP830853|nap|NAP021410-001 | Olfr984 | -2.91 |
| A_52_P182118 | ref|XR_031890|ref|XR_032623|nap|NAP058998-1 | Gm7776 | -2.91 |
| A_51_P256747 | ref|NM_173029|ens|ENSMUST00000027852|ens|ENSMUST00000111439|ens|ENSMUST00000111440 | Adcy10 | -2.91 |
| A_55_P1963384 | ref|XM_001472070|ref|XM_001473342|nap|NAP114886-1|nap|NAP114545-1 | LOC100044361 | -2.90 |
| A_51_P478581 | ref|NM_028180|ens|ENSMUST00000062631|gb|BC048613|gb|AK006542 | 1700030F18Rik | -2.90 |
| A_30_P01020995 | Unknown | chr4:132641388-132645061_F | -2.90 |
| A_55_P2195202 | gb|BU938278 | 1700016K05Rik | -2.90 |
| A_55_P2128073 | ens|ENSMUST00000014063|ens|ENSMUST00000107970|gb|AK009217|riken|2310008B01 | ENSMUST00000014063 | -2.90 |
| A_30_P01023409 | Unknown | chr8:26347473-26348052_R | -2.90 |
| A_55_P2057168 | ref|XM_001480650 | LOC100048601 | -2.90 |
| A_55_P2329973 | gb|AK032445|tc|TC1617883|riken|6430548G04|nap|NAP075102-1 | 6430548G04 | -2.90 |
| A_55_P2077473 | Unknown | A_55_P2077473 | -2.90 |
| A_52_P393738 | ref|NM_182785|ens|ENSMUST00000079306|gb|AK077071|gb|BC049744 | Lypd4 | -2.90 |
| A_55_P2084089 | ens|ENSMUST00000093081|ens|ENSMUST00000108834|ref|XM_916080|ref|XM_001477470 | ENSMUST00000093081 | -2.89 |
| A_30_P01018381 | Unknown | chr1:33893475-33903900_R | -2.89 |
| A_55_P2285599 | gb|AK017624|tc|TC1635769|riken|5730437C12|nap|NAP100258-001 | 5730437C12Rik | -2.89 |
| A_55_P1981589 | ref|NM_147110|ens|ENSMUST00000098215|gb|BC116966|tc|NP646532 | Olfr570 | -2.88 |
| A_30_P01026657 | Unknown | chr13:60508775-60534625_F | -2.88 |
| A_55_P2149736 | ref|NM_134182|ens|ENSMUST00000089830|gb|BC125351|gb|BC132235 | Vmn1r19 | -2.88 |
| A_30_P01026777 | Unknown | chr8:19959345-20020379_R | -2.88 |
| A_55_P2416837 | gb|AK006991|riken|1700084F23|nap|NAP091853-1 | 1700084F23Rik | -2.88 |
| A_55_P2091297 | ens|ENSMUST00000108646 | ENSMUST00000108646 | -2.88 |
| A_51_P189082 | ref|NM_030611|ens|ENSMUST00000021630|gb|D45850|gb|BC056643 | Akr1c6 | -2.87 |
| A_30_P01031101 | Unknown | chr18:36480559-36481146_R | -2.87 |
| A_52_P148952 | ref|NM_001127576|ens|ENSMUST00000100378|tc|TC1637868|nap|NAP039003-1 | Gm1564 | -2.87 |
| A_55_P1980139 | ref|NM_001123362|ens|ENSMUST00000113470 | Prdm12 | -2.87 |
| A_55_P2089577 | ref|XM_001476475|ref|XM_001476494|gb|AK172643|riken|F830223E18 | LOC100046567 | -2.87 |
| A_30_P01020254 | Unknown | chr6:5311375-5328075_R | -2.87 |
| A_30_P01023967 | Unknown | chr1:42972297-42972716_R | -2.86 |
| A_52_P399140 | ref|NM_028411|ens|ENSMUST00000025568|gb|BC107026|gb|BC132574 | Tmem138 | -2.86 |
| A_55_P2382070 | gb|AK017132|riken|4933440I01|nap|NAP073645-1 | 4930593A02Rik | -2.86 |
| A_55_P2118794 | ref|NM_172801|ens|ENSMUST00000055490|ens|ENSMUST00000106544|gb|AK028866 | Otop2 | -2.86 |
| A_30_P01032987 | Unknown | chr13:66381200-66410200_R | -2.86 |
| A_30_P01019354 | Unknown | chr8:87504644-87513694_F | -2.85 |
| A_55_P2017343 | ref|NM_146318|ens|ENSMUST00000082131|gb|BC137767|tc|TC1600279 | Olfr767 | -2.85 |
| A_55_P1996290 | ref|XM_001473079|ref|XM_001473934 | Gm2550 | -2.85 |
| A_55_P2193487 | gb|AA986695 | AA986695 | -2.85 |
| A_66_P138206 | ref|NM_146560|ens|ENSMUST00000086474|gb|BC120630|gb|BC120632 | Olfr872 | -2.84 |
| A_30_P01023398 | Unknown | chr1:59775022-59780348_F | -2.84 |
| A_51_P207380 | ref|NM_199023|ens|ENSMUST00000132409|ens|ENSMUST00000052416|gb|AK076877 | 4930526D03Rik | -2.84 |
| A_55_P2165510 | ref|XM_001472435|ref|XM_001478737|ref|XM_001479345 | LOC100048825 | -2.84 |
| A_55_P1974740 | ref|NM_019481|ens|ENSMUST00000115350|ens|ENSMUST00000031713|gb|AF199366 | Slc13a1 | -2.84 |
| A_30_P01027300 | Unknown | chr17:84355587-84356863_F | -2.83 |
| A_55_P2070105 | ref|XM_001476213 | LOC100046442 | -2.83 |
| A_30_P01026975 | Unknown | chr2:118408095-118415745_F | -2.83 |
| A_30_P01028950 | Unknown | chr12:111322030-111323387_F | -2.83 |
| A_30_P01025084 | Unknown | chr3:146014132-146014545_R | -2.83 |
| A_66_P126662 | ref|XM_001478080|ref|XM_001480517|ref|XM_001480579|gb|AK076313 | 4732414G09Rik | -2.83 |
| A_55_P1998882 | ref|XM_975198|gb|AK135898|gb|AK162140|riken|7420434O15 | Gm7538 | -2.82 |
| A_55_P1996181 | ref|NM_001103368 | Vmn2r80 | -2.82 |
| A_30_P01020060 | Unknown | chr3:36280098-36347798_R | -2.82 |
| A_55_P1976142 | ens|ENSMUST00000098321 | ENSMUST00000098321 | -2.82 |
| A_51_P474053 | ref|NM_001166206|ens|ENSMUST00000040941|gb|AK005451|gb|BC099428 | Erv3 | -2.82 |
| A_55_P1955252 | ref|XM_001474354 | Gm2678 | -2.82 |
| A_55_P1989175 | ref|NM_146433|ens|ENSMUST00000099925|tc|NP830796|nap|NAP021420-001 | Olfr994 | -2.82 |
| A_51_P174914 | ref|NM_146686|ens|ENSMUST00000073507|gb|BC132238|gb|BC132388 | Olfr235 | -2.81 |
| A_55_P2374212 | gb|AK016084|riken|4930550C17|nap|NAP074568-1 | 4930550C17Rik | -2.81 |
| A_55_P2053459 | ref|NM_001161355|ref|NM_134249|ref|NM_001161356|ens|ENSMUST00000055102 | Timd2 | -2.81 |
| A_55_P2016049 | ref|NM_001163014|ens|ENSMUST00000108590|gb|AK079424|tc|TC1624504 | Gp6 | -2.81 |
| A_55_P2121046 | ref|XR_005060 | LOC677422 | -2.81 |
| A_55_P2140271 | ens|ENSMUST00000104981|gb|BG071895 | ENSMUST00000104981 | -2.81 |
| A_30_P01028320 | Unknown | chr10:126151060-126156450_R | -2.81 |
| A_52_P121491 | ref|NM_177092|ens|ENSMUST00000092143|gb|AK028036|gb|AK137163 | Msrb3 | -2.80 |
| A_52_P67007 | ens|ENSMUST00000061675|ref|XM_001479023|ref|XM_001480951|gb|AK006338 | 1700025H01Rik | -2.80 |
| A_55_P2074801 | ref|NM_008976|ens|ENSMUST00000027899|ens|ENSMUST00000097442|ens|ENSMUST00000027898 | Ptpn14 | -2.79 |
| A_52_P5567 | ref|NM_138746|ens|ENSMUST00000039605|gb|BC049659|gb|AK016195 | Fam50b | -2.79 |
| A_30_P01019182 | Unknown | chr1:138473514-138476054_R | -2.79 |
| A_66_P138634 | ref|XM_001478841 | Gm15264 | -2.79 |
| A_55_P1980391 | ref|XM_001473826 | LOC100045192 | -2.79 |
| A_55_P1969176 | ref|XM_001473558|ref|XM_001479812 | B130016D09Rik | -2.79 |
| A_55_P1976949 | ens|ENSMUST00000105334|gb|AB109391 | ENSMUST00000105334 | -2.79 |
| A_30_P01032103 | Unknown | chr9:70017793-70045355_R | -2.79 |
| A_55_P2185553 | ref|NM_020051|ens|ENSMUST00000035372|gb|AJ277605|gb|AB046448 | Ascl3 | -2.79 |
| A_55_P1955488 | ref|XM_001487782|ref|XM_001476567 | Gm7817 | -2.78 |
| A_55_P1965584 | ens|ENSMUST00000116120|ref|XM_001004193|gb|AK133004|tc|TC1641181 | ENSMUST00000116120 | -2.78 |
| A_52_P469789 | ref|NM_007588|ref|NM_001042725|ens|ENSMUST00000075644|ens|ENSMUST00000115622 | Calcr | -2.78 |
| A_30_P01031358 | Unknown | chr9:40033002-40033539_R | -2.78 |
| A_55_P2042494 | ens|ENSMUST00000118071|tc|TC1722947|nap|NAP051741-1 | ENSMUST00000118071 | -2.77 |
| A_55_P2105507 | ref|XM_001473690|ref|XM_001481043|gb|CX242839 | Gm4681 | -2.77 |
| A_55_P2091149 | ens|ENSMUST00000097347|ens|ENSMUST00000142317|ens|ENSMUST00000139063|tc|TC1726346 | ENSMUST00000097347 | -2.77 |
| A_30_P01020945 | Unknown | chr12:88235624-88264465_F | -2.77 |
| A_55_P2116435 | ref|NM_010951|ens|ENSMUST00000026383|nap|NAP099407-001 | Gpr143 | -2.77 |
| A_55_P2069550 | ref|XM_001478560 | Gm6750 | -2.76 |
| A_30_P01023965 | Unknown | chr3:50320320-50411144_R | -2.76 |
| A_55_P2019557 | ref|NM_153101|ref|NM_001172588|ens|ENSMUST00000119798|ens|ENSMUST00000066821 | Mrgpra2b | -2.76 |
| A_55_P2127223 | ens|ENSMUST00000056170|gb|AK076716|tc|TC1593373|riken|4930420A19 | ENSMUST00000056170 | -2.76 |
| A_55_P2248095 | gb|AK019787|riken|4930565N06|nap|NAP022748-001 | 4930565N06Rik | -2.76 |
| A_30_P01025466 | Unknown | chr13:98038568-98068079_F | -2.76 |
| A_55_P2376363 | gb|BC016220|gb|AK019591|tc|TC1592287|riken|4930432F04 | 4930432F04Rik | -2.76 |
| A_52_P504342 | ref|NM_027250|ens|ENSMUST00000131656|gb|AK168344|gb|AK008518 | 2010305A19Rik | -2.76 |
| A_55_P2305900 | gb|CA481107 | AA617406 | -2.76 |
| A_30_P01032820 | Unknown | chr15:3230734-3251196_R | -2.76 |
| A_55_P2399208 | gb|AK172666|riken|F830226J21 | 4930472D12Rik | -2.75 |
| A_55_P2060736 | ref|NM_207271|ens|ENSMUST00000081780|ref|XM_142982|tc|NP955491 | Tdpoz3 | -2.75 |
| A_55_P2391759 | gb|AK084979|riken|D430021D02|nap|NAP086486-1 | 2010007H06Rik | -2.75 |
| A_55_P2074488 | ref|NM_015800|ens|ENSMUST00000112503|ens|ENSMUST00000024876|ens|ENSMUST00000112498 | Crim1 | -2.74 |
| A_51_P401184 | ref|NM_001164763|ens|ENSMUST00000054825|gb|AK077393|tc|TC1610913 | Rarres1 | -2.74 |
| A_30_P01022563 | Unknown | chr8:87513394-87514107_R | -2.74 |
| A_30_P01023189 | Unknown | chr15:66682293-66722977_F | -2.74 |
| A_55_P2066458 | ref|XM_891592|ref|XM_910744|nap|NAP027415-1|nap|NAP113176-1 | Gm6725 | -2.73 |
| A_51_P420577 | ref|NM_146827|ens|ENSMUST00000050996|tc|TC1600009|nap|NAP057851-1 | Olfr983 | -2.73 |
| A_55_P2103011 | ref|NM_013660|ens|ENSMUST00000021900|ens|ENSMUST00000110040|ens|ENSMUST00000110039 | Sema4d | -2.73 |
| A_66_P128733 | ref|XM_001480181|ref|XM_001473151|gb|BC106175|tc|TC1656832 | BC106175 | -2.73 |
| A_55_P2108216 | ref|XM_001472666|ref|XM_001476710 | Gm3437 | -2.73 |
| A_52_P664656 | ref|NM_007396|ens|ENSMUST00000112783|ens|ENSMUST00000063886|gb|AK041246 | Acvr2a | -2.73 |
| A_66_P114616 | ref|NR_033595|nap|NAP060825-1 | Gm13498 | -2.73 |
| A_55_P2006158 | ref|XM_001475590 | LOC100046110 | -2.73 |
| A_55_P2042778 | ref|NM_153422|ens|ENSMUST00000066728|gb|AF541937|tc|TC1589900 | Pde5a | -2.72 |
| A_55_P2186822 | ens|ENSMUST00000091541|ref|XM_981055|gb|AK010804|tc|TC1596545 | Gm7896 | -2.72 |
| A_55_P2089060 | ens|ENSMUST00000108245 | ENSMUST00000108245 | -2.72 |
| A_55_P2064955 | ref|XM_001477789|ref|XM_001479434|gb|AK053416|tc|TC1616236 | Gm3728 | -2.72 |
| A_30_P01028782 | Unknown | chr2:65240793-65292293_F | -2.71 |
| A_52_P465946 | ref|NM_146858|ens|ENSMUST00000095085|gb|BC101951|gb|BC141896 | Olfr275 | -2.71 |
| A_30_P01027527 | Unknown | chr8:73645975-73696275_R | -2.71 |
| A_30_P01018281 | Unknown | chr5:75183748-75184179_R | -2.71 |
| A_55_P1971433 | ref|NM_011460|gb|AK028382|tc|TC1591590|riken|3830431C16 | Serpinb9d | -2.71 |
| A_55_P2147296 | ref|NR_027958|ens|ENSMUST00000064493|ens|ENSMUST00000117859|gb|AK086740 | D930048N14Rik | -2.71 |
| A_51_P248638 | ref|NM_021503|ens|ENSMUST00000029761|gb|AY013296|gb|AJ252148 | Myoz2 | -2.71 |
| A_51_P333518 | ref|NM_172966|ref|NM_001146299|ens|ENSMUST00000074679|ens|ENSMUST00000072008 | Sh3rf2 | -2.71 |
| A_55_P2054372 | ref|NM_001170955|ref|NR_003146|gb|AY746431|gb|U03028 | Gm15299 | -2.70 |
| A_66_P127796 | ref|XM_001475445|ref|XM_001476072|gb|GU144514|gb|AK005822 | LOC100046038 | -2.70 |
| A_30_P01021458 | Unknown | chr2:71205705-71226510_F | -2.70 |
| A_55_P2080789 | ref|NM_173432|ens|ENSMUST00000049699|gb|AK154570|gb|AK215598 | Pskh1 | -2.69 |
| A_55_P2119204 | ref|NM_146682|ens|ENSMUST00000087822|gb|BC138072|gb|BC138073 | Olfr76 | -2.69 |
| A_30_P01028686 | Unknown | chr4:155564927-155571580_R | -2.69 |
| A_55_P2145356 | ens|ENSMUST00000025099 | ENSMUST00000025099 | -2.69 |
| A_30_P01024561 | Unknown | chr15:60989423-60997290_F | -2.69 |
| A_55_P2106379 | ref|NM_001018019|ens|ENSMUST00000094715|gb|AY616753|tc|TC1594340 | Grxcr1 | -2.68 |
| A_30_P01027466 | Unknown | chr4:35106125-35127700_R | -2.68 |
| A_30_P01028503 | Unknown | chr2:174982600-175005825_F | -2.68 |
| A_66_P108468 | ref|XM_980662|ref|XM_987802|gb|AK012157|gb|BC049685 | 2610528A11Rik | -2.68 |
| A_52_P63855 | ref|NM_199422|ens|ENSMUST00000079286|gb|AY465109|tc|TC1592055 | S100a7a | -2.68 |
| A_51_P141546 | ref|NM_011016|ens|ENSMUST00000075341|gb|M27009|gb|M12566 | Orm2 | -2.68 |
| A_55_P2268790 | gb|AK015390|riken|4930445G23|nap|NAP074116-1 | 4930445G23Rik | -2.67 |
| A_51_P426875 | ref|NM_024228|ens|ENSMUST00000032944|gb|AK003726|gb|BC002172 | Gdpd3 | -2.67 |
| A_55_P2125114 | ref|NM_001085541|ref|NM_001085540|ens|ENSMUST00000105751|ens|ENSMUST00000105762 | Gm13128 | -2.67 |
| A_30_P01033048 | Unknown | chr3:85784436-85784624_R | -2.67 |
| A_55_P2143105 | ref|NR_033584|ens|ENSMUST00000093117|gb|AY512935|tc|TC1592813 | Gm10280 | -2.67 |
| A_55_P1968340 | ref|NM_181073|ens|ENSMUST00000021546|ens|ENSMUST00000039928|gb|AK052692 | Plekhh1 | -2.67 |
| A_51_P486971 | ens|ENSMUST00000032506|gb|AK016804|tc|TC1591108|riken|4933413G19 | ENSMUST00000032506 | -2.66 |
| A_55_P2062279 | ref|NM_146523|ens|ENSMUST00000115568|ens|ENSMUST00000077347|gb|BC113136 | Olfr850 | -2.66 |
| A_52_P62011 | ref|NR_033518|gb|AK009885|gb|AK136084|gb|BC061003 | Gm16039 | -2.66 |
| A_55_P2117287 | ref|XM_001479930|ref|XM_001477287 | Gm9636 | -2.66 |
| A_55_P2161671 | ref|XR_033956 | LOC100047791 | -2.66 |
| A_52_P559975 | ref|NM_009909|ens|ENSMUST00000027372|gb|AK158234|gb|BC051677 | Cxcr2 | -2.66 |
| A_30_P01022229 | Unknown | chr8:124648600-124731250_R | -2.66 |
| A_30_P01018036 | Unknown | chr14:73746324-73749119_R | -2.65 |
| A_30_P01028115 | Unknown | chr9:27153416-27153677_F | -2.65 |
| A_55_P2106358 | ref|XM_001478945 | Gm4077 | -2.65 |
| A_55_P2398089 | gb|AK045073|gb|BE949301|riken|B130024M06|nap|NAP124764-1 | B130024M06Rik | -2.65 |
| A_55_P1981401 | ref|XM_001480390 | LOC100048510 | -2.65 |
| A_55_P2363652 | gb|AK019498|riken|4632415I09|nap|NAP121284-001 | AK019498 | -2.65 |
| A_52_P412574 | ref|NM_183131|ens|ENSMUST00000061695|gb|AK019625|gb|BC048622 | 4930451I11Rik | -2.64 |
| A_51_P406796 | ref|NM_013873|ens|ENSMUST00000082365|gb|AF059257|gb|AK010293 | Sult4a1 | -2.63 |
| A_66_P131494 | ref|XM_001000764|ref|XM_982552|nap|NAP049722-1 | Gm9096 | -2.63 |
| A_55_P1981276 | ref|XM_001472979|ref|XM_001474323|gb|AK131772|riken|1700102N24 | Gm2582 | -2.63 |
| A_55_P2014460 | ref|NM_007539|ens|ENSMUST00000041229 | Bdkrb1 | -2.63 |
| A_30_P01017579 | Unknown | chr1:138442536-138521080_R | -2.62 |
| A_66_P100284 | ens|ENSMUST00000058450|gb|AK079763|tc|TC1591960|riken|A430060F13 | ENSMUST00000058450 | -2.62 |
| A_66_P114875 | ref|XM_001479501|ref|XM_001481104|gb|AK005780|tc|TC1633139 | 1700008K24Rik | -2.62 |
| A_55_P2001459 | ref|NM_178889|ens|ENSMUST00000079024|ref|XM_001480554|ref|XM_001480624 | Zscan29 | -2.62 |
| A_30_P01019194 | Unknown | chrX:155945151-155945654_R | -2.62 |
| A_52_P670188 | ref|NM_027301|ens|ENSMUST00000047134|gb|BC064820 | Sdr9c7 | -2.62 |
| A_30_P01032907 | Unknown | chr5:106353430-106353843_F | -2.61 |
| A_30_P01031306 | Unknown | chr14:52874069-52877398_R | -2.61 |
| A_30_P01024639 | Unknown | chr6:28870220-28870692_R | -2.61 |
| A_66_P136454 | ref|XM_989761|ref|XM_001477095|gb|AK006733|tc|TC1649160 | 1700048O14Rik | -2.61 |
| A_51_P128397 | ref|NM_015743|gb|AB221628|gb|AF191211|gb|BC128308 | Nr4a3 | -2.60 |
| A_30_P01026140 | Unknown | chr8:105795290-105864030_F | -2.60 |
| A_30_P01024608 | Unknown | chr15:41288197-41522572_R | -2.60 |
| A_55_P2014174 | ref|XM_001475643|ref|XM_001477468|gb|AK046843|riken|B830024C08 | 9130004C02Rik | -2.60 |
| A_55_P2100460 | ref|XM_001001937|ref|XM_001003092|gb|AK145257|riken|G930028K18 | Gm8781 | -2.60 |
| A_51_P225903 | ref|NM_173070|ens|ENSMUST00000062129|gb|AY158996|gb|BC107019 | Sprr4 | -2.60 |
| A_55_P1982444 | ref|XM_001474139 | LOC100045362 | -2.60 |
| A_55_P2080225 | ref|NM_172695|gb|AK153616|riken|A630021O04 | Plaa | -2.60 |
| A_51_P413910 | ref|NM_008257|ens|ENSMUST00000046093|gb|X75330|tc|TC1599408 | Hmx3 | -2.60 |
| A_55_P2150228 | ref|NM_018878|ens|ENSMUST00000002291|gb|BC066014|tc|TC1575974 | Paxip1 | -2.59 |
| A_52_P372418 | ens|ENSMUST00000103280|gb|AF126462|gb|M87851|gb|M87849 | ENSMUST00000103280 | -2.59 |
| A_55_P2127080 | ref|NM_001005481|ens|ENSMUST00000087128|gb|BC107196|gb|BC165981 | Olfr132 | -2.59 |
| A_51_P389265 | ref|NM_054088|gb|AY037763|tc|TC1582216|nap|NAP100813-001 | Pnpla3 | -2.59 |
| A_30_P01020808 | Unknown | chr18:3003075-3016150_F | -2.59 |
| A_30_P01025174 | Unknown | chr7:131819366-131852866_F | -2.58 |
| A_52_P532355 | ref|NM_001031772|ens|ENSMUST00000099849|ens|ENSMUST00000105489|ens|ENSMUST00000079390 | Lin28b | -2.58 |
| A_51_P448127 | ref|NM_025890|ens|ENSMUST00000034737|gb|AB211060|gb|AK010377 | 2410004A20Rik | -2.58 |
| A_55_P2345939 | gb|AK015485|tc|TC1691490|riken|4930458K08|nap|NAP074171-1 | 4930458K08Rik | -2.58 |
| A_55_P1962713 | ens|ENSMUST00000103340|nap|NAP062343-1 | ENSMUST00000103340 | -2.58 |
| A_30_P01031808 | Unknown | chr17:88466300-88495175_F | -2.58 |
| A_52_P618932 | ref|NM_029959|ens|ENSMUST00000023978|gb|BC140235|gb|AK020305 | Lcn9 | -2.58 |
| A_55_P2103006 | ref|XM_001475063 | Gm9485 | -2.57 |
| A_30_P01026298 | Unknown | chr1:64696508-64699504_F | -2.57 |
| A_55_P2180454 | ref|XM_001474560|ref|XM_001473512|ref|XM_001474580|nap|NAP063315-1 | Gm2739 | -2.57 |
| A_30_P01032992 | Unknown | chr12:20996314-21091646_F | -2.57 |
| A_55_P2166282 | ens|ENSMUST00000110529 | ENSMUST00000110529 | -2.57 |
| A_51_P464394 | ref|NM_031180|ens|ENSMUST00000031096|gb|BC138008|gb|BC138010 | Klb | -2.57 |
| A_55_P2042808 | ref|XM_001472736|ref|XM_001474030|tc|TC1721149 | Gm2177 | -2.57 |
| A_66_P120018 | ref|XR_002016|ref|XR_033771|gb|CK379678|nap|NAP060548-1 | Gm5821 | -2.57 |
| A_55_P1975927 | ref|XM_001480350|ref|XM_001480435 | Gm4550 | -2.57 |
| A_55_P2133470 | ref|NM_001146329|ens|ENSMUST00000032598|ref|XM_001475332 | Sbk2 | -2.57 |
| A_51_P288341 | ref|NM_008277|ens|ENSMUST00000144679|ens|ENSMUST00000031398|gb|X59530 | Hpd | -2.57 |
| A_55_P2143366 | ref|XM_001474047|nap|NAP018839-001 | LOC100044075 | -2.56 |
| A_55_P2149921 | ref|XM_001472863|tc|TC1780716 | LOC100044692 | -2.56 |
| A_30_P01028269 | Unknown | chr3:125556184-125556476_R | -2.56 |
| A_55_P2106255 | ref|NM_134163|tc|TC1623327 | Mbnl3 | -2.56 |
| A_55_P2336188 | gb|BF662369 | 6330564D18Rik | -2.56 |
| A_55_P2015620 | ref|NM_146568|ens|ENSMUST00000111595|ens|ENSMUST00000099916|gb|BC111581 | Olfr1012 | -2.56 |
| A_51_P377179 | ref|NM_001145259|ens|ENSMUST00000037090|ref|XM_001477913|gb|AK006077 | 1700018A14Rik | -2.55 |
| A_66_P139052 | ref|NM_001039038|ens|ENSMUST00000088234|ens|ENSMUST00000085027|gb|BC147284 | Nhlrc4 | -2.55 |
| A_55_P2114594 | ref|NM_001025353|ens|ENSMUST00000095430|ens|ENSMUST00000115930|ref|XM_001472172 | Gm6040 | -2.55 |
| A_30_P01021950 | Unknown | chr3:36298850-36299384_F | -2.55 |
| A_55_P1960631 | ref|NM_199473|ens|ENSMUST00000106130|ens|ENSMUST00000070132|gb|AK131153 | Col8a2 | -2.55 |
| A_51_P221823 | ref|NM_028621|ens|ENSMUST00000078422|gb|AF345297|gb|BC147776 | Krtap16-7 | -2.54 |
| A_55_P2046709 | ref|XR_032416|ref|XR_032235 | Gm8893 | -2.54 |
| A_51_P330452 | ref|NM_147039|ens|ENSMUST00000062353|gb|BC120830|tc|TC1600103 | Olfr1414 | -2.54 |
| A_55_P2246269 | gb|AK015322|riken|4930435E18|nap|NAP074146-1 | 4933409F18Rik | -2.54 |
| A_55_P2380890 | gb|BQ552818|tc|TC1622180 | AU040958 | -2.54 |
| A_52_P418814 | ref|NM_029971|ens|ENSMUST00000048621|gb|AK138363|gb|AK020723 | Pmch | -2.54 |
| A_55_P2303868 | gb|AK050516|tc|TC1613479|riken|C820006M04|nap|NAP117042-1 | 5033421B08Rik | -2.54 |
| A_30_P01027410 | Unknown | chr1:4889810-4890012_R | -2.54 |
| A_55_P2024041 | ref|NM_011964|ens|ENSMUST00000081213|ens|ENSMUST00000004657|gb|X98111 | Psg19 | -2.53 |
| A_55_P2391619 | gb|BG063913 | AI449595 | -2.53 |
| A_30_P01018175 | Unknown | chr14:26093652-26208156_F | -2.53 |
| A_30_P01022325 | Unknown | chr11:102833388-102833781_F | -2.53 |
| A_55_P2161200 | ens|ENSMUST00000103567|gb|AF012171|tc|NP050710|tc|TC1706189 | ENSMUST00000103567 | -2.53 |
| A_55_P2077458 | ens|ENSMUST00000107887|gb|AK033889|gb|BB075648|tc|TC1614869 | ENSMUST00000107887 | -2.53 |
| A_55_P2158510 | ref|NM_001039238|ens|ENSMUST00000088594|ens|ENSMUST00000088598|tc|TC1719790 | Dcpp2 | -2.52 |
| A_55_P2054708 | ref|XM_001473132|ref|XM_001476910|gb|AK143362|gb|BB554823 | LOC100044824 | -2.52 |
| A_55_P2101040 | ref|NM_028737|ens|ENSMUST00000013497|gb|AK030125|tc|TC1591832 | 4931406B18Rik | -2.52 |
| A_51_P392055 | ref|NM_146968|ens|ENSMUST00000111540|ens|ENSMUST00000099777|gb|BC120865 | Olfr1242 | -2.52 |
| A_30_P01032978 | Unknown | chr15:77164450-77201325_R | -2.52 |
| A_55_P2007447 | ref|NM_134130|ens|ENSMUST00000002549|ens|ENSMUST00000117726|ens|ENSMUST00000144150 | Abhd3 | -2.52 |
| A_55_P2037386 | ref|NM_029548|ens|ENSMUST00000108420|ens|ENSMUST00000152035|ens|ENSMUST00000108421 | Rph3al | -2.52 |
| A_30_P01021401 | Unknown | chr15:58981936-58982322_F | -2.51 |
| A_66_P100451 | ref|NM_028550|ens|ENSMUST00000031935|ens|ENSMUST00000122181|gb|AK006951 | 1700074P13Rik | -2.51 |
| A_55_P2238132 | gb|AK006966|riken|1700081H04|nap|NAP091839-1 | 1700081H04Rik | -2.51 |
| A_30_P01030821 | Unknown | chr12:12876192-12918398_R | -2.51 |
| A_30_P01028369 | Unknown | chr6:120616386-120674938_F | -2.51 |
| A_55_P1985633 | ens|ENSMUST00000121829|gb|AK153375|gb|AK153549|riken|I830150I18 | ENSMUST00000121829 | -2.51 |
| A_52_P412698 | ens|ENSMUST00000103390|ens|ENSMUST00000103382|gb|AJ131195|tc|NP061588 | ENSMUST00000103390 | -2.51 |
| A_55_P2119683 | ref|XM_985564|ref|XM_979447|gb|AK020613|tc|TC1649328 | 9530057J20Rik | -2.51 |
| A_55_P2008835 | ref|XM_001473293|ref|XM_001472444|ref|XM_001472494 | LOC100041522 | -2.50 |
| A_55_P2165364 | ref|NM_010291|ens|ENSMUST00000046498|gb|AK082917|tc|TC1582580 | Gjb5 | -2.50 |
| A_55_P1987284 | ref|NM_207574|ens|ENSMUST00000077143|ens|ENSMUST00000077164|gb|BC127965 | Olfr1383 | -2.50 |
| A_52_P483409 | ref|NM_008941|ref|NM_178855|ens|ENSMUST00000060402|ens|ENSMUST00000023566 | Tmprss15 | -2.50 |
| A_51_P200203 | ref|NM_007501|ens|ENSMUST00000061571|gb|AK012183|gb|BC042639 | Neurod4 | -2.50 |
| A_55_P2178834 | ens|ENSMUST00000103456|ref|XM_001477982|gb|Z22061|gb|Z22066 | ENSMUST00000103456 | -2.50 |
| A_55_P2130463 | ref|NM_009962|ens|ENSMUST00000037261|gb|AB109092|tc|TC1588254 | Gpr44 | -2.50 |
| A_30_P01028465 | Unknown | chr1:135956616-135957485_R | -2.50 |
| A_55_P2009037 | Unknown | A_55_P2009037 | -2.50 |
| A_55_P2080070 | ens|ENSMUST00000128258|ref|XM_001477593 | ENSMUST00000128258 | -2.50 |
| A_55_P2097967 | ref|XM_001474923|ref|XM_001477929|tc|TC1731287 | Gm11527 | -2.49 |
| A_51_P373619 | ref|NM_010775|ens|ENSMUST00000047095|gb|D11441|gb|BC012245 | Mbl1 | -2.49 |
| A_55_P1954527 | ens|ENSMUST00000085342|tc|TC1617498 | ENSMUST00000085342 | -2.49 |
| A_30_P01019508 | Unknown | chr12:25852471-25852681_F | -2.49 |
| A_52_P374882 | ref|NM_008493|ens|ENSMUST00000069789|gb|U18812|gb|AK142589 | Lep | -2.49 |
| A_55_P1981649 | ref|XM_001475139 | Gm2929 | -2.49 |
| A_55_P2040830 | ens|ENSMUST00000103592|ens|ENSMUST00000103618|ref|XM_001478285|ref|XM_001478458 | ENSMUST00000103592 | -2.49 |
| A_30_P01021362 | Unknown | chr4:115577276-115577566_F | -2.49 |
| A_51_P208922 | ref|NM_011491|gb|AF056244|gb|BC012206|gb|AK010318 | Stc2 | -2.49 |
| A_55_P2049892 | ref|NM_001105182 | Vmn2r69 | -2.49 |
| A_55_P2164075 | ref|XM_001479672|tc|TC1721720 | LOC100048172 | -2.48 |
| A_55_P2122374 | ens|ENSMUST00000100923|ref|XR_035429|ref|XR_035440|gb|AK136664 | ENSMUST00000100923 | -2.48 |
| A_55_P2006436 | ref|NM_146457|ens|ENSMUST00000075851|gb|BC153041|gb|BC146434 | Olfr282 | -2.48 |
| A_55_P2113954 | ref|NM_146276|ens|ENSMUST00000052668|gb|BC127968|tc|NP647028 | Olfr1394 | -2.48 |
| A_51_P252565 | ref|NM_009660|ens|ENSMUST00000019068|gb|CT010263|gb|U04331 | Alox15 | -2.48 |
| A_30_P01018963 | Unknown | chr13:98221098-98480773_R | -2.47 |
| A_55_P2042126 | ref|XM_001473489 | LOC100045220 | -2.47 |
| A_55_P2421352 | gb|AK020298|tc|TC1621681|riken|9130604C24|nap|NAP024010-001 | 9130604C24Rik | -2.47 |
| A_51_P372372 | ref|NM_146305|ens|ENSMUST00000068403|gb|BC145885|tc|NP646999 | Olfr420 | -2.47 |
| A_30_P01029921 | Unknown | chrX:121675099-121675731_F | -2.47 |
| A_55_P2116365 | ref|NM_001099634|ens|ENSMUST00000040369|ens|ENSMUST00000087265|ens|ENSMUST00000041475 | Myof | -2.47 |
| A_51_P481679 | ref|NM_013913|ens|ENSMUST00000030280|gb|AK163595|gb|AK149555 | Angptl3 | -2.47 |
| A_55_P2161380 | ref|NM_015738|ens|ENSMUST00000058004|gb|BC131950|gb|BC131952 | Galr3 | -2.47 |
| A_55_P2178664 | ens|ENSMUST00000104931 | ENSMUST00000104931 | -2.47 |
| A_55_P2176871 | ref|XR_002294|ref|XR_001794 | Gm7760 | -2.47 |
| A_55_P2041733 | ref|NM_001085499|ens|ENSMUST00000114928|gb|AK133392|gb|AV256626 | Gm595 | -2.47 |
| A_30_P01021210 | Unknown | chr5:52461266-52541416_F | -2.47 |
| A_30_P01023711 | Unknown | chr6:86448774-86450531_R | -2.46 |
| A_51_P298615 | ref|NM_010713|ens|ENSMUST00000029852|gb|D49658|gb|AK160121 | Lhx8 | -2.46 |
| A_55_P1974572 | ens|ENSMUST00000099661 | ENSMUST00000099661 | -2.46 |
| A_55_P1954346 | ref|NM_028918|ens|ENSMUST00000006976|gb|AK161541|riken|5131400N01 | Ttc25 | -2.46 |
| A_55_P2090077 | ref|NM_001081250|ens|ENSMUST00000081911|ens|ENSMUST00000108684|gb|AK136634 | Myh13 | -2.46 |
| A_55_P1965030 | ref|NM_001003915|ref|NM_001177624|ens|ENSMUST00000111026|ens|ENSMUST00000045972 | Slc5a12 | -2.46 |
| A_30_P01017549 | Unknown | chr3:95500407-95500904_F | -2.46 |
| A_55_P2413598 | ref|NM_001080813|ref|NM_029423|ens|ENSMUST00000054212|ens|ENSMUST00000033878 | Rab11fip1 | -2.45 |
| A_51_P208152 | ref|NR_030715|ens|ENSMUST00000040608|gb|AK014460|tc|TC1589300 | 3930402G23Rik | -2.45 |
| A_55_P2224116 | gb|AK016028|tc|NP741981|riken|4930543E12|nap|NAP074561-1 | 4930543E12Rik | -2.45 |
| A_30_P01032943 | Unknown | chr1:162965001-162966376_F | -2.45 |
| A_55_P2000978 | ref|NM_176931|ens|ENSMUST00000119693|gb|AB109020|gb|AB109019 | Syt15 | -2.45 |
| A_55_P2058340 | ens|ENSMUST00000038776|gb|AK165551|gb|AK014651|gb|AK029349 | ENSMUST00000038776 | -2.45 |
| A_52_P649210 | ens|ENSMUST00000103677|ens|ENSMUST00000103678|gb|AJ271436|gb|M37280 | ENSMUST00000103677 | -2.45 |
| A_30_P01029973 | Unknown | chrX:98680220-98718445_R | -2.44 |
| A_30_P01023642 | Unknown | chr1:135783286-135783637_R | -2.44 |
| A_51_P249286 | ref|NM_011267|ens|ENSMUST00000027748|gb|AK154324|gb|AK079543 | Rgs16 | -2.44 |
| A_30_P01033394 | Unknown | chr1:92412246-92423471_R | -2.44 |
| A_55_P2000756 | ref|NM_146695|ens|ENSMUST00000077538|tc|TC1600287|nap|NAP022190-001 | Olfr1469 | -2.44 |
| A_55_P1988364 | ref|NM_001013609|ens|ENSMUST00000095375|gb|AK007143|gb|AY552600 | Tex24 | -2.43 |
| A_55_P1961444 | ref|NM_177568|ens|ENSMUST00000077829|ens|ENSMUST00000006415|ens|ENSMUST00000102524 | Plcb2 | -2.43 |
| A_30_P01026875 | Unknown | chr4:8606650-8614031_F | -2.43 |
| A_30_P01022128 | Unknown | chr13:62701350-62715650_F | -2.43 |
| A_55_P2063588 | ref|NM_025394|ens|ENSMUST00000030851 | Tomm7 | -2.43 |
| A_55_P1979978 | ref|XM_001473041|ref|XM_994015|nap|NAP071037-1|nap|NAP061144-1 | Gm8954 | -2.43 |
| A_51_P334174 | ref|NM_145383|ens|ENSMUST00000032471|gb|BC013125|gb|BC094460 | Rho | -2.43 |
| A_55_P2090349 | ref|NM_029280|ens|ENSMUST00000060447|ref|XM_001475732 | Mettl5 | -2.43 |
| A_52_P551844 | ens|ENSMUST00000108880|gb|AK079810|gb|BC096763|tc|TC1703075 | ENSMUST00000108880 | -2.43 |
| A_55_P2108825 | ref|XM_001472067 | LOC100044089 | -2.42 |
| A_52_P538363 | ref|NM_053127|ens|ENSMUST00000056522|gb|AY013781|gb|BC141154 | Pcdhb2 | -2.42 |
| A_55_P2016445 | ref|NM_146693|ens|ENSMUST00000076832|gb|BC128026|tc|TC1666202 | Olfr1462 | -2.42 |
| A_55_P2112097 | ref|XM_001472072|ref|XR_031432 | LOC100039005 | -2.42 |
| A_51_P509573 | ref|NM_013652|ens|ENSMUST00000019074|gb|AF128218|gb|AF128219 | Ccl4 | -2.42 |
| A_30_P01030281 | Unknown | chr11:97276846-97310021_F | -2.42 |
| A_52_P152952 | ref|NM_027203|ens|ENSMUST00000019878|gb|AK005095|gb|AK037634 | Leng1 | -2.42 |
| A_55_P2050526 | ref|XR_033915|ref|XR_033926 | Gm4034 | -2.42 |
| A_52_P646552 | ref|NM_206816|ens|ENSMUST00000080231|gb|BC139087|tc|TC1599729 | Olfr128 | -2.42 |
| A_55_P2183035 | ens|ENSMUST00000106408|ens|ENSMUST00000124281 | ENSMUST00000106408 | -2.41 |
| A_52_P275069 | ref|NM_001177416|ref|NM_001177417|ens|ENSMUST00000121024|ens|ENSMUST00000155248 | Gm6792 | -2.41 |
| A_30_P01025383 | Unknown | chr10:66349796-66378261_R | -2.41 |
| A_55_P2175341 | ens|ENSMUST00000107402|ens|ENSMUST00000066489|gb|AK029271|riken|4832408F05 | ENSMUST00000107402 | -2.41 |
| A_55_P2049313 | ref|XM_001476964|ref|XM_980747|ref|XM_990471|ref|XR_030637 | LOC433064 | -2.41 |
| A_30_P01024733 | Unknown | chr17:17315025-17460175_R | -2.41 |
| A_55_P1980550 | ref|XM_001480465 | LOC100048388 | -2.41 |
| A_30_P01029690 | Unknown | chr16:45016828-45069177_F | -2.41 |
| A_55_P2005394 | ref|XR_032336|ref|XR_002283 | Gm9436 | -2.41 |
| A_30_P01026706 | Unknown | chr12:21548850-21568550_F | -2.41 |
| A_30_P01033281 | Unknown | chr16:84707202-84707748_F | -2.41 |
| A_55_P2099468 | ref|XM_001478414|ref|XM_001478853|gb|CB174020 | Gm4147 | -2.41 |
| A_30_P01028595 | Unknown | chr18:90750100-90772025_F | -2.41 |
| A_30_P01024993 | Unknown | chr1:167218236-167218418_F | -2.41 |
| A_30_P01018741 | Unknown | chr14:79304650-79345152_F | -2.41 |
| A_55_P2030612 | ens|ENSMUST00000079189|ref|XM_001000244|ref|XM_994088|gb|AK015021 | 4922502B01Rik | -2.41 |
| A_55_P2319533 | gb|AK018667|tc|TC1600330|riken|9130410C08|nap|NAP073841-1 | 9130410C08Rik | -2.40 |
| A_55_P1968544 | ref|NR_003619|ens|ENSMUST00000059190|gb|AK032024|gb|AK088233 | 6330549D23Rik | -2.40 |
| A_51_P136303 | ref|NM_134127|ens|ENSMUST00000008801|gb|BC021377|gb|AF233645 | Cyp4f15 | -2.40 |
| A_30_P01018884 | Unknown | chr7:137669440-137672712_F | -2.40 |
| A_30_P01018246 | Unknown | chr1:93812836-93816128_F | -2.39 |
| A_55_P1987675 | ref|XM_001472024|ref|XM_001472350 | Gm2001 | -2.39 |
| A_30_P01024399 | Unknown | chr12:88216358-88221647_R | -2.39 |
| A_66_P129377 | ref|XM_977131|ref|XM_984331|gb|AK087822|tc|TC1618346 | Gm7644 | -2.39 |
| A_55_P2052131 | ref|XM_001472361|nap|NAP059579-1 | LOC674190 | -2.39 |
| A_51_P128657 | ens|ENSMUST00000060220|gb|AK015957|tc|TC1594559|riken|4930533L02 | ENSMUST00000060220 | -2.39 |
| A_30_P01021350 | Unknown | chr13:65338067-65365992_F | -2.39 |
| A_55_P2088380 | ref|XM_001003864|ref|XM_916741 | C230072F16Rik | -2.39 |
| A_30_P01018549 | Unknown | chr15:62164419-62165018_F | -2.39 |
| A_55_P2176670 | ref|XM_141989|ref|XR_033707|nap|NAP061168-1 | Pabpc1l2a-ps | -2.38 |
| A_55_P1976814 | ref|XM_001474628|ref|XM_001476102|tc|TC1679329 | Gm2753 | -2.38 |
| A_55_P2182187 | ref|NM_009719|ens|ENSMUST00000050103|gb|AK008017|tc|TC1592863 | Neurog3 | -2.38 |
| A_55_P2184889 | ref|XM_001476948 | LOC100046866 | -2.38 |
| A_55_P2102260 | ref|NM_008217|ens|ENSMUST00000034385|ref|XM_001479247|ref|XM_001479255 | Has3 | -2.38 |
| A_52_P669922 | ref|NM_032541|ens|ENSMUST00000062620|gb|AF297664|gb|BC021587 | Hamp | -2.38 |
| A_30_P01021990 | Unknown | chr2:145492995-145523670_F | -2.38 |
| A_52_P489999 | ens|ENSMUST00000084243|gb|AY512921|tc|TC1641831|nap|NAP125213-1 | ENSMUST00000084243 | -2.38 |
| A_66_P135601 | ref|XM_001473655|ref|XM_001477485 | Gm8420 | -2.38 |
| A_30_P01021552 | Unknown | chr2:129416848-129417413_R | -2.38 |
| A_30_P01020921 | Unknown | chr9:61071844-61072324_F | -2.38 |
| A_55_P2239928 | gb|CD766363 | 1810013D15Rik | -2.38 |
| A_55_P1952240 | ref|NM_001161431|ref|NM_198863|ens|ENSMUST00000036043|gb|BC111888 | Slitrk2 | -2.37 |
| A_30_P01032540 | Unknown | chr13:19617358-19617879_F | -2.37 |
| A_55_P2123557 | ref|XR_035737 | EG640142 | -2.37 |
| A_55_P2100355 | ref|NM_011326|ens|ENSMUST00000000221|gb|AF112187|gb|AK143995 | Scnn1g | -2.37 |
| A_55_P1995512 | ref|NM_175408|ens|ENSMUST00000095987|gb|AK053212|gb|BC096053 | Tmem139 | -2.37 |
| A_30_P01032389 | Unknown | chr13:97697927-97698400_F | -2.37 |
| A_55_P2130870 | ref|XM_893095 | Gm6871 | -2.37 |
| A_55_P2067131 | ref|NM_175683|ref|NM_001110214|ens|ENSMUST00000061852|gb|BC030680 | Dclre1c | -2.37 |
| A_52_P569549 | ref|NM_176953|gb|AK048442|gb|CJ199109|riken|C130061B15 | Lig4 | -2.37 |
| A_55_P2073284 | ref|NM_001105066|ref|NM_001105075|ref|NM_009489|ens|ENSMUST00000072787 | Vmn2r34 | -2.36 |
| A_52_P523712 | ref|NM_011537|ens|ENSMUST00000018407|gb|AK044843|gb|BC090639 | Tbx5 | -2.36 |
| A_55_P2122945 | ref|NM_146962|ens|ENSMUST00000080681|gb|BC119150|gb|BC120836 | Olfr541 | -2.36 |
| A_30_P01020768 | Unknown | chr14:99729955-99730583_F | -2.36 |
| A_51_P311958 | ref|NM_013623|ens|ENSMUST00000006687|gb|BC145560|gb|BC141235 | Orm3 | -2.36 |
| A_52_P594756 | ref|NM_023048|ens|ENSMUST00000043294|gb|AK133894|gb|BC046819 | Asb4 | -2.36 |
| A_52_P2710 | ref|NM_023493|ens|ENSMUST00000032074|gb|AK007530|gb|AF187099 | Cml5 | -2.36 |
| A_52_P149577 | ref|NM_016899|ens|ENSMUST00000131775|ens|ENSMUST00000008745|gb|AB232620 | Rab25 | -2.36 |
| A_55_P2035407 | ref|NM_001105201|ens|ENSMUST00000073391|ref|XM_914939|gb|BC151098 | Cyp26c1 | -2.36 |
| A_66_P107068 | ref|XM_357735|nap|NAP029108-1 | Gm5330 | -2.35 |
| A_55_P1952434 | ref|NM_053157|ens|ENSMUST00000107052|ens|ENSMUST00000102793|ens|ENSMUST00000030292 | Tm2d1 | -2.35 |
| A_55_P2040316 | ref|XM_001473027 | Gm2280 | -2.35 |
| A_52_P151240 | ref|XM_973835|ref|XM_975315|gb|AK087278|tc|TC1623461 | Fam150a | -2.35 |
| A_55_P2174761 | ref|NM_146726|ens|ENSMUST00000084754|gb|BC148431|gb|BC153013 | Olfr514 | -2.35 |
| A_66_P107348 | ref|NM_207141|ens|ENSMUST00000073946|gb|BC146333|gb|BC148817 | Olfr955 | -2.35 |
| A_30_P01018192 | Unknown | chr15:41486232-41499767_R | -2.34 |
| A_55_P2143436 | ref|XR_032105|ref|XR_033879 | Gm7765 | -2.34 |
| A_30_P01028250 | Unknown | chr3:75451370-75459674_R | -2.34 |
| A_52_P144263 | ref|NM_020296|ref|NM_001141931|ref|NM_001141932|ens|ENSMUST00000074547 | Rbms1 | -2.34 |
| A_66_P111191 | ens|ENSMUST00000103555|gb|Z48592|gb|AK133996|tc|TC1675302 | ENSMUST00000103555 | -2.34 |
| A_30_P01026662 | Unknown | chr18:82934418-82935002_F | -2.34 |
| A_51_P422360 | ref|NM_139270|ens|ENSMUST00000027083|gb|AK045576|gb|AK083278 | Pth2r | -2.34 |
| A_30_P01029319 | Unknown | chr17:14683411-14698476_F | -2.33 |
| A_30_P01022741 | Unknown | chr9:20313342-20323517_R | -2.33 |
| A_55_P2360077 | gb|AK042842|gb|BY655930|riken|A730029P14|nap|NAP082174-1 | 2610035F20Rik | -2.33 |
| A_51_P223902 | ens|ENSMUST00000063585|gb|AK031916|gb|AV328766|riken|6330444E15 | ENSMUST00000063585 | -2.33 |
| A_55_P1968015 | ref|XM_001480598 | LOC100048460 | -2.33 |
| A_51_P313397 | ref|XM_001477748|gb|AK007220|gb|AK018972|gb|BY707324 | 1700121C10Rik | -2.33 |
| A_55_P2327359 | gb|AK082282|riken|C230032F09|nap|NAP084606-1 | LOC433611 | -2.33 |
| A_30_P01029347 | Unknown | chr8:41934312-41945487_F | -2.32 |
| A_55_P1985935 | ref|XM_001477673|ref|XM_001478208|gb|AK035625|tc|TC1612231 | 9130015G15Rik | -2.32 |
| A_55_P1952658 | ref|XM_001477892|gb|AK135955|riken|7420441G07 | Gm3772 | -2.32 |
| A_55_P1994739 | ens|ENSMUST00000112542|ens|ENSMUST00000068500|gb|AK028243|gb|BY712647 | ENSMUST00000112542 | -2.32 |
| A_55_P2207335 | gb|AK042215|tc|TC1629789|riken|A630071L07|nap|NAP081918-1 | A630071L07Rik | -2.32 |
| A_55_P2070224 | ref|XM_001476170 | LOC100041330 | -2.32 |
| A_55_P1997421 | ref|NM_001082542|ens|ENSMUST00000089628|gb|BC115936|gb|BC146444 | Gm5416 | -2.32 |
| A_52_P236705 | ref|NM_133229|ens|ENSMUST00000023660|gb|AK041355|gb|AK172430 | Ripply3 | -2.32 |
| A_51_P435671 | ref|XM_904758|gb|X96776|gb|Z26768|gb|Z26769 | LOC631064 | -2.32 |
| A_51_P390954 | ref|NR_033509|ens|ENSMUST00000068397|gb|AK080168|gb|BB637412 | Gm9961 | -2.32 |
| A_52_P279110 | ens|ENSMUST00000046683|gb|AK018637|tc|TC1594146|riken|9130019P16 | ENSMUST00000046683 | -2.32 |
| A_55_P1972858 | ref|XM_619521|tc|TC1723607 | Gm5819 | -2.31 |
| A_30_P01029196 | Unknown | chr7:50545068-50545454_R | -2.31 |
| A_51_P480241 | ref|NM_007921|ref|NM_001163131|ens|ENSMUST00000003135|gb|BC145380 | Elf3 | -2.31 |
| A_30_P01020753 | Unknown | chrX:11684504-11685304_F | -2.31 |
| A_52_P533265 | ref|NM_177711|gb|AK077018|tc|TC1589904|riken|4932411G14 | 4932411G14Rik | -2.31 |
| A_30_P01027476 | Unknown | chr12:20240893-20435318_R | -2.31 |
| A_55_P1971518 | ref|XM_001478508|ref|XM_486235 | Gm3648 | -2.31 |
| A_30_P01029470 | Unknown | chr6:3333825-3349525_R | -2.31 |
| A_55_P2031397 | ens|ENSMUST00000101255|gb|AK136626|tc|NP1484759|riken|9230009I02 | ENSMUST00000101255 | -2.30 |
| A_55_P2156121 | ens|ENSMUST00000075018|tc|TC1639303|nap|NAP121129-001|nap|NAP029825-1 | ENSMUST00000075018 | -2.30 |
| A_51_P277431 | ref|NM_028804|ens|ENSMUST00000027988|gb|AK172247|gb|AK009833 | Ccdc3 | -2.30 |
| A_30_P01019400 | Unknown | chr16:94775095-94775634_F | -2.30 |
| A_30_P01020528 | Unknown | chr9:121093500-121104550_F | -2.30 |
| A_55_P2044498 | ref|NM_153106|ens|ENSMUST00000038749|ens|ENSMUST00000105796|gb|AF529423 | Padi6 | -2.30 |
| A_66_P130541 | ref|NM_011634|ens|ENSMUST00000049348|ref|XR_031822|ref|XR_031887 | Traip | -2.29 |
| A_52_P117408 | ref|NM_009375|ens|ENSMUST00000065916|gb|AF076186|gb|AF076187 | Tg | -2.29 |
| A_55_P2068121 | ref|NM_029599|ens|ENSMUST00000028982|ref|XR_031396|gb|AY307077 | Sun5 | -2.29 |
| A_30_P01031708 | Unknown | chr12:12768528-12849976_R | -2.29 |
| A_55_P2314316 | gb|CA491940 | 0610008F07Rik | -2.29 |
| A_55_P2172515 | ens|ENSMUST00000029642|ref|XM_485306|gb|AK006854|tc|TC1593035 | 1700061I17Rik | -2.29 |
| A_55_P2171563 | ref|NM_011746|ens|ENSMUST00000094340|gb|AK134870|gb|BC054771 | Mkrn3 | -2.29 |
| A_55_P2081116 | ref|NM_001081120|ens|ENSMUST00000055257|ref|XM_001006406|ref|XM_001478908 | Fam89a | -2.29 |
| A_55_P2071349 | ref|NM_009426|ens|ENSMUST00000006046|gb|AK010666|gb|BC053493 | Trh | -2.29 |
| A_30_P01026793 | Unknown | chr6:39267707-39268180_R | -2.28 |
| A_55_P2054703 | ref|XM_001474258|nap|NAP061306-1|nap|NAP060700-1|nap|NAP062657-1 | Gm2659 | -2.28 |
| A_30_P01020290 | Unknown | chr9:24617562-24625262_F | -2.28 |
| A_55_P2078138 | ref|NM_172709|ens|ENSMUST00000114099|ens|ENSMUST00000063136|gb|BC146285 | Otop1 | -2.28 |
| A_55_P2097230 | ens|ENSMUST00000120586|ens|ENSMUST00000142316|gb|BC026933|tc|TC1656102 | ENSMUST00000120586 | -2.28 |
| A_66_P108918 | ref|NM_001011836|ref|NM_146838|ens|ENSMUST00000081034|ens|ENSMUST00000079711 | Olfr1129 | -2.28 |
| A_55_P2026487 | ens|ENSMUST00000099390|gb|AK164675|riken|D530038N07 | LOC100046701 | -2.28 |
| A_30_P01024296 | Unknown | chr9:7214753-7217212_F | -2.28 |
| A_55_P2077831 | ens|ENSMUST00000057543|ref|XR_035313|ref|XR_035377|gb|AK042646 | ENSMUST00000057543 | -2.27 |
| A_55_P1969890 | ref|NM_146659|ens|ENSMUST00000099852|gb|BC120620|gb|BC120618 | Olfr1136 | -2.27 |
| A_55_P2036693 | ref|NM_011611|ref|NM_170704|ref|NM_170703|ref|NM_170702 | Cd40 | -2.27 |
| A_55_P2033393 | ref|NM_172577|ref|NM_001167976|ens|ENSMUST00000110680|ens|ENSMUST00000044634 | Slc25a21 | -2.27 |
| A_52_P538412 | ref|NM_147081|ens|ENSMUST00000063109|gb|BC113199|tc|TC1600026 | Olfr610 | -2.27 |
| A_55_P1954680 | ref|NR_033532|ref|XM_001471874|gb|AK038776|gb|AK080797 | B230206H07Rik | -2.27 |
| A_30_P01023776 | Unknown | chr4:56552801-56553404_R | -2.27 |
| A_55_P2032192 | ref|NM_001164249|ref|NM_001164250|ref|NM_024427|ref|NM_001164252 | Tpm1 | -2.27 |
| A_52_P212025 | ens|ENSMUST00000039630|gb|AK012979|riken|2810404D04|nap|NAP002524-001 | Ror1 | -2.27 |
| A_51_P303749 | ref|NM_178683|ens|ENSMUST00000051594|gb|AK077676|gb|AK036550 | Depdc1b | -2.26 |
| A_51_P341336 | ref|NM_025778|ens|ENSMUST00000032321|gb|AK016670|tc|TC1585007 | Bcl2l14 | -2.26 |
| A_51_P241769 | ref|NM_011270|ens|ENSMUST00000030627|gb|AK079335|gb|AK171641 | Rhd | -2.26 |
| A_55_P2067391 | ref|XM_891766|ref|XM_908883 | Gm6732 | -2.26 |
| A_30_P01028270 | Unknown | chr15:3946037-3972747_F | -2.26 |
| A_51_P370717 | ref|NM_010351|ens|ENSMUST00000021513|gb|Y13149|gb|Y13150 | Gsc | -2.26 |
| A_51_P123625 | ref|NM_008392|ens|ENSMUST00000022722|gb|AK152177|gb|L38281 | Irg1 | -2.26 |
| A_30_P01023794 | Unknown | chr5:113771346-113771431_F | -2.26 |
| A_55_P2082356 | ref|NM_146317|ens|ENSMUST00000089844|gb|BC125411|gb|BC125417 | Olfr725 | -2.26 |
| A_51_P123475 | ref|NM_146461|ens|ENSMUST00000099809|gb|BC104269|gb|BC104270 | Olfr1209 | -2.26 |
| A_51_P362176 | ref|NM_133862|ens|ENSMUST00000048486|gb|AK013887|gb|AK149448 | Fgg | -2.25 |
| A_52_P312371 | ref|NM_001034900|ref|NM_001145863|ens|ENSMUST00000109929|ens|ENSMUST00000109926 | Zfp345 | -2.25 |
| A_55_P1957148 | ref|XM_925521|tc|TC1719863 | 6430503K07Rik | -2.25 |
| A_51_P452153 | ref|NM_027222|ens|ENSMUST00000025211|gb|AK088094|gb|AK008016 | 2010001M09Rik | -2.25 |
| A_55_P2117818 | ens|ENSMUST00000099167|gb|AK162913|riken|A330029E17 | ENSMUST00000099167 | -2.25 |
| A_55_P2063283 | ens|ENSMUST00000103418|ens|ENSMUST00000103417|gb|GQ984290|gb|EF392837 | ENSMUST00000103418 | -2.25 |
| A_30_P01033342 | Unknown | chr3:121997064-122031218_F | -2.25 |
| A_51_P131335 | ref|NM_153568|ens|ENSMUST00000087177|gb|AK051875|gb|BC031901 | Lrrc66 | -2.25 |
| A_51_P483639 | ref|NM_175485|ens|ENSMUST00000055535|gb|AK168030|gb|AK039115 | Prtg | -2.25 |
| A_55_P2133978 | ref|NM_001009950|ens|ENSMUST00000036748|gb|BC089013|tc|TC1588540 | Slc38a8 | -2.25 |
| A_55_P2093634 | ref|NM_001017966|ref|NM_023544|ens|ENSMUST00000105782|ens|ENSMUST00000038278 | Ddi2 | -2.25 |
| A_55_P2162622 | ens|ENSMUST00000103685|ref|XM_903846|ref|XM_920890|ref|XM_900951 | ENSMUST00000103685 | -2.25 |
| A_55_P2150349 | ref|XM_001474860|ref|XM_001475612|tc|TC1661165 | LOC100045743 | -2.24 |
| A_55_P2105045 | ref|XM_901831|ref|XM_921526|gb|AK141411|tc|TC1691243 | Gm11458 | -2.24 |
| A_52_P1004509 | ref|NR_033324|ens|ENSMUST00000089350|gb|AK084423|gb|AK160692 | BC065397 | -2.24 |
| A_55_P1998641 | ref|NR_002870|ens|ENSMUST00000104989|gb|AY751519|gb|AB159607 | Dnm3os | -2.24 |
| A_30_P01026702 | Unknown | chr1:161046625-161069450_R | -2.24 |
| A_66_P130916 | ref|NM_010389|ens|ENSMUST00000069091|ens|ENSMUST00000095342|gb|BC100709 | H2-Ob | -2.24 |
| A_55_P2151360 | ref|XR_033652|ref|XR_034674|nap|NAP112021-1 | Gm5496 | -2.23 |
| A_30_P01019690 | Unknown | chr7:140017838-140017923_F | -2.23 |
| A_30_P01022176 | Unknown | chr4:63810346-63810958_F | -2.23 |
| A_55_P2132723 | ref|XM_001471978 | LOC100038957 | -2.23 |
| A_55_P2020841 | ref|XM_001477845|ref|XM_001478295 | Gm9476 | -2.23 |
| A_55_P2205459 | ref|NM_009330|ens|ENSMUST00000021016|ens|ENSMUST00000108113|ens|ENSMUST00000108114 | Hnf1b | -2.23 |
| A_55_P2242124 | gb|BG079244|tc|TC1660859|nap|NAP036689-1 | D15Ertd50e | -2.22 |
| A_55_P2031026 | ref|NM_147032|ens|ENSMUST00000106764|ens|ENSMUST00000060879|gb|BC111577 | Olfr705 | -2.22 |
| A_30_P01030573 | Unknown | chr12:76763402-76763857_R | -2.22 |
| A_30_P01018909 | Unknown | chr10:98761521-98885681_F | -2.22 |
| A_55_P2143070 | ref|NM_007494|ens|ENSMUST00000102840|gb|M31690|gb|AK149489 | Ass1 | -2.22 |
| A_55_P2267240 | gb|BU938417 | 4930471G03Rik | -2.22 |
| A_55_P1965861 | ens|ENSMUST00000061201|ref|XM_912945|ref|XM_001474122|ref|XM_912283 | ENSMUST00000061201 | -2.22 |
| A_51_P331098 | ens|ENSMUST00000026083|gb|AK015359|tc|TC1594081|riken|4930442E04 | ENSMUST00000026083 | -2.22 |
| A_51_P286357 | ref|NM_139138|ens|ENSMUST00000025004|gb|AK037483|gb|AK036459 | Emr4 | -2.22 |
| A_52_P235880 | ref|NM_198635|ens|ENSMUST00000099577|gb|AK133270|riken|4932428C01 | Gm5134 | -2.22 |
| A_51_P199168 | ref|NM_007702|ens|ENSMUST00000025404|gb|AF041376|gb|BC096649 | Cidea | -2.21 |
| A_55_P1995278 | ref|XM_001474138 | LOC100045361 | -2.21 |
| A_55_P2155814 | ref|XM_001480336|nap|NAP030414-1 | LOC100048485 | -2.21 |
| A_55_P2049494 | ref|XM_001480313 | LOC100048469 | -2.21 |
| A_52_P549348 | ref|NM_198601|ens|ENSMUST00000022708|gb|AK029521|tc|TC1592501 | Trim52 | -2.21 |
| A_52_P493477 | ref|NM_173051|ens|ENSMUST00000021834|gb|BC104332|tc|TC1589741 | Serpinb1c | -2.21 |
| A_51_P239984 | ref|NM_012012|ens|ENSMUST00000039725|gb|AK166425|gb|AJ238213 | Exo1 | -2.21 |
| A_52_P272145 | ref|XM_001476021|nap|NAP026501-1|nap|NAP070944-1|nap|NAP060384-1 | LOC677414 | -2.20 |
| A_51_P354126 | ref|NM_011260|ens|ENSMUST00000032089|gb|AK008446|gb|AK008608 | Reg3g | -2.20 |
| A_30_P01025070 | Unknown | chr12:88216358-88221647_F | -2.20 |
| A_30_P01021899 | Unknown | chr8:122788975-122789224_R | -2.20 |
| A_55_P1976882 | ref|XM_001473718|ref|XM_001479086 | Gm2475 | -2.20 |
| A_55_P2090357 | ref|XM_001472591 | LOC100044107 | -2.20 |
| A_55_P2092993 | ref|NM_175326|ens|ENSMUST00000046763|ens|ENSMUST00000113030|gb|AK155588 | D330045A20Rik | -2.20 |
| A_51_P194503 | ref|NM_146717|ens|ENSMUST00000052975|gb|BC119238|gb|AK154347 | Olfr433 | -2.20 |
| A_55_P2210724 | ref|NM_001014398|ens|ENSMUST00000093837|ens|ENSMUST00000093836|gb|AY701871 | Trcg1 | -2.19 |
| A_52_P203770 | ref|NM_001105058|nap|NAP113605-1|nap|NAP102873-1 | Vmn2r61 | -2.19 |
| A_30_P01022473 | Unknown | chr8:108120629-108123201_F | -2.19 |
| A_52_P650108 | ref|NM_011274|ens|ENSMUST00000085513|gb|AK165045|gb|BC038523 | C80913 | -2.19 |
| A_55_P2163744 | ref|NM_020604|ens|ENSMUST00000038382|gb|AB024445|gb|BC120839 | Jph1 | -2.19 |
| A_55_P2204676 | gb|BG069650 | AU020147 | -2.19 |
| A_55_P2079630 | ref|NM_009873|ens|ENSMUST00000042410|gb|AF132483|gb|AK154197 | Cdk6 | -2.19 |
| A_51_P104569 | ref|NM_146505|ens|ENSMUST00000050807|gb|BC125353|gb|BC132239 | Olfr148 | -2.19 |
| A_55_P1991674 | ens|ENSMUST00000092790 | ENSMUST00000092790 | -2.19 |
| A_30_P01025842 | Unknown | chr10:84005221-84022896_F | -2.19 |
| A_55_P2158494 | ref|XM_913775 | LOC637948 | -2.19 |
| A_55_P2127971 | ref|NM_001177365|ens|ENSMUST00000113610|ens|ENSMUST00000113602|ref|XM_001001147 | Gm9112 | -2.19 |
| A_30_P01026743 | Unknown | chr18:75513062-75523889_F | -2.18 |
| A_51_P448178 | ref|NM_175519|ens|ENSMUST00000054095|gb|AK047519|gb|AK043351 | Kctd8 | -2.18 |
| A_52_P579517 | ref|NM_178379|ens|ENSMUST00000049091|gb|AK156865|gb|AK172718 | Cox10 | -2.18 |
| A_30_P01018392 | Unknown | chr10:81589292-81626369_R | -2.18 |
| A_30_P01020274 | Unknown | chr8:59994074-60002058_R | -2.18 |
| A_55_P2056846 | ref|NM_146956|ens|ENSMUST00000078103|gb|BC139241|gb|BC139246 | Olfr525 | -2.18 |
| A_30_P01020889 | Unknown | chrX:123303474-123305531_F | -2.18 |
| A_55_P2304482 | gb|AK053996|tc|TC1611548|riken|E230011F24|nap|NAP088140-1 | 2700086A05Rik | -2.18 |
| A_30_P01026287 | Unknown | chr8:49911596-49912196_R | -2.17 |
| A_51_P107722 | ens|ENSMUST00000061643|gb|AK011970|tc|TC1648870|riken|2610303G11 | ENSMUST00000061643 | -2.17 |
| A_55_P2036673 | ref|NM_030096|ens|ENSMUST00000049257|gb|BC029094|gb|AK141200 | Ddx52 | -2.17 |
| A_55_P2061448 | ref|XM_001480247|ref|XM_001480156 | LOC100048579 | -2.17 |
| A_55_P2120931 | ref|NR_028264|gb|AK042416|gb|AK084358|gb|AF380423 | Dleu2 | -2.17 |
| A_30_P01020582 | Unknown | chr13:114514159-114514765_R | -2.17 |
| A_52_P68883 | ref|NM_183026|ens|ENSMUST00000052601|gb|BC116198|gb|AJ578468 | Defb14 | -2.17 |
| A_55_P2285461 | gb|BG069502 | AU019542 | -2.17 |
| A_30_P01027450 | Unknown | chr15:85534202-85537090_F | -2.17 |
| A_66_P128342 | ref|XM_001472313|ref|XM_001474964|gb|AJ311366|tc|TC1695364 | Gm8635 | -2.16 |
| A_55_P2018865 | ref|NM_001005418|ref|NM_007847|ref|XM_001471604|gb|AJ564861 | Defa-rs4 | -2.16 |
| A_30_P01028566 | Unknown | chr11:120041124-120052299_F | -2.16 |
| A_66_P112305 | ref|NM_053214|ens|ENSMUST00000087605|gb|AK153908|gb|AK131188 | Myo1f | -2.16 |
| A_66_P126153 | ens|ENSMUST00000113602|ref|XM_001001147|ref|XM_001474336|gb|AK005877 | 1700011M02Rik | -2.16 |
| A_55_P2161530 | ref|NM_172882|ens|ENSMUST00000104987|gb|BC014824|gb|BC049866 | Wdfy3 | -2.16 |
| A_55_P1953723 | ens|ENSMUST00000107373|gb|AK080805|tc|TC1608067|riken|B230212B15 | ENSMUST00000107373 | -2.16 |
| A_55_P2343929 | gb|AK015944|tc|NP742032|riken|4930532I03|nap|NAP074543-1 | 4930532I03Rik | -2.16 |
| A_55_P1961628 | ref|NM_013776|ref|NM_013773|ens|ENSMUST00000105217|ens|ENSMUST00000000717 | Tcl1b5 | -2.16 |
| A_30_P01021483 | Unknown | chr6:57560819-57569928_F | -2.16 |
| A_55_P2162060 | ens|ENSMUST00000063789 | ENSMUST00000063789 | -2.16 |
| A_66_P111352 | ref|NM_013833|ens|ENSMUST00000025396|gb|AK133951|gb|BC024731 | Rax | -2.16 |
| A_55_P1968608 | ref|NM_146341|ens|ENSMUST00000090695|ens|ENSMUST00000111514|nap|NAP110761-1 | Olfr1259 | -2.16 |
| A_52_P404302 | ref|NM_172854|gb|AK220202|gb|BC137871|gb|AK035313 | Olfml2a | -2.16 |
| A_55_P2116059 | ref|NM_172393|ens|ENSMUST00000020017|ens|ENSMUST00000099866|gb|AK088102 | Aim1 | -2.16 |
| A_52_P546676 | ref|NM_173861|ens|ENSMUST00000089279|gb|BC137818|gb|AF463503 | Csnka2ip | -2.15 |
| A_55_P1955871 | ref|NM_175290|ens|ENSMUST00000081507|ens|ENSMUST00000037372|gb|AK049352 | Nlrp4f | -2.15 |
| A_55_P1987326 | ref|NM_025896|ens|ENSMUST00000049463|gb|AK005474|gb|BC100334 | Prl3a1 | -2.15 |
| A_52_P534810 | ref|NM_001013750|ens|ENSMUST00000059937|gb|BC150996|gb|AK076972 | Gm597 | -2.15 |
| A_30_P01029369 | Unknown | chr7:129299957-129300406_F | -2.15 |
| A_55_P1963920 | ref|NM_001045526|ens|ENSMUST00000108534|ens|ENSMUST00000074572|gb|AK138818 | A430084P05Rik | -2.15 |
| A_55_P2402312 | gb|AK141776|riken|D030062O11 | D030062O11Rik | -2.15 |
| A_30_P01028603 | Unknown | chrX:6982891-7008528_R | -2.15 |
| A_30_P01018976 | Unknown | chr15:92197603-92208534_F | -2.14 |
| A_55_P2174143 | ref|NM_009265|ens|ENSMUST00000062160|gb|AK003398|tc|TC1585817 | Sprr1b | -2.14 |
| A_30_P01024000 | Unknown | chr5:125078875-125098525_R | -2.14 |
| A_52_P1093529 | ref|NM_177320|ens|ENSMUST00000021283|gb|AK149847|gb|AK154643 | Pik3r5 | -2.14 |
| A_52_P618774 | ref|NM_021429|ens|ENSMUST00000020927|gb|BC141416|gb|BC148679 | Hs1bp3 | -2.14 |
| A_66_P132787 | ens|ENSMUST00000103277|gb|M11859|gb|D12896|gb|X04047 | ENSMUST00000103277 | -2.14 |
| A_30_P01025449 | Unknown | chr15:101088974-101089576_R | -2.14 |
| A_51_P278266 | ref|NM_146020|ens|ENSMUST00000057685|gb|AK050107|gb|AK050212 | Gltpd2 | -2.14 |
| A_55_P1954748 | ref|XM_001478924 | Gm4062 | -2.14 |
| A_55_P2138465 | ref|NM_001011530|ens|ENSMUST00000073741|gb|BC150980|gb|BC120628 | Olfr723 | -2.14 |
| A_55_P2151373 | ref|XM_905487 | Rpl19-ps4 | -2.14 |
| A_55_P1954698 | ref|XM_001475897|ref|XM_001474722|gb|AK158295|gb|BY596749 | LOC100046261 | -2.14 |
| A_30_P01030041 | Unknown | chr13:23487906-23488198_F | -2.14 |
| A_55_P2081990 | ref|NM_029401|ens|ENSMUST00000105812|gb|AK016425|gb|BC144974 | Vwa5b1 | -2.14 |
| A_30_P01032651 | Unknown | chr9:61077284-61077751_R | -2.13 |
| A_30_P01020418 | Unknown | chr3:3030146-3033623_F | -2.13 |
| A_55_P2089035 | ens|ENSMUST00000112482|gb|AK165879|tc|TC1677989|riken|G730012N14 | ENSMUST00000112482 | -2.13 |
| A_55_P1968325 | ref|NM_178630|ens|ENSMUST00000115016|ens|ENSMUST00000115017|gb|AK029793 | Agbl3 | -2.13 |
| A_30_P01025973 | Unknown | chr1:77819988-77820546_F | -2.13 |
| A_66_P113116 | ref|NM_001167581|ens|ENSMUST00000071847|gb|AK006248|tc|TC1590356 | Gsdmcl1 | -2.13 |
| A_52_P475450 | ref|NM_172991|ens|ENSMUST00000121877|ens|ENSMUST00000042753|gb|AK041876 | C030048B08Rik | -2.13 |
| A_55_P2162718 | ref|NM_146473|ens|ENSMUST00000073824|gb|BC146338|gb|BC148821 | Olfr1387 | -2.13 |
| A_55_P2009483 | ens|ENSMUST00000115920|ens|ENSMUST00000099229|ref|XM_979799|ref|XM_001475853 | ENSMUST00000115920 | -2.13 |
| A_30_P01019865 | Unknown | chr17:87483814-87493039_R | -2.13 |
| A_51_P372393 | ref|NM_009641|ens|ENSMUST00000028955|gb|AF113707|gb|BC129965 | Angpt4 | -2.12 |
| A_55_P2113853 | ens|ENSMUST00000075769 | ENSMUST00000075769 | -2.12 |
| A_52_P6524 | ref|NM_001099277|ens|ENSMUST00000108509|gb|BC157962|gb|DQ864732 | Zfp541 | -2.12 |
| A_52_P372032 | ref|NM_019553|gb|BC043655|gb|BC059237|gb|BC060220 | Ddx21 | -2.12 |
| A_30_P01026099 | Unknown | chr5:31856417-31916414_R | -2.12 |
| A_55_P2180201 | ref|NM_026154|ens|ENSMUST00000054252|ens|ENSMUST00000001485|gb|AK007643 | Mrpl10 | -2.12 |
| A_51_P270904 | ref|NM_172641|ref|NM_001164263|ens|ENSMUST00000039666|gb|AK053921 | 9930023K05Rik | -2.12 |
| A_55_P2302750 | gb|C79829 | C80120 | -2.12 |
| A_55_P2366323 | ref|XR_033662|gb|BQ923649|tc|TC1700402 | Gm8234 | -2.12 |
| A_55_P2084283 | ref|XM_149934|ref|XM_923359|tc|TC1696689|nap|NAP063568-1 | Gm581 | -2.12 |
| A_55_P2087147 | ref|XM_983220 | Dnhd1 | -2.12 |
| A_55_P1996911 | ens|ENSMUST00000117551|ens|ENSMUST00000033833|gb|AK053353|gb|AK147896 | ENSMUST00000117551 | -2.11 |
| A_55_P2159760 | ref|NM_146937|ens|ENSMUST00000067840|gb|BC127997|tc|TC1599793 | Olfr63 | -2.11 |
| A_51_P284716 | ref|NM_001166475|ens|ENSMUST00000100396|gb|AK132940|gb|AK029593 | 4930407I10Rik | -2.11 |
| A_55_P2105165 | ens|ENSMUST00000120672|gb|AK013216|tc|TC1698041|riken|2810431L23 | ENSMUST00000120672 | -2.11 |
| A_55_P2361138 | gb|AI451250 | AI451250 | -2.11 |
| A_55_P2353833 | gb|AI481207 | AI481207 | -2.11 |
| A_30_P01031002 | Unknown | chr13:49488852-49489322_F | -2.11 |
| A_55_P2060642 | ref|NM_146068|ens|ENSMUST00000040248|ens|ENSMUST00000115757|gb|AK170784 | 2310008H04Rik | -2.11 |
| A_55_P2035003 | gb|AK163054|riken|A430106F04 | AK163054 | -2.11 |
| A_66_P119550 | ens|ENSMUST00000045546|ref|XM_001476076|ref|XM_978766|tc|NP818202 | Olfr607 | -2.11 |
| A_55_P2187899 | gb|AK015708|riken|4930505N22|nap|NAP122568-001 | 4930505N22Rik | -2.11 |
| A_51_P110640 | ref|NM_010640|ens|ENSMUST00000007156|gb|BC013660|tc|TC1583970 | Klk1b11 | -2.11 |
| A_51_P184306 | ref|NM_026351|ens|ENSMUST00000053168|ens|ENSMUST00000134652|gb|AK016184 | Ttc39d | -2.11 |
| A_55_P2322981 | gb|AK144254|riken|G630001L02 | 2700078F05Rik | -2.11 |
| A_55_P1964098 | ref|XM_001479536|gb|AJ833571|gb|AB070545|tc|NP1397724 | Gm4242 | -2.11 |
| A_51_P262670 | ref|NM_019547|ens|ENSMUST00000029014|gb|AK052886|gb|AK146026 | Rbm38 | -2.10 |
| A_51_P273556 | ref|NM_027975|ens|ENSMUST00000029183|gb|AK159308|gb|BC064129 | Fam83d | -2.10 |
| A_55_P1996451 | ref|XR_002006|ref|XR_033109 | Gm7162 | -2.10 |
| A_55_P2047912 | ref|NM_010015|ens|ENSMUST00000128231|gb|AK030925|riken|5830460N15 | Dad1 | -2.10 |
| A_55_P2230154 | gb|AK039668|riken|A330081K12 | A730046J19Rik | -2.10 |
| A_55_P2128556 | ens|ENSMUST00000106832|gb|AK046571|tc|TC1590646|riken|B430104F01 | ENSMUST00000106832 | -2.10 |
| A_55_P2150717 | ref|NM_010136|ref|NM_001164789|ens|ENSMUST00000111763|ens|ENSMUST00000035020 | Eomes | -2.10 |
| A_55_P2092391 | ref|NM_028836|ens|ENSMUST00000061937|ens|ENSMUST00000029840|gb|AK008791 | Ctbs | -2.10 |
| A_51_P198292 | ref|NM_080452|ref|NM_001166031|ens|ENSMUST00000038600|gb|AB055389 | Mrps2 | -2.10 |
| A_51_P108226 | ref|NM_183249|ens|ENSMUST00000070832|gb|AK003352|gb|AK003164 | 1100001G20Rik | -2.10 |
| A_52_P207509 | ref|NM_172924|ens|ENSMUST00000061552|gb|AK173328|gb|AK090133 | C230081A13Rik | -2.10 |
| A_55_P2049717 | ref|NM_007427|ens|ENSMUST00000005849|gb|AK138796|gb|BC079902 | Agrp | -2.10 |
| A_51_P286748 | ref|NM_011356|ens|ENSMUST00000028389|gb|AK019093|gb|U68058 | Frzb | -2.10 |
| A_51_P361348 | ref|XM_888068|ref|XM_917140|tc|TC1600644|nap|NAP103240-1 | Olfr1293 | -2.10 |
| A_51_P188281 | ref|NM_008656|ens|ENSMUST00000000445|gb|AK083402|gb|X56182 | Myf5 | -2.10 |
| A_55_P2069480 | ref|XM_001475707|ref|XM_001474579 | XM_001475707 | -2.10 |
| A_52_P68087 | ref|NM_177304|ens|ENSMUST00000119686|ens|ENSMUST00000039840|ens|ENSMUST00000149593 | Enpp6 | -2.10 |
| A_55_P2169259 | ref|NM_019792|ref|NR_030782|ens|ENSMUST00000068317|ens|ENSMUST00000145062 | Cyp3a25 | -2.10 |
| A_51_P487004 | ref|NM_027732|ens|ENSMUST00000011493|gb|BC145988|gb|BC138203 | Dmrtc2 | -2.09 |
| A_55_P1983262 | ref|XM_001478787|ref|XM_001479656|tc|TC1684502 | Gm4026 | -2.09 |
| A_30_P01024149 | Unknown | chr10:3229404-3229856_F | -2.09 |
| A_30_P01030676 | Unknown | chr7:117140887-117152562_R | -2.09 |
| A_30_P01022694 | Unknown | chr7:35405125-35415223_F | -2.09 |
| A_30_P01020228 | Unknown | chr15:97856041-97867907_R | -2.09 |
| A_51_P516119 | ref|NM_001011519|ens|ENSMUST00000077580|tc|NP1399046|nap|NAP108744-1 | Olfr1148 | -2.09 |
| A_30_P01027145 | Unknown | chr4:88572008-88572151_F | -2.09 |
| A_55_P2255325 | ref|NM_011831|ens|ENSMUST00000106869|ens|ENSMUST00000084382|gb|AF054842 | Insl5 | -2.09 |
| A_66_P110542 | ens|ENSMUST00000112841|ref|XR_035236|ref|XR_035282|gb|AK079355 | ENSMUST00000112841 | -2.09 |
| A_66_P137374 | ref|XM_001473708|gb|AK042398|tc|TC1648949|riken|A630088K24 | LOC100045219 | -2.09 |
| A_30_P01019552 | Unknown | chr15:36300306-36310544_R | -2.09 |
| A_30_P01024127 | Unknown | chr1:193755364-193770539_F | -2.08 |
| A_55_P2023329 | ens|ENSMUST00000095817|ref|XM_001473279|ref|XM_001474944|nap|NAP059072-1 | ENSMUST00000095817 | -2.08 |
| A_55_P2184345 | ref|XM_901881|ref|XM_923278|tc|TC1690702|tc|TC1675865 | Gm7008 | -2.08 |
| A_55_P1957114 | ref|XM_001472790|ref|XM_001472827|ref|XM_001478649|ref|XM_001478653 | LOC100039380 | -2.08 |
| A_55_P2023364 | ref|NM_183272|ens|ENSMUST00000033147|ens|ENSMUST00000084505|gb|AK018853 | Fam24a | -2.08 |
| A_30_P01031234 | Unknown | chr13:58474388-58487288_F | -2.08 |
| A_52_P545003 | ref|NM_028959|ens|ENSMUST00000036456|gb|AK034892|gb|AK161033 | Cep72 | -2.08 |
| A_51_P106455 | ref|NM_007430|ens|ENSMUST00000026036|gb|AK133411|gb|AK141239 | Nr0b1 | -2.08 |
| A_55_P2180504 | ref|NM_027697|ens|ENSMUST00000100926|gb|AK161342|riken|4930430B19 | 4933413G19Rik | -2.08 |
| A_30_P01028027 | Unknown | chr18:38776580-38841080_R | -2.08 |
| A_55_P2025490 | ref|NM_009400|ref|NM_021985|ens|ENSMUST00000122001|ens|ENSMUST00000040274 | Tnfrsf18 | -2.08 |
| A_55_P1963324 | ref|NM_146494|ens|ENSMUST00000053290|gb|BC051403|tc|TC1594552 | Olfr722 | -2.08 |
| A_55_P2091928 | ref|NM_009017|ref|NM_198193|ens|ENSMUST00000105523|ens|ENSMUST00000065527 | Raet1b | -2.08 |
| A_51_P212068 | ref|NM_029638|ref|NM_001161621|ref|NM_001161622|ens|ENSMUST00000031835 | Abp1 | -2.08 |
| A_30_P01022437 | Unknown | chr12:109603526-109647403_F | -2.08 |
| A_55_P2070977 | ref|XR_032757|nap|NAP070997-1 | Gm13668 | -2.08 |
| A_55_P2121240 | ref|NM_018800|ens|ENSMUST00000118117|ens|ENSMUST00000098785|ens|ENSMUST00000151985 | Syt6 | -2.08 |
| A_30_P01021047 | Unknown | chr8:124364048-124365705_F | -2.08 |
| A_55_P2051983 | ref|NM_001001803|ens|ENSMUST00000076194|gb|BC148575|gb|BC156750 | Spink7 | -2.07 |
| A_55_P2160837 | ref|NM_008123|ens|ENSMUST00000062944|gb|BC125569|gb|BC120513 | Gja8 | -2.07 |
| A_55_P2006708 | ref|NM_009755|ens|ENSMUST00000111001|ens|ENSMUST00000100405|ens|ENSMUST00000022693 | Bmp1 | -2.07 |
| A_51_P107053 | ref|NM_198623|ens|ENSMUST00000057254|gb|AK077072|gb|BC100419 | Ubqln3 | -2.07 |
| A_30_P01023748 | Unknown | chr2:175374875-175465550_F | -2.07 |
| A_51_P241935 | ref|NM_013921|ens|ENSMUST00000147626|ens|ENSMUST00000151448|ens|ENSMUST00000135549 | Prss30 | -2.07 |
| A_55_P2042341 | ref|NM_146772|ens|ENSMUST00000090700|tc|TC1600306|nap|NAP025049-001 | Olfr1189 | -2.07 |
| A_55_P1990250 | ref|NM_207558|ens|ENSMUST00000048478|gb|BC145881|tc|NP955442 | Olfr750 | -2.07 |
| A_51_P440985 | ref|NM_008003|ens|ENSMUST00000033389|gb|AK017829|gb|AF007268 | Fgf15 | -2.07 |
| A_55_P2142499 | ens|ENSMUST00000103502|ref|XM_912564|gb|FM179586|gb|EU568191 | LOC636979 | -2.07 |
| A_30_P01031718 | Unknown | chr3:85806420-85889300_R | -2.07 |
| A_55_P2025483 | ref|NM_178916|ref|NM_001131068|ref|NM_001131069|ens|ENSMUST00000050997 | Rfesd | -2.07 |
| A_55_P2033595 | ref|XM_001480092|ref|XM_001474297|gb|AK137757|gb|D86421 | LOC100048365 | -2.07 |
| A_51_P455338 | ref|NM_053113|ens|ENSMUST00000061936|gb|BC027557|tc|TC1589572 | Ear11 | -2.07 |
| A_51_P376207 | ref|NM_146948|ens|ENSMUST00000074192|gb|BC120521|gb|BC120523 | Olfr342 | -2.07 |
| A_51_P296535 | ref|NM_007734|ens|ENSMUST00000113457|gb|AF169387|gb|AK033387 | Col4a3 | -2.07 |
| A_55_P1958431 | ref|XM_001472997|nap|NAP062970-1 | LOC100044762 | -2.06 |
| A_55_P2118002 | ens|ENSMUST00000064351|ref|XM_001475271|ref|XM_001480416|gb|AK006609 | ENSMUST00000064351 | -2.06 |
| A_55_P2111782 | ref|XM_001480354 | Gm13134 | -2.06 |
| A_30_P01024615 | Unknown | chr3:58730418-58742493_F | -2.06 |
| A_55_P2026836 | ref|XM_001473032 | LOC676792 | -2.06 |
| A_51_P467389 | ref|NM_134226|ens|ENSMUST00000053008|tc|NP496243|nap|NAP057747-1 | Vmn1r89 | -2.06 |
| A_51_P113588 | ref|NM_146050|ens|ENSMUST00000022269|gb|AK009397|gb|AK010206 | Oit1 | -2.06 |
| A_66_P113346 | ref|XM_001473003|ref|XM_001477420|gb|AK042136|tc|TC1612599 | Gm5103 | -2.06 |
| A_55_P2402577 | gb|AK078387|tc|TC1594960|riken|6530439I21|nap|NAP089438-1 | 6530439I21 | -2.06 |
| A_55_P2169668 | ref|XR_030739|ref|XR_031614|nap|NAP060597-1 | Gm6235 | -2.06 |
| A_30_P01024758 | Unknown | chr13:41337973-41379821_F | -2.06 |
| A_55_P2035579 | ref|XM_001472990|gb|AF141019|gb|BC066063|nap|NAP096134-001 | LOC100048858 | -2.06 |
| A_55_P2098170 | ref|NM_146706|ref|NM_146707|ens|ENSMUST00000079827|ens|ENSMUST00000080365 | Olfr401 | -2.06 |
| A_55_P2173857 | ref|XM_001477951|ref|XM_001476563|ref|XM_889502|ref|XM_001472705 | LOC100042664 | -2.06 |
| A_51_P383884 | ens|ENSMUST00000028819|gb|AK014842|tc|TC1594334|riken|4921508D12 | ENSMUST00000028819 | -2.06 |
| A_55_P2128238 | ref|XR_033563 | LOC637224 | -2.06 |
| A_51_P510900 | ref|NM_026460|ens|ENSMUST00000039047|gb|AK007347|gb|AK007510 | Serpini2 | -2.05 |
| A_55_P2145224 | ens|ENSMUST00000103743|ens|ENSMUST00000103749|gb|AF290571|gb|AF138742 | ENSMUST00000103743 | -2.05 |
| A_66_P101132 | ref|NM_178202|ens|ENSMUST00000110473|gb|M25487|gb|BC061044 | Hist1h2bp | -2.05 |
| A_55_P2123616 | ref|NM_001033541|ens|ENSMUST00000101297|gb|AK142438|tc|TC1591130 | Gm5127 | -2.05 |
| A_55_P2022798 | ref|XM_915944 | LOC639496 | -2.05 |
| A_55_P2280811 | gb|AK016151|riken|4930556N08|nap|NAP074582-1 | 4930556N08Rik | -2.05 |
| A_30_P01029837 | Unknown | chr1:138442536-138521080_F | -2.05 |
| A_51_P492346 | ref|NM_026685|ens|ENSMUST00000050389|gb|AK002365|gb|AK057630 | Tmem174 | -2.04 |
| A_55_P2083396 | ref|NM_178702|ens|ENSMUST00000110787|ens|ENSMUST00000110785|ens|ENSMUST00000110784 | Radil | -2.04 |
| A_51_P325281 | ens|ENSMUST00000042610|gb|AK054438|tc|TC1616093|riken|E330025H10 | ENSMUST00000042610 | -2.04 |
| A_52_P135263 | ref|NM_173436|ens|ENSMUST00000041758|gb|AF463499|gb|BC060953 | Cypt2-ps | -2.04 |
| A_55_P1987585 | ref|XM_001473504|ref|XM_001474095|ref|XM_001474243|ref|XM_985872 | LOC100045012 | -2.04 |
| A_30_P01018362 | Unknown | chr15:10718080-10719858_F | -2.04 |
| A_55_P2161347 | ref|NM_001033041|ens|ENSMUST00000038006|gb|AK143962|gb|AK165228 | Acmsd | -2.04 |
| A_55_P2091963 | ref|XM_001480881 | Gm4625 | -2.04 |
| A_30_P01027876 | Unknown | chr12:110653148-110653701_F | -2.04 |
| A_55_P2160416 | ref|NM_028765|ens|ENSMUST00000110344|ens|ENSMUST00000028859|gb|BC048717 | Acoxl | -2.04 |
| A_55_P2098423 | ref|NM_001018013|ens|ENSMUST00000033100|gb|BC139270|gb|AB195681 | Izumo1 | -2.04 |
| A_30_P01017437 | Unknown | chr12:81221532-81232030_F | -2.03 |
| A_30_P01030404 | Unknown | chr9:78368697-78369095_R | -2.03 |
| A_55_P2080511 | ref|NM_177651|ens|ENSMUST00000102713|ens|ENSMUST00000066615 | 4933409G03Rik | -2.03 |
| A_30_P01025753 | Unknown | chr9:78367773-78374723_R | -2.03 |
| A_55_P2096179 | ref|XR_034737 | LOC100048514 | -2.03 |
| A_51_P245405 | ref|NM_008915|ens|ENSMUST00000078434|gb|AK133393|gb|M81475 | Ppp3cc | -2.03 |
| A_55_P2099620 | ref|NM_145998|ens|ENSMUST00000051997|gb|AK138228|gb|S80989 | Hmx2 | -2.03 |
| A_55_P2045976 | ens|ENSMUST00000103274|gb|GU599378|gb|GU599295|gb|GU599307 | ENSMUST00000103274 | -2.03 |
| A_55_P2132626 | ref|XM_001473248|ref|XM_908050|nap|NAP061569-1 | Gm6236 | -2.03 |
| A_30_P01031979 | Unknown | chr18:5567150-5589800_F | -2.03 |
| A_30_P01031558 | Unknown | chr17:13812666-13820504_R | -2.02 |
| A_30_P01027827 | Unknown | chr2:128137419-128141735_R | -2.02 |
| A_66_P103124 | ens|ENSMUST00000103482|gb|AF113109|gb|AF113110|gb|AF045485 | ENSMUST00000103482 | -2.02 |
| A_66_P118212 | ref|NM_001011832|ens|ENSMUST00000080162|tc|NP1398928|nap|NAP108677-1 | Olfr1490 | -2.02 |
| A_51_P309754 | ref|XM_001475864|ref|XM_001475887|gb|DQ237936|gb|U03711 | LOC100046808 | -2.02 |
| A_30_P01021407 | Unknown | chr6:37732925-37784092_F | -2.02 |
| A_65_P10174 | ref|XM_001472496|ref|XM_001479863|ref|XM_001472514|ref|XM_001479866 | Gm2084 | -2.02 |
| A_52_P348201 | ref|NM_201370|ens|ENSMUST00000038907|gb|BC052883|gb|AK163344 | Wee2 | -2.02 |
| A_55_P2054315 | ref|NM_008571|ens|ENSMUST00000015576|gb|BC119248|gb|BC119250 | Mcpt2 | -2.02 |
| A_55_P2007368 | ref|XM_001481005|ref|XM_001481009 | Gm14211 | -2.02 |
| A_55_P2214408 | ref|NR_030676|gb|AK048683|tc|TC1727635|riken|C230004D03 | A130049A11Rik | -2.02 |
| A_51_P455166 | ref|NM_011165|ens|ENSMUST00000021779|gb|AK164009|gb|AK141285 | Prl4a1 | -2.02 |
| A_52_P336768 | tc|TC1615392|nap|NAP052385-1 | TC1615392 | -2.01 |
| A_55_P2236542 | gb|BG079276|tc|TC1683055|nap|NAP038298-1 | C77681 | -2.01 |
| A_55_P2154919 | ref|XM_915550 | LOC639204 | -2.01 |
| A_55_P2060238 | ref|NM_145467|ens|ENSMUST00000038582|ens|ENSMUST00000100289|gb|AK029068 | Itgbl1 | -2.01 |
| A_51_P437692 | ref|NM_009581|ens|ENSMUST00000142416|ens|ENSMUST00000039862|ens|ENSMUST00000128128 | Zp3r | -2.01 |
| A_55_P2145740 | ref|NM_001048219|ref|NM_001048220|ens|ENSMUST00000071780|gb|AK145355 | Nlrp9a | -2.01 |
| A_55_P2058433 | ref|NM_001039555|ref|NM_001104525|ref|NM_010004|ens|ENSMUST00000099472 | Cyp2c68 | -2.01 |
| A_66_P106789 | ref|XM_894271|nap|NAP014486-001 | Gm6970 | -2.01 |
| A_30_P01020194 | Unknown | chr16:40467818-40468474_F | -2.01 |
| A_55_P1976670 | ref|NM_146934|ens|ENSMUST00000072655|gb|AF102520|gb|AF102526 | Olfr46 | -2.01 |
| A_51_P120875 | ref|NM_147034|ens|ENSMUST00000098139|gb|BC100704|gb|BC152882 | Olfr713 | -2.01 |
| A_52_P163011 | ref|NM_194335|gb|AK029812|tc|TC1598989|tc|TC1691204 | Naif1 | -2.01 |
| A_55_P1985346 | ref|XM_001475143|ref|XM_001477850 | Gm13017 | -2.00 |
| A_55_P2117092 | ens|ENSMUST00000095920|ref|XM_001475685|ref|XM_001476125|tc|NP1260081 | ENSMUST00000095920 | -2.00 |
| A_55_P2120373 | ref|XM_994395|ref|XM_001472506|tc|TC1641987|nap|NAP011193-001 | Gm4990 | -2.00 |
| A_66_P116077 | ref|XM_001473750|ref|XM_001474136|gb|AK019125|gb|BY710177 | 2410088K16Rik | -2.00 |
| A_55_P2242575 | gb|BY715062|tc|TC1601038 | 4930466K18Rik | -2.00 |
| A_52_P682996 | ens|ENSMUST00000063442|ref|XM_001476881|ref|XM_890097|tc|NP818152 | Olfr664 | -2.00 |
| A_51_P195153 | ref|NM_013882|ref|NM_001168672|ens|ENSMUST00000023018|gb|AK081582 | Gtse1 | -2.00 |
| A_66_P133703 | ref|XR_005005|ref|XR_031375|nap|NAP103486-1|nap|NAP112518-1 | Gm5705 | -2.00 |
| A_55_P2352652 | gb|BG864327 | Gm12346 | -2.00 |

**Table S5**: Genes up regulated ≥ 2 fold in the cerebral hemisphere of male pups from mothers having FA supplementation during gestation at 4 mg/kg in comparison to mothers at 0.4 mg/kg diet.

| **UniqueID** | **Accession** | **Symbol** | **Fold Change** |
| --- | --- | --- | --- |
| A_55_P2156219 | ens|ENSMUST00000097634|ref|XM_001476022|gb|AK141051|riken|C230022F01 | Gm10549 | 8.20 |
| A_66_P119877 | ens|ENSMUST00000117592|gb|AK015465|tc|NP742345|riken|4930455H04 | ENSMUST00000117592 | 7.69 |
| A_55_P2100335 | ref|XM_897940|ref|XM_924616 | Gm6449 | 7.58 |
| A_55_P1979566 | ref|NM_001025574|ref|NM_175167|ens|ENSMUST00000088924|ens|ENSMUST00000088921 | 5430413K10Rik | 5.95 |
| A_51_P436342 | ref|NM_008268|ens|ENSMUST00000049272|gb|M26283|gb|AK133512 | Hoxb5 | 5.52 |
| A_30_P01029744 | Unknown | chr10:21542515-21579890_F | 5.38 |
| A_51_P455366 | ref|NM_178263|gb|AK085796|gb|BC016493|tc|TC1588562 | Ankrd27 | 5.21 |
| A_30_P01019261 | Unknown | chrX:147610870-147614259_F | 5.10 |
| A_52_P374157 | ref|NM_001008785|ref|NM_001102670|ens|ENSMUST00000032107|ens|ENSMUST00000119582 | Kbtbd8 | 5.05 |
| A_55_P2139092 | ref|XM_001477317|ref|XM_485865|tc|TC1752932|nap|NAP062617-1 | Gm5585 | 4.98 |
| A_55_P1994867 | ref|NM_013617|ens|ENSMUST00000081748|ens|ENSMUST00000104878|ref|XM_001475963 | Olfr64 | 4.95 |
| A_30_P01020309 | Unknown | chr16:44717531-44724206_F | 4.95 |
| A_66_P124775 | ref|NM_172851|ens|ENSMUST00000086738|gb|AK082656|tc|TC1593437 | Cntnap5b | 4.83 |
| A_55_P2013118 | ref|XM_001477435|ref|XM_001479273|tc|TC1694992 | Gm3640 | 4.74 |
| A_55_P2150851 | ens|ENSMUST00000044682|ens|ENSMUST00000064236|gb|AK081075|tc|TC1593053 | ENSMUST00000044682 | 4.55 |
| A_55_P2132819 | ens|ENSMUST00000095633|ref|XM_354985|ref|XM_913544|nap|NAP030943-1 | Gm5145 | 4.52 |
| A_55_P2034285 | ens|ENSMUST00000097030 | ENSMUST00000097030 | 4.52 |
| A_55_P2276889 | gb|AK085451|riken|D630028L22 | D630014O11Rik | 4.41 |
| DCP_20_9 | Unknown | DCP_20_9 | 4.41 |
| A_30_P01031581 | Unknown | chr2:104640443-104659168_R | 4.37 |
| A_55_P2001336 | ref|XM_001472965|ref|XR_032598|ref|XR_032539|ref|XR_032451 | LOC100048857 | 4.33 |
| A_52_P325116 | ens|ENSMUST00000119373|ens|ENSMUST00000121357|ens|ENSMUST00000137454|gb|BC070451 | ENSMUST00000119373 | 4.29 |
| A_51_P114722 | ref|NM_019545|ens|ENSMUST00000029464|gb|AF272947|gb|AK018684 | Hao2 | 4.27 |
| A_55_P2060672 | ref|NM_010452|ens|ENSMUST00000118397|ens|ENSMUST00000048882|ens|ENSMUST00000114434 | Hoxa3 | 4.20 |
| A_66_P140224 | ens|ENSMUST00000119133|gb|AK006881|tc|TC1678225|riken|1700064H15 | ENSMUST00000119133 | 4.20 |
| A_55_P2084492 | ref|XR_031297|ref|XR_032247 | Gm15782 | 4.20 |
| A_30_P01031841 | Unknown | chr15:61153994-61285386_F | 4.17 |
| A_55_P2075378 | ref|XM_001472043|ref|XM_001478914|gb|AK090094|riken|G430108A14 | Gm1999 | 4.17 |
| A_55_P2181645 | ref|XR_034632 | LOC100048338 | 4.10 |
| A_30_P01029411 | Unknown | chr16:90008636-90015586_R | 4.08 |
| A_30_P01033491 | Unknown | chrX:6790023-6896433_R | 4.07 |
| A_55_P2116833 | ref|NM_029886|ens|ENSMUST00000068996|gb|AK078159|tc|TC1581856 | 9430038I01Rik | 4.05 |
| A_55_P1967341 | ref|NM_001111313|ens|ENSMUST00000081668|ens|ENSMUST00000095268|ref|XM_001476653 | Gm9104 | 4.02 |
| A_55_P2064014 | ref|NM_028671|ens|ENSMUST00000114835|ref|XM_001478102|gb|AK015916 | Fam122c | 4.02 |
| A_55_P2165249 | ref|NM_130887|ens|ENSMUST00000121733|ens|ENSMUST00000021646|gb|AK053198 | Papln | 3.95 |
| A_51_P198453 | ens|ENSMUST00000095217|ens|ENSMUST00000044489|tc|TC1659185|nap|NAP099524-001 | ENSMUST00000095217 | 3.92 |
| A_55_P1970097 | ref|NM_001126488|ens|ENSMUST00000078060|ens|ENSMUST00000114328|nap|NAP027228-1 | Tex28 | 3.89 |
| A_51_P202596 | ref|NM_172628|ens|ENSMUST00000051720|gb|AK052534|gb|AK173317 | Sh3tc2 | 3.89 |
| A_55_P1970464 | ens|ENSMUST00000103507|ens|ENSMUST00000103504|ens|ENSMUST00000103550|ref|XM_908887 | ENSMUST00000103507 | 3.88 |
| A_55_P2202524 | gb|AK034200|tc|TC1598905|riken|9330162O12|nap|NAP122182-001 | 9330162012Rik | 3.85 |
| A_55_P2173210 | ens|ENSMUST00000111210|ref|XM_486348|nap|NAP063609-1 | ENSMUST00000111210 | 3.83 |
| A_55_P2029013 | ref|NM_144890|ens|ENSMUST00000045959|ref|XR_031143|gb|AK142982 | BC018465 | 3.82 |
| A_52_P342654 | ref|NM_001081399|ens|ENSMUST00000059906|gb|BC103541|gb|BC101950 | Prss33 | 3.79 |
| A_30_P01029641 | Unknown | chr5:31883451-31884082_F | 3.76 |
| A_55_P2061253 | ref|NM_138944|ens|ENSMUST00000034115|gb|AK084240|gb|AK087546 | Pou4f2 | 3.76 |
| A_55_P2186195 | ref|NM_001024136|ens|ENSMUST00000098085|ens|ENSMUST00000084616|ref|XM_906034 | Anks6 | 3.73 |
| A_30_P01031827 | Unknown | chr13:28477490-28985604_R | 3.73 |
| A_52_P425734 | ref|NM_145146|ens|ENSMUST00000113179|gb|BC100597|gb|AJ011080 | Afm | 3.72 |
| A_52_P625218 | ref|NM_025696|ens|ENSMUST00000078880|gb|AK082681|gb|AK018111 | Sorcs3 | 3.70 |
| A_30_P01029344 | Unknown | chr10:94850300-94855601_R | 3.69 |
| A_55_P1970612 | ref|XM_619699|nap|NAP070743-1 | LOC545383 | 3.64 |
| A_55_P2002426 | ref|NM_021485|ens|ENSMUST00000118483|ens|ENSMUST00000137431|ens|ENSMUST00000130469 | Rps6kb2 | 3.61 |
| A_55_P2391394 | gb|AY672066|gb|AK044157|tc|TC1600804|riken|A830095L23 | AY672066 | 3.60 |
| A_52_P519904 | ref|NM_008422|ens|ENSMUST00000107907|ens|ENSMUST00000107906|gb|S69381 | Kcnc3 | 3.58 |
| A_55_P2159299 | ref|NM_011919|ref|XR_031278|gb|AF177757|gb|AK035490 | Ing1 | 3.57 |
| A_55_P1970570 | ref|NM_001030290|ens|ENSMUST00000066387|tc|TC1601540 | Rdh8 | 3.57 |
| A_52_P24690 | gb|AK038006|gb|AK041363|tc|TC1590472|riken|A130071C16 | AK038006 | 3.56 |
| A_55_P2112702 | ref|XM_001481253 | Gm9462 | 3.53 |
| A_55_P2073489 | ens|ENSMUST00000091905|nap|NAP096137-001 | ENSMUST00000091905 | 3.52 |
| A_55_P2148655 | ref|NM_198410|ens|ENSMUST00000107540|ens|ENSMUST00000107546|ens|ENSMUST00000147948 | Paqr6 | 3.52 |
| A_55_P1966838 | ref|NM_001037713|ens|ENSMUST00000146233|ens|ENSMUST00000140842|tc|TC1667977 | Xaf1 | 3.52 |
| A_52_P113537 | ref|NR_001463|gb|AK051106|tc|TC1664940|riken|D030072M03 | Xist | 3.52 |
| A_51_P126835 | ref|NM_001025102|ref|NM_173750|ens|ENSMUST00000093883|ens|ENSMUST00000028536 | 2700007P21Rik | 3.50 |
| A_55_P2032302 | ens|ENSMUST00000122450|gb|AK045713|tc|TC1589247|riken|B230307C23 | ENSMUST00000122450 | 3.48 |
| A_55_P2055638 | ens|ENSMUST00000113016|gb|AK168829|gb|AK167918|gb|AK169041 | ENSMUST00000113016 | 3.48 |
| A_55_P2005570 | ref|NM_010160|ref|NM_001110231|ref|NM_001160293|ref|NM_001110229 | Celf2 | 3.47 |
| A_51_P503937 | ref|NM_009790|ens|ENSMUST00000110082|ens|ENSMUST00000001204|gb|BC018592 | Calm1 | 3.47 |
| A_65_P11816 | ref|NM_001037848|ens|ENSMUST00000020374|gb|AK172132|tc|TC1591239 | Cnot2 | 3.46 |
| A_51_P363791 | ref|NM_018829|gb|AK004740|gb|BC010667|tc|TC1572627 | Ap3m1 | 3.46 |
| A_55_P2105988 | ens|ENSMUST00000114775|gb|AK161955|riken|6430561E11 | ENSMUST00000114775 | 3.45 |
| A_52_P63343 | ref|NM_001033302|ens|ENSMUST00000036418|gb|AK156084|gb|AK164001 | Gm129 | 3.44 |
| A_30_P01033215 | Unknown | chr9:41300137-41301556_F | 3.42 |
| A_52_P417990 | ref|NM_172754|ref|NM_001045553|ens|ENSMUST00000121886|ens|ENSMUST00000074982 | Zfp868 | 3.42 |
| A_30_P01021928 | Unknown | chr13:54397214-54407430_F | 3.42 |
| A_55_P2018403 | ref|NM_011516|ens|ENSMUST00000029448|ref|XR_031643|gb|Z38118 | Sycp1 | 3.42 |
| A_55_P2027852 | ref|NR_033527|ens|ENSMUST00000098949|ens|ENSMUST00000127460|ens|ENSMUST00000069762 | Ccl25 | 3.41 |
| A_65_P12955 | ens|ENSMUST00000114078|gb|AK082458|gb|AK038296|tc|TC1622497 | ENSMUST00000114078 | 3.41 |
| A_55_P1984886 | ref|NM_011827|ens|ENSMUST00000075062|ens|ENSMUST00000079384|gb|AK008005 | Hcst | 3.41 |
| A_55_P2144481 | ens|ENSMUST00000059880|nap|NAP021787-001 | ENSMUST00000059880 | 3.39 |
| A_30_P01030751 | Unknown | chr7:71093563-71095094_F | 3.37 |
| A_51_P347154 | ref|NM_010084|ens|ENSMUST00000033957|gb|AF167405|gb|AK076633 | Adam18 | 3.37 |
| A_66_P113401 | ref|NM_009557|ens|ENSMUST00000069195|gb|M98502|tc|TC1645389 | Zfp46 | 3.36 |
| A_52_P112110 | ref|NM_145987|ens|ENSMUST00000053263|ens|ENSMUST00000143154|gb|AK162322 | Tmem82 | 3.34 |
| A_66_P136129 | ref|NR_033597|gb|AK076575|tc|TC1632128|riken|4921519A02 | Gm7134 | 3.32 |
| A_30_P01029147 | Unknown | chr5:108540155-108540662_F | 3.32 |
| A_30_P01028348 | Unknown | chr15:38206477-38206961_R | 3.32 |
| A_55_P2096837 | ref|XM_488779|ref|XM_489681 | Gm5484 | 3.32 |
| A_30_P01024836 | Unknown | chrX:123295327-123300036_F | 3.31 |
| A_51_P324651 | ref|NM_181039|ens|ENSMUST00000141158|gb|AK013763|gb|BC078414 | Lphn1 | 3.30 |
| A_55_P2002165 | ref|NM_019929|ens|ENSMUST00000099538|ens|ENSMUST00000020501|gb|AK148306 | Sumo3 | 3.29 |
| A_55_P2117487 | ref|XR_031813|ref|XR_033930 | Gm8276 | 3.27 |
| A_55_P1981415 | ref|NM_019827|ens|ENSMUST00000023507|gb|AK154293|gb|AK088784 | Gsk3b | 3.27 |
| A_51_P426283 | ref|NM_133347|ens|ENSMUST00000136969|ens|ENSMUST00000111991|ens|ENSMUST00000127744 | Dhx30 | 3.26 |
| A_30_P01027883 | Unknown | chrX:11671167-11700242_R | 3.24 |
| A_51_P104172 | ref|NM_153802|ens|ENSMUST00000144578|gb|AK035906|gb|AK032046 | Zfp128 | 3.23 |
| A_52_P109319 | ref|NM_007892|ens|ENSMUST00000029069|gb|X86925|gb|AK156760 | E2f5 | 3.22 |
| A_51_P226962 | ref|NM_015830|ens|ENSMUST00000041641|gb|AK148209|tc|TC1643688 | Solh | 3.19 |
| A_55_P2005730 | ens|ENSMUST00000105399|gb|AK039405|riken|A330040H08|nap|NAP080506-1 | ENSMUST00000105399 | 3.19 |
| A_55_P2033987 | ref|XM_890873|ref|XM_910693|nap|NAP029826-1|nap|NAP068775-1 | Gm6661 | 3.18 |
| A_55_P2348409 | gb|AK084992|gb|AI848933|riken|D430022A14 | D430022A14Rik | 3.17 |
| A_55_P1968895 | ref|NM_013639|ref|NM_001163588|ref|NM_001163589|ens|ENSMUST00000047104 | Prph | 3.16 |
| A_51_P334072 | ref|NM_009989|ens|ENSMUST00000028430|gb|M20625|gb|AK018833 | Cyct | 3.15 |
| A_52_P438082 | ref|NM_009884|ens|ENSMUST00000070191|ens|ENSMUST00000130491|gb|AK169583 | Cebpg | 3.14 |
| A_55_P1968718 | ref|NM_182930|ref|NM_001160268|ens|ENSMUST00000086504|ens|ENSMUST00000038295 | Plekha6 | 3.14 |
| A_55_P2140941 | ref|NM_023476|ref|NM_001168333|ens|ENSMUST00000030560|ens|ENSMUST00000061267 | Tinagl1 | 3.13 |
| A_52_P578790 | ref|NM_013739|ens|ENSMUST00000047877|gb|BC116922|gb|BC120535 | Dok3 | 3.13 |
| A_55_P1956553 | ens|ENSMUST00000107605|ens|ENSMUST00000056442|ref|XM_001004722|ref|XM_001004719 | ENSMUST00000107605 | 3.12 |
| A_52_P680761 | ref|NM_198418|ref|NM_001161367|ref|NM_001161366|ens|ENSMUST00000045717 | Tdrd6 | 3.12 |
| A_55_P2368405 | gb|CO045656 | AI504002 | 3.12 |
| A_55_P2055242 | ref|NM_010229|ens|ENSMUST00000110547|ens|ENSMUST00000049324|ens|ENSMUST00000110549 | Flt3 | 3.11 |
| A_55_P2000284 | ref|NM_008887|ens|ENSMUST00000008090|gb|X75014|tc|TC1587102 | Phox2a | 3.11 |
| A_55_P2035772 | ref|NM_011987|ens|ENSMUST00000023364|ens|ENSMUST00000115807|gb|AK076921 | Pla2g10 | 3.10 |
| A_55_P2013321 | ref|XM_001479464 | Gm9573 | 3.10 |
| A_52_P232314 | ref|XR_030954|nap|NAP102462-1|nap|NAP111325-1 | Gm7459 | 3.10 |
| A_55_P2146668 | ref|XM_001478971|ref|XM_001481080 | Gm4658 | 3.09 |
| A_55_P2067151 | ref|XR_034366|ref|XR_032449 | Gm7985 | 3.08 |
| A_52_P486234 | ens|ENSMUST00000108216|gb|AK033497|gb|AK031520|gb|BB040069 | ENSMUST00000108216 | 3.08 |
| A_55_P2069331 | ens|ENSMUST00000106975|ens|ENSMUST00000144207|ens|ENSMUST00000098237|gb|AK135201 | ENSMUST00000106975 | 3.08 |
| A_52_P683211 | ref|XR_031582|ref|XR_031686|nap|NAP102269-1|nap|NAP112091-1 | Gm5910 | 3.08 |
| A_30_P01027213 | Unknown | chr11:59217249-59217785_R | 3.06 |
| A_55_P2105017 | ref|XR_030862 | LOC100044649 | 3.06 |
| A_30_P01023956 | Unknown | chr3:65460193-65460769_R | 3.05 |
| A_52_P305658 | ref|NM_030699|ref|NM_001163351|ref|NM_001163349|ref|NM_133488 | Ntng1 | 3.04 |
| A_51_P151732 | ref|NM_019645|ens|ENSMUST00000027667|gb|Y07941|tc|TC1581735 | Pkp1 | 3.04 |
| A_51_P222280 | ref|NM_019777|ens|ENSMUST00000062108|gb|AK180824|gb|AK088580 | Ikbke | 3.04 |
| A_30_P01025949 | Unknown | chr9:31873801-31874407_F | 3.04 |
| A_55_P2236138 | gb|AK015404|riken|4930447F24|nap|NAP074161-1 | 4930447F24Rik | 3.03 |
| A_30_P01029220 | Unknown | chr4:115639749-115641787_R | 3.02 |
| A_55_P2095839 | ens|ENSMUST00000114961|nap|NAP065329-1 | ENSMUST00000114961 | 3.02 |
| A_65_P11539 | ens|ENSMUST00000082026|gb|BC146602|gb|AK155029|gb|BC040249 | Dennd4c | 3.02 |
| A_55_P1969072 | ref|NM_007624|ens|ENSMUST00000081455|ens|ENSMUST00000031862|ens|ENSMUST00000114446 | Cbx3 | 3.01 |
| A_30_P01024431 | Unknown | chr18:6144775-6202232_R | 3.01 |
| A_30_P01019633 | Unknown | chr18:35977112-35977538_F | 3.01 |
| A_55_P2011950 | ens|ENSMUST00000103552|ref|XM_917510|gb|AB159872|gb|AB160753 | LOC435333 | 3.01 |
| A_55_P2068315 | ref|NM_182957 | Speer4c | 3.00 |
| A_55_P2037689 | ref|XR_031802|ref|XR_030527 | Gm13549 | 2.99 |
| A_66_P104118 | ref|XR_033950|nap|NAP114231-1 | EG667078 | 2.99 |
| A_51_P374436 | ref|NM_177450|ens|ENSMUST00000070139|gb|AK162089|gb|AK085308 | Cndp1 | 2.99 |
| A_55_P1994862 | ref|NM_001081215|ens|ENSMUST00000093485|ens|ENSMUST00000070631|gb|AK136403 | Ddx60 | 2.99 |
| A_30_P01023101 | Unknown | chr17:27703181-27716306_R | 2.97 |
| A_51_P162162 | ref|NM_009349|ens|ENSMUST00000003569|gb|AK002281|gb|AK013010 | Inmt | 2.97 |
| A_66_P101664 | ref|NM_178778|ens|ENSMUST00000038874|gb|AK142499|riken|D530048H07 | Scai | 2.96 |
| A_30_P01023652 | Unknown | XIST | 2.96 |
| A_55_P1980583 | ref|NM_175036|ens|ENSMUST00000106927|ens|ENSMUST00000030254|gb|AK009569 | Leprot | 2.96 |
| A_51_P385718 | ref|NM_026862|ens|ENSMUST00000063956|gb|BC027283|gb|BC005615 | Cd177 | 2.96 |
| A_30_P01023072 | Unknown | chr15:68161768-68193518_R | 2.96 |
| A_52_P600304 | ref|NM_026165|gb|AK030346|riken|5230400P09|nap|NAP115956-1 | Slc25a46 | 2.96 |
| A_30_P01031408 | Unknown | chr8:67552315-67561235_R | 2.93 |
| A_51_P318856 | ref|NM_145935|gb|AK039262|gb|BC024434|gb|BC010799 | Glyat | 2.93 |
| A_55_P2361731 | gb|BU848224|tc|TC1678489 | AW047481 | 2.93 |
| A_55_P2099061 | ref|XR_031698|ref|XR_033180|nap|NAP094021-001 | Gm12933 | 2.92 |
| A_51_P378336 | ref|NM_172687|ens|ENSMUST00000029909|gb|AK028680|gb|AK142287 | Coq3 | 2.92 |
| A_30_P01022821 | Unknown | chr7:91558475-91733625_F | 2.92 |
| A_30_P01021491 | Unknown | chr16:44717531-44724206_R | 2.91 |
| A_52_P594927 | ref|NM_146091|ref|NM_001163505|ens|ENSMUST00000025668|gb|AK028842 | Atl3 | 2.91 |
| A_52_P638459 | ref|NM_013653|ens|ENSMUST00000035938|gb|BC033508|gb|AK003101 | Ccl5 | 2.90 |
| A_51_P499551 | ref|NM_133983|ens|ENSMUST00000039788|gb|BC019436|gb|AK155114 | Cd276 | 2.90 |
| A_52_P30312 | ref|NM_009913|ref|NM_001166625|ens|ENSMUST00000111454|gb|AK050615 | Ccr9 | 2.90 |
| A_30_P01017862 | Unknown | chr4:116790373-116797823_R | 2.89 |
| A_30_P01021534 | Unknown | chr14:28218945-28241903_R | 2.89 |
| A_51_P289131 | ref|NM_010059|gb|AK076777|gb|AK169570|gb|D64107 | Dmc1 | 2.89 |
| A_30_P01025143 | Unknown | chr1:163528200-163528398_F | 2.89 |
| A_51_P295131 | ref|NM_011039|gb|AF254422|nap|NAP014244-001 | Pax7 | 2.88 |
| A_55_P2116180 | ref|NM_145379|ens|ENSMUST00000117718|ens|ENSMUST00000033386|gb|AK157988 | Mrgprf | 2.88 |
| A_55_P2041501 | ref|XM_620708|ref|XM_913611|tc|TC1726497 | Gm5915 | 2.87 |
| A_55_P1958652 | ref|NM_007477|ens|ENSMUST00000063347|ens|ENSMUST00000057921|gb|AK077591 | Arf2 | 2.87 |
| A_55_P2075553 | ref|NM_183391|ens|ENSMUST00000086084|gb|AJ577579|tc|TC1587959 | Tnfsf18 | 2.87 |
| A_30_P01026003 | Unknown | chr11:96175764-96176029_F | 2.87 |
| A_55_P2024993 | ref|NM_010459|ens|ENSMUST00000049241|gb|AK133945|tc|TC1638091 | Hoxb4 | 2.87 |
| A_55_P2178568 | ref|NM_133715|ens|ENSMUST00000041385|ens|ENSMUST00000107024|gb|AK046763 | Arhgap27 | 2.87 |
| A_55_P2078690 | ref|XM_001474796 | LOC100045716 | 2.86 |
| A_51_P360396 | ref|NM_001025305|ref|NM_009334|ens|ENSMUST00000064976|ens|ENSMUST00000027059 | Tcfap2b | 2.85 |
| A_51_P310741 | ref|NM_146930|ens|ENSMUST00000057477|tc|NP646686|nap|NAP022632-001 | Olfr791 | 2.85 |
| A_55_P2334684 | ref|NM_001081267|ens|ENSMUST00000107153|gb|AK029311|tc|TC1597856 | Rsf1 | 2.84 |
| A_51_P278427 | ref|NM_146051|ref|NM_178141|ens|ENSMUST00000039207|gb|BC057656 | 3830406C13Rik | 2.84 |
| A_52_P496726 | ref|NM_009026|ens|ENSMUST00000062405|gb|AK038932|gb|AF009246 | Rasd1 | 2.84 |
| A_52_P194851 | ref|NM_001162906|ens|ENSMUST00000044206|ens|ENSMUST00000110617|ref|XM_486078 | 2410089E03Rik | 2.83 |
| A_51_P339154 | ens|ENSMUST00000055719|ref|XR_035099|gb|BC025446|tc|TC1677835 | ENSMUST00000055719 | 2.82 |
| A_51_P449233 | ref|NM_172444|ref|NM_001040426|ens|ENSMUST00000034829|ens|ENSMUST00000098660 | Thsd4 | 2.82 |
| A_30_P01027106 | Unknown | chr5:113920850-113950200_F | 2.82 |
| A_30_P01017468 | Unknown | chr11:87841800-87858725_R | 2.82 |
| A_66_P126480 | ref|XM_001472413|ref|XM_001477785|gb|AK085764|gb|BB603218 | Gm13861 | 2.82 |
| A_52_P628067 | ref|NM_013538|ens|ENSMUST00000024270|gb|AK008606|gb|AK169792 | Cdca3 | 2.81 |
| A_55_P2025815 | ref|XM_001478120|tc|TC1635172 | LOC100048869 | 2.81 |
| A_51_P339331 | ref|NM_029002|ens|ENSMUST00000071588|gb|AK147163|gb|AK018683 | Nkx6-3 | 2.80 |
| A_55_P2103075 | ens|ENSMUST00000112988|gb|AK039479|tc|TC1634443|riken|A330048M13 | ENSMUST00000112988 | 2.80 |
| A_55_P2039530 | ens|ENSMUST00000099065|gb|AK135602|tc|TC1721917|riken|7330401N19 | ENSMUST00000099065 | 2.79 |
| A_55_P2038514 | ref|XM_001472992|ref|XM_001473858 | LOC100044759 | 2.79 |
| A_55_P2045777 | ref|XM_001475033|ref|XM_001478809|nap|NAP095730-001 | Gm7902 | 2.79 |
| A_51_P211765 | ref|NM_145495|ens|ENSMUST00000025818|gb|BC011277|tc|TC1577567 | Rin1 | 2.79 |
| A_51_P390038 | ref|NM_177834|ens|ENSMUST00000035577|ens|ENSMUST00000153695|gb|AY773477 | Cpa6 | 2.79 |
| A_30_P01029441 | Unknown | chr5:74340084-74346709_R | 2.79 |
| A_52_P632601 | ref|NM_183177|ens|ENSMUST00000080905|gb|BC052046|tc|TC1587744 | Zfp811 | 2.79 |
| A_51_P426096 | ref|NM_010810|ens|ENSMUST00000018767|gb|BC119057|gb|BC120655 | Mmp7 | 2.78 |
| A_55_P2063761 | ref|XM_001474953|ref|XM_001477955|gb|AK050857|riken|D030026J23 | Gm2567 | 2.78 |
| A_30_P01030148 | Unknown | chr6:31233512-31241062_F | 2.77 |
| A_52_P280114 | ref|NM_172803|ens|ENSMUST00000101460|ens|ENSMUST00000037488|gb|BC156169 | Dock4 | 2.77 |
| A_55_P2137494 | ref|XR_031915|ref|XR_031488 | LOC634135 | 2.77 |
| A_55_P1991668 | ref|NM_001007574|ref|NM_001168557|ens|ENSMUST00000112671|gb|AK036000 | A830010M20Rik | 2.76 |
| A_55_P2032493 | ref|NM_001111145|ref|XM_917855|tc|TC1604714 | Gm514 | 2.76 |
| A_55_P2109782 | ref|NM_007677|ens|ENSMUST00000004655|gb|BC100345|tc|TC1588518 | Psg17 | 2.76 |
| A_55_P2161824 | ref|NM_133205|ens|ENSMUST00000082016|ens|ENSMUST00000113769|gb|BC017145 | Arr3 | 2.76 |
| A_66_P111735 | ref|XM_894409|ref|XM_904701|nap|NAP093408-001 | Gm4880 | 2.74 |
| A_55_P2182134 | ref|NM_030174|ens|ENSMUST00000099361|ens|ENSMUST00000109589|ens|ENSMUST00000126960 | Mctp1 | 2.74 |
| A_30_P01029474 | Unknown | chrX:93157194-93160542_R | 2.74 |
| A_55_P2179582 | ref|NM_153079|ens|ENSMUST00000037682|gb|AK036756|gb|BB171918 | Nmur2 | 2.74 |
| A_55_P2149990 | ref|XM_001474702 | LOC100044124 | 2.73 |
| A_55_P2055369 | ref|XM_001474166|ref|XM_001474184|tc|TC1780673 | LOC100045699 | 2.73 |
| A_51_P321150 | ref|NM_017372|ens|ENSMUST00000092163|gb|AK148516|gb|AK153475 | Lyz2 | 2.73 |
| A_55_P2049287 | ref|XR_033618 | LOC100047207 | 2.72 |
| A_55_P2023878 | ens|ENSMUST00000097980|gb|AK160947|riken|4022406H20 | ENSMUST00000097980 | 2.72 |
| A_66_P119283 | ref|XM_001477076|ref|XM_001478554|gb|AK080098|tc|TC1589378 | LOC675594 | 2.72 |
| A_55_P2095508 | ens|ENSMUST00000121245|ens|ENSMUST00000119941|ens|ENSMUST00000121803|ref|XM_001478695 | ENSMUST00000121245 | 2.72 |
| A_51_P259555 | ref|NM_172876|ens|ENSMUST00000030662|gb|AK053051|gb|BC121828 | Gpatch3 | 2.72 |
| A_51_P374991 | ref|NR_003555|gb|AK018294|gb|BC096375|gb|AK039785 | Vmn2r29 | 2.72 |
| A_52_P441036 | ref|NM_153393|ens|ENSMUST00000102765|gb|AK162470|gb|AK078486 | Col23a1 | 2.72 |
| A_30_P01031292 | Unknown | chr9:13349094-13352732_F | 2.72 |
| A_55_P2321185 | gb|AK015643|tc|TC1726966|riken|4930488B22|nap|NAP074289-1 | 4930488B22Rik | 2.71 |
| A_55_P2134172 | ref|NM_001008793|ref|NM_001008792|ref|NM_028640|ref|NM_001008795 | Whrn | 2.71 |
| A_55_P2207342 | ref|XR_035221|gb|AK144510|tc|TC1687180|riken|G630085G17 | 1810059H22Rik | 2.71 |
| A_51_P500082 | ref|NM_001110517|ref|NM_001101605|ens|ENSMUST00000112467|gb|AK090072 | Gm14446 | 2.71 |
| A_52_P256955 | ref|NM_177585|ens|ENSMUST00000063263|gb|AK052136|tc|TC1592225 | Iqcj | 2.70 |
| A_55_P2057380 | ref|XR_033591|ref|XR_033150 | Gm6745 | 2.70 |
| A_66_P136569 | ens|ENSMUST00000103515|gb|AJ277813|gb|EU568213|gb|AM262178 | ENSMUST00000103515 | 2.70 |
| A_55_P2120155 | ref|NM_001081291|ens|ENSMUST00000080351|ens|ENSMUST00000113440|gb|BC151009 | Ccdc88b | 2.70 |
| A_55_P2185840 | ref|NM_010924|ens|ENSMUST00000034808|gb|AK006371|gb|U86105 | Nnmt | 2.70 |
| A_55_P2006300 | ref|NM_010661|ens|ENSMUST00000017741|tc|TC1578522|nap|NAP009850-001 | Krt12 | 2.70 |
| A_55_P2397400 | gb|AK156640|riken|F830034J09 | F830034J09Rik | 2.70 |
| A_30_P01017751 | Unknown | chr4:57372663-57377164_R | 2.70 |
| A_51_P497741 | ref|NM_029440|ens|ENSMUST00000121108|ens|ENSMUST00000110502|gb|AK087389 | 4930434E21Rik | 2.70 |
| A_52_P146711 | ref|NM_009432|ref|NM_001165939|ref|NM_001165940|ens|ENSMUST00000029450 | Tshb | 2.70 |
| A_55_P2152528 | ref|NM_001177356 | Gm3852 | 2.70 |
| A_52_P345539 | ref|NM_009484|ens|ENSMUST00000069309|ens|ENSMUST00000154666|ens|ENSMUST00000143286 | Uty | 2.69 |
| A_55_P2019287 | ref|NM_009541|gb|AK134251|riken|5930405B01|nap|NAP037839-1 | Zbtb17 | 2.69 |
| A_55_P2102027 | ref|XM_001480200|tc|TC1705674 | LOC100048297 | 2.69 |
| A_55_P2174977 | ens|ENSMUST00000121162|ens|ENSMUST00000120044 | ENSMUST00000121162 | 2.69 |
| A_55_P2160862 | ref|NM_011721|ref|NM_001122822|ens|ENSMUST00000033990|ens|ENSMUST00000033991 | Wrn | 2.69 |
| A_55_P2050226 | gb|AY072938|gb|AY072796|tc|TC1596586|tc|TC1596584 | Ccrl1 | 2.68 |
| A_30_P01033406 | Unknown | chr18:46651944-46652663_R | 2.68 |
| A_55_P2169004 | ref|NM_019475|ens|ENSMUST00000079234|gb|BC137911|gb|BC137912 | Olfr157 | 2.68 |
| A_30_P01021300 | Unknown | chr13:94069868-94070007_R | 2.67 |
| A_30_P01019307 | Unknown | chr4:88583425-88584070_R | 2.67 |
| A_55_P2008755 | ref|XM_001473763|ref|XM_001477448|gb|BC004614 | Gm11945 | 2.67 |
| A_51_P136337 | ref|NM_176963|ens|ENSMUST00000039205|gb|AK040998|gb|AK170014 | Galm | 2.67 |
| A_55_P2081660 | ref|NM_029067|ref|NM_028848|ens|ENSMUST00000110945|ens|ENSMUST00000027908 | Spata17 | 2.67 |
| A_52_P458647 | ens|ENSMUST00000054878|gb|AK021182|tc|TC1613289|riken|C330006D17 | ENSMUST00000054878 | 2.66 |
| A_51_P519301 | ref|NM_145856|ens|ENSMUST00000039046|gb|AB116259|gb|AF458064 | Il17f | 2.66 |
| A_55_P1983268 | ens|ENSMUST00000088510|ens|ENSMUST00000151004|ens|ENSMUST00000115367|ref|XM_976954 | ENSMUST00000088510 | 2.66 |
| A_30_P01023676 | Unknown | chr8:60132300-60146830_F | 2.65 |
| A_55_P1952304 | ref|XM_001480062 | LOC100048073 | 2.65 |
| A_55_P2077278 | ref|NM_001017362|ens|ENSMUST00000084698|gb|BC141237|tc|TC1603574 | Arid3c | 2.65 |
| A_55_P2017510 | ref|NM_028883|ens|ENSMUST00000093096|ens|ENSMUST00000049156|gb|AK014579 | 4632415K11Rik | 2.65 |
| A_52_P248673 | ref|NM_146757|ens|ENSMUST00000098211|gb|BC141579|gb|BC146589 | Olfr583 | 2.65 |
| A_51_P154780 | ref|NM_011691|ref|NM_001163815|ens|ENSMUST00000005889|gb|X64361 | Vav1 | 2.65 |
| A_30_P01028922 | Unknown | chr18:63355660-63369610_R | 2.65 |
| A_55_P2181334 | ref|NM_176921|ens|ENSMUST00000085658|ref|XM_001479950|gb|AK047220 | 6030419C18Rik | 2.65 |
| A_66_P120205 | ens|ENSMUST00000084298|gb|AK082664|gb|AK137318|tc|TC1587526 | ENSMUST00000084298 | 2.64 |
| A_55_P1972617 | ref|XM_001472128 | LOC675714 | 2.64 |
| A_55_P2084496 | ref|NM_023672|ref|NM_198438|ens|ENSMUST00000106761|ens|ENSMUST00000097933 | Ssbp3 | 2.64 |
| A_55_P1990483 | ref|NM_144560|ref|NM_001190408|ens|ENSMUST00000037146|ens|ENSMUST00000109895 | Gas2l1 | 2.63 |
| A_52_P311853 | ref|NM_030143|ens|ENSMUST00000053855|gb|AK029362|gb|AK053333 | Ddit4l | 2.63 |
| A_51_P405375 | ref|NM_175358|ens|ENSMUST00000042070|gb|AK029137|gb|AK132304 | Zdhhc15 | 2.63 |
| A_66_P138406 | ref|NM_027218|ens|ENSMUST00000077228|gb|AY230260|gb|AK007794 | Clec4b1 | 2.62 |
| A_55_P2030204 | ref|NM_009297|ens|ENSMUST00000108314|ens|ENSMUST00000002121|gb|U40375 | Supt6h | 2.62 |
| A_52_P533707 | ref|NM_007389|ens|ENSMUST00000028515|ref|XM_001476441|gb|AK132589 | Chrna1 | 2.62 |
| A_55_P2125252 | ens|ENSMUST00000114923|tc|TC1631514|nap|NAP040607-1 | ENSMUST00000114923 | 2.62 |
| A_55_P2184501 | ens|ENSMUST00000115863|gb|AK037793|tc|TC1734552|tc|TC1636831 | ENSMUST00000115863 | 2.62 |
| A_55_P2351670 | gb|AK038598|tc|TC1649622|riken|A230049F01|nap|NAP080192-1 | 1700112J16Rik | 2.62 |
| A_52_P151528 | ref|NM_001164658|ref|NM_029714|ens|ENSMUST00000069861|ens|ENSMUST00000047846 | Catsperg1 | 2.62 |
| A_52_P444611 | ens|ENSMUST00000109290|gb|AK049857|gb|BB426814|tc|TC1590499 | ENSMUST00000109290 | 2.62 |
| A_52_P305230 | ref|NM_198610|ens|ENSMUST00000039331|gb|AK137439|gb|BC055811 | Igsf21 | 2.61 |
| A_55_P2089233 | ref|NM_011136|ens|ENSMUST00000034554|ens|ENSMUST00000114429|gb|AK135080 | Pou2af1 | 2.61 |
| A_55_P2038582 | ref|NM_001044697|ref|NM_001044698|ref|NM_001044699|ref|NM_001044700 | Zfp2 | 2.61 |
| A_55_P2122394 | ref|XM_001476042 | Gm9608 | 2.61 |
| A_51_P169415 | ref|NM_133167|ens|ENSMUST00000023072|ref|XM_001001678|ref|XM_001001690 | Parvb | 2.60 |
| A_55_P1983638 | ref|NM_027418|ref|NM_015806|ens|ENSMUST00000049355|gb|BC024684 | Mapk6 | 2.60 |
| A_66_P120707 | ref|NM_175367|ens|ENSMUST00000052969|gb|AK036612|tc|TC1586570 | Ston2 | 2.60 |
| A_55_P1964832 | ref|NM_016678|ens|ENSMUST00000030198|gb|AK157885|gb|AV245803 | Reck | 2.60 |
| A_51_P153013 | ref|NR_027800|ens|ENSMUST00000042143|gb|AK009371|gb|AK010055 | Gm16516 | 2.60 |
| A_30_P01019388 | Unknown | chr16:25517037-25531362_F | 2.59 |
| A_55_P2459897 | ref|NM_175628|ens|ENSMUST00000032203|ref|XR_005046|gb|AK086336 | A2m | 2.58 |
| A_30_P01023174 | Unknown | chr13:34027900-34052050_F | 2.58 |
| A_55_P2079855 | ref|NM_011166|ens|ENSMUST00000091680|gb|AF011384|gb|AF015563 | Prl6a1 | 2.58 |
| A_51_P503247 | ref|NM_029300|ens|ENSMUST00000085113|gb|AK005734|gb|BC125546 | Iqcf5 | 2.58 |
| A_55_P2042600 | ref|XM_001473076|ref|XM_001475398|gb|AK158677|riken|K230050P16 | XM_001473076 | 2.58 |
| A_30_P01031083 | Unknown | chr15:84737071-84753414_R | 2.58 |
| A_51_P203955 | ref|NM_010260|ens|ENSMUST00000029937|gb|AF109168|gb|AF077007 | Gbp2 | 2.57 |
| A_55_P2008244 | ref|XR_031867|ref|XR_031912|gb|BI734812 | Gm5511 | 2.57 |
| A_52_P22324 | ref|NM_023129|ref|NM_001141927|ens|ENSMUST00000046221|gb|AK052199 | Pln | 2.57 |
| A_30_P01032275 | Unknown | chr10:114762889-114763210_F | 2.57 |
| A_51_P513013 | ref|NM_144912|ens|ENSMUST00000049009|gb|AY297460|gb|BC021784 | Rad9b | 2.57 |
| A_55_P2166607 | ref|NM_028718|ens|ENSMUST00000047242|gb|AY613437|gb|BC117868 | Traf3ip1 | 2.57 |
| A_55_P2084703 | ref|NM_133360|ens|ENSMUST00000020843|ens|ENSMUST00000103201|ref|XR_005038 | Acaca | 2.57 |
| A_55_P2065074 | ens|ENSMUST00000085463|ens|ENSMUST00000063046|gb|AK133334|riken|4932434O14 | ENSMUST00000085463 | 2.56 |
| A_52_P205031 | ref|NM_001113384|ens|ENSMUST00000034199|gb|M36778|tc|TC1584512 | Gnao1 | 2.56 |
| A_52_P367745 | ref|NM_007383|ens|ENSMUST00000150291|ens|ENSMUST00000031524|gb|AK155361 | Acads | 2.56 |
| A_30_P01020518 | Unknown | chr9:78104935-78116974_F | 2.56 |
| A_52_P115579 | ref|NM_173439|ens|ENSMUST00000042732|gb|AK011438|gb|BC098203 | Fbxo45 | 2.56 |
| A_51_P260265 | ref|NM_010469|ens|ENSMUST00000111980|ens|ENSMUST00000047904|gb|J03770 | Hoxd4 | 2.56 |
| A_66_P125496 | ref|XR_033785|ref|XR_034221|nap|NAP062887-1 | Rpl19-ps10 | 2.56 |
| A_55_P1997490 | ref|NM_001177412|gb|BC059867|tc|TC1696922 | Usp35 | 2.56 |
| A_52_P237997 | ref|NM_177274|ens|ENSMUST00000041425|gb|AK035218|tc|TC1590147 | Negr1 | 2.55 |
| A_55_P2243558 | gb|BG084090 | 2310007H06Rik | 2.55 |
| A_55_P2040044 | ref|XM_001477839|ref|XM_891048|ref|XM_001475922 | Gm16434 | 2.55 |
| A_55_P2093814 | ens|ENSMUST00000070905 | ENSMUST00000070905 | 2.54 |
| A_55_P2030354 | ref|XM_001478488 | LOC100047604 | 2.54 |
| A_55_P2065506 | ens|ENSMUST00000103513|ens|ENSMUST00000103510|gb|U58290|gb|Z22117 | LOC639930 | 2.54 |
| A_55_P2213583 | gb|AK015129|tc|TC1623619|riken|4930413F20|nap|NAP074012-1 | 4930413F20Rik | 2.54 |
| A_55_P2141835 | ref|XM_001474721|ref|XM_001480742 | Gm4591 | 2.54 |
| A_51_P365674 | ref|NM_147002|ens|ENSMUST00000079955|nap|NAP097636-001|nap|NAP108642-1 | Olfr174 | 2.54 |
| A_55_P2070331 | ens|ENSMUST00000099340|gb|AK146072|tc|TC1705349|riken|I530028F09 | ENSMUST00000099340 | 2.54 |
| A_55_P2149189 | ref|XM_001478257|ref|XM_001479146|gb|CJ055109 | Gm3997 | 2.54 |
| A_30_P01020724 | Unknown | chr18:36449530-36456800_F | 2.53 |
| A_52_P184149 | ref|NM_008638|ens|ENSMUST00000005810|gb|AK192847|gb|AK190402 | Mthfd2 | 2.53 |
| A_51_P456721 | ref|NM_013478|ens|ENSMUST00000035390|gb|D21059|gb|BC061646 | Azgp1 | 2.53 |
| A_52_P594302 | ref|NM_001077688|gb|AF188506|tc|TC1669010|nap|NAP036747-1 | Lrba | 2.53 |
| A_55_P1999289 | ref|NM_026924|ref|NM_152947|ens|ENSMUST00000110022|ens|ENSMUST00000103171 | Ovol2 | 2.53 |
| A_51_P407193 | ref|NM_133840|ens|ENSMUST00000028475|gb|BC003237|gb|AK169709 | Clp1 | 2.53 |
| A_52_P110070 | ref|NR_033596|gb|AK017567|tc|NP741065|riken|5730416F02 | 5730416F02Rik | 2.53 |
| A_66_P130993 | ref|XR_030720|ref|XR_031410 | Gm6140 | 2.53 |
| A_55_P2067011 | ens|ENSMUST00000109105|gb|AK087703|tc|TC1588648|riken|E330009F12 | ENSMUST00000109105 | 2.53 |
| A_55_P1975080 | ref|NM_009467|ens|ENSMUST00000067790|ens|ENSMUST00000113327|gb|X06358 | Ugt2b5 | 2.53 |
| A_55_P2036698 | ref|NM_146944|ens|ENSMUST00000112950|ens|ENSMUST00000056865|tc|TC1647591 | Olfr348 | 2.52 |
| A_52_P329250 | ref|NM_007690|ens|ENSMUST00000024627|gb|L10410|gb|BC115822 | Chd1 | 2.52 |
| A_55_P1954017 | ref|XR_033068|ref|XR_033349|nap|NAP113704-1 | Gm8970 | 2.52 |
| A_51_P476960 | ref|NM_001081391|ens|ENSMUST00000110261|ens|ENSMUST00000022923|ens|ENSMUST00000100670 | Csmd3 | 2.51 |
| A_52_P139788 | ens|ENSMUST00000120678|gb|AK020115|tc|TC1587912|riken|6720426B09 | ENSMUST00000120678 | 2.51 |
| A_52_P316933 | ref|NM_172507|gb|AK049293|gb|BC038473|gb|AK077373 | Sh3bgrl2 | 2.51 |
| A_52_P373065 | ref|NM_010085|ref|NM_001009547|ref|NM_145745|ens|ENSMUST00000110412 | Adam26a | 2.51 |
| A_55_P2081945 | ref|XM_001476869 | Gm5137 | 2.51 |
| A_55_P2019949 | ref|XM_001474328 | LOC100046093 | 2.51 |
| A_55_P1959580 | ref|XR_034312|ref|XR_032425 | Gm8347 | 2.51 |
| A_51_P403049 | ref|NM_001081179|ens|ENSMUST00000097281|gb|AK141783|gb|AK052391 | Heatr5b | 2.51 |
| A_55_P2147306 | ens|ENSMUST00000034643|ens|ENSMUST00000081943|gb|AF060570|tc|TC1589197 | ENSMUST00000034643 | 2.50 |
| A_55_P2139984 | ref|NM_027438|ens|ENSMUST00000061425|gb|BC116687|gb|BC116688 | Pnma1 | 2.50 |
| A_55_P2160965 | ref|NM_173738|ens|ENSMUST00000072567|ens|ENSMUST00000091530|ens|ENSMUST00000085792 | BC027344 | 2.50 |
| A_30_P01030254 | Unknown | chrX:34344102-34344294_F | 2.50 |
| A_66_P133225 | ref|NM_178795|ens|ENSMUST00000110622|ens|ENSMUST00000155568|ens|ENSMUST00000132114 | Ppip5k1 | 2.50 |
| A_55_P2020183 | ref|XM_001473211|tc|TC1766543 | LOC100044864 | 2.50 |
| A_55_P2145922 | ens|ENSMUST00000065458|gb|BC116417|gb|BC116418|gb|BC128497 | ENSMUST00000065458 | 2.50 |
| A_55_P2252891 | gb|AK160358|tc|TC1620325|riken|A930017D03 | D11Ertd717e | 2.49 |
| A_55_P1992582 | ref|NM_008256|ens|ENSMUST00000090746|gb|AK004865|tc|TC1572016 | Hmgcs2 | 2.49 |
| A_55_P2108820 | ens|ENSMUST00000051989|gb|AK031900|tc|TC1586989|riken|6330442E02 | ENSMUST00000051989 | 2.49 |
| A_51_P431772 | ens|ENSMUST00000039415|gb|AK006243|tc|TC1590636|riken|1700022J01 | ENSMUST00000039415 | 2.49 |
| A_55_P2069974 | ref|NM_001142731|ref|NM_134112|ens|ENSMUST00000025992|ens|ENSMUST00000092040 | Kctd1 | 2.49 |
| A_52_P248343 | ref|NM_153789|ref|NM_181043|ens|ENSMUST00000038275|gb|AY434449 | Mylip | 2.49 |
| A_55_P2053597 | ref|XR_031259|ref|XR_033241 | Gm8467 | 2.49 |
| A_52_P356228 | ref|NM_178746|ens|ENSMUST00000091218|gb|AK076463|tc|TC1585667 | Slc38a9 | 2.49 |
| A_51_P380709 | ref|NM_029761|ref|NM_001163686|ens|ENSMUST00000029075|gb|AF418208 | Dok5 | 2.49 |
| A_55_P2154639 | ens|ENSMUST00000115135|gb|AK134880|riken|6430503A17 | ENSMUST00000115135 | 2.49 |
| A_52_P304720 | ref|NM_018827|ens|ENSMUST00000132648|ens|ENSMUST00000008032|gb|BC160203 | Crlf1 | 2.49 |
| A_55_P2042121 | ref|NM_026646|ref|NM_001177576|ens|ENSMUST00000106007|ens|ENSMUST00000124266 | Slc25a22 | 2.49 |
| A_55_P2139069 | ref|NM_001039485 | Fam38b | 2.49 |
| A_30_P01027727 | Unknown | chr1:167219841-167220505_F | 2.48 |
| A_55_P1960423 | ens|ENSMUST00000060793|gb|AK018868|gb|AK018877|gb|AK006684 | ENSMUST00000060793 | 2.48 |
| A_55_P1980671 | ref|NM_001011743|ens|ENSMUST00000054683|gb|BC147575|gb|BC147579 | Olfr324 | 2.48 |
| A_30_P01028098 | Unknown | chr12:45415565-45423519_F | 2.48 |
| A_55_P2279927 | gb|AK148051|gb|CJ265080|riken|G370004A16 | 5930430L01Rik | 2.48 |
| A_55_P1994534 | ens|ENSMUST00000052189|ref|XR_035255|ref|XR_035287|gb|AK050074 | ENSMUST00000052189 | 2.48 |
| A_55_P2094262 | ref|NM_009414|ref|NM_001136084|ens|ENSMUST00000049298|ens|ENSMUST00000107669 | Tph1 | 2.48 |
| A_55_P2144132 | ref|XM_001472163|ref|XM_001476105 | Gm16506 | 2.48 |
| A_55_P2137927 | ref|NM_001136055|ref|NM_007656|ens|ENSMUST00000111257|ens|ENSMUST00000028644 | Cd82 | 2.48 |
| A_55_P2125200 | ref|NM_001042670|ens|ENSMUST00000065462|gb|BC141337|gb|AK016498 | Gm9897 | 2.48 |
| A_55_P2081030 | ref|NM_001101516|ens|ENSMUST00000086395|tc|TC1609240|nap|NAP030675-1 | Gpr25 | 2.48 |
| A_55_P2164659 | ref|NM_009392|ens|ENSMUST00000089641|gb|AK013072|tc|TC1691599 | Tlx2 | 2.46 |
| A_30_P01031797 | Unknown | chr6:12072440-12075161_R | 2.46 |
| A_51_P262325 | ref|NM_172461|ens|ENSMUST00000038648|gb|AK030042|tc|TC1589085 | Nek11 | 2.46 |
| A_55_P2140843 | ref|XR_034250 | Gm4157 | 2.46 |
| A_55_P1991054 | ref|NM_001081111|ens|ENSMUST00000095664|ens|ENSMUST00000095665|ens|ENSMUST00000124173 | Tmf1 | 2.46 |
| A_30_P01019105 | Unknown | chr8:48480224-48480698_R | 2.46 |
| A_51_P515462 | ref|NM_146725|ens|ENSMUST00000071410|gb|BC114636|gb|BC146354 | Olfr516 | 2.46 |
| A_51_P247799 | ref|NM_009812|ref|NM_001080126|ens|ENSMUST00000027189|gb|AJ000641 | Casp8 | 2.45 |
| A_51_P439996 | ref|NM_008148|ens|ENSMUST00000061190|gb|BC156183|gb|BC156923 | Gp5 | 2.45 |
| A_55_P2025635 | ref|XM_918513 | LOC641170 | 2.45 |
| A_51_P132400 | ref|NM_013707|gb|AF003691|gb|BC104262|gb|BC104263 | Krtap14 | 2.45 |
| A_51_P271417 | ref|NM_178887|ens|ENSMUST00000028188|gb|AK046338|gb|BC060634 | Fibcd1 | 2.44 |
| A_55_P2025173 | ref|NM_024277|ref|NM_001033865|ens|ENSMUST00000102845|tc|TC1673833 | Rps27a | 2.44 |
| A_55_P1970309 | ref|NM_175003|ref|NM_001099288|ens|ENSMUST00000130420|ens|ENSMUST00000093003 | AU040829 | 2.44 |
| A_55_P2232023 | gb|BB756663 | AI448005 | 2.44 |
| A_30_P01024887 | Unknown | chr18:47329242-47329439_R | 2.44 |
| A_55_P2128860 | Unknown | A_55_P2128860 | 2.44 |
| A_66_P101903 | ref|NM_198617|ens|ENSMUST00000099194|ens|ENSMUST00000103153|gb|AK167722 | Tspyl3 | 2.44 |
| A_55_P1973950 | ref|NR_033444|ens|ENSMUST00000088018|gb|BC160264|gb|BC145633 | Krt74 | 2.44 |
| A_55_P2063583 | gb|BC089325|gb|BC021590|tc|TC1604719|tc|TC1595281 | Tomm6 | 2.43 |
| A_55_P1986356 | ens|ENSMUST00000099739|gb|AK141206|tc|TC1589134|riken|C330006K03 | D630037F22Rik | 2.43 |
| A_55_P2152862 | ens|ENSMUST00000110241|gb|AK047093|tc|TC1595091|riken|B930018M20 | ENSMUST00000110241 | 2.43 |
| A_55_P1954608 | ref|NM_001037923|ens|ENSMUST00000099075|gb|AK137452|tc|TC1584986 | Lekr1 | 2.43 |
| A_30_P01025065 | Unknown | chr13:98221098-98480773_F | 2.43 |
| A_52_P254825 | ref|NM_133711|ens|ENSMUST00000033917|gb|AF530515|gb|AK005612 | Spata4 | 2.43 |
| A_55_P2074165 | ref|XM_977410|ref|XM_977583 | XM_977410 | 2.43 |
| A_51_P442023 | ref|NM_010623|ens|ENSMUST00000030539|gb|BC140373|gb|BC148703 | Kif17 | 2.43 |
| A_55_P2128443 | ens|ENSMUST00000098753 | ENSMUST00000098753 | 2.43 |
| A_55_P2164055 | ref|NM_001110506|ens|ENSMUST00000032468|gb|BC060267|tc|TC1584269 | BC060267 | 2.42 |
| A_52_P51024 | ref|NM_010304|ens|ENSMUST00000043709|gb|M80632|gb|AK010411 | Gna15 | 2.42 |
| A_30_P01024417 | Unknown | chr18:83087114-83122014_R | 2.42 |
| A_30_P01024696 | Unknown | chr17:10517466-10522172_F | 2.42 |
| A_55_P2078040 | ref|NM_001042528|ref|NM_007579|ens|ENSMUST00000114442|ens|ENSMUST00000133892 | Cacna1b | 2.42 |
| A_55_P2375194 | gb|AK019679|tc|TC1608517|riken|4930513E20 | 4930513E20Rik | 2.42 |
| A_66_P111301 | ref|NM_025479|ref|NM_001177513|ens|ENSMUST00000049744|gb|AK012770 | 2810021B07Rik | 2.42 |
| A_55_P1999625 | ref|XM_909616|ref|XM_001478684 | LOC634780 | 2.42 |
| A_52_P231292 | ref|NM_010008|ens|ENSMUST00000030303|gb|AK078893|tc|TC1623536 | Cyp2j6 | 2.42 |
| A_55_P2070401 | ref|NM_029432|ens|ENSMUST00000120316|ens|ENSMUST00000110246|ens|ENSMUST00000110247 | 4930402H24Rik | 2.41 |
| A_52_P254149 | ref|NM_153134|ens|ENSMUST00000049020|gb|BC056347|gb|BC080294 | Irgq | 2.41 |
| A_51_P479548 | ref|NM_175495|ens|ENSMUST00000056130|gb|AK047609|tc|TC1588687 | Gpr150 | 2.41 |
| A_55_P2027900 | ref|XM_001478553|gb|AK046953|gb|CK621530|tc|TC1592597 | LOC100047443 | 2.41 |
| A_55_P2025340 | ref|NM_146395|ens|ENSMUST00000111008|ens|ENSMUST00000073322|tc|NP830861 | Olfr1276 | 2.41 |
| A_51_P468020 | ref|NM_026648|ens|ENSMUST00000093100|gb|AK019633|tc|TC1582249 | Lrrc50 | 2.41 |
| A_55_P2065325 | ref|NM_028014|ens|ENSMUST00000103034|ens|ENSMUST00000103033|ens|ENSMUST00000093912 | 2310067B10Rik | 2.40 |
| A_30_P01029724 | Unknown | chr1:138520504-138521227_R | 2.40 |
| A_55_P2098940 | ref|XM_001471969 | 1700110I01Rik | 2.40 |
| A_55_P2363040 | gb|AK005727|riken|1700007J24 | 1700007J24Rik | 2.40 |
| A_30_P01032551 | Unknown | chr1:163508244-163586072_R | 2.40 |
| A_55_P1957282 | ens|ENSMUST00000059032|gb|BC006743|gb|BE569287|tc|TC1593798 | ENSMUST00000059032 | 2.40 |
| A_55_P2136757 | ref|NM_025996|ens|ENSMUST00000018466|ens|ENSMUST00000109384|gb|AK144450 | Tomm34 | 2.39 |
| A_30_P01033353 | Unknown | chr13:67932042-67944229_F | 2.39 |
| A_55_P2061874 | ref|NM_025596|ens|ENSMUST00000021942|ref|XR_032554|ref|XR_034300 | Prelid1 | 2.39 |
| A_30_P01031432 | Unknown | chr13:108548345-108679004_R | 2.39 |
| A_55_P1999162 | ens|ENSMUST00000115934|ens|ENSMUST00000113394|ref|XM_001475751|ref|XM_001472101 | ENSMUST00000115934 | 2.39 |
| A_66_P101538 | ref|NM_025730|ens|ENSMUST00000060642|gb|AK050702|tc|TC1597475 | Lrrk2 | 2.39 |
| A_52_P82701 | ref|NM_001081060|ens|ENSMUST00000036208|gb|AF139194|gb|BC152921 | Slc9a3 | 2.39 |
| A_66_P137410 | ref|NM_001039231|ens|ENSMUST00000071978|gb|AK132470|tc|TC1594230 | C230055K05Rik | 2.39 |
| A_51_P459908 | ref|NM_010999|ens|ENSMUST00000102785|ens|ENSMUST00000056759|gb|AF102533 | Olfr56 | 2.39 |
| A_30_P01023935 | Unknown | chr11:95483973-95490751_F | 2.39 |
| A_55_P1968153 | ref|NM_009545|ref|NM_001163307|ref|NM_001163308|ens|ENSMUST00000018681 | Pcgf2 | 2.39 |
| A_51_P225592 | ref|NM_001001491|ens|ENSMUST00000003575|gb|AK198949|gb|BC070421 | Tpm4 | 2.39 |
| A_55_P2078325 | ref|NM_001039558|ens|ENSMUST00000068293|gb|AK045581|tc|TC1587560 | C030030A07Rik | 2.39 |
| A_30_P01017903 | Unknown | chr15:100905367-100905654_R | 2.39 |
| A_55_P2207220 | gb|AK034247|riken|9330168G06 | Gm10065 | 2.39 |
| A_55_P2394620 | gb|AK020395|tc|TC1626345|riken|9330198N18|nap|NAP078345-1 | 9330198N18Rik | 2.39 |
| A_55_P2137596 | ref|NM_029935|gb|AK082318|gb|AK083614|gb|AK031995 | Chst15 | 2.39 |
| A_51_P155152 | ref|NM_020332|ens|ENSMUST00000022875|gb|AF274752|gb|AK086014 | Ank | 2.39 |
| A_52_P301495 | ref|NM_009497|ens|ENSMUST00000021273|gb|AK090178|gb|BC039745 | Vamp2 | 2.38 |
| A_52_P290221 | ref|NM_001111076|ref|NM_001013387|gb|AK032094|gb|AK048587 | Zfp182 | 2.38 |
| A_52_P84234 | ens|ENSMUST00000124556|ens|ENSMUST00000025897|gb|AK154323|gb|AK133541 | ENSMUST00000124556 | 2.38 |
| A_52_P284927 | ens|ENSMUST00000114005|ens|ENSMUST00000114004|gb|BC027257|gb|AK034046 | ENSMUST00000114005 | 2.38 |
| A_30_P01025550 | Unknown | chr4:130039725-130069550_F | 2.38 |
| A_55_P1971647 | ref|NM_028705|ens|ENSMUST00000031823|ens|ENSMUST00000114286|ens|ENSMUST00000041401 | Herc3 | 2.38 |
| A_55_P2036290 | ens|ENSMUST00000101129 | ENSMUST00000101129 | 2.38 |
| A_55_P2101357 | ref|NM_181411|ens|ENSMUST00000094376|ens|ENSMUST00000035350|ens|ENSMUST00000146722 | Aftph | 2.38 |
| A_52_P334796 | ref|NM_001008499|ens|ENSMUST00000092660|gb|AY255547|gb|BC117729 | Taar4 | 2.38 |
| A_55_P2101541 | ens|ENSMUST00000050123|ref|XM_001471995|gb|AK030224|tc|TC1593469 | ENSMUST00000050123 | 2.38 |
| A_30_P01020155 | Unknown | chr7:140018492-140019064_R | 2.38 |
| A_55_P2066563 | ens|ENSMUST00000009791|gb|BC139254|gb|BC139257|gb|AK161322 | ENSMUST00000009791 | 2.38 |
| A_30_P01023011 | Unknown | chr16:4867322-4874772_R | 2.38 |
| A_55_P2174524 | ref|XM_001475997 | LOC100046165 | 2.37 |
| A_51_P420859 | ref|NM_011773|ens|ENSMUST00000031037|gb|AK144568|gb|U76007 | Slc30a3 | 2.37 |
| A_55_P2028228 | ref|XM_001473094|ref|XM_001475794 | Gm12827 | 2.37 |
| A_66_P140574 | ref|XM_001477066|gb|AK043526|riken|A830005H01|nap|NAP082486-1 | 6530403H02Rik | 2.37 |
| A_30_P01032122 | Unknown | chrX:148803114-148851243_F | 2.37 |
| A_55_P2132993 | ref|XR_030763|gb|AK166824|gb|AK145477|gb|AK166784 | LOC100045342 | 2.37 |
| A_55_P1959213 | ens|ENSMUST00000089614|gb|AK140159|riken|B230320C11 | ENSMUST00000089614 | 2.37 |
| A_55_P2306893 | gb|AK163891|tc|TC1723052|riken|C230022K22 | 4931406E20Rik | 2.36 |
| A_55_P2081398 | ref|XM_001473767|gb|AK041390|tc|TC1617004|riken|A630006J12 | LOC100045282 | 2.36 |
| A_66_P108796 | ref|NM_175651|ens|ENSMUST00000120068|gb|AK035828|tc|TC1590940 | Cnpy1 | 2.36 |
| A_30_P01018673 | Unknown | chr2:80338640-80339980_R | 2.36 |
| A_55_P2151438 | ref|XR_030778 | Gm16410 | 2.36 |
| A_55_P2065113 | ref|NM_001177484|ens|ENSMUST00000107436|ens|ENSMUST00000092694|gb|AK017437 | Gm11559 | 2.36 |
| A_66_P122433 | ref|NM_029107|ens|ENSMUST00000111154|gb|AK029492|gb|AK133272 | 4930417G10Rik | 2.36 |
| A_55_P2006210 | ens|ENSMUST00000020577|gb|AK008922|tc|TC1604725|riken|2210414E06 | Fgf22 | 2.36 |
| A_66_P104751 | ref|XM_488798|gb|AK019108|tc|TC1594615|riken|2410011O22 | LOC433068 | 2.36 |
| A_55_P2116621 | ref|NM_010828|ens|ENSMUST00000038107|gb|AK131664|gb|AK133791 | Cited2 | 2.36 |
| A_55_P2007339 | ens|ENSMUST00000105022|nap|NAP068828-1 | ENSMUST00000105022 | 2.36 |
| A_55_P1982744 | ref|XR_031319|ref|XR_034484|ref|XR_031690|ref|XR_034139 | Gm12118 | 2.36 |
| A_66_P118906 | ref|NM_007413|ens|ENSMUST00000018644|ref|XM_001473907|gb|BC116415 | Adora2b | 2.36 |
| A_30_P01023567 | Unknown | chr8:123571640-123607481_R | 2.36 |
| A_55_P2232297 | ref|NM_177164|ens|ENSMUST00000053922|ens|ENSMUST00000113937|gb|AK080585 | Vwc2l | 2.36 |
| A_55_P1976634 | ref|NM_146312|ref|NM_001011858|ens|ENSMUST00000098171|ens|ENSMUST00000075595 | Olfr657 | 2.35 |
| A_55_P2060323 | ens|ENSMUST00000118559|tc|TC1678819 | ENSMUST00000118559 | 2.35 |
| A_52_P30920 | ref|NM_013761|ref|NM_001163311|ens|ENSMUST00000138612|ens|ENSMUST00000123855 | Srr | 2.35 |
| A_55_P1961700 | ens|ENSMUST00000027905|ens|ENSMUST00000127077|gb|DQ463440|tc|TC1698426 | ENSMUST00000027905 | 2.35 |
| A_66_P111433 | ref|XR_002105|ref|XR_031204 | Gm6027 | 2.35 |
| A_52_P26086 | ref|NM_146958|ens|ENSMUST00000079891|tc|TC1599945|nap|NAP099168-001 | Olfr171 | 2.35 |
| A_51_P395050 | ref|NM_199306|ens|ENSMUST00000105906|ens|ENSMUST00000043305|ref|XM_001478861 | Wdtc1 | 2.35 |
| A_66_P105801 | ref|NM_010513|ens|ENSMUST00000005671|gb|BB079945|tc|TC1707039 | Igf1r | 2.35 |
| A_30_P01027240 | Unknown | chr10:92798725-92811622_F | 2.35 |
| A_55_P2164433 | ref|NM_139152|ens|ENSMUST00000086882|gb|AY057054|gb|BC167192 | Asb18 | 2.35 |
| A_55_P1991598 | ens|ENSMUST00000106723|gb|BC025441|tc|NP437391 | ENSMUST00000106723 | 2.35 |
| A_30_P01018853 | Unknown | chr4:44197185-44197997_R | 2.34 |
| A_52_P514107 | ref|NM_001038592|ref|NM_001038593|ref|NM_023505|ref|NM_001038594 | Glrx2 | 2.34 |
| A_30_P01030487 | Unknown | chr12:15735906-15798506_F | 2.34 |
| A_55_P1984487 | ref|NM_007418|ens|ENSMUST00000049545|ref|XM_001475557|tc|TC1586475 | Adra2c | 2.34 |
| A_30_P01033055 | Unknown | chr2:174897225-174920850_R | 2.34 |
| A_30_P01023646 | Unknown | chr11:102983499-102994170_R | 2.34 |
| A_55_P2257341 | gb|AK010390|tc|TC1653012|riken|2410004I01|nap|NAP090792-1 | 2410004I01Rik | 2.34 |
| A_55_P2061640 | ens|ENSMUST00000108308|gb|BC005523|tc|TC1721898|nap|NAP110482-1 | ENSMUST00000108308 | 2.34 |
| A_52_P474636 | ref|NM_021420|ens|ENSMUST00000109382|ens|ENSMUST00000018353|gb|AF271360 | Stk4 | 2.34 |
| A_55_P2160181 | ref|NM_145744|ens|ENSMUST00000037715|gb|AF357887|nap|NAP057805-1 | Dusp15 | 2.34 |
| A_51_P378511 | ref|NM_153123|ens|ENSMUST00000044005|gb|AK145634|gb|BC018510 | Atf7ip2 | 2.34 |
| A_55_P1955202 | ens|ENSMUST00000115023|gb|AK145592|tc|TC1716614|riken|I1C0020A07 | ENSMUST00000115023 | 2.34 |
| A_66_P132834 | ref|NM_027602|ens|ENSMUST00000031109|ens|ENSMUST00000131585|ref|XM_001478324 | Nsun7 | 2.33 |
| A_52_P534749 | ref|NM_008719|ens|ENSMUST00000056815|gb|U77969|gb|AK075824 | Npas2 | 2.33 |
| A_51_P396385 | ref|NM_175439|ens|ENSMUST00000061334|gb|BC132343|gb|BC137739 | Mars2 | 2.33 |
| A_66_P133525 | ref|XM_001478645|ref|XM_001479808|gb|AK082053|tc|NP718970 | XM_001478645 | 2.33 |
| A_52_P340073 | ref|NM_010111|ens|ENSMUST00000001319|gb|BC057009|gb|AK134108 | Efnb2 | 2.33 |
| A_55_P2184641 | ref|XM_001476131|ref|XM_001478432 | 2810011L19Rik | 2.33 |
| A_51_P411045 | ens|ENSMUST00000049565|gb|AK035647|gb|AK079280|tc|TC1628497 | ENSMUST00000049565 | 2.33 |
| A_55_P2001269 | ens|ENSMUST00000103522|ens|ENSMUST00000103531|ens|ENSMUST00000103543|ens|ENSMUST00000103521 | ENSMUST00000103522 | 2.33 |
| A_30_P01019222 | Unknown | chr3:85348237-85358728_R | 2.33 |
| A_55_P2062495 | ref|NM_009762|ref|NM_001160127|ens|ENSMUST00000068289|ens|ENSMUST00000114186 | Smyd1 | 2.33 |
| A_30_P01018627 | Unknown | chr1:63163023-63180223_R | 2.33 |
| A_51_P286488 | ref|NM_133662|ens|ENSMUST00000003635|gb|AK203877|gb|AK170477 | Ier3 | 2.33 |
| A_51_P104710 | ref|NM_173428|ens|ENSMUST00000043676|gb|AJ491857|gb|AK142699 | Sspo | 2.33 |
| A_55_P2339961 | gb|AK133430|gb|AV208205|riken|4933428H08 | 1700010B13Rik | 2.33 |
| A_55_P2004821 | ref|NM_146936|ens|ENSMUST00000061235|gb|BC153816|gb|BC148189 | Olfr1417 | 2.32 |
| A_55_P1989215 | ref|NM_028093|ens|ENSMUST00000044078|ens|ENSMUST00000114380|ens|ENSMUST00000114376 | Entpd8 | 2.32 |
| A_55_P2039617 | ens|ENSMUST00000067929|gb|AK033674|tc|TC1594374|riken|9130217P20 | ENSMUST00000067929 | 2.31 |
| A_55_P1956752 | ref|XM_001479792|ref|XM_001480213|ref|XM_890112|ref|XM_916146 | Ska2l-ps | 2.31 |
| A_52_P488361 | ref|NM_028839|ens|ENSMUST00000006701|gb|AK009341|gb|AK007740 | Tmem110 | 2.31 |
| A_55_P1957993 | ref|XM_001475570 | LOC100046123 | 2.31 |
| A_55_P2194054 | gb|AK021077|tc|TC1614394|riken|C030014A21 | C030014A21Rik | 2.31 |
| A_30_P01024972 | Unknown | chr17:3198868-3394643_R | 2.31 |
| A_55_P2003596 | ref|XM_001472384|tc|TC1681662 | LOC100048880 | 2.31 |
| A_51_P469285 | ref|NM_008737|ens|ENSMUST00000026917|gb|D50086|gb|AK159219 | Nrp1 | 2.30 |
| A_52_P449629 | ref|NM_001013806|ens|ENSMUST00000040787|gb|BC138991|gb|BC138992 | Ankrd13c | 2.30 |
| A_55_P2071107 | ens|ENSMUST00000109251|gb|BC115767 | ENSMUST00000109251 | 2.30 |
| A_30_P01024112 | Unknown | chr11:18753941-18760118_R | 2.30 |
| A_55_P2020601 | ref|XR_031461|ref|XR_031734|ref|XR_034279 | Gm9713 | 2.30 |
| A_55_P2014427 | ref|NM_001034031|ref|NM_001034029|ref|NM_145826|ens|ENSMUST00000113073 | Il17re | 2.30 |
| A_30_P01031283 | Unknown | chr3:145269449-145298239_F | 2.30 |
| A_51_P210350 | ref|NM_182959|ens|ENSMUST00000099372|ens|ENSMUST00000105295|ens|ENSMUST00000020102 | Slc17a8 | 2.30 |
| A_66_P136097 | ref|NM_001164220|ref|NM_023233|ens|ENSMUST00000039562|gb|BC138576 | Trim13 | 2.30 |
| A_51_P329322 | ref|NM_025512|ens|ENSMUST00000037839|gb|AK030472|gb|AK009240 | Zfand1 | 2.30 |
| A_52_P260555 | ref|NM_009509|ens|ENSMUST00000027366|gb|M98454|gb|AK027908 | Vil1 | 2.30 |
| A_51_P288010 | ref|NM_009604|gb|BC156729|gb|X03818|gb|M30514 | Chrng | 2.30 |
| A_55_P2224830 | gb|AK052079|riken|D230044B12 | D230044B12Rik | 2.29 |
| A_30_P01020163 | Unknown | chrX:157038750-157039018_F | 2.29 |
| A_30_P01029490 | Unknown | chr12:55844075-55898175_R | 2.29 |
| A_55_P2294074 | gb|CB196702|tc|TC1659663 | AA536887 | 2.29 |
| A_30_P01021254 | Unknown | chr4:33375464-33377138_R | 2.29 |
| A_55_P1957957 | ref|NM_027883|ens|ENSMUST00000094816|ens|ENSMUST00000119102|ens|ENSMUST00000118795 | Dhx34 | 2.29 |
| A_51_P461452 | ref|NM_026275|ens|ENSMUST00000040008|gb|AK007517|gb|AK003550 | Ube2r2 | 2.29 |
| A_55_P2104219 | ref|NM_010464|ens|ENSMUST00000001700|gb|AF193796|tc|TC1591398 | Hoxc13 | 2.29 |
| A_52_P368134 | ref|NM_026149|ref|NM_001113554|ens|ENSMUST00000038719|gb|AK014996 | Nudcd1 | 2.29 |
| A_55_P2090874 | ref|NM_011737|nap|NAP058716-1 | Ysk4 | 2.29 |
| A_55_P2085731 | ref|NM_134115|ens|ENSMUST00000119274|ens|ENSMUST00000009138|gb|AY292399 | Stk38 | 2.29 |
| A_55_P2041086 | ref|NM_001114102|tc|TC1662746 | 1110034B05Rik | 2.29 |
| A_30_P01026617 | Unknown | chr4:147108244-147183905_R | 2.29 |
| A_30_P01019862 | Unknown | chr7:68849401-68958266_R | 2.29 |
| A_55_P2020026 | ref|XM_001479264|ref|XM_001472276 | 4930439D14Rik | 2.29 |
| A_30_P01031997 | Unknown | chr9:88437245-88465751_R | 2.29 |
| A_51_P179480 | ref|NM_172450|ens|ENSMUST00000062357|gb|AK033585|tc|TC1586421 | 4930539E08Rik | 2.28 |
| A_55_P1983883 | ref|NM_033560|ens|ENSMUST00000098817|tc|TC1656249 | Vps37a | 2.28 |
| A_55_P2200780 | ref|NR_026798|gb|AK132930|riken|4930521G09|nap|NAP014220-001 | Zfp389 | 2.28 |
| A_30_P01028285 | Unknown | chr17:46972160-46997919_R | 2.28 |
| A_30_P01025569 | Unknown | chr11:69106907-69112857_R | 2.28 |
| A_55_P2027230 | ref|XR_031437|ref|XR_031485 | Gm7105 | 2.28 |
| A_55_P1963878 | ref|XM_484599|ref|XM_913581 | Gm5492 | 2.28 |
| A_30_P01023679 | Unknown | chr3:38324807-38342556_R | 2.28 |
| A_30_P01020601 | Unknown | chr13:78370478-78371404_F | 2.28 |
| A_55_P2357581 | gb|AK047256|tc|TC1597928|riken|B930042K01 | B930042K01Rik | 2.27 |
| A_55_P2100025 | ref|NM_001001447|ens|ENSMUST00000120809|ens|ENSMUST00000055528|ens|ENSMUST00000119989 | Zscan22 | 2.27 |
| A_55_P2026848 | ref|NM_001079932|ens|ENSMUST00000081042|ens|ENSMUST00000106248|gb|AB231474 | Trim72 | 2.27 |
| A_51_P487027 | ref|NM_010607|ref|NM_001159850|ref|NR_027627|ens|ENSMUST00000110920 | Kcnk2 | 2.27 |
| A_66_P124445 | ref|NM_029623|ens|ENSMUST00000025276|gb|AK154240|gb|AK159691 | 3110002H16Rik | 2.27 |
| A_55_P2166733 | ref|XM_001472838|nap|NAP059265-1 | LOC100044685 | 2.27 |
| A_55_P1975690 | ref|NM_031397|ens|ENSMUST00000014473|ens|ENSMUST00000105434|ens|ENSMUST00000131445 | Bicc1 | 2.27 |
| A_55_P2094955 | ens|ENSMUST00000100068|ens|ENSMUST00000044306|gb|AK140273|tc|TC1638890 | ENSMUST00000100068 | 2.27 |
| A_52_P676359 | ref|NM_010190|ens|ENSMUST00000028179|gb|AF063217|gb|BC107220 | Fcnb | 2.27 |
| A_55_P2073030 | ref|XM_001473833 | LOC100045195 | 2.27 |
| A_30_P01022638 | Unknown | chr5:125205454-125209470_F | 2.27 |
| A_51_P521090 | ens|ENSMUST00000044964|gb|AK144870|gb|AK008838|tc|TC1612220 | ENSMUST00000044964 | 2.27 |
| A_55_P2387915 | ens|ENSMUST00000099693|gb|AK143789|riken|F430108K24 | ENSMUST00000099693 | 2.26 |
| A_52_P417978 | ref|NM_027510|ref|NM_001100609|ref|NM_001102678|ref|NM_009529 | 3830403N18Rik | 2.26 |
| A_30_P01030381 | Unknown | chr17:88225376-88238756_F | 2.26 |
| A_52_P164286 | ref|NM_001038619|ref|NM_172646|ens|ENSMUST00000086074|ens|ENSMUST00000070330 | Dnm3 | 2.26 |
| A_55_P2049252 | ref|XM_001479242 | Mapkapk5 | 2.26 |
| A_55_P2111832 | ens|ENSMUST00000121047 | ENSMUST00000121047 | 2.26 |
| A_55_P2181216 | ref|XR_031691|ref|XR_031947 | Gm5628 | 2.26 |
| A_51_P291839 | ref|NM_183256|ens|ENSMUST00000023761|gb|BC092142|gb|AK009401 | 2310016M24Rik | 2.26 |
| A_55_P2122200 | ref|NM_011158|ens|ENSMUST00000036497|gb|AK041013|gb|AK138963 | Prkar2b | 2.26 |
| A_30_P01023771 | Unknown | chr9:58197113-58200614_F | 2.26 |
| A_55_P2015074 | ref|NM_029415|ens|ENSMUST00000031263|ref|XM_001474618|gb|AJ583504 | Slc10a6 | 2.26 |
| A_51_P424290 | ref|NM_181818|ens|ENSMUST00000105212|gb|BC148499|gb|BC153102 | Olfr141 | 2.25 |
| A_30_P01017452 | Unknown | chr3:30064327-30064680_R | 2.25 |
| A_66_P128942 | ref|XM_001480382|gb|AK038479|gb|BC144798|gb|BC145711 | Mipep | 2.25 |
| A_55_P2147265 | ens|ENSMUST00000100438 | ENSMUST00000100438 | 2.25 |
| A_52_P45841 | ref|NM_028976|gb|BC012251|gb|AK017293|tc|TC1575497 | Gorasp1 | 2.25 |
| A_55_P2112529 | ref|XM_001476088|ref|XM_001474224 | Gm3247 | 2.25 |
| A_55_P2244871 | gb|AI574175 | AI574175 | 2.25 |
| A_51_P120254 | ref|NM_015821|ens|ENSMUST00000126923|ens|ENSMUST00000036221|gb|AK053206 | Fbxl8 | 2.25 |
| A_55_P1968743 | ref|NM_008689|ens|ENSMUST00000106275|ens|ENSMUST00000106276|ens|ENSMUST00000132668 | Nfkb1 | 2.25 |
| A_55_P2099329 | ens|ENSMUST00000094465 | ENSMUST00000094465 | 2.25 |
| A_55_P2032960 | ref|NM_028227|ens|ENSMUST00000111765|ens|ENSMUST00000140996|ens|ENSMUST00000031414 | Brap | 2.24 |
| A_55_P2237843 | gb|AK042831|tc|TC1597909|riken|A730028G07 | A730028G07Rik | 2.24 |
| A_52_P247559 | ref|NM_001142570|ens|ENSMUST00000113382|gb|BC059233|gb|AK051529 | Dach2 | 2.24 |
| A_55_P2175454 | ref|XM_001478438 | LOC100047580 | 2.24 |
| A_30_P01025104 | Unknown | chr19:32904115-32904397_R | 2.24 |
| A_55_P2033278 | ref|NR_001582|ref|NR_027506|ref|XM_001474043|ref|XM_001477479 | Speer5-ps1 | 2.24 |
| A_52_P516021 | ref|NM_011201|ens|ENSMUST00000029053|gb|AK211808|gb|AK085713 | Ptpn1 | 2.24 |
| A_51_P221256 | ref|NM_011402|ens|ENSMUST00000094787|gb|AF081499|gb|AK145599 | Slc34a2 | 2.24 |
| A_52_P497625 | ref|NM_177055|ens|ENSMUST00000064788|gb|AK041324|gb|AK139572 | A630001G21Rik | 2.24 |
| A_55_P2100118 | ens|ENSMUST00000107844|tc|TC1676895 | ENSMUST00000107844 | 2.24 |
| A_55_P2037873 | ref|XM_001472485|ref|XM_001472290 | Gm10322 | 2.24 |
| A_55_P1989956 | ref|NM_001081298|ens|ENSMUST00000106128|ens|ENSMUST00000106127|ens|ENSMUST00000037655 | Lphn2 | 2.23 |
| A_55_P2083919 | ref|NM_175549|ens|ENSMUST00000117200|ens|ENSMUST00000114262|ens|ENSMUST00000117785 | Robo2 | 2.23 |
| A_55_P1979986 | ref|NM_007761|ens|ENSMUST00000111301|ens|ENSMUST00000026608|gb|AF028242 | Crcp | 2.23 |
| A_55_P2012478 | ref|NM_028326|ens|ENSMUST00000064814|gb|AK173309|gb|AK012894 | Zfp618 | 2.23 |
| A_55_P2074688 | ref|NM_013553|ens|ENSMUST00000100164|gb|D11329|tc|TC1647762 | Hoxc4 | 2.23 |
| A_30_P01024003 | Unknown | chr7:6108350-6119600_R | 2.23 |
| A_55_P2004562 | ref|NM_001170851|ref|NM_008462|ens|ENSMUST00000088867|ens|ENSMUST00000032306 | Klra2 | 2.23 |
| A_52_P629333 | ref|NM_145608|ens|ENSMUST00000034316|gb|AK220424|gb|AK136437 | BC021891 | 2.23 |
| A_30_P01026976 | Unknown | chr17:84397891-84412748_F | 2.23 |
| A_55_P2046605 | ref|NR_015521|gb|AK006529|gb|BC100365|tc|TC1618781 | 1700030C10Rik | 2.23 |
| A_55_P2180156 | ref|XM_001479936 | LOC100048288 | 2.23 |
| A_55_P2031272 | ref|NM_001039653|ens|ENSMUST00000054099|ens|ENSMUST00000028302|gb|L38248 | Lhx3 | 2.23 |
| A_52_P408230 | ens|ENSMUST00000112452|ens|ENSMUST00000112454|gb|AK052366|gb|AK034003 | ENSMUST00000112452 | 2.23 |
| A_55_P2138217 | ref|NM_176965|ens|ENSMUST00000108399|ens|ENSMUST00000060491|ens|ENSMUST00000130901 | Efcab5 | 2.23 |
| A_55_P2040921 | ref|XR_033752|tc|TC1702148|nap|NAP065213-1 | LOC100047121 | 2.23 |
| A_55_P2061779 | ref|NM_001001186|ens|ENSMUST00000057070|gb|AK164227|riken|D030021C08 | Zfp456 | 2.23 |
| A_55_P2157799 | ref|NM_032394|ens|ENSMUST00000025251|ens|ENSMUST00000134663|gb|BC138341 | Myo7b | 2.22 |
| A_52_P284821 | ref|NM_172658|gb|BC119022|gb|AK049253|gb|AY654588 | Slco4c1 | 2.22 |
| A_55_P2180810 | ref|XM_148801|ref|XM_914449|gb|BC172076|tc|TC1654687 | Prr22 | 2.22 |
| A_30_P01030864 | Unknown | chr5:110424250-110443025_F | 2.22 |
| A_30_P01020403 | Unknown | chr13:98038568-98068079_R | 2.22 |
| A_55_P2130627 | ref|NM_001099917|ens|ENSMUST00000118206|ens|ENSMUST00000105322|gb|BC117027 | 2210404O07Rik | 2.22 |
| A_66_P119350 | ref|XM_001479091|ref|XM_001480154|gb|AK009724|gb|BC070414 | 2310040G24Rik | 2.22 |
| A_55_P2184741 | ref|NM_153572|ens|ENSMUST00000110509|ens|ENSMUST00000047257|gb|BC030434 | Katnal1 | 2.22 |
| A_30_P01028439 | Unknown | chr8:26866152-26872502_F | 2.22 |
| A_30_P01031237 | Unknown | chr16:40470294-40470935_F | 2.21 |
| A_55_P2161030 | ref|NM_172426|ref|NM_001110240|ens|ENSMUST00000044990|ens|ENSMUST00000107155 | Slc24a2 | 2.21 |
| A_52_P446677 | ref|NM_183163|ens|ENSMUST00000053202|ens|ENSMUST00000106204|gb|BC052650 | Rhbdl2 | 2.21 |
| A_52_P483959 | ref|NM_207233|ens|ENSMUST00000037286|gb|DQ002402|gb|AK144471 | C1ql2 | 2.21 |
| A_51_P513776 | ens|ENSMUST00000103535|ref|XM_918237|gb|AF296427|gb|U09599 | LOC640979 | 2.21 |
| A_55_P2004385 | ref|NM_181417|ens|ENSMUST00000028911|ref|XM_001480791|gb|BC031563 | Csrp2bp | 2.21 |
| A_55_P2387104 | gb|AK015209|tc|TC1618374|riken|4930427C17|nap|NAP074079-1 | 4930471M09Rik | 2.21 |
| A_30_P01028151 | Unknown | chr4:54669012-54940960_R | 2.21 |
| A_55_P2109096 | ens|ENSMUST00000067768|ref|XM_001472329|gb|AK040144|gb|BB635464 | ENSMUST00000067768 | 2.21 |
| A_55_P2089015 | ens|ENSMUST00000023920|gb|AK009779|tc|TC1585276|riken|2310043I08 | Tmem52 | 2.21 |
| A_55_P2018054 | ref|XM_001477608 | LOC100047188 | 2.21 |
| A_55_P2212733 | gb|BG079846|tc|TC1632670 | C80012 | 2.21 |
| A_30_P01029997 | Unknown | chrX:148819227-148844227_R | 2.21 |
| A_55_P2269988 | gb|AK031263|riken|5930438M14|nap|NAP075491-1 | 5930438M14 | 2.20 |
| A_55_P1975177 | ens|ENSMUST00000065913|gb|AK027969|tc|TC1595094|riken|1110050B14 | ENSMUST00000065913 | 2.20 |
| A_51_P377856 | ref|NM_133994|ens|ENSMUST00000001715|gb|AK154161|gb|AK168226 | Gstt3 | 2.20 |
| A_55_P2034311 | ref|XR_032300|nap|NAP111484-1 | Gm6477 | 2.20 |
| A_66_P101930 | ref|NM_025501|ens|ENSMUST00000046234|gb|BC115788|gb|BC115817 | Lce3b | 2.20 |
| A_52_P676947 | ref|NM_021382|ens|ENSMUST00000029822|gb|AK046424|gb|AK031898 | Tacr3 | 2.20 |
| A_55_P2181404 | ens|ENSMUST00000108691|gb|AK082443|tc|TC1598859|riken|C230051G16 | ENSMUST00000108691 | 2.19 |
| A_52_P583050 | ref|NM_181649|gb|AK082537|gb|AK148331|gb|BC092522 | Ccdc75 | 2.19 |
| A_55_P2282830 | gb|AK008708|tc|TC1725007|riken|2210011K15|nap|NAP091165-1 | 2210011K15Rik | 2.19 |
| A_30_P01019315 | Unknown | chr12:86421771-86422222_F | 2.19 |
| A_30_P01022182 | Unknown | chr6:67054389-67054857_F | 2.19 |
| A_51_P202633 | ref|NM_015766|ens|ENSMUST00000003274|gb|AK155903|gb|AK155023 | Ebi3 | 2.19 |
| A_51_P277275 | ref|NM_009065|ens|ENSMUST00000153060|ens|ENSMUST00000082070|gb|AK162953 | Rit2 | 2.19 |
| A_55_P2060532 | ref|NM_001039658|ens|ENSMUST00000151341|gb|AK015732|tc|NP352447 | Mtl5 | 2.19 |
| A_51_P111455 | ref|NM_027432|ens|ENSMUST00000010278|ens|ENSMUST00000130994|ens|ENSMUST00000128005 | Wdr77 | 2.19 |
| A_55_P2008681 | ref|NM_008477|ens|ENSMUST00000111745|ens|ENSMUST00000022391|ens|ENSMUST00000111744 | Ktn1 | 2.19 |
| A_30_P01033517 | Unknown | chr6:108162092-108162886_R | 2.18 |
| A_51_P410681 | ref|NM_018826|ens|ENSMUST00000034184|gb|AK004747|gb|AK213017 | Irx5 | 2.18 |
| A_51_P185259 | ref|NM_010313|ref|NM_138719|ens|ENSMUST00000076889|gb|U69145 | Gnb5 | 2.18 |
| A_55_P1976102 | ref|NM_026244|ens|ENSMUST00000085245|gb|BC117754|gb|BC117755 | Slc39a9 | 2.18 |
| A_30_P01028568 | Unknown | chr16:40592137-40594284_F | 2.18 |
| A_55_P2028365 | ref|NM_001170788|ref|NM_001170789|ref|NM_001033371|ens|ENSMUST00000109355 | Lrrc36 | 2.18 |
| A_55_P2093023 | ref|NM_026858|ens|ENSMUST00000026504|gb|AK010471|gb|BC030630 | Xrcc6bp1 | 2.18 |
| A_51_P183971 | ref|NM_019446|ref|NM_001164186|ens|ENSMUST00000113847|ens|ENSMUST00000113849 | Barhl1 | 2.18 |
| A_52_P327402 | ref|NM_173370|ens|ENSMUST00000031273|gb|AF533367|gb|AK014670 | Cds1 | 2.18 |
| A_51_P208987 | ref|NM_028352|ref|NM_001163746|ens|ENSMUST00000070064|gb|AK028066 | Pgm3 | 2.18 |
| A_30_P01027126 | Unknown | chr12:112223388-112223755_F | 2.18 |
| A_55_P2102429 | ref|NM_175550|ens|ENSMUST00000002063|tc|TC1632803 | Ap4e1 | 2.18 |
| A_55_P2288670 | gb|AK165039|riken|E130314D17 | E130118H10Rik | 2.18 |
| A_55_P2379480 | gb|AK136853|tc|TC1632888|riken|9330142D14|nap|NAP039589-1 | A630065K11Rik | 2.18 |
| A_51_P341349 | ref|NM_024433|ens|ENSMUST00000058030|gb|AK167319|gb|AK005064 | Mtap | 2.18 |
| A_55_P1997465 | ref|NM_027544|ens|ENSMUST00000133257|ens|ENSMUST00000053683|gb|AY141192 | Ggnbp1 | 2.18 |
| A_55_P2062300 | ref|NM_145524|ref|NM_001110512|ens|ENSMUST00000090849|ens|ENSMUST00000149181 | Mettl8 | 2.18 |
| A_55_P1990693 | ref|XM_001473203|ref|XM_001479239|gb|DQ140166 | LOC100044858 | 2.18 |
| A_55_P2075136 | ref|XM_142100 | Gm4992 | 2.17 |
| A_30_P01033497 | Unknown | chr2:181024098-181039073_R | 2.17 |
| A_55_P2241299 | ref|NM_001025613|ref|NM_001025614|ens|ENSMUST00000090785|ens|ENSMUST00000098849 | Otud7b | 2.17 |
| A_55_P2178530 | ref|XM_001474761 | Gm13274 | 2.17 |
| A_51_P432950 | ref|NM_009216|ens|ENSMUST00000044299|ens|ENSMUST00000110671|gb|BC140315 | Sstr1 | 2.17 |
| A_52_P448466 | ref|NM_030561|ens|ENSMUST00000064709|gb|AK030596|gb|BC058575 | BC004004 | 2.17 |
| A_55_P2143202 | ens|ENSMUST00000099509|gb|AK140323|riken|B430112C04 | ENSMUST00000099509 | 2.17 |
| A_55_P2258109 | gb|AK140478|gb|BC037704|tc|TC1630782|riken|B930008M14 | BC037704 | 2.17 |
| A_55_P2032930 | ref|NM_025524|ens|ENSMUST00000092700|gb|AK009410|tc|TC1584551 | Krtap3-3 | 2.17 |
| A_52_P350664 | ref|NM_153781|ens|ENSMUST00000045441|gb|AK142716|gb|AK032769 | Pygb | 2.17 |
| A_52_P108502 | ref|NM_001033270|ens|ENSMUST00000074344|ens|ENSMUST00000057015|gb|AF218295 | Slc4a7 | 2.17 |
| A_52_P15388 | ref|NM_008522|ens|ENSMUST00000035077|gb|CT010339|gb|FJ538998 | Ltf | 2.17 |
| A_52_P37757 | ref|NM_026146|ens|ENSMUST00000086372|gb|AK019490|gb|BC100383 | Eps8l1 | 2.16 |
| DCP_22_9 | Unknown | DCP_22_9 | 2.16 |
| A_52_P194971 | ref|NM_010460|ens|ENSMUST00000049352|gb|X06762|tc|TC1585082 | Hoxb7 | 2.16 |
| A_30_P01023284 | Unknown | chr19:61177675-61219875_F | 2.16 |
| A_55_P2016216 | ref|XM_001480883|ref|XM_001475722 | LOC100048674 | 2.16 |
| A_55_P1953874 | ref|NM_001109743|ref|XM_972803|gb|AB358976 | Skor2 | 2.16 |
| A_30_P01032556 | Unknown | chr8:94841965-94881130_F | 2.16 |
| A_52_P87503 | ref|NM_001033981|ens|ENSMUST00000101387|gb|AK157060|gb|BC141224 | F830116E18Rik | 2.16 |
| A_30_P01026549 | Unknown | chrX:121032646-121038407_R | 2.16 |
| A_52_P208600 | ref|NM_194339|ens|ENSMUST00000032237|gb|AK133013|gb|AK129082 | Bms1 | 2.16 |
| A_52_P383653 | ref|NM_001033851|ens|ENSMUST00000014777|gb|AK005311|gb|BC076564 | Cpne8 | 2.16 |
| A_52_P647408 | ens|ENSMUST00000113891|gb|AK050859|tc|TC1591123|riken|D030026K16 | ENSMUST00000113891 | 2.16 |
| A_51_P470414 | ref|NM_138600|ref|NM_001127338|gb|AK004991|tc|TC1583648 | Aldh7a1 | 2.16 |
| A_55_P1996431 | ref|XR_034101|ref|XR_032658 | Gm9697 | 2.16 |
| A_30_P01026831 | Unknown | chr10:24269165-24276690_R | 2.16 |
| A_55_P1966518 | ref|NM_175730|ens|ENSMUST00000001709|gb|AK078527|tc|TC1584514 | Hoxc5 | 2.15 |
| A_30_P01022073 | Unknown | chr6:83367433-83370986_R | 2.15 |
| A_55_P1981670 | ref|NM_033144|ens|ENSMUST00000120878|ens|ENSMUST00000018542|ens|ENSMUST00000147912 | 8-Sep | 2.15 |
| A_55_P2065751 | ref|NM_010676|ens|ENSMUST00000053460|gb|D86422|gb|BC160311 | Krtap8-2 | 2.15 |
| A_66_P130449 | ref|NM_207270|ens|ENSMUST00000049113|gb|AK078884|gb|AB217856 | Ptprh | 2.15 |
| A_52_P165521 | ref|NM_053081|ref|NM_001163233|ens|ENSMUST00000030165|gb|AY049715 | Fancg | 2.15 |
| A_55_P2038723 | ref|XM_001476035|ref|XM_001474080|gb|AK136780|riken|9330010N06 | LOC100046336 | 2.15 |
| A_55_P2349747 | gb|AK034152|riken|9330159N05|nap|NAP124558-1 | 9330159N05Rik | 2.15 |
| A_30_P01025430 | Unknown | chr18:38478632-38482159_F | 2.15 |
| A_55_P2119478 | ref|NM_028388|ens|ENSMUST00000024909|ens|ENSMUST00000143987|gb|AK150404 | Ndufv2 | 2.15 |
| A_51_P198335 | ref|NM_008068|ref|NM_001099641|ens|ENSMUST00000020703|gb|AK137331 | Gabra6 | 2.15 |
| A_55_P2153872 | ref|NM_026348|ens|ENSMUST00000146258|gb|AK045037|tc|TC1582290 | Itgb3bp | 2.15 |
| A_55_P2095513 | ref|NM_001003405|ens|ENSMUST00000101480|ens|ENSMUST00000064324|gb|BC139223 | Try5 | 2.15 |
| A_55_P2026898 | ref|XM_001478893|nap|NAP114020-1 | Gm4087 | 2.14 |
| A_55_P2103661 | ref|NM_001004149|ens|ENSMUST00000056558|gb|BC070399|tc|TC1592912 | Zfp366 | 2.14 |
| A_55_P2165008 | ref|NM_183297|ens|ENSMUST00000059904|ens|ENSMUST00000095266|gb|BC140288 | Nxph4 | 2.14 |
| A_51_P304859 | ref|NM_022985|ens|ENSMUST00000069537|gb|AK002775|gb|AK144902 | Zfand6 | 2.14 |
| A_30_P01024548 | Unknown | chrX:146945573-146950036_R | 2.14 |
| A_52_P426863 | ref|NM_178643|ens|ENSMUST00000154597|ens|ENSMUST00000032704|gb|AK040248 | C230052I12Rik | 2.14 |
| A_55_P2162712 | ref|NM_173391|ens|ENSMUST00000006949|gb|BC120514|gb|BC125430 | Tph2 | 2.14 |
| A_55_P2073552 | ref|NR_015488|ref|NR_027896|ens|ENSMUST00000100083|gb|AK020802 | A930003A15Rik | 2.14 |
| A_66_P108263 | ref|NM_001164231|ens|ENSMUST00000094294|gb|AK171257|gb|BC044810 | Pwwp2a | 2.14 |
| A_51_P272735 | ref|XM_986599|gb|AK036791|riken|9930010I05 | Gm7968 | 2.14 |
| A_55_P2040549 | ref|NM_008888|ens|ENSMUST00000012664|gb|AK148638|gb|BC079610 | Phox2b | 2.14 |
| A_52_P463962 | ref|NM_183296|gb|BC132658|gb|BC132660|gb|BC107303 | Krtap16-10 | 2.13 |
| A_55_P2062589 | ref|NM_008639|gb|U52222|tc|NP381599|nap|NAP057386-1 | Mtnr1a | 2.13 |
| A_55_P2157384 | ref|NM_001111293|ref|NM_001045539|ens|ENSMUST00000114520|ens|ENSMUST00000097221 | Xlr5b | 2.13 |
| A_55_P2085865 | ref|XM_916809 | LOC640067 | 2.13 |
| A_55_P2081790 | ref|NM_001159633|ens|ENSMUST00000107646|gb|AK077951|tc|TC1621271 | Slc44a1 | 2.13 |
| A_52_P455251 | ref|NM_010132|ens|ENSMUST00000062216|gb|AK132093|gb|AY117415 | Emx2 | 2.13 |
| A_55_P2048912 | ref|NM_001033308|ens|ENSMUST00000045154|gb|AK150471|gb|AK153536 | BC013712 | 2.13 |
| A_55_P2184945 | ref|NM_028025|ref|NM_001113734|ens|ENSMUST00000071597|ens|ENSMUST00000101408 | Mageb16 | 2.13 |
| A_55_P1987966 | ens|ENSMUST00000111863|ens|ENSMUST00000111864|ens|ENSMUST00000100696|ens|ENSMUST00000064162 | ENSMUST00000111863 | 2.13 |
| A_66_P117366 | ens|ENSMUST00000122044|gb|AK158709|gb|AK164945|riken|K230304A10 | ENSMUST00000122044 | 2.13 |
| A_55_P2357254 | gb|BB554159 | 5330430P22Rik | 2.13 |
| A_55_P2068461 | ref|NM_008312|ens|ENSMUST00000112831|ens|ENSMUST00000036303|ens|ENSMUST00000096299 | Htr2c | 2.12 |
| A_30_P01028508 | Unknown | chr6:121035524-121061074_F | 2.12 |
| A_55_P1964245 | ref|NM_146959|ens|ENSMUST00000059011|ref|XM_001471823|nap|NAP057865-1 | Olfr631 | 2.12 |
| A_52_P452787 | ref|NM_011052|ref|NM_001164677|ref|NM_001164678|ens|ENSMUST00000111861 | Pdcd6ip | 2.12 |
| A_55_P1972104 | ref|NM_212441|ref|NM_212442|ref|NM_016870|ens|ENSMUST00000106528 | Acsm3 | 2.12 |
| A_30_P01025907 | Unknown | chr4:135138014-135174889_R | 2.12 |
| A_55_P1952027 | ref|NM_001011769|ens|ENSMUST00000075607|gb|BC147132|gb|BC147131 | Olfr317 | 2.12 |
| A_55_P2213348 | gb|AK039175|riken|A230104L24 | 5330421C15Rik | 2.12 |
| A_30_P01032426 | Unknown | chr5:108538417-108550517_R | 2.12 |
| A_51_P233018 | ref|NM_146314|ens|ENSMUST00000080474|gb|BC131956|gb|BC131958 | Olfr601 | 2.12 |
| A_55_P2167930 | ens|ENSMUST00000101404|gb|AK149002|riken|7120474D01 | ENSMUST00000101404 | 2.12 |
| A_55_P2135486 | ref|XM_920697|ref|XM_898878|tc|TC1649424 | Gm5907 | 2.12 |
| A_52_P531651 | ref|NM_022321|ref|NM_001162500|ens|ENSMUST00000023074|gb|AK088419 | Parvg | 2.12 |
| A_51_P511800 | ref|NM_025909|ens|ENSMUST00000035780|gb|AK076209|gb|AK008020 | Oma1 | 2.12 |
| A_55_P2026405 | ref|NM_022378|ens|ENSMUST00000071281|tc|TC1588480 | Foxb1 | 2.12 |
| A_51_P441745 | ref|XR_002314|ref|XR_005138|gb|AK021396|tc|TC1593713 | E130119H09Rik | 2.11 |
| A_52_P58283 | ref|NM_145983|gb|AF108659|gb|BC021787|gb|L22218 | Kcna5 | 2.11 |
| A_51_P471458 | ref|NM_020564|ens|ENSMUST00000122819|ens|ENSMUST00000000755|gb|AK142243 | Sult5a1 | 2.11 |
| A_51_P102421 | ref|NM_019952|ens|ENSMUST00000138090|ens|ENSMUST00000046506|gb|BC104258 | Clcf1 | 2.11 |
| A_30_P01027676 | Unknown | chr19:24978025-24988025_F | 2.11 |
| A_30_P01018769 | Unknown | chr13:108577843-108679708_R | 2.11 |
| A_30_P01025191 | Unknown | chr12:16447157-16447657_F | 2.11 |
| A_30_P01026584 | Unknown | chr16:57811599-58042454_F | 2.11 |
| A_55_P2160761 | ref|NM_001122676|ref|NM_001122675|ens|ENSMUST00000063190|ens|ENSMUST00000118196 | Zcchc2 | 2.11 |
| A_55_P2416494 | gb|AK020235|gb|BC051511|gb|BC038155|tc|TC1660714 | 8430426J06Rik | 2.11 |
| A_30_P01019654 | Unknown | chr18:47327300-47333819_F | 2.11 |
| A_55_P1990755 | ref|NM_024459|ens|ENSMUST00000102880|gb|AK160938|gb|AK145541 | Ppp3r1 | 2.11 |
| A_55_P2296320 | gb|AK030206|riken|4933422O13|nap|NAP073525-1 | Gm13112 | 2.11 |
| A_30_P01022665 | Unknown | chr15:84737071-84744158_F | 2.11 |
| A_55_P2017397 | ref|NR_033524|ens|ENSMUST00000100522|gb|AK138406|riken|A230096H11 | Gm11529 | 2.11 |
| A_55_P1994418 | ref|NM_025634|gb|AK009997|gb|AK009632|gb|AK009984 | 2310042E22Rik | 2.11 |
| A_51_P388325 | ref|NM_009904|ens|ENSMUST00000109831|gb|D86323|gb|U08373 | Clgn | 2.11 |
| A_51_P201945 | ref|NM_025747|ens|ENSMUST00000034048|gb|AK045291|gb|AK129358 | 4933411K20Rik | 2.11 |
| A_55_P2070786 | ens|ENSMUST00000097193|tc|TC1673037 | ENSMUST00000097193 | 2.11 |
| A_51_P131442 | ref|NM_013874|ens|ENSMUST00000049977|gb|U48238|gb|AK158649 | Dpf1 | 2.11 |
| A_30_P01023169 | Unknown | chr2:32950586-32984936_R | 2.11 |
| A_55_P2067831 | ens|ENSMUST00000044306|ref|XM_001473538 | ENSMUST00000044306 | 2.11 |
| A_55_P2077488 | ens|ENSMUST00000060348|ref|XM_001477403|ref|XM_001478760|gb|AK017242 | 5330417H12Rik | 2.11 |
| A_51_P408227 | ref|NM_030218|ens|ENSMUST00000046833|gb|AK168406|gb|AK020266 | 9130017N09Rik | 2.11 |
| A_55_P2010401 | ens|ENSMUST00000079975|gb|AF038896|tc|NP049127 | ENSMUST00000079975 | 2.11 |
| A_66_P119636 | ref|NM_153808|ens|ENSMUST00000036334|ens|ENSMUST00000087556|ref|XR_033678 | Smc5 | 2.11 |
| A_52_P640204 | ref|NM_011484|ens|ENSMUST00000028050|ens|ENSMUST00000114738|ens|ENSMUST00000102960 | Stam | 2.11 |
| A_55_P2091193 | ref|XM_001480396 | LOC100048513 | 2.11 |
| A_52_P593465 | ref|NM_178407|ens|ENSMUST00000076623|ref|XM_140607|ref|XM_001474633 | Arap2 | 2.11 |
| A_55_P1987984 | ref|XM_001478602|tc|TC1649157|tc|TC1711149 | LOC100047651 | 2.10 |
| A_55_P2046064 | ref|NM_178258|ens|ENSMUST00000105526|ens|ENSMUST00000036564|gb|AK040641 | Il22ra2 | 2.10 |
| A_52_P521882 | ref|NM_026812|ens|ENSMUST00000032747|gb|AK004078|gb|BC038310 | Hddc3 | 2.10 |
| A_55_P2142326 | ref|XM_923315|ref|XM_356204|tc|TC1616511 | XM_923315 | 2.10 |
| A_55_P2439712 | ref|NM_028102|ens|ENSMUST00000098860|ens|ENSMUST00000033975|gb|AK035532 | Ddhd2 | 2.10 |
| A_66_P132870 | ens|ENSMUST00000064242|gb|AK039020|tc|TC1621751|riken|A230086C11 | ENSMUST00000064242 | 2.10 |
| A_30_P01028966 | Unknown | chr6:99472981-99484210_R | 2.10 |
| A_66_P127007 | ref|XM_001479118|gb|AK052659|tc|TC1621318|riken|D630015H07 | Gm12273 | 2.10 |
| A_30_P01023122 | Unknown | chr5:124489025-124506750_F | 2.10 |
| A_55_P2077635 | ref|XR_030560 | LOC673642 | 2.10 |
| A_55_P2133096 | ref|NM_029578|ens|ENSMUST00000022727|gb|AK165955|gb|AK154341 | Tgds | 2.10 |
| A_55_P2170439 | ref|XM_001474691|ref|XM_001477738|gb|AK157043|riken|F830114M07 | Gm2680 | 2.10 |
| A_55_P2017377 | ref|NM_146721|ens|ENSMUST00000053941|gb|BC104096|gb|BC104097 | Olfr424 | 2.10 |
| A_55_P2154719 | gb|AK134020|riken|5830408J16 | BC064078 | 2.10 |
| A_55_P2137979 | ref|NM_177638|ens|ENSMUST00000097299|ens|ENSMUST00000112924|ens|ENSMUST00000071826 | Crb3 | 2.10 |
| A_52_P37702 | ens|ENSMUST00000045307|gb|AK011813|tc|TC1733112|riken|2610103N14 | ENSMUST00000045307 | 2.10 |
| A_55_P1993348 | ref|XR_031283|ref|XR_032644|ref|XR_031115|ref|XR_034756 | Gm6274 | 2.10 |
| A_51_P445841 | ref|NM_145470|gb|AK219129|gb|AK220300|gb|BC004774 | Depdc6 | 2.09 |
| A_55_P2164189 | ref|NM_023603|ens|ENSMUST00000030623|ref|XM_001475106 | Sfpq | 2.09 |
| A_51_P123262 | ref|NM_029530|ens|ENSMUST00000057503|gb|AK014127|gb|AK018222 | 6330527O06Rik | 2.09 |
| A_30_P01025244 | Unknown | chr17:21653676-21667959_F | 2.09 |
| A_55_P1965114 | ref|NM_001163750|ref|NM_027141|ref|NM_001163751|ens|ENSMUST00000068508 | Spsb3 | 2.09 |
| A_55_P2129068 | ref|NM_001115130|ens|ENSMUST00000061588|gb|BC037671|gb|BC027138 | Zbtb44 | 2.09 |
| A_30_P01024586 | Unknown | chr13:34693404-34717987_R | 2.09 |
| A_55_P2179206 | ref|NM_027394|ens|ENSMUST00000034986|gb|AK049229|tc|TC1585477 | Ube2cbp | 2.09 |
| A_55_P2416862 | gb|AK076446|tc|TC1620522|riken|4832439D14 | Tmem175 | 2.09 |
| A_55_P2323951 | gb|AK087124|riken|E030028L23 | AK087124 | 2.09 |
| A_66_P100496 | ref|NM_028561|ens|ENSMUST00000053257|gb|AK006975|tc|TC1594403 | Speer4b | 2.09 |
| A_30_P01019480 | Unknown | chr17:74580316-74599991_R | 2.09 |
| A_55_P2032905 | ref|XM_001478453 | LOC100047581 | 2.09 |
| A_52_P499299 | ens|ENSMUST00000098472|gb|AK042481|gb|AK029729|riken|A630095L01 | ENSMUST00000098472 | 2.09 |
| A_55_P1999097 | ref|NM_009615|ens|ENSMUST00000101551|gb|BC114999|tc|TC1574522 | Adam17 | 2.09 |
| A_51_P181865 | ref|NM_028053|ens|ENSMUST00000144167|ens|ENSMUST00000030127|gb|AB261159 | Tmem38b | 2.09 |
| A_52_P1004481 | ref|XM_001472482|gb|AK082308|riken|C230036E17|nap|NAP084636-1 | Gm2109 | 2.09 |
| A_55_P2017169 | ref|XM_978743|ref|XM_975505 | Gm7750 | 2.09 |
| A_55_P2322064 | gb|AK089619|tc|TC1598397|riken|F830005D05|nap|NAP099890-001 | F830005D05Rik | 2.09 |
| A_52_P560006 | ref|NM_008426|gb|BC103671|gb|BC103673|gb|BC103674 | Kcnj3 | 2.09 |
| A_55_P1971538 | ref|NM_001081177|ens|ENSMUST00000100473|gb|BC172821|nap|NAP122201-001 | Kif13b | 2.09 |
| A_30_P01023197 | Unknown | chr7:28588159-28617009_R | 2.08 |
| A_30_P01020300 | Unknown | chr9:37063322-37063718_F | 2.08 |
| A_55_P1965462 | tc|TC1746812 | TC1746812 | 2.08 |
| A_30_P01028838 | Unknown | chr7:137669441-137675722_R | 2.08 |
| A_30_P01018267 | Unknown | chr13:16050871-16065396_R | 2.08 |
| A_52_P89717 | ref|XM_001472869|ref|XM_885330|nap|NAP017720-001 | Dph3b-ps | 2.08 |
| A_52_P410732 | ref|NM_011135|ens|ENSMUST00000149992|ens|ENSMUST00000135269|ens|ENSMUST00000132032 | Cnot7 | 2.08 |
| A_52_P609695 | ref|NM_020278|ens|ENSMUST00000087252|gb|AK031996|gb|BC066090 | Lgi1 | 2.08 |
| A_55_P1959849 | ref|NM_001099643|ref|NM_001145038|ref|NM_001168334|ref|NM_001168337 | Gm3750 | 2.08 |
| A_30_P01021331 | Unknown | chrX:75813213-75829323_R | 2.08 |
| A_51_P193189 | ens|ENSMUST00000040158|gb|BC027076|tc|TC1590529|nap|NAP110697-1 | Slc26a8 | 2.08 |
| A_55_P1988232 | ens|ENSMUST00000108743|gb|AK050942|tc|TC1595450|riken|D030041I21 | ENSMUST00000108743 | 2.08 |
| A_66_P100951 | ens|ENSMUST00000050921|gb|AK079546|tc|TC1592419|riken|A230052G05 | ENSMUST00000050921 | 2.08 |
| A_30_P01029553 | Unknown | chrX:45814752-45816313_R | 2.08 |
| A_52_P306697 | ref|NM_007868|ens|ENSMUST00000113992|ens|ENSMUST00000057711|ens|ENSMUST00000113991 | Dmd | 2.08 |
| A_55_P2054817 | ref|NM_212452|ens|ENSMUST00000078527|gb|AY509975|gb|BC172664 | Rxfp1 | 2.07 |
| A_30_P01032410 | Unknown | chr9:56910620-56919495_F | 2.07 |
| A_55_P2028883 | ref|NM_172998|ref|NM_001109902|ens|ENSMUST00000100822|ens|ENSMUST00000121369 | Rnft2 | 2.07 |
| A_30_P01032840 | Unknown | chr14:118774481-118873242_R | 2.07 |
| A_55_P2054982 | ref|XM_001479457|ref|XM_001479588|tc|TC1670867 | Gm4053 | 2.07 |
| A_55_P1973326 | ref|XM_001476308 | Gm3165 | 2.07 |
| A_30_P01025317 | Unknown | chr1:182693388-182698787_R | 2.07 |
| A_55_P2082539 | ref|NM_134198|ens|ENSMUST00000079633|gb|BC127026|tc|NP496264 | Vmn1r234 | 2.07 |
| A_55_P2202043 | gb|AK021252|riken|C430045I18|nap|NAP116938-1|nap|NAP105423-1 | C430045I18Rik | 2.07 |
| A_30_P01026328 | Unknown | chr1:123016343-123017009_F | 2.07 |
| A_55_P2060592 | ref|NM_010449|ens|ENSMUST00000120363|ens|ENSMUST00000114436|ens|ENSMUST00000000964 | Hoxa1 | 2.07 |
| A_51_P393968 | ref|NM_007701|ens|ENSMUST00000021665|gb|AK162139|gb|L34808 | Vsx2 | 2.07 |
| A_55_P2007121 | ref|NM_177278|ens|ENSMUST00000093007|gb|AK042907|gb|AK139210 | L3mbtl4 | 2.07 |
| A_55_P2040594 | ref|NM_053114|ens|ENSMUST00000040961|gb|AK050551|tc|TC1591003 | Pabpc5 | 2.07 |
| A_51_P398037 | ref|NM_018732|gb|AK048407|tc|TC1606576|riken|C130058B11 | Scn3a | 2.07 |
| A_52_P398211 | ref|XR_032858|ref|XR_033266|gb|W75484|nap|NAP028510-1 | Gm12229 | 2.07 |
| A_52_P673499 | ref|NM_009171|gb|AF237702|gb|AK010439|tc|TC1575515 | Shmt1 | 2.07 |
| A_55_P2017389 | ref|XR_031757|ref|XR_032947 | Gm8876 | 2.07 |
| A_55_P1979364 | ens|ENSMUST00000103550|ens|ENSMUST00000103548|ens|ENSMUST00000103545|ens|ENSMUST00000103537 | ENSMUST00000103550 | 2.07 |
| A_30_P01018763 | Unknown | chr16:59556175-59603176_R | 2.07 |
| A_55_P1960246 | ref|XR_032704 | Gm13247 | 2.07 |
| A_55_P2000658 | ref|NR_033578|ref|XR_002142|gb|AK136817|riken|9330023E08 | Gm15645 | 2.07 |
| A_51_P520639 | ref|NM_177822|ens|ENSMUST00000047098|gb|AK028891|gb|BC052484 | Mslnl | 2.07 |
| A_52_P662244 | ref|NM_001012310|ens|ENSMUST00000057026|gb|AK149369|gb|BC042739 | AI132487 | 2.07 |
| A_55_P2095663 | ref|NM_008829|gb|AK137726|tc|TC1635777|riken|9930117J11 | Pgr | 2.07 |
| A_55_P2072479 | ens|ENSMUST00000050767 | ENSMUST00000050767 | 2.07 |
| A_55_P1954403 | ref|NM_177184|ens|ENSMUST00000057972|ens|ENSMUST00000098601|ens|ENSMUST00000077879 | Vps13c | 2.07 |
| A_30_P01031830 | Unknown | chr8:110196655-110390197_F | 2.07 |
| A_30_P01021769 | Unknown | chr10:14888108-14978308_R | 2.07 |
| A_51_P434059 | ref|NM_001001496|ens|ENSMUST00000069417|gb|BC117760|gb|AK146838 | Gja6 | 2.07 |
| A_52_P502849 | ens|ENSMUST00000103401|gb|BC108385|gb|Z25444|gb|Z25452 | ENSMUST00000103401 | 2.06 |
| A_55_P2093221 | ref|NM_033604|ens|ENSMUST00000113595|ens|ENSMUST00000034739|gb|AK164502 | Rnf111 | 2.06 |
| A_55_P2017799 | ref|XM_975517 | XM_975517 | 2.06 |
| A_55_P2095380 | ref|XM_001474730|gb|AK005914|riken|1700012I11|nap|NAP090535-1 | 1700012I11Rik | 2.06 |
| A_55_P2134591 | ref|NM_178776|ens|ENSMUST00000052702|ref|XM_001471885|gb|BC049715 | BC049715 | 2.06 |
| A_55_P2318016 | gb|DT902267 | BB236558 | 2.06 |
| A_30_P01030408 | Unknown | chr12:72103832-72116875_F | 2.06 |
| A_30_P01026071 | Unknown | chr4:97106832-97107437_F | 2.06 |
| A_55_P2142605 | ref|XM_001474988|ref|XM_001476951 | LOC100045825 | 2.06 |
| A_55_P2142937 | ref|XR_033718|ref|XR_033082 | Gm8007 | 2.06 |
| A_55_P2091561 | ref|NM_147003|ens|ENSMUST00000050678|gb|BC104101|gb|BC104102 | Olfr139 | 2.06 |
| A_52_P625880 | ens|ENSMUST00000064330|gb|AK029366|tc|TC1591311|riken|4833406C17 | ENSMUST00000064330 | 2.06 |
| A_55_P2091230 | ref|XR_031106|ref|XR_031303|nap|NAP113008-1 | Gm5391 | 2.06 |
| A_55_P2415701 | gb|AK015835|riken|4930518P08|nap|NAP074351-1 | 4930518P08Rik | 2.06 |
| A_52_P876147 | ens|ENSMUST00000138595|ref|XM_001474855|ref|XM_977156|gb|AK086911 | E030010N08Rik | 2.06 |
| A_51_P378576 | ref|NM_145222|ens|ENSMUST00000113306|gb|BC060507|gb|AK131975 | B3gnt7 | 2.06 |
| A_55_P1984846 | ens|ENSMUST00000070594|tc|TC1676502|nap|NAP068452-1 | ENSMUST00000070594 | 2.06 |
| A_30_P01022900 | Unknown | chr5:113769776-113769837_F | 2.06 |
| A_51_P109570 | ref|NM_030599|ens|ENSMUST00000032472|ref|XM_001471613|ref|XM_001471771 | Klrb1b | 2.06 |
| A_55_P2425432 | gb|AK006067|tc|TC1654422|riken|1700017I07|nap|NAP090574-1 | 1700017I07Rik | 2.06 |
| A_55_P2043481 | ref|NM_021717|ens|ENSMUST00000001561|gb|BC115523|gb|AJ278170 | Nrip2 | 2.06 |
| A_30_P01027673 | Unknown | chr16:13752215-13764390_F | 2.06 |
| A_51_P390775 | ref|NM_009455|ens|ENSMUST00000022296|gb|X92665|gb|AK148266 | Ube2e1 | 2.06 |
| A_52_P297773 | ref|NM_001081252|ens|ENSMUST00000022732|ens|ENSMUST00000156203|ens|ENSMUST00000046196 | Uggt2 | 2.05 |
| A_55_P2003973 | ref|XM_486328|ref|XM_905189 | Gm4980 | 2.05 |
| A_51_P416822 | ref|NM_020259|gb|AK034610|tc|TC1601104|riken|9430014J21 | Hhip | 2.05 |
| A_55_P2011732 | ref|NR_033482 | Gm6938 | 2.05 |
| A_55_P2006604 | ref|NM_026054|ens|ENSMUST00000100765|ens|ENSMUST00000046689|gb|BC139436 | 2810474O19Rik | 2.05 |
| A_30_P01027943 | Unknown | chr3:9401052-9415202_F | 2.05 |
| A_30_P01028296 | Unknown | chr1:42759171-42771337_F | 2.05 |
| A_55_P2117028 | ref|NM_028860|ens|ENSMUST00000123506|ens|ENSMUST00000040448|gb|AK122261 | Mtmr3 | 2.05 |
| A_55_P2118143 | ref|NM_009561|ens|ENSMUST00000145131|ens|ENSMUST00000077780|ref|XM_001478839 | Zfp61 | 2.05 |
| A_51_P177071 | ref|NM_008734|ref|NM_001190187|ref|NM_001190188|ens|ENSMUST00000042378 | Nrg3 | 2.05 |
| A_55_P2025829 | ref|XR_031362|ref|XR_031383|ref|XR_034780 | LOC633219 | 2.05 |
| A_55_P2040255 | ens|ENSMUST00000095282|gb|AK034302|tc|TC1589114|riken|9330175B10 | ENSMUST00000095282 | 2.04 |
| A_55_P1964951 | ens|ENSMUST00000085967|ref|XM_001473929|gb|AK046282|tc|TC1613882 | ENSMUST00000085967 | 2.04 |
| A_55_P2196185 | ref|NR_030693|gb|AK076677|tc|TC1614291|riken|4930412C18 | 4930412C18Rik | 2.04 |
| A_51_P204898 | ref|NM_027050|ens|ENSMUST00000020508|gb|AK005748|gb|BU936803 | 1700008A04Rik | 2.04 |
| A_30_P01032042 | Unknown | chr3:137736327-137752677_F | 2.04 |
| A_55_P1975874 | ref|NM_001142959|ens|ENSMUST00000106820|ens|ENSMUST00000062945|gb|BC027668 | Bcl2l15 | 2.04 |
| A_30_P01023171 | Unknown | chr10:93819821-93976621_F | 2.04 |
| A_30_P01025825 | Unknown | chr14:27930334-27930974_F | 2.04 |
| A_51_P125607 | ref|NM_176954|ens|ENSMUST00000120508|ens|ENSMUST00000120856|ens|ENSMUST00000119060 | Celf5 | 2.04 |
| A_51_P260288 | ens|ENSMUST00000098443|gb|AK016705|tc|NP584394|riken|4933406O10 | ENSMUST00000098443 | 2.04 |
| A_52_P517224 | ref|NM_001039048|ens|ENSMUST00000030638|ens|ENSMUST00000105875|gb|AJ278734 | Trim63 | 2.04 |
| A_55_P2196922 | ref|NR_001461|gb|AK042559|tc|TC1617188|riken|A730005L09 | Kcnq1ot1 | 2.04 |
| A_55_P2029995 | ref|NM_001085521|ens|ENSMUST00000109934|ens|ENSMUST00000109935|tc|TC1625584 | Tmem90b | 2.04 |
| A_30_P01026803 | Unknown | chr2:177247075-177272025_R | 2.04 |
| A_52_P156452 | ref|NM_007717|ref|NM_001111110|ens|ENSMUST00000110391|ens|ENSMUST00000110390 | Cmah | 2.04 |
| A_55_P2321125 | gb|AK039302|gb|AK163456|riken|A330019B12|riken|B230113G15 | D230040A04Rik | 2.04 |
| A_55_P2011425 | ref|NM_176999|ens|ENSMUST00000077659|gb|AK020037|tc|TC1600808 | Atp10b | 2.04 |
| A_30_P01029435 | Unknown | chr6:99472981-99484210_F | 2.04 |
| A_55_P2140745 | ref|XM_135380 | LOC235882 | 2.04 |
| A_55_P2126002 | ens|ENSMUST00000108187|ref|XM_001000510|ref|XM_906694|gb|AK006173 | Thap8 | 2.04 |
| A_52_P594410 | ref|NM_010827|ens|ENSMUST00000027062|gb|BC103592|gb|BC103593 | Msc | 2.04 |
| A_66_P100182 | ref|NM_001011753|gb|BC139227|gb|BC139226|tc|TC1600211 | Olfr115 | 2.04 |
| A_30_P01022573 | Unknown | chr19:32911584-32911747_F | 2.04 |
| A_55_P2157478 | ref|XR_033658|ref|XR_034433|gb|AV503795 | Gm8590 | 2.04 |
| A_55_P1977548 | ref|NM_011949|ens|ENSMUST00000023462|ens|ENSMUST00000069107|gb|AK048127 | Mapk1 | 2.04 |
| A_55_P2121772 | ref|NM_001164679|ens|ENSMUST00000093450|ref|XM_905080|gb|BC059855 | Ano8 | 2.04 |
| A_55_P1979893 | ref|NM_017376|ref|NM_153484|ens|ENSMUST00000023024|ens|ENSMUST00000052561 | Tef | 2.04 |
| A_52_P206998 | ref|XM_001473915|gb|AK029093|riken|4732490P15 | LOC100045240 | 2.04 |
| A_55_P2039541 | ref|NM_153820|ens|ENSMUST00000112822|ens|ENSMUST00000112821|ens|ENSMUST00000055776 | Arhgap15 | 2.03 |
| A_55_P1957932 | ens|ENSMUST00000020855|ens|ENSMUST00000108696|ref|XM_917005|ref|XM_894470 | 1700086D15Rik | 2.03 |
| A_51_P310821 | ref|NM_010453|ens|ENSMUST00000048794|gb|X16840|gb|M36604 | Hoxa5 | 2.03 |
| A_55_P2073542 | ref|NM_001034894|ens|ENSMUST00000096265|gb|AK145057|tc|TC1592580 | Foxr2 | 2.03 |
| A_55_P2021861 | ref|NM_183424|ens|ENSMUST00000057407|gb|AK029144|tc|TC1589309 | Qrfp | 2.03 |
| A_55_P2167530 | ens|ENSMUST00000043503|gb|BU610818|tc|TC1601307|tc|TC1680956 | Scube3 | 2.03 |
| A_55_P2100830 | ref|NM_001111141|ref|XM_001480333 | Gm505 | 2.03 |
| A_55_P1972252 | ref|NM_133649|ref|NM_133648|ens|ENSMUST00000110991|ens|ENSMUST00000110987 | Slc12a6 | 2.03 |
| A_30_P01024587 | Unknown | chr12:76769763-76770169_R | 2.03 |
| A_55_P1955159 | ref|NM_025746|ens|ENSMUST00000073179|ref|XM_001474610|ref|XM_001476648 | 4933415F23Rik | 2.03 |
| A_66_P105046 | ref|NM_008360|ens|ENSMUST00000059081|gb|AY362457|gb|AY157834 | Il18 | 2.03 |
| A_55_P2013019 | ref|NM_172989|ref|NM_010336|ens|ENSMUST00000107566|ens|ENSMUST00000107571 | Lpar1 | 2.03 |
| A_52_P231737 | ref|NM_019673|ens|ENSMUST00000029214|gb|AK176960|gb|AF041476 | Actl6a | 2.03 |
| A_66_P132462 | ref|XM_001006607|ref|XM_488311|ref|XM_906768|nap|NAP030024-1 | Gm14451 | 2.02 |
| A_51_P223776 | ref|NM_145434|ens|ENSMUST00000064941|gb|AK041047|gb|AK154931 | Nr1d1 | 2.02 |
| A_55_P2028474 | ref|NM_001098225|ens|ENSMUST00000088761|ens|ENSMUST00000115386|ens|ENSMUST00000050166 | Adam22 | 2.02 |
| A_51_P358122 | ref|NM_001011770|ens|ENSMUST00000050287|tc|TC1599563|nap|NAP108583-1 | Olfr332 | 2.02 |
| A_30_P01020525 | Unknown | chr2:94017095-94019092_R | 2.02 |
| A_55_P2129360 | ref|NM_133500|ref|NM_133501|ens|ENSMUST00000071201|ens|ENSMUST00000113833 | Ntng2 | 2.02 |
| A_55_P2090045 | ens|ENSMUST00000110901|gb|BC057874|tc|TC1590471 | ENSMUST00000110901 | 2.02 |
| A_55_P2032363 | ref|NM_001037170|ens|ENSMUST00000005817|ens|ENSMUST00000111327|ens|ENSMUST00000111326 | Tomm40l | 2.02 |
| A_55_P1984755 | ref|XM_001475415 | Gm7972 | 2.02 |
| A_55_P2275590 | gb|BC100571 | A630012P03Rik | 2.02 |
| A_51_P125882 | ref|NM_175681|ens|ENSMUST00000108679|ens|ENSMUST00000021289|ens|ENSMUST00000051765 | Glp2r | 2.02 |
| A_30_P01026060 | Unknown | chr17:22090757-22099418_R | 2.02 |
| A_55_P1973491 | ref|NM_022320|ref|NM_001104529|ens|ENSMUST00000027489|ens|ENSMUST00000064480 | Gpr35 | 2.02 |
| A_66_P102607 | ref|NM_001037758|ref|NM_009771|gb|AK220183|tc|TC1631878 | Btrc | 2.02 |
| A_51_P285042 | ref|NM_030112|ens|ENSMUST00000028767|gb|AK018342|tc|TC1604980 | Rtf1 | 2.02 |
| A_51_P470724 | ref|NM_026030|ens|ENSMUST00000099173|gb|BC003848|tc|TC1573963 | Eif2s2 | 2.02 |
| A_55_P1993775 | ens|ENSMUST00000070075|ens|ENSMUST00000085023|ens|ENSMUST00000111671|gb|BC019386 | ENSMUST00000070075 | 2.02 |
| A_30_P01027602 | Unknown | chr7:50403100-50418175_F | 2.02 |
| A_30_P01032162 | Unknown | chr16:29907604-29946602_R | 2.02 |
| A_30_P01018588 | Unknown | chr2:175640275-175701875_F | 2.02 |
| A_52_P372400 | ref|XM_976926|gb|BC032201|gb|AK139826|tc|TC1592195 | Gm7109 | 2.02 |
| A_30_P01025783 | Unknown | chr4:129376440-129391690_R | 2.02 |
| A_52_P1831 | ref|NM_009560|ref|NM_029531|nap|NAP121783-001 | Zfp60 | 2.01 |
| A_30_P01026348 | Unknown | chr15:60656586-60685333_R | 2.01 |
| A_55_P2201426 | gb|C87011 | Gm11381 | 2.01 |
| A_55_P2035667 | ref|NM_008194|ref|NM_212444|ens|ENSMUST00000081161|ens|ENSMUST00000069684 | Gyk | 2.01 |
| A_52_P436447 | ref|NM_028048|ens|ENSMUST00000102606|gb|AK007453|gb|BC004569 | Slc25a35 | 2.01 |
| A_55_P2024918 | ref|XM_001479149|tc|TC1612244 | XM_001479149 | 2.01 |
| A_30_P01032053 | Unknown | chr17:79042382-79052046_R | 2.01 |
| A_55_P2135291 | gb|AK158289|tc|TC1591133|riken|F930101C16 | Gm7276 | 2.01 |
| A_55_P2121081 | ref|NM_029402|ens|ENSMUST00000080089|gb|AK160597|gb|AK144693 | Cul2 | 2.01 |
| A_30_P01028771 | Unknown | chr13:78566508-78581785_F | 2.01 |
| A_51_P466221 | ref|NM_144547|ens|ENSMUST00000023809|gb|BC119571|gb|AF503863 | Amhr2 | 2.01 |
| A_55_P2102489 | ens|ENSMUST00000064509|ens|ENSMUST00000136421|gb|AK038656|riken|A230052N11 | ENSMUST00000064509 | 2.01 |
| A_55_P2019734 | ens|ENSMUST00000070502|gb|AK083112|tc|TC1587882|riken|C630015B10 | ENSMUST00000070502 | 2.01 |
| A_30_P01027046 | Unknown | chr18:78190463-78235438_F | 2.01 |
| A_30_P01019796 | Unknown | chr2:129402701-129403112_R | 2.01 |
| A_55_P1980601 | ref|NM_172701|ens|ENSMUST00000106156|ref|XM_001472102|gb|AK007366 | Oscp1 | 2.01 |
| A_55_P1994190 | ref|NM_181273|ens|ENSMUST00000079322|gb|AK081330|tc|TC1583790 | Heph | 2.01 |
| A_55_P2107387 | ref|NM_172676|ens|ENSMUST00000098967|ens|ENSMUST00000060173|gb|BC019475 | Samd10 | 2.01 |
| A_55_P2157403 | ens|ENSMUST00000092883|gb|AY512957|nap|NAP125253-1|nap|NAP054559-1 | ENSMUST00000092883 | 2.01 |
| A_30_P01023577 | Unknown | chr5:77188626-77219161_F | 2.01 |
| A_55_P2023572 | ens|ENSMUST00000100486|gb|AK160591|gb|AK158012|riken|2510025A17 | ENSMUST00000100486 | 2.01 |
| A_55_P2084696 | ref|NM_175658|ens|ENSMUST00000072391|gb|BC107354|gb|BC107355 | Hist1h2aa | 2.01 |
| A_66_P123743 | ref|XR_033426|nap|NAP112752-1 | Gm4939 | 2.00 |
| A_55_P2024021 | ref|NM_013795|ens|ENSMUST00000075431|ref|XM_892716|ref|XM_907199 | Atp5l | 2.00 |
| A_30_P01025476 | Unknown | chr2:25182970-25188091_R | 2.00 |
| A_52_P151278 | ref|NM_146164|ref|NM_001168652|ens|ENSMUST00000031734|gb|AK088331 | Lrch4 | 2.00 |
| A_55_P2088870 | ref|NM_173424|gb|BC113763|gb|AK148347|gb|AK084871 | Zbtb37 | 2.00 |
| A_55_P2231342 | gb|AK042844|tc|TC1632527|riken|A730030A11|nap|NAP024299-001 | A030010E16Rik | 2.00 |
| A_30_P01021344 | Unknown | chr4:128059190-128061132_F | 2.00 |
| A_30_P01027726 | Unknown | chr8:26212577-26218527_F | 2.00 |
| A_55_P2018821 | ref|XR_032088 | LOC634350 | 2.00 |
| A_51_P480328 | ref|NM_133222|ens|ENSMUST00000046977|gb|AF385682|tc|TC1579650 | Eltd1 | 2.00 |
| A_30_P01026509 | Unknown | chr10:92550314-92577555_F | 2.00 |
| A_55_P2105958 | ref|NM_177781|ens|ENSMUST00000041447|gb|AK045771|tc|TC1587947 | Trpa1 | 2.00 |
| A_55_P2021345 | ref|XM_001475884|ref|XM_001476423 | Gm14717 | 2.00 |
| A_55_P2187030 | ens|ENSMUST00000103537|ref|XM_911832|gb|AF296436|gb|AF303857 | ENSMUST00000103537 | 2.00 |
| A_55_P2050088 | ens|ENSMUST00000111952|gb|AK053686|gb|AK042745|tc|TC1589440 | ENSMUST00000111952 | 2.00 |
| A_55_P2066440 | ref|NM_001099635|ens|ENSMUST00000108689|ref|XM_908146|gb|M74753 | Myh3 | 2.00 |

**Table S6**: Genes down regulated ≥ 2 fold in the cerebral hemisphere of female pups from mothers having FA supplementation during gestation at 4 mg/kg in comparison to mothers at 0.4 mg/kg diet.

| **UniqueID** | **Accession** | **Symbol** | **Fold Change** |
| --- | --- | --- | --- |
| A_55_P2174736 | ref|XM_001480007 | LOC100048027 | -6.38 |
| A_30_P01025211 | Unknown | chr5:22810359-22836386_F | -6.31 |
| A_55_P2100866 | Unknown | A_55_P2100866 | -5.76 |
| A_30_P01027666 | Unknown | chr10:122410471-122422759_F | -5.76 |
| A_55_P2114908 | ref|NM_001162947|ref|NM_011848|ens|ENSMUST00000110730|ens|ENSMUST00000033865 | Nek3 | -5.75 |
| A_55_P2182596 | ref|XM_001475501|gb|AK077057|riken|4932441L02|nap|NAP089207-1 | Gm3045 | -5.62 |
| A_55_P2204676 | gb|BG069650 | AU020147 | -5.53 |
| A_55_P2097284 | ref|XM_001479063 | LOC100047654 | -5.25 |
| A_30_P01030848 | Unknown | chr7:48794017-48815817_R | -5.17 |
| A_30_P01030367 | Unknown | chr17:7838192-7853103_F | -5.06 |
| A_55_P1981276 | ref|XM_001472979|ref|XM_001474323|gb|AK131772|riken|1700102N24 | Gm2582 | -5.04 |
| A_55_P2275590 | gb|BC100571 | A630012P03Rik | -4.96 |
| A_55_P2137688 | ref|NM_009889|ens|ENSMUST00000029975|gb|J00643|tc|TC1585366 | Cga | -4.89 |
| A_55_P2057821 | ref|NM_029793|ens|ENSMUST00000039165|ens|ENSMUST00000112850|gb|BC037037 | Golga1 | -4.87 |
| A_55_P1969128 | ref|NM_178373|ens|ENSMUST00000113091|ens|ENSMUST00000032416|ens|ENSMUST00000113089 | Cidec | -4.82 |
| A_52_P575078 | ref|NM_026636|ens|ENSMUST00000002473|gb|AK077402|gb|AK003366 | 5430437P03Rik | -4.78 |
| A_55_P2051577 | ref|XM_001480146|ref|XM_001480240 | Gm4495 | -4.69 |
| A_55_P2100360 | ref|NM_080858|ens|ENSMUST00000033549|gb|AF403040|gb|AK009644 | Asb12 | -4.55 |
| A_55_P1987514 | ref|XM_001477297|ref|XM_001474105|ref|XM_001474121|ref|XM_001472131 | Gm11353 | -4.45 |
| A_51_P511418 | ref|NM_027699|ens|ENSMUST00000020853|gb|AK007140|tc|TC1590195 | 1700108M19Rik | -4.44 |
| A_55_P2024456 | ens|ENSMUST00000097954|gb|AK149896|riken|G530102J04 | ENSMUST00000097954 | -4.37 |
| A_55_P2045096 | ref|NM_198652|ens|ENSMUST00000127446|ens|ENSMUST00000054674|ref|XM_001473656 | Hjurp | -4.34 |
| A_55_P2074776 | ref|XM_993473|ref|XM_001476215 | Gm8894 | -4.29 |
| A_51_P104687 | ref|NM_008117|ens|ENSMUST00000103071|gb|X02891|gb|AK030419 | Gh | -4.28 |
| A_30_P01026116 | Unknown | chr1:71939741-71947891_F | -4.25 |
| A_55_P2111703 | ref|XM_981456 | Gm7921 | -4.22 |
| A_55_P1999033 | ens|ENSMUST00000103540|ref|XM_001475919|gb|U22990|gb|U22986 | LOC100046275 | -4.21 |
| A_51_P360880 | ref|NM_001082485|ref|NM_001135019|gb|AK088432|gb|BF101229 | 5730601F06Rik | -4.21 |
| A_55_P1962707 | ref|XR_033865|ref|XR_033878 | Gm5263 | -4.14 |
| A_66_P140200 | ref|NM_146631|ens|ENSMUST00000077498|gb|BC141645|gb|BC140275 | Olfr120 | -4.11 |
| A_30_P01023697 | Unknown | chr2:167388900-167402475_R | -4.10 |
| A_30_P01023694 | Unknown | chr17:13227704-13229876_F | -4.10 |
| A_55_P1970464 | ens|ENSMUST00000103507|ens|ENSMUST00000103504|ens|ENSMUST00000103550|ref|XM_908887 | ENSMUST00000103507 | -4.09 |
| A_55_P2150591 | ref|NM_177759|ens|ENSMUST00000111995|ens|ENSMUST00000086483|gb|BC150892 | Ccdc60 | -4.09 |
| A_55_P2089015 | ens|ENSMUST00000023920|gb|AK009779|tc|TC1585276|riken|2310043I08 | Tmem52 | -4.06 |
| A_55_P2088796 | ref|XR_033381|nap|NAP114227-1 | LOC100047261 | -3.94 |
| A_66_P123282 | ref|NM_001007460|gb|AY668949|gb|BC145527|gb|BC139052 | Zdhhc23 | -3.90 |
| A_30_P01021886 | Unknown | chr4:145450600-145461600_R | -3.88 |
| A_55_P2001154 | ref|XR_032843|gb|AV583749 | LOC637860 | -3.88 |
| A_55_P2134862 | ref|NM_001163262|ref|NM_028941|tc|TC1601894 | 4933407C03Rik | -3.86 |
| A_51_P275365 | ref|NM_146490|ens|ENSMUST00000073748|gb|AY053401|gb|BC128013 | Olfr1411 | -3.85 |
| A_30_P01030348 | Unknown | chr10:9322308-9332183_R | -3.81 |
| A_55_P2000254 | ref|NM_001163498|ens|ENSMUST00000115814|tc|TC1697538|nap|NAP069887-1 | Rundc2a | -3.80 |
| A_52_P618728 | ens|ENSMUST00000103362|gb|M61952|tc|NP061620|tc|TC1730176 | ENSMUST00000103362 | -3.75 |
| A_55_P2054315 | ref|NM_008571|ens|ENSMUST00000015576|gb|BC119248|gb|BC119250 | Mcpt2 | -3.74 |
| A_55_P1968428 | ref|XM_001474934|ref|XM_990534|ref|XM_001472244 | Gm7122 | -3.73 |
| A_55_P2052101 | ref|XM_001479774|ref|XR_035644|nap|NAP025077-001 | Gm6582 | -3.72 |
| A_51_P153995 | ref|NM_018762|ens|ENSMUST00000032133|gb|AB007464|tc|TC1586240 | Gp9 | -3.72 |
| A_55_P2349243 | gb|AK144371|riken|G630043O09 | Gm10776 | -3.72 |
| A_30_P01030257 | Unknown | chr14:61252837-61310872_F | -3.71 |
| A_55_P2094666 | ens|ENSMUST00000101606|gb|AK145354|riken|I0C0021B14 | ENSMUST00000101606 | -3.69 |
| A_51_P324704 | ref|NM_025606|ens|ENSMUST00000025586|gb|AK009687|gb|AK186416 | Mrpl16 | -3.64 |
| A_55_P2157403 | ens|ENSMUST00000092883|gb|AY512957|nap|NAP125253-1|nap|NAP054559-1 | ENSMUST00000092883 | -3.64 |
| A_30_P01031383 | Unknown | chr9:96670035-96682896_F | -3.64 |
| A_55_P2369534 | gb|AK141396|riken|C430049E01 | C430049E01Rik | -3.63 |
| A_51_P512157 | ref|NM_144798|ens|ENSMUST00000024870|gb|BC066162|gb|BC068169 | Slc30a6 | -3.61 |
| A_30_P01020955 | Unknown | chr3:26975323-26978719_R | -3.57 |
| A_30_P01030586 | Unknown | chr1:81854900-81906900_R | -3.57 |
| A_55_P2248806 | gb|AK015642|gb|BF461800|tc|TC1695412|riken|4930488B01 | 4930488B01Rik | -3.56 |
| A_52_P533707 | ref|NM_007389|ens|ENSMUST00000028515|ref|XM_001476441|gb|AK132589 | Chrna1 | -3.54 |
| A_55_P2132132 | ens|ENSMUST00000097531|gb|AK147021|gb|BY760880|riken|I920088N03 | Gm5532 | -3.53 |
| A_55_P2267367 | gb|AK019492|riken|4632411P08|nap|NAP073182-1 | 4632411P08Rik | -3.53 |
| A_30_P01018651 | Unknown | chr6:126502067-126507755_R | -3.51 |
| A_30_P01030538 | Unknown | chr15:90074814-90087814_F | -3.50 |
| A_55_P2058340 | ens|ENSMUST00000038776|gb|AK165551|gb|AK014651|gb|AK029349 | ENSMUST00000038776 | -3.49 |
| A_52_P487436 | ref|NM_145829|ref|NM_178053|ens|ENSMUST00000055409|gb|AJ489814 | Nags | -3.46 |
| A_30_P01025840 | Unknown | chr4:119811853-119832606_F | -3.46 |
| A_55_P2002058 | ens|ENSMUST00000112670|gb|AK147939|riken|G270110H15 | ENSMUST00000112670 | -3.43 |
| A_55_P1982896 | ref|NM_173014|ens|ENSMUST00000046290|gb|AK039431|tc|TC1580995 | Lpcat2 | -3.43 |
| A_55_P2145759 | ref|NM_177397|ens|ENSMUST00000027643|gb|AK032887|tc|TC1588575 | Atp6v1g3 | -3.43 |
| A_51_P307964 | ref|NM_010662|ens|ENSMUST00000107412|gb|BC060286|tc|TC1727877 | Krt13 | -3.43 |
| A_55_P1986743 | ref|XR_031573|ref|XR_032281 | LOC674800 | -3.43 |
| A_30_P01030369 | Unknown | chr18:70400082-70404236_R | -3.43 |
| A_55_P2145465 | ref|NM_177187|ens|ENSMUST00000094593|ref|XM_001473565|ref|XM_001472169 | D5Ertd577e | -3.43 |
| A_30_P01029048 | Unknown | chr5:118919606-118925333_R | -3.41 |
| A_55_P2156556 | ref|XM_001472797|nap|NAP112173-1|nap|NAP112209-1|nap|NAP058516-1 | Gm2212 | -3.40 |
| A_51_P292823 | ref|NR_024017|gb|AK019839|tc|TC1604963|riken|4930594C11 | 4930594C11Rik | -3.40 |
| A_51_P140207 | ref|NM_025601|ens|ENSMUST00000134023|gb|BC049575|gb|AK006340 | 1700029H14Rik | -3.39 |
| A_51_P164393 | ref|NM_153519|ref|NM_001146002|ens|ENSMUST00000050236|gb|AF196282 | Txndc2 | -3.39 |
| A_52_P139747 | ref|NM_019443|ens|ENSMUST00000016571|gb|Y07708|gb|AK131584 | Ndufa1 | -3.38 |
| A_55_P2041086 | ref|NM_001114102|tc|TC1662746 | 1110034B05Rik | -3.37 |
| A_55_P1966159 | ref|NM_001163513|ref|NM_027726|ens|ENSMUST00000090398|ens|ENSMUST00000073687 | Dlg5 | -3.37 |
| A_52_P146711 | ref|NM_009432|ref|NM_001165939|ref|NM_001165940|ens|ENSMUST00000029450 | Tshb | -3.36 |
| A_52_P273812 | ref|NM_053088|ens|ENSMUST00000026562|gb|EU380257|gb|AJ009781 | Ifitm5 | -3.35 |
| A_55_P1986706 | ref|NM_029406|ens|ENSMUST00000085375|ens|ENSMUST00000107811|ens|ENSMUST00000107813 | Pih1d1 | -3.33 |
| A_30_P01029809 | Unknown | chrX:34347467-34347843_R | -3.33 |
| A_55_P2186125 | ref|NM_028784|ref|NM_001166391|ens|ENSMUST00000095869|ens|ENSMUST00000099580 | F13a1 | -3.33 |
| A_55_P2083844 | ref|NM_001001295|ref|NM_001177784|ref|NM_172519|ens|ENSMUST00000120760 | Dis3l | -3.32 |
| A_55_P1964955 | ref|XM_001478025|ref|XM_001478341|gb|BC038278 | Gm3716 | -3.32 |
| A_30_P01026944 | Unknown | chr1:72297489-72298058_R | -3.32 |
| A_55_P2154132 | ref|NM_011654|ens|ENSMUST00000077577|ref|XM_486246|ref|XM_904657 | Tuba1b | -3.32 |
| A_55_P2078084 | ref|NM_001126318|ens|ENSMUST00000024200|ref|XR_034282|nap|NAP062013-1 | Gm13011 | -3.31 |
| A_55_P1982171 | ref|NM_016750|ens|ENSMUST00000041045|ref|XM_001000460|ref|XM_001480384 | H2afz | -3.31 |
| A_52_P547612 | ref|NM_178715|gb|AK030302|gb|AK169014|gb|AK037246 | Tmem30b | -3.30 |
| A_52_P359819 | ref|NM_198190|ens|ENSMUST00000058879|gb|AK019330|gb|BC052191 | Ntf5 | -3.30 |
| A_55_P2157799 | ref|NM_032394|ens|ENSMUST00000025251|ens|ENSMUST00000134663|gb|BC138341 | Myo7b | -3.30 |
| A_30_P01033329 | Unknown | chr6:11994002-11997859_F | -3.29 |
| A_52_P376269 | ref|NM_010938|ref|NM_001164228|ref|NM_001164227|ref|NM_001164229 | Nrf1 | -3.29 |
| A_55_P2063608 | ens|ENSMUST00000103655|ens|ENSMUST00000103592|ens|ENSMUST00000103618|ref|XM_001475238 | ENSMUST00000103655 | -3.28 |
| A_30_P01026095 | Unknown | chr7:128341797-128414692_R | -3.27 |
| A_55_P2097820 | ref|XR_002170|ref|XR_033117 | Gm9132 | -3.27 |
| A_66_P102143 | ens|ENSMUST00000103534|gb|AF303846|gb|AF303847|gb|X87728 | ENSMUST00000103534 | -3.26 |
| A_51_P467410 | ref|NM_008722|ens|ENSMUST00000093201|ens|ENSMUST00000075641|gb|AK165860 | Npm1 | -3.24 |
| A_51_P453736 | ref|NM_001143686|gb|AY662646|gb|AK039744|tc|TC1687342 | Apol11b | -3.24 |
| A_55_P2000224 | ref|NM_001042580|ref|NM_007653|ens|ENSMUST00000105229|ens|ENSMUST00000026407 | Cd63 | -3.24 |
| A_30_P01024134 | Unknown | chr2:60038543-60046318_R | -3.20 |
| A_55_P2158565 | ref|XM_001476513|nap|NAP070950-1 | LOC100046618 | -3.20 |
| A_51_P496400 | ref|NM_138675|ens|ENSMUST00000081980|gb|AF467888|gb|BC019367 | Med9 | -3.19 |
| A_55_P2037752 | ens|ENSMUST00000053770|gb|AK028402|tc|TC1594198|riken|3830432L08 | ENSMUST00000053770 | -3.19 |
| A_55_P2061796 | ref|XM_001475863|gb|AK007238|tc|TC1667925|riken|1700122H20 | 1700122H20Rik | -3.17 |
| A_55_P2137979 | ref|NM_177638|ens|ENSMUST00000097299|ens|ENSMUST00000112924|ens|ENSMUST00000071826 | Crb3 | -3.17 |
| A_55_P2151373 | ref|XM_905487 | Rpl19-ps4 | -3.17 |
| A_30_P01024296 | Unknown | chr9:7214753-7217212_F | -3.17 |
| A_55_P2127478 | ens|ENSMUST00000064857|gb|AK088220|tc|TC1704908|riken|E430007J01 | ENSMUST00000064857 | -3.16 |
| A_55_P2055732 | ref|XM_001472616 | LOC100044574 | -3.16 |
| A_55_P2045083 | ref|XR_035065 | Gm14864 | -3.15 |
| A_55_P2115435 | ref|NM_013638|ens|ENSMUST00000050864|gb|AK132894|gb|BC090986 | Prm3 | -3.15 |
| A_55_P2000489 | ref|NM_172523|ens|ENSMUST00000086765|ens|ENSMUST00000026084|gb|AK078096 | Slc18a2 | -3.14 |
| A_55_P2002908 | ref|NM_001001451|ens|ENSMUST00000076565|gb|BC128016|gb|AY916509 | Tas2r138 | -3.14 |
| A_55_P1959648 | ref|NM_008305|ens|ENSMUST00000097839|ens|ENSMUST00000030547|ref|XM_001477315 | Hspg2 | -3.13 |
| A_30_P01019307 | Unknown | chr4:88583425-88584070_R | -3.13 |
| A_55_P2016471 | ref|NM_021332|ens|ENSMUST00000114574|ref|XM_001471951|gb|BC103624 | Glp1r | -3.13 |
| A_30_P01026718 | Unknown | chr1:39763924-39774099_F | -3.13 |
| A_55_P2046220 | ref|NM_146667|ens|ENSMUST00000089838|tc|TC1600044|nap|NAP059883-1 | Olfr740 | -3.12 |
| A_30_P01020734 | Unknown | chr17:32213027-32228368_R | -3.12 |
| A_55_P2074864 | ref|XR_031978|ref|XR_033255 | Gm9435 | -3.12 |
| A_55_P1988571 | ens|ENSMUST00000103641|ens|ENSMUST00000103578|ens|ENSMUST00000103609|ens|ENSMUST00000103610 | ENSMUST00000103641 | -3.11 |
| A_30_P01019149 | Unknown | chr3:10081893-10094218_F | -3.11 |
| A_55_P2102002 | ref|NM_021315|ens|ENSMUST00000025963|gb|AK168944|riken|I920066M10 | Noc3l | -3.10 |
| A_55_P2249379 | gb|AK016549|riken|4932434L04|nap|NAP022832-001 | LOC100036523 | -3.10 |
| A_52_P314157 | ref|XM_001478109|ref|XM_001472971|gb|AK030470|gb|AK014217 | 3110054G05Rik | -3.09 |
| A_51_P356283 | ref|NM_133765|ens|ENSMUST00000034268|gb|AK009859|gb|AK140412 | Fbxo31 | -3.09 |
| A_30_P01020873 | Unknown | chr12:111079867-111080561_R | -3.09 |
| A_55_P2135261 | ref|NM_172730|gb|AK081505|riken|C130022K22|nap|NAP002772-003 | C130022K22Rik | -3.08 |
| A_30_P01023811 | Unknown | chr3:65729664-65731697_R | -3.08 |
| A_55_P1988918 | ens|ENSMUST00000091674|gb|AK045506|tc|TC1614951|riken|B230207N07 | ENSMUST00000091674 | -3.07 |
| A_55_P1996374 | ref|NM_001081240|ens|ENSMUST00000118622|ens|ENSMUST00000056237|gb|AK045315 | Prmt10 | -3.07 |
| A_55_P2163003 | ref|NM_030596|ens|ENSMUST00000070892|gb|AK148657|tc|TC1684209 | Dsg3 | -3.07 |
| A_55_P1959858 | ref|NM_021405|ens|ENSMUST00000109939|ens|ENSMUST00000047008|ens|ENSMUST00000109938 | Cst10 | -3.06 |
| A_55_P1994309 | ref|NM_008967|ens|ENSMUST00000086101|ens|ENSMUST00000060386|ens|ENSMUST00000144408 | Ptgir | -3.06 |
| A_30_P01017519 | Unknown | chr4:40202350-40205120_R | -3.06 |
| A_55_P2011794 | ref|XM_911832 | LOC636441 | -3.05 |
| A_55_P1956598 | ref|XR_033948|gb|BC019489|gb|BC012207|gb|BC018365 | LOC100047788 | -3.04 |
| A_55_P2088360 | ref|XM_355309|ref|XM_909470|nap|NAP101660-1 | Gm13194 | -3.04 |
| A_55_P2073121 | ref|NM_146024|ens|ENSMUST00000021227|gb|AK017451|tc|TC1755851 | Ankrd40 | -3.04 |
| A_55_P2045936 | ref|XM_897643|ref|XM_922733|gb|AK015502|tc|NP742320 | 4930465A12Rik | -3.04 |
| A_55_P1960688 | ref|NM_177392|ens|ENSMUST00000079077|ens|ENSMUST00000061786|gb|AK043094 | Tmem220 | -3.04 |
| A_55_P1967025 | ref|XM_001473268|gb|AK143106|riken|E130104E13 | LOC100044696 | -3.04 |
| A_55_P2061135 | ref|NM_001002239|ens|ENSMUST00000100558|ens|ENSMUST00000091931|ens|ENSMUST00000085340 | Rpl17 | -3.03 |
| A_30_P01017817 | Unknown | chr2:84344350-84370075_F | -3.03 |
| A_52_P140356 | ref|NM_007590|ens|ENSMUST00000019514|gb|AK165568|gb|AK049104 | Calm3 | -3.02 |
| A_66_P113188 | ens|ENSMUST00000103492|gb|EU568202|gb|AF006586|gb|EF492992 | ENSMUST00000103492 | -3.02 |
| A_52_P237048 | ref|NR_024139|ens|ENSMUST00000061464|gb|AK087608|gb|AK157132 | Prmt6 | -3.00 |
| A_51_P307370 | ref|NM_011305|ens|ENSMUST00000113934|ens|ENSMUST00000100251|ens|ENSMUST00000077257 | Rxra | -3.00 |
| A_55_P2031397 | ens|ENSMUST00000101255|gb|AK136626|tc|NP1484759|riken|9230009I02 | ENSMUST00000101255 | -3.00 |
| A_51_P138772 | ref|NM_153065|ens|ENSMUST00000018143|gb|AK134274|gb|BC011321 | Ddx27 | -3.00 |
| A_30_P01022761 | Unknown | chr11:102878974-102888924_F | -3.00 |
| A_55_P2337073 | ref|NM_183151|ref|NM_010797|ens|ENSMUST00000112107|ens|ENSMUST00000112105 | Mid1 | -3.00 |
| A_55_P2046524 | ref|NM_146274|ens|ENSMUST00000099601|gb|BC127967|tc|NP647030 | Olfr1311 | -2.99 |
| A_52_P83623 | ref|NM_175258|ens|ENSMUST00000108895|ens|ENSMUST00000094536|ens|ENSMUST00000050145 | Rapgef6 | -2.99 |
| A_55_P1954658 | ref|XM_487581|ref|XM_914336|nap|NAP070792-1 | Gm5695 | -2.99 |
| A_55_P2149540 | ref|NM_020493|ens|ENSMUST00000015749|gb|BC051950|tc|TC1577357 | Srf | -2.98 |
| A_55_P2005005 | ref|NM_025876|ens|ENSMUST00000109731|ens|ENSMUST00000028990|gb|AK010081 | Cdk5rap1 | -2.97 |
| A_55_P2243162 | gb|BC049679|tc|TC1645926 | BC049679 | -2.97 |
| A_30_P01024260 | Unknown | chr8:26428975-26490975_F | -2.96 |
| A_51_P486188 | ref|NM_011033|ens|ENSMUST00000063219|gb|X75959|gb|AK077154 | Pabpc2 | -2.96 |
| A_55_P2117863 | ref|NM_134240|ens|ENSMUST00000077116|gb|BC120858|gb|BC120860 | Vmn1r222 | -2.95 |
| A_55_P2362391 | ref|NR_015604|gb|AK087434|riken|E130112N10 | E130112N10Rik | -2.95 |
| A_51_P377228 | ens|ENSMUST00000067468|gb|AK044378|tc|TC1590676|riken|A930010C09 | ENSMUST00000067468 | -2.95 |
| A_55_P2021460 | ref|XM_906502|ref|XM_992620|nap|NAP113591-1 | Gm1096 | -2.95 |
| A_55_P2110210 | ref|XM_978106|ref|XM_993976|tc|TC1609259 | XM_978106 | -2.94 |
| A_52_P503334 | ref|NM_153592|ens|ENSMUST00000033873|gb|AK160885|gb|AK160391 | Erlin2 | -2.94 |
| A_55_P1955039 | ref|NM_001161817|ref|NM_010863|ens|ENSMUST00000018561|ens|ENSMUST00000114541 | Myo1b | -2.94 |
| A_52_P520341 | ens|ENSMUST00000051742|gb|AK088084|gb|AK037526|tc|TC1610314 | ENSMUST00000051742 | -2.93 |
| A_52_P562267 | ref|NM_001033819|ens|ENSMUST00000036819|gb|BC147378|gb|BC147379 | 9130409I23Rik | -2.93 |
| A_55_P2028029 | ref|NM_021022|ens|ENSMUST00000102709|ens|ENSMUST00000102710|gb|AK144553 | Abcb11 | -2.93 |
| A_52_P192084 | ref|NM_030231|ref|NM_001048189|ens|ENSMUST00000097920|ens|ENSMUST00000080744 | Agbl4 | -2.93 |
| A_66_P112109 | ref|NR_030681|gb|AK087307|tc|TC1617241|riken|E030043B18 | Gm4598 | -2.92 |
| A_52_P158376 | ref|NM_027926|ens|ENSMUST00000049251|gb|AK030705|gb|BC061206 | Cpa4 | -2.92 |
| A_55_P2257306 | gb|AK015045|tc|TC1649531|riken|4930402D18|nap|NAP073968-1 | 4930402D18Rik | -2.92 |
| A_55_P2111247 | ref|NM_147004|ens|ENSMUST00000059440|gb|BC120820|gb|BC120846 | Olfr399 | -2.92 |
| A_55_P2304602 | gb|BX513744 | AA414903 | -2.91 |
| A_51_P375969 | ref|NM_053200|ens|ENSMUST00000034172|gb|AK078879|gb|BC019198 | Ces3 | -2.91 |
| A_30_P01027879 | Unknown | chr6:47959825-47960502_R | -2.91 |
| A_66_P136857 | ref|NM_001081118|ens|ENSMUST00000155123|ens|ENSMUST00000130687|ens|ENSMUST00000142572 | Phrf1 | -2.91 |
| A_51_P512960 | ens|ENSMUST00000064445|gb|AK083698|tc|TC1593629|riken|D030068E18 | ENSMUST00000064445 | -2.89 |
| A_66_P123196 | ens|ENSMUST00000071072|gb|AK037606|gb|BB156127|gb|AI850956 | ENSMUST00000071072 | -2.89 |
| A_51_P449995 | ref|NM_016704|ens|ENSMUST00000022788|gb|AF184900|gb|BC011251 | C6 | -2.89 |
| A_55_P2134571 | ref|XM_973961|ref|XM_139814|tc|TC1674325 | Gm4944 | -2.89 |
| A_52_P625912 | ref|NM_153094|ref|NR_024262|ref|NR_024263|ens|ENSMUST00000032257 | Klrb1f | -2.88 |
| A_52_P670188 | ref|NM_027301|ens|ENSMUST00000047134|gb|BC064820 | Sdr9c7 | -2.88 |
| A_30_P01027382 | Unknown | chr7:49829800-49843475_F | -2.87 |
| A_55_P2080178 | ref|NR_033473|ens|ENSMUST00000077779|gb|AK145809|riken|I1C0048O07 | Gm13939 | -2.87 |
| A_55_P2062024 | ens|ENSMUST00000072628|gb|AK053725|tc|TC1585380|riken|E130302P05 | ENSMUST00000072628 | -2.87 |
| A_30_P01029603 | Unknown | chr11:16831925-16851121_F | -2.86 |
| A_55_P2280316 | gb|AK017489|riken|5730405A17|nap|NAP073019-1 | 5730405A17Rik | -2.86 |
| A_55_P2066894 | ens|ENSMUST00000103442|gb|AY172004|gb|AF547085|gb|AY172005 | ENSMUST00000103442 | -2.86 |
| A_55_P2045512 | ref|XR_034819|ref|XR_031213 | Gm9276 | -2.86 |
| A_55_P1972590 | ref|NM_173396|ens|ENSMUST00000081335|ens|ENSMUST00000109564|ens|ENSMUST00000073352 | Tgif2 | -2.85 |
| A_51_P462790 | ens|ENSMUST00000112293|gb|AK007130|riken|1700106N22|nap|NAP014581-001 | ENSMUST00000112293 | -2.85 |
| A_55_P2296641 | ens|ENSMUST00000094228|gb|AK015427|tc|TC1596934|riken|4930449A18 | ENSMUST00000094228 | -2.85 |
| A_55_P2057085 | ref|XM_001472692|ref|XM_001472849 | LOC100039329 | -2.85 |
| A_55_P2112142 | ens|ENSMUST00000115515|gb|AK019559|tc|TC1726062|riken|4930403J02 | ENSMUST00000115515 | -2.85 |
| A_51_P221753 | ref|NM_010116|ens|ENSMUST00000081399|gb|M17962|gb|BC024624 | Klk1b9 | -2.85 |
| A_55_P2020726 | ref|XM_001472218|ref|XM_001472410 | Gm16505 | -2.84 |
| A_55_P2269814 | gb|AK016769|tc|TC1624105|riken|4933411E08|nap|NAP073468-1 | 4933411E08Rik | -2.84 |
| A_55_P2074942 | ref|NM_010484|ens|ENSMUST00000021195|ens|ENSMUST00000108402|gb|AK008410 | Slc6a4 | -2.84 |
| A_51_P315555 | ref|NM_027350|ref|NM_001142950|ens|ENSMUST00000025483|gb|AK077699 | Nars | -2.84 |
| A_66_P139646 | ref|NM_001168504|ref|NM_001004762|ens|ENSMUST00000043612|ens|ENSMUST00000108528 | Pla2g4c | -2.83 |
| A_30_P01024264 | Unknown | chr14:99734507-99734889_R | -2.82 |
| A_52_P633481 | ref|NM_001164503|ens|ENSMUST00000022593|ens|ENSMUST00000123853|gb|AK032821 | Akap11 | -2.82 |
| A_52_P434974 | ref|NM_152234|ref|NM_026842|ens|ENSMUST00000058735|ens|ENSMUST00000076454 | Ubqln1 | -2.82 |
| A_55_P1989081 | ens|ENSMUST00000104986 | ENSMUST00000104986 | -2.82 |
| A_55_P2065455 | ref|NM_009646|ens|ENSMUST00000143735|ens|ENSMUST00000019257|ens|ENSMUST00000128241 | Aire | -2.82 |
| A_30_P01022399 | Unknown | chr5:13125413-13310754_F | -2.81 |
| A_55_P2011557 | ens|ENSMUST00000097699|ens|ENSMUST00000150198|ref|XM_001476401|ref|XM_001478864 | ENSMUST00000097699 | -2.80 |
| A_55_P2112135 | ref|XM_001471959|ref|XM_973024|ref|XM_001473240 | Gm1979 | -2.80 |
| A_30_P01028328 | Unknown | chrX:135880775-135891525_F | -2.80 |
| A_51_P138859 | ref|NM_028097|ens|ENSMUST00000154922|ens|ENSMUST00000029891|gb|AK088600 | Tmem68 | -2.80 |
| A_55_P2044557 | ref|XM_001472583|ref|XM_001473311|ref|XM_001473900|ref|XM_001478364 | LOC100044494 | -2.80 |
| A_55_P2016962 | ref|XR_004604 | LOC674135 | -2.79 |
| A_30_P01026926 | Unknown | chr8:71495043-71525582_R | -2.79 |
| A_55_P2037862 | ref|XM_001476437|gb|AK154753|tc|TC1742881|riken|F630105L23 | LOC100041476 | -2.79 |
| A_55_P2120856 | ens|ENSMUST00000066461 | ENSMUST00000066461 | -2.79 |
| A_55_P2035817 | ref|XM_989078|ref|XM_355598 | Gm1043 | -2.78 |
| A_55_P2284322 | gb|AK139166|riken|A730057B05 | 4930444A19Rik | -2.78 |
| A_66_P128440 | ref|XM_973091|ref|XM_973277|gb|AK029472|riken|4833442K13 | BB094273 | -2.78 |
| A_55_P2070326 | ref|NM_146280|ens|ENSMUST00000054181|ens|ENSMUST00000109163|gb|BC120834 | Olfr281 | -2.78 |
| A_66_P122837 | ref|NM_001005419|ens|ENSMUST00000075686|gb|AK155334|gb|BC057106 | Ado | -2.77 |
| A_30_P01019357 | Unknown | chr3:48906985-48957532_R | -2.77 |
| A_66_P103052 | ref|XM_897430|ref|XM_921604|nap|NAP058187-1 | Gm6282 | -2.77 |
| A_55_P2389714 | gb|AK020418|tc|TC1614280|riken|9430014N10|nap|NAP078385-1 | 9430014N10Rik | -2.77 |
| A_55_P2130198 | ref|NM_172515|ens|ENSMUST00000107775|ens|ENSMUST00000107776|ens|ENSMUST00000039269 | Zbbx | -2.77 |
| A_55_P1991275 | ref|XM_001480022 | Gm4385 | -2.76 |
| A_30_P01023011 | Unknown | chr16:4867322-4874772_R | -2.76 |
| A_51_P386270 | ref|NM_201360|ens|ENSMUST00000068861|gb|BC139169|gb|BC139170 | Cyp2d12 | -2.76 |
| A_55_P2159875 | ref|NM_011760|ens|ENSMUST00000115548|ref|XM_001471671|gb|BC080782 | Zfp54 | -2.76 |
| A_30_P01020701 | Unknown | chr13:19611679-19620954_R | -2.75 |
| A_55_P1963553 | ref|NM_207548|ens|ENSMUST00000098702|tc|TC1595833|nap|NAP111443-1 | Vmn1r174 | -2.75 |
| A_55_P2055487 | ref|NM_145619|ens|ENSMUST00000067218|gb|AF368233|gb|AK147868 | Parp3 | -2.75 |
| A_55_P2201733 | ref|NM_001145759|gb|AK016274|gb|BC048566|tc|TC1633902 | 4930571K23Rik | -2.75 |
| A_30_P01028320 | Unknown | chr10:126151060-126156450_R | -2.74 |
| A_55_P2085592 | ens|ENSMUST00000110825|tc|TC1758852 | ENSMUST00000110825 | -2.74 |
| A_55_P2116435 | ref|NM_010951|ens|ENSMUST00000026383|nap|NAP099407-001 | Gpr143 | -2.74 |
| A_66_P122155 | ref|NM_145514|ens|ENSMUST00000111053|ens|ENSMUST00000036329|ref|XM_001474668 | Wdr26 | -2.74 |
| A_55_P2373423 | ref|NM_001079690|ref|NM_183354|ens|ENSMUST00000110495|ens|ENSMUST00000110494 | Slc12a1 | -2.74 |
| A_30_P01020782 | Unknown | chr4:88582120-88582754_F | -2.73 |
| A_51_P377452 | ref|NM_008677|ens|ENSMUST00000096357|gb|AK171315|gb|AB002665 | Ncf4 | -2.73 |
| A_55_P1952618 | ref|NM_007895|ref|NM_007894|ens|ENSMUST00000074839|ens|ENSMUST00000100691 | Ear2 | -2.73 |
| A_30_P01029523 | Unknown | chr16:21794415-21815365_R | -2.73 |
| A_66_P108733 | ens|ENSMUST00000054105|nap|NAP029229-1 | ENSMUST00000054105 | -2.73 |
| A_55_P2200429 | gb|AK015187|tc|TC1623240|riken|4930423D22|nap|NAP074040-1 | 4930423D22Rik | -2.72 |
| A_30_P01025842 | Unknown | chr10:84005221-84022896_F | -2.72 |
| A_30_P01026324 | Unknown | chr4:149383134-149451259_R | -2.71 |
| A_55_P2167565 | ref|XR_002354|ref|XR_035366 | A330068G13Rik | -2.71 |
| A_55_P2004471 | ens|ENSMUST00000072612|ref|XM_912293|ref|XM_978968|ref|XM_486266 | ENSMUST00000072612 | -2.71 |
| A_55_P2257765 | gb|AK078161|tc|TC1616796|riken|6330586M15|nap|NAP124386-1 | Gm7111 | -2.71 |
| A_55_P1998616 | ref|XM_001478663|gb|AK038843|tc|TC1628594|riken|A230067N12 | Gm3983 | -2.70 |
| A_55_P2009037 | Unknown | A_55_P2009037 | -2.70 |
| A_55_P2162797 | ref|XM_001480165 | LOC100048401 | -2.70 |
| A_55_P2084706 | ref|XR_033425|gb|BC023946|gb|BC049923 | Gm5182 | -2.70 |
| A_55_P2279498 | gb|AK017111|tc|TC1610556|riken|4933438K21|nap|NAP073621-1 | 4933438K21Rik | -2.70 |
| A_55_P2012071 | ens|ENSMUST00000101619|gb|BB229236 | ENSMUST00000101619 | -2.70 |
| A_55_P2053918 | ref|XM_001472566 | LOC100044556 | -2.69 |
| A_66_P115389 | ref|XM_001476916|gb|BB558042|nap|NAP096703-001 | LOC100046855 | -2.69 |
| A_55_P2182945 | ref|XM_978072|ref|XM_895862|tc|TC1671545 | Gm6154 | -2.69 |
| A_52_P633560 | ref|NM_013611|ens|ENSMUST00000049339|gb|X70514|gb|AK049290 | Nodal | -2.69 |
| A_30_P01021434 | Unknown | chr16:91174611-91200786_F | -2.69 |
| A_55_P1961265 | ref|XM_914561 | LOC638517 | -2.69 |
| A_51_P333896 | ref|NM_146471|ens|ENSMUST00000078932|gb|BC104066|gb|BC104067 | Olfr1393 | -2.69 |
| A_55_P2084915 | ref|XM_001479136|gb|AK047213|gb|BB342285|tc|TC1606331 | LOC100047923 | -2.69 |
| A_30_P01018716 | Unknown | chr12:106572079-106575298_R | -2.69 |
| A_55_P2194908 | gb|AU022717 | D7Ertd558e | -2.68 |
| A_51_P505823 | ref|NM_028013|ens|ENSMUST00000041780|gb|AK173036|gb|AK078056 | Endod1 | -2.68 |
| A_55_P2000182 | ref|NM_001177621|ref|NM_177870|ref|NM_001177622|ens|ENSMUST00000114668 | Slc5a6 | -2.68 |
| A_52_P206998 | ref|XM_001473915|gb|AK029093|riken|4732490P15 | LOC100045240 | -2.67 |
| A_51_P187625 | ref|NM_013878|ref|NM_001160253|ref|NM_001160252|ens|ENSMUST00000025785 | Cabp2 | -2.67 |
| A_51_P306066 | ref|NM_013507|ref|NM_001040131|ens|ENSMUST00000005750|ens|ENSMUST00000106666 | Eif4g2 | -2.67 |
| A_55_P1960522 | ens|ENSMUST00000109473|ens|ENSMUST00000109472|gb|BY109983 | ENSMUST00000109473 | -2.67 |
| A_55_P2112459 | ref|NM_029383|ens|ENSMUST00000038693|gb|AK008821|gb|AV360254 | Cldn22 | -2.67 |
| A_51_P334072 | ref|NM_009989|ens|ENSMUST00000028430|gb|M20625|gb|AK018833 | Cyct | -2.67 |
| A_55_P2249963 | gb|AW542521 | AA408865 | -2.66 |
| A_55_P1973264 | ref|NM_001040055|ens|ENSMUST00000103097|gb|BC147380|gb|BC147381 | Gm14316 | -2.66 |
| A_30_P01024993 | Unknown | chr1:167218236-167218418_F | -2.66 |
| A_52_P412585 | ref|NM_007894|ens|ENSMUST00000100691|gb|U72032|gb|BC117060 | Ear1 | -2.65 |
| A_55_P2156533 | ref|XM_001479074|ref|XM_001479643 | Gm4265 | -2.65 |
| A_51_P373619 | ref|NM_010775|ens|ENSMUST00000047095|gb|D11441|gb|BC012245 | Mbl1 | -2.65 |
| A_30_P01018990 | Unknown | chr5:123582742-123588202_F | -2.65 |
| A_55_P2141909 | ref|XM_001472440|ref|XM_001472597|ref|XM_001472205|ref|XM_001472389 | LOC100044499 | -2.65 |
| A_51_P295034 | ref|NM_010915|ens|ENSMUST00000077354|gb|M11434|gb|BC034518 | Klk1b4 | -2.65 |
| A_55_P2113936 | ref|XM_001472542|ref|XM_001477248|gb|AK141947|riken|D130073C15 | Gm9776 | -2.65 |
| A_55_P1960683 | ref|NM_010168|ens|ENSMUST00000111335|ens|ENSMUST00000028681|gb|AK167532 | F2 | -2.64 |
| A_55_P2023577 | ens|ENSMUST00000109747|gb|AK090199|tc|TC1610716|riken|G630016F24 | ENSMUST00000109747 | -2.64 |
| A_55_P2180454 | ref|XM_001474560|ref|XM_001473512|ref|XM_001474580|nap|NAP063315-1 | Gm2739 | -2.64 |
| A_55_P2005310 | ens|ENSMUST00000054846|gb|AK083240|tc|TC1593789|riken|C630030K01 | ENSMUST00000054846 | -2.64 |
| A_30_P01024804 | Unknown | chr8:50456266-50587338_F | -2.64 |
| A_30_P01033001 | Unknown | chr10:16603950-16794075_F | -2.64 |
| A_55_P2086323 | ref|XM_987111|ref|XM_981215|gb|BC051408 | BC051408 | -2.63 |
| A_55_P2078494 | ref|NM_001080812|ens|ENSMUST00000098630|gb|BC120812|gb|BC120814 | Cib3 | -2.63 |
| A_30_P01028115 | Unknown | chr9:27153416-27153677_F | -2.63 |
| A_55_P2013406 | ref|XM_001479773 | Gm4300 | -2.62 |
| A_30_P01025574 | Unknown | chr15:79795261-79796945_R | -2.62 |
| A_30_P01028523 | Unknown | chr16:30195156-30200385_F | -2.62 |
| A_55_P2326647 | gb|AK035630|tc|TC1608979|riken|9530078M16|nap|NAP017286-001 | 9530078M16Rik | -2.62 |
| A_51_P470414 | ref|NM_138600|ref|NM_001127338|gb|AK004991|tc|TC1583648 | Aldh7a1 | -2.61 |
| A_55_P2152225 | ref|NM_010544|ens|ENSMUST00000006713|gb|AK144322|gb|BC046984 | Ihh | -2.61 |
| A_55_P2378827 | gb|AK047890|tc|TC1662410|riken|C130020E14 | LOC553096 | -2.61 |
| A_55_P2060445 | ref|NM_026511|ens|ENSMUST00000040519|ref|XM_001477284|gb|AK012648 | 2810002N01Rik | -2.61 |
| A_30_P01017648 | Unknown | chr4:136735305-136735523_F | -2.61 |
| A_30_P01021821 | Unknown | chr2:153325670-153352170_R | -2.61 |
| A_55_P1969392 | ref|NM_026594|ens|ENSMUST00000121292|ens|ENSMUST00000044103|gb|AK005645 | Rpl39l | -2.60 |
| A_30_P01029351 | Unknown | chr16:9129538-9137898_R | -2.60 |
| A_30_P01020419 | Unknown | chr3:57414750-57427825_F | -2.60 |
| A_51_P478722 | ref|NM_011579|ref|NM_001145164|ens|ENSMUST00000068063|ens|ENSMUST00000046745 | Tgtp1 | -2.60 |
| A_30_P01029251 | Unknown | chr5:33174092-33200792_F | -2.60 |
| A_55_P2159114 | ref|NM_146281|ens|ENSMUST00000063289|gb|BC111907|gb|BC152883 | Olfr284 | -2.60 |
| A_52_P557170 | ref|NM_146437|ens|ENSMUST00000077075|gb|BC120782|gb|BC120784 | Olfr996 | -2.60 |
| A_55_P2108077 | ens|ENSMUST00000103275|gb|M13677|gb|AF481157|gb|AF481151 | ENSMUST00000103275 | -2.59 |
| A_66_P106289 | ref|NM_139061|ens|ENSMUST00000094375|ens|ENSMUST00000006221|gb|AF424699 | Vps54 | -2.59 |
| A_55_P2084990 | ens|ENSMUST00000114828|ens|ENSMUST00000129769|ens|ENSMUST00000123034|ens|ENSMUST00000068983 | ENSMUST00000114828 | -2.59 |
| A_51_P418432 | ref|NM_026253|ref|NM_001146021|ens|ENSMUST00000038956|ens|ENSMUST00000120866 | Lrrc18 | -2.59 |
| A_66_P130759 | ens|ENSMUST00000111270|ens|ENSMUST00000099699|gb|AK028444|gb|BC100298 | ENSMUST00000111270 | -2.59 |
| A_55_P2092501 | ref|NM_134027|ens|ENSMUST00000092735|ens|ENSMUST00000107545|gb|AK135480 | Med1 | -2.59 |
| A_55_P2063770 | ref|NM_008674|ens|ENSMUST00000070514|gb|AK077364|tc|TC1591862 | Nat3 | -2.58 |
| A_30_P01018311 | Unknown | chr2:181664903-181665707_R | -2.58 |
| A_51_P208435 | ref|NM_029858|ens|ENSMUST00000150023|ens|ENSMUST00000064035|gb|AK029878 | Ston1 | -2.58 |
| A_30_P01019129 | Unknown | chr15:86035939-86042539_R | -2.57 |
| A_55_P2170335 | ref|NM_007552|ens|ENSMUST00000051929|ens|ENSMUST00000028071|ens|ENSMUST00000112739 | Bmi1 | -2.57 |
| A_30_P01019860 | Unknown | chr8:41730287-41746862_R | -2.57 |
| A_30_P01022299 | Unknown | chr13:94194458-94195068_R | -2.57 |
| A_55_P2014328 | ref|XM_001479296 | LOC100047736 | -2.57 |
| A_51_P320444 | ref|NM_009072|ens|ENSMUST00000116446|ens|ENSMUST00000020904|gb|U58513 | Rock2 | -2.57 |
| A_51_P417815 | ref|NM_153067|ens|ENSMUST00000046797|gb|AY042193|tc|TC1596770 | Mrgpra3 | -2.57 |
| A_52_P521882 | ref|NM_026812|ens|ENSMUST00000032747|gb|AK004078|gb|BC038310 | Hddc3 | -2.57 |
| A_51_P201982 | ref|NM_007426|ens|ENSMUST00000033846|gb|AK019860|gb|AK048622 | Angpt2 | -2.57 |
| A_30_P01033055 | Unknown | chr2:174897225-174920850_R | -2.56 |
| A_30_P01018392 | Unknown | chr10:81589292-81626369_R | -2.56 |
| A_55_P2053597 | ref|XR_031259|ref|XR_033241 | Gm8467 | -2.56 |
| A_55_P1999576 | ref|NM_028792|ens|ENSMUST00000023061|gb|AK004913|gb|AK154799 | Josd1 | -2.56 |
| A_51_P482148 | ref|NM_007963|ens|ENSMUST00000029230|ens|ENSMUST00000108270|ens|ENSMUST00000108271 | Mecom | -2.56 |
| A_55_P2307496 | gb|AK041010|tc|TC1615397|riken|A530060J09 | LOC620306 | -2.56 |
| A_30_P01021658 | Unknown | chr18:35974770-35975091_F | -2.56 |
| A_55_P2025006 | ref|NM_010644|ref|NM_010115|ens|ENSMUST00000048945|ref|XR_032310 | Klk1b26 | -2.55 |
| A_55_P2066931 | ref|XM_001476950 | LOC100046868 | -2.55 |
| A_51_P134785 | ref|NM_146771|ens|ENSMUST00000057439|gb|BC127992|tc|NP646818 | Olfr1176 | -2.55 |
| A_55_P1969201 | ref|XM_001474377 | LOC100045481 | -2.55 |
| A_55_P2119687 | ref|XM_001472715|ref|XM_001472770 | LOC100044618 | -2.55 |
| A_51_P217336 | ref|NM_029153|ens|ENSMUST00000022197|gb|AK154671|gb|BC002185 | Scamp1 | -2.55 |
| A_55_P1954101 | ref|XM_143339 | Wdr49 | -2.55 |
| A_55_P2069781 | ref|XM_988096 | LOC676203 | -2.55 |
| A_55_P2169980 | ens|ENSMUST00000115297|tc|TC1656617 | ENSMUST00000115297 | -2.55 |
| A_55_P2029217 | ens|ENSMUST00000103573|ens|ENSMUST00000103571|ref|XM_990610|gb|U21455 | ENSMUST00000103573 | -2.55 |
| A_30_P01017452 | Unknown | chr3:30064327-30064680_R | -2.54 |
| A_55_P1992398 | ens|ENSMUST00000064487|gb|AK048705|tc|TC1655136|riken|C230012D11 | ENSMUST00000064487 | -2.54 |
| A_30_P01020010 | Unknown | chr12:25866632-25874507_F | -2.54 |
| A_55_P2006148 | ref|NM_001177438|ens|ENSMUST00000100032|ens|ENSMUST00000143380|ref|XM_974140 | Aldh3b2 | -2.54 |
| A_55_P2053384 | ref|NM_146921|ens|ENSMUST00000120303|ens|ENSMUST00000092924|gb|BC141608 | Olfr1 | -2.54 |
| A_55_P2225772 | gb|AK005722|tc|NP746825|riken|1700007J10|nap|NAP090523-1 | 1700007J10Rik | -2.54 |
| A_30_P01020274 | Unknown | chr8:59994074-60002058_R | -2.54 |
| A_55_P2012141 | ref|NM_177061|ens|ENSMUST00000054781|gb|BC147050|gb|BC147051 | Olfr1344 | -2.54 |
| A_30_P01025325 | Unknown | chr6:89207813-89209131_R | -2.53 |
| A_55_P2010401 | ens|ENSMUST00000079975|gb|AF038896|tc|NP049127 | ENSMUST00000079975 | -2.53 |
| A_55_P2177904 | ens|ENSMUST00000025070|gb|AK016156|tc|TC1593413|riken|4930557B01 | ENSMUST00000025070 | -2.53 |
| A_55_P2015887 | ens|ENSMUST00000109107 | ENSMUST00000109107 | -2.53 |
| A_55_P2175296 | ref|XR_031690|ref|XR_034139 | Gm9480 | -2.53 |
| A_55_P1955252 | ref|XM_001474354 | Gm2678 | -2.53 |
| A_51_P296429 | ref|NM_025781|ens|ENSMUST00000034431|gb|AK018496|tc|TC1585690 | Tmem170 | -2.52 |
| A_55_P2039086 | ref|NM_030612|ref|NM_001159394|ref|NM_001159395|ens|ENSMUST00000096026 | Nfkbiz | -2.52 |
| A_55_P1952854 | ref|XR_032273|ref|XR_034448 | Gm12960 | -2.52 |
| A_55_P1987284 | ref|NM_207574|ens|ENSMUST00000077143|ens|ENSMUST00000077164|gb|BC127965 | Olfr1383 | -2.52 |
| A_55_P2096076 | ref|XM_985614|nap|NAP101223-1 | Gm8256 | -2.51 |
| A_52_P589169 | ref|NM_028079|ens|ENSMUST00000091560|gb|AK141931|gb|BC048855 | 2010111I01Rik | -2.51 |
| A_66_P127022 | ref|NM_001081446|ens|ENSMUST00000152136|ens|ENSMUST00000106547|ens|ENSMUST00000106546 | Iqck | -2.51 |
| A_55_P2162880 | ref|NM_001100180|ens|ENSMUST00000079268|nap|NAP062269-1|nap|NAP027004-1 | Cyp3a57 | -2.51 |
| A_55_P2053404 | ens|ENSMUST00000070650|gb|AK170618|riken|F630110L20 | ENSMUST00000070650 | -2.51 |
| A_55_P1952725 | ref|XM_916981 | LOC640171 | -2.50 |
| A_55_P2288524 | ens|ENSMUST00000136396|ens|ENSMUST00000066533|ens|ENSMUST00000154818|ens|ENSMUST00000114762 | ENSMUST00000136396 | -2.50 |
| A_30_P01018519 | Unknown | chr11:22659730-22699427_F | -2.50 |
| A_30_P01019991 | Unknown | chr14:19295319-19310619_R | -2.50 |
| A_55_P2472435 | ref|NM_018734|ens|ENSMUST00000106222|ens|ENSMUST00000106221|ens|ENSMUST00000029935 | Gbp3 | -2.50 |
| A_55_P1959122 | ref|XM_001476142|tc|TC1760362 | LOC100046390 | -2.50 |
| A_30_P01022371 | Unknown | chr1:72983462-72987115_F | -2.50 |
| A_55_P2041868 | ref|NM_001081211 | Ptafr | -2.50 |
| A_51_P469568 | ref|NM_020504|ens|ENSMUST00000008987|gb|BC115481|gb|BC115482 | Cldn13 | -2.50 |
| A_55_P1977570 | ref|XM_001473156|ref|XM_001477363|ref|XM_001477451|ref|XM_001472587 | Gm2259 | -2.50 |
| A_30_P01028312 | Unknown | chr10:24273299-24277089_F | -2.50 |
| A_55_P2122329 | ref|NM_008688|ref|NM_026756|ens|ENSMUST00000116053|ens|ENSMUST00000105321 | Nfic | -2.49 |
| A_51_P423709 | ref|NM_029007|ens|ENSMUST00000020926|gb|BC002154|gb|BC005488 | Fam84a | -2.49 |
| A_55_P2018616 | ref|XM_001478384|ref|XM_001479640|ref|XM_001471582|gb|BC126859 | LOC100044027 | -2.49 |
| A_30_P01020850 | Unknown | chr7:46705675-46735750_R | -2.49 |
| A_55_P2020148 | ref|NM_001001792|ref|NM_008616|ens|ENSMUST00000112857|ens|ENSMUST00000079405 | Zfp239 | -2.49 |
| A_55_P1998932 | ref|NM_177260|ens|ENSMUST00000070636|ens|ENSMUST00000107682|gb|AK154236 | Tmem154 | -2.48 |
| A_55_P1959041 | ref|NM_203396|ens|ENSMUST00000069023|gb|AY528666|tc|TC1588250 | Eapa2 | -2.48 |
| A_30_P01019534 | Unknown | chr13:9818700-9824125_R | -2.48 |
| A_51_P340653 | ref|NM_146136|ens|ENSMUST00000029502|gb|AK045797|gb|AK136837 | Slc16a4 | -2.48 |
| A_30_P01022830 | Unknown | chr4:11926618-11936190_R | -2.48 |
| A_55_P2046037 | ref|XM_001475231|ref|XM_001473902|nap|NAP092996-001 | Gm7083 | -2.47 |
| A_30_P01026013 | Unknown | chr4:59093718-59108018_R | -2.47 |
| A_51_P424290 | ref|NM_181818|ens|ENSMUST00000105212|gb|BC148499|gb|BC153102 | Olfr141 | -2.47 |
| A_30_P01020634 | Unknown | chr4:106506151-106537682_F | -2.47 |
| A_55_P2169410 | ens|ENSMUST00000024896|ref|XM_128729|ref|XM_915248|gb|AK019827 | 4930583I09Rik | -2.47 |
| A_55_P2187899 | gb|AK015708|riken|4930505N22|nap|NAP122568-001 | 4930505N22Rik | -2.47 |
| A_55_P1955264 | ref|XM_001474501|ref|XM_001480118 | Gm7306 | -2.47 |
| A_55_P1988453 | ens|ENSMUST00000106176 | ENSMUST00000106176 | -2.46 |
| A_30_P01025961 | Unknown | chr18:69958121-69958487_F | -2.46 |
| A_55_P2021595 | ens|ENSMUST00000053451|gb|AK016848|gb|AK015077|tc|TC1762701 | ENSMUST00000053451 | -2.46 |
| A_55_P2171325 | ens|ENSMUST00000098069|gb|AK132941|riken|4930526N04 | ENSMUST00000098069 | -2.46 |
| A_52_P157880 | ref|XM_357943|ref|XM_911488|nap|NAP000142-002 | Gm1947 | -2.45 |
| A_52_P417978 | ref|NM_027510|ref|NM_001100609|ref|NM_001102678|ref|NM_009529 | 3830403N18Rik | -2.45 |
| A_51_P342926 | ref|NM_012050|ens|ENSMUST00000065494|gb|AB007848|gb|BC095997 | Omd | -2.45 |
| A_66_P139088 | ens|ENSMUST00000114920|ref|XM_001473620|gb|AK080422|gb|BB639734 | LOC100044967 | -2.45 |
| A_30_P01026801 | Unknown | chr6:116116949-116119051_R | -2.45 |
| A_52_P619248 | ref|NM_181852|ref|NM_001045532|ens|ENSMUST00000091677|ens|ENSMUST00000126540 | Prl2c5 | -2.45 |
| A_52_P337126 | ref|NM_024285|ens|ENSMUST00000095715|gb|AF124510|gb|AF204174 | Bves | -2.45 |
| A_30_P01032369 | Unknown | chr14:65431652-65437902_F | -2.44 |
| A_55_P2034720 | ref|NM_025740|ens|ENSMUST00000093243|ens|ENSMUST00000020754|gb|AK016479 | Ccdc104 | -2.44 |
| A_55_P2205459 | ref|NM_009330|ens|ENSMUST00000021016|ens|ENSMUST00000108113|ens|ENSMUST00000108114 | Hnf1b | -2.44 |
| A_55_P2148809 | ref|NM_011741|ens|ENSMUST00000111040|ens|ENSMUST00000117564|gb|U97068 | Zan | -2.44 |
| A_30_P01021952 | Unknown | chr15:58327703-58329443_R | -2.44 |
| A_55_P2361927 | gb|AK015599|tc|TC1600640|riken|4930480G23|nap|NAP074308-1 | 4930480G23Rik | -2.44 |
| A_30_P01032977 | Unknown | chr6:149221500-149237350_F | -2.44 |
| A_66_P134441 | ref|NM_144868|ens|ENSMUST00000127876|ens|ENSMUST00000137313|ens|ENSMUST00000145270 | Pcnxl3 | -2.44 |
| A_55_P2019507 | ref|NM_207618|ens|ENSMUST00000075461|gb|BC107173|gb|BC107174 | V1rd18 | -2.43 |
| A_55_P2376413 | gb|AK016745|riken|4933408N05|nap|NAP073507-1 | 4933408N05Rik | -2.43 |
| A_30_P01025225 | Unknown | chr4:33364774-33364898_R | -2.43 |
| A_55_P1997046 | ref|NM_001040692|ref|NM_001168646|ref|NM_001136087|ref|NM_001168644 | Slc6a18 | -2.43 |
| A_55_P2005611 | ref|NM_146924|ens|ENSMUST00000077249|gb|AY043306|tc|TC1600902 | Olfr476 | -2.43 |
| A_55_P2015304 | ref|XR_034918|ref|XR_034919|ref|XR_034581|ref|XR_034823 | Gm14049 | -2.42 |
| A_55_P1981461 | ref|XM_001475205|gb|AK017289|riken|5430410E06|nap|NAP000023-012 | 5430410E06Rik | -2.42 |
| A_55_P2134566 | ens|ENSMUST00000056413|gb|AK006222|gb|CR522540|tc|TC1589936 | ENSMUST00000056413 | -2.42 |
| A_52_P90805 | ref|NM_028973|ens|ENSMUST00000064606|gb|AK162723|gb|BC050245 | Lrrc15 | -2.42 |
| A_51_P276802 | ref|NM_054080|ens|ENSMUST00000078239|gb|AK012162|tc|TC1591493 | Akr1c20 | -2.42 |
| A_51_P399905 | ens|ENSMUST00000087544|gb|AK030541|riken|5330429D05|nap|NAP124409-1 | ENSMUST00000087544 | -2.41 |
| A_51_P356842 | ref|NM_207230|ens|ENSMUST00000082307|nap|NAP098943-001 | Olfr320 | -2.41 |
| A_51_P506755 | ref|NM_009351|ens|ENSMUST00000006444|gb|AK154212|gb|AK133745 | Tep1 | -2.41 |
| A_52_P534810 | ref|NM_001013750|ens|ENSMUST00000059937|gb|BC150996|gb|AK076972 | Gm597 | -2.41 |
| A_30_P01032551 | Unknown | chr1:163508244-163586072_R | -2.41 |
| A_52_P830312 | ref|NM_173785|ens|ENSMUST00000054523|gb|AK133203|gb|AK172525 | A230051G13Rik | -2.41 |
| A_55_P2157859 | ref|NM_133203|ens|ENSMUST00000014687|ens|ENSMUST00000087985|ens|ENSMUST00000122219 | Klra17 | -2.41 |
| A_66_P113466 | ens|ENSMUST00000103334|gb|DQ372793|gb|X14098|gb|DQ372769 | ENSMUST00000103334 | -2.41 |
| A_55_P1957114 | ref|XM_001472790|ref|XM_001472827|ref|XM_001478649|ref|XM_001478653 | LOC100039380 | -2.41 |
| A_65_P02958 | ref|NM_001113413|ens|ENSMUST00000041826|gb|AK158046|gb|AK167278 | Rnf13 | -2.41 |
| A_55_P2082482 | ref|XM_001475161 | Gm2935 | -2.41 |
| A_52_P357128 | ref|NM_026094|ens|ENSMUST00000020383|gb|BC114539|gb|BC118977 | Atp8b3 | -2.41 |
| A_52_P60303 | ref|NM_001033455|ens|ENSMUST00000047207|gb|BC150930|gb|BC151167 | Ccdc27 | -2.41 |
| A_30_P01030342 | Unknown | chr7:151085617-151100071_F | -2.40 |
| A_30_P01032376 | Unknown | chr12:33611742-33638648_R | -2.40 |
| A_55_P1985346 | ref|XM_001475143|ref|XM_001477850 | Gm13017 | -2.40 |
| A_55_P2018412 | ref|NM_177191|ens|ENSMUST00000081134|gb|AK029735|tc|TC1587910 | Sycp2 | -2.40 |
| A_55_P2119897 | ref|NM_010153|gb|AY686636|gb|BC106091|tc|TC1581078 | Erbb3 | -2.40 |
| A_55_P2023912 | ref|XR_030502 | LOC630896 | -2.40 |
| A_30_P01024865 | Unknown | chr12:117623500-117643575_F | -2.40 |
| A_55_P2025902 | ref|XR_031747|ref|XR_030507|ref|XR_030520|ref|XR_030658 | Gm2651 | -2.40 |
| A_55_P1974447 | ref|NM_144894|ref|NM_001048148|ens|ENSMUST00000029105|ens|ENSMUST00000156258 | Zgpat | -2.39 |
| A_55_P2144586 | ens|ENSMUST00000110374|gb|AK044519|tc|TC1594901|riken|A930018I09 | ENSMUST00000110374 | -2.39 |
| A_55_P2182056 | ref|NM_153096|ref|NM_153102|ens|ENSMUST00000080541|ens|ENSMUST00000094971 | Zfp353 | -2.39 |
| A_55_P2100505 | ens|ENSMUST00000089855|gb|AK079781|tc|TC1594427|riken|A430065P05 | ENSMUST00000089855 | -2.39 |
| A_51_P157802 | ref|NM_025731|ens|ENSMUST00000025929|gb|AK014971|gb|AK006527 | Hrasls5 | -2.39 |
| A_55_P1953588 | ref|NM_007959|ens|ENSMUST00000006474|ens|ENSMUST00000108147|ref|XM_001479243 | Etv2 | -2.39 |
| A_51_P355434 | ens|ENSMUST00000118228|gb|AK041212|tc|TC1587498|riken|A530090O15 | ENSMUST00000118228 | -2.39 |
| A_55_P2163238 | ref|NM_207247|ens|ENSMUST00000047660|gb|BC128291|gb|AY518698 | Pglyrp3 | -2.39 |
| A_51_P275751 | ref|NM_033477|ens|ENSMUST00000061859|gb|BC138707|gb|BC138708 | D17H6S53E | -2.39 |
| A_55_P2042456 | ref|NM_153786|ens|ENSMUST00000058347|gb|AK048390|gb|AJ578054 | Vgll2 | -2.39 |
| A_55_P2052136 | ref|NM_019784|ens|ENSMUST00000021453|ref|XR_034206|gb|AB000682 | Tex21 | -2.39 |
| A_55_P1993280 | ref|NM_001166738|ref|NM_001166739|ref|NM_001166744|ref|NM_001166747 | Vmn1r104 | -2.39 |
| A_30_P01031482 | Unknown | chr8:122712014-122732520_F | -2.38 |
| A_30_P01027769 | Unknown | chr4:135896689-135918414_R | -2.38 |
| A_51_P230324 | ref|NM_001081046|ens|ENSMUST00000021029|gb|BC050801|gb|AK014861 | Efcab3 | -2.38 |
| A_51_P239994 | ref|NM_011376|ens|ENSMUST00000020071|gb|U40575|nap|NAP013924-001 | Sim1 | -2.38 |
| A_30_P01021594 | Unknown | chr1:134867906-134885481_F | -2.38 |
| A_55_P2012627 | ref|NR_033455|ens|ENSMUST00000097569|gb|AK141979|riken|D130079J24 | Gm10536 | -2.38 |
| A_30_P01032162 | Unknown | chr16:29907604-29946602_R | -2.38 |
| A_52_P24980 | ens|ENSMUST00000105292|gb|AK053219|gb|BC115567|gb|BC115566 | ENSMUST00000105292 | -2.38 |
| A_55_P2052913 | ens|ENSMUST00000103448|ref|XM_903427|ref|XM_001473588|gb|X17631 | ENSMUST00000103448 | -2.38 |
| A_55_P2098593 | ens|ENSMUST00000117191|ref|XM_001476986|gb|AK136374|gb|BC086661 | LOC100046353 | -2.38 |
| A_55_P2056714 | ref|NM_001042612|ens|ENSMUST00000085944|gb|AY596203|gb|AK145425 | Nlrp9c | -2.37 |
| A_55_P2179030 | ref|NM_017375|gb|AK155028|gb|U58888|gb|AK207431 | Ostf1 | -2.37 |
| A_52_P473106 | ref|NM_153114|ens|ENSMUST00000053144|gb|BC132494|gb|BC132498 | Otos | -2.37 |
| A_55_P2109655 | ref|XR_004857|ref|XR_033885|nap|NAP028851-1 | LOC675962 | -2.37 |
| A_55_P2003833 | ref|XR_031446|ref|XR_032413 | LOC100045868 | -2.37 |
| A_52_P14856 | ref|XM_895628|ref|XM_924746|gb|AK139678|tc|TC1640687 | D16Ertd519e | -2.37 |
| A_55_P2076471 | ref|NM_173015|ref|NM_001048143|ens|ENSMUST00000035777|ens|ENSMUST00000109120 | Mon1b | -2.37 |
| A_51_P243454 | ens|ENSMUST00000053743|ref|XM_133663|ref|XM_907780|gb|AK016497 | 4931431F19Rik | -2.37 |
| A_52_P365044 | ref|NR_027879|ens|ENSMUST00000068576|gb|AK015785|gb|AK016343 | 4930583K01Rik | -2.37 |
| A_55_P2006774 | ref|NM_139197|ens|ENSMUST00000028172|gb|AF292399|gb|BC106831 | Gbgt1 | -2.37 |
| A_52_P90463 | ref|NM_146653|ens|ENSMUST00000058118|tc|NP830903|nap|NAP021345-001 | Olfr435 | -2.37 |
| A_55_P2324654 | gb|CN842534 | 4933437N03Rik | -2.37 |
| A_55_P2134591 | ref|NM_178776|ens|ENSMUST00000052702|ref|XM_001471885|gb|BC049715 | BC049715 | -2.37 |
| A_65_P16231 | ref|NM_027238|ens|ENSMUST00000102823|gb|AK134680|riken|6330522E13 | Ttc39b | -2.37 |
| A_55_P2428968 | ref|NM_028838|ens|ENSMUST00000035076|gb|AK010252|gb|AJ428068 | Lrrc2 | -2.36 |
| A_52_P307874 | ens|ENSMUST00000062899|gb|AK053135|tc|TC1593433|riken|E030011O05 | ENSMUST00000062899 | -2.36 |
| A_66_P132695 | ens|ENSMUST00000156282|ens|ENSMUST00000115447|ens|ENSMUST00000128151|ref|XM_622366 | ENSMUST00000156282 | -2.36 |
| A_52_P551844 | ens|ENSMUST00000108880|gb|AK079810|gb|BC096763|tc|TC1703075 | ENSMUST00000108880 | -2.36 |
| A_51_P335694 | ref|NM_029613|ens|ENSMUST00000055121|gb|BC115508|gb|BC115509 | Krtap4-7 | -2.36 |
| A_55_P2063736 | ref|NM_008147|ref|NM_013532|ens|ENSMUST00000105480|ens|ENSMUST00000049800 | Gp49a | -2.36 |
| A_55_P2305005 | gb|AK020767|riken|A430105J06|nap|NAP076109-1 | A430105J06Rik | -2.35 |
| A_55_P2112524 | ref|NM_028231|ens|ENSMUST00000119970|ens|ENSMUST00000119310|ens|ENSMUST00000045917 | Kcnmb2 | -2.35 |
| A_55_P2106175 | ref|NM_008102|ens|ENSMUST00000089959|gb|L09737|gb|BC069921 | Gch1 | -2.35 |
| A_55_P1998943 | ref|NM_145211|ref|NM_011852|ens|ENSMUST00000086373|ens|ENSMUST00000111842 | Oas1a | -2.35 |
| A_51_P409637 | ref|NM_001100394|ens|ENSMUST00000152718|ens|ENSMUST00000041763|gb|BC049719 | 4930505A04Rik | -2.35 |
| A_55_P1979659 | ref|NM_198654|ens|ENSMUST00000076952|ens|ENSMUST00000139340|ens|ENSMUST00000078259 | Nsl1 | -2.35 |
| A_66_P104180 | ref|XM_001474641|gb|AK009355|riken|2310015D24|nap|NAP019118-001 | 2310015D24Rik | -2.35 |
| A_55_P2151591 | ens|ENSMUST00000032909|ref|XM_001477682|gb|AK142889|riken|D930038O19 | ENSMUST00000032909 | -2.34 |
| A_30_P01021493 | Unknown | chr3:93564825-93576725_F | -2.34 |
| A_55_P2319717 | ref|NM_011139|ens|ENSMUST00000034513|gb|L14677|gb|AK153697 | Pou2f3 | -2.34 |
| A_55_P2250514 | gb|AK016828|tc|NP741502|riken|4933416A02|nap|NAP073562-1 | 4933416A02Rik | -2.34 |
| A_55_P2148504 | ref|NM_010668|ens|ENSMUST00000052664|ens|ENSMUST00000023712|gb|BC120485 | Krt2 | -2.34 |
| A_51_P354003 | ref|NM_008901|gb|BC138657|gb|BC145919|nap|NAP102847-1 | Pou3f4 | -2.34 |
| A_51_P419103 | ens|ENSMUST00000009354|gb|AK009213|tc|TC1593558|riken|2310007L24 | ENSMUST00000009354 | -2.34 |
| A_55_P2186210 | ref|XM_889972|ref|XM_903276|ref|XM_905533 | Gm13015 | -2.34 |
| A_30_P01033174 | Unknown | chr6:31078802-31079644_R | -2.33 |
| A_55_P1982499 | ref|NM_177350|ens|ENSMUST00000056740|gb|AK031523|gb|AF548022 | Gldn | -2.33 |
| A_30_P01027449 | Unknown | chr13:52068582-52071952_F | -2.33 |
| A_52_P47645 | ref|NM_016716|ens|ENSMUST00000004478|gb|AF129738|gb|BC027304 | Cul3 | -2.33 |
| A_55_P2122460 | ref|NM_001033255|ens|ENSMUST00000095129|gb|BC147519|gb|BC147522 | Gm136 | -2.33 |
| A_52_P978481 | ref|XM_923007|ref|XM_901213|gb|AK006354|tc|TC1636873 | 1700025K24Rik | -2.33 |
| A_55_P2065958 | ref|XM_001475657|ref|XM_001478974|gb|BY326470 | Gm3119 | -2.33 |
| A_55_P1998682 | ref|XM_001473014 | Gm2273 | -2.33 |
| A_55_P1956582 | ref|XM_001000740|ref|XM_001001649|ref|XM_973308|gb|AK052049 | XM_001000740 | -2.33 |
| A_55_P2033225 | ref|NM_138948|gb|AF419324|gb|BC104356|gb|BC104357 | Cabp7 | -2.33 |
| A_55_P2098385 | ref|XM_897306 | Gm6355 | -2.33 |
| A_51_P420321 | ref|NM_026286|ens|ENSMUST00000025388|gb|AK015346|gb|AK015400 | Ftmt | -2.32 |
| A_51_P331870 | ref|NM_028862|ens|ENSMUST00000019333|gb|AK014408|gb|AK043002 | Rnf145 | -2.32 |
| A_55_P2421835 | gb|AK015812|riken|4930517G19|nap|NAP074454-1 | 4930517G19Rik | -2.32 |
| A_55_P2213418 | gb|AK016843|riken|4933417E11|nap|NAP073515-1 | 4933417E11Rik | -2.32 |
| A_55_P2040255 | ens|ENSMUST00000095282|gb|AK034302|tc|TC1589114|riken|9330175B10 | ENSMUST00000095282 | -2.32 |
| A_52_P576180 | ref|NM_177738|ens|ENSMUST00000054799|gb|AK077180|gb|BC048671 | BC048671 | -2.32 |
| A_55_P2348579 | gb|AK053343|tc|TC1593122|riken|E130010O07|nap|NAP020908-001 | D6Ertd160e | -2.32 |
| A_66_P126848 | ref|NM_001038697|ens|ENSMUST00000097029|gb|BC104362|nap|NAP103413-1 | Gm5635 | -2.32 |
| A_30_P01029792 | Unknown | chr4:140841206-140841639_F | -2.32 |
| A_55_P2041501 | ref|XM_620708|ref|XM_913611|tc|TC1726497 | Gm5915 | -2.32 |
| A_51_P329811 | ref|NM_009042|ens|ENSMUST00000079926|gb|AK075798|gb|AK133506 | Reg1 | -2.32 |
| A_55_P2101577 | ens|ENSMUST00000041220|gb|AK029603|riken|4930412E20|nap|NAP073993-1 | ENSMUST00000041220 | -2.32 |
| A_30_P01026226 | Unknown | chr17:6514179-6517720_R | -2.32 |
| A_52_P533280 | ref|NM_013637|ens|ENSMUST00000023144|gb|AK007068|gb|AK018980 | Prm1 | -2.31 |
| A_52_P538363 | ref|NM_053127|ens|ENSMUST00000056522|gb|AY013781|gb|BC141154 | Pcdhb2 | -2.31 |
| A_55_P2166833 | ens|ENSMUST00000067600|ref|XM_357051|ref|XM_908720 | Ms4a14 | -2.31 |
| A_55_P2005345 | ref|NM_183428|ref|NM_001128606|ref|NM_001128607|ens|ENSMUST00000105972 | Epb4.1 | -2.31 |
| A_30_P01025727 | Unknown | chr2:167602966-167608069_F | -2.31 |
| A_55_P2026530 | ref|NM_177814|ens|ENSMUST00000100776|ens|ENSMUST00000090302|ens|ENSMUST00000100777 | Erc2 | -2.31 |
| A_55_P2071334 | ref|NM_009102|ens|ENSMUST00000078035|ens|ENSMUST00000029648|ens|ENSMUST00000090295 | Rrh | -2.31 |
| A_55_P2017496 | ens|ENSMUST00000076984|ref|XM_001476211|ref|XM_001475840|gb|AK006461 | ENSMUST00000076984 | -2.31 |
| A_55_P2098170 | ref|NM_146706|ref|NM_146707|ens|ENSMUST00000079827|ens|ENSMUST00000080365 | Olfr401 | -2.30 |
| A_55_P2005295 | ref|XM_903553|ref|XM_908262|ref|XM_001478118|nap|NAP112771-1 | Gm16452 | -2.30 |
| A_52_P207057 | ref|NM_175556|ref|NM_001113360|ens|ENSMUST00000135665|ens|ENSMUST00000097751 | Plch2 | -2.30 |
| A_51_P192924 | ref|NM_001145300|ref|NR_026896|gb|BC145149|gb|AK016512 | 4931440L10Rik | -2.30 |
| A_55_P2111141 | ref|NM_021377|ens|ENSMUST00000072685|gb|AF195056|gb|AK046724 | Sorcs1 | -2.30 |
| A_66_P111011 | ref|NM_008091|ens|ENSMUST00000102976|gb|AK157625|gb|AK156634 | Gata3 | -2.30 |
| A_55_P2160737 | ens|ENSMUST00000103631|ens|ENSMUST00000103604|ref|XM_622800|ref|XM_908681 | ENSMUST00000103631 | -2.30 |
| A_55_P1991124 | ref|NM_153080|ref|NM_001039092|ens|ENSMUST00000000550|ens|ENSMUST00000095254 | Tom1l2 | -2.30 |
| A_52_P549973 | ref|NM_025744|ens|ENSMUST00000100041|ens|ENSMUST00000134607|gb|AK006384 | 4933404M02Rik | -2.30 |
| A_52_P221776 | ref|NM_010616|ens|ENSMUST00000030042|gb|AK018598|gb|BC022225 | Kif12 | -2.30 |
| A_55_P2079991 | ref|NM_148951|ens|ENSMUST00000045102|gb|BC156914|gb|AB074494 | Gipc3 | -2.30 |
| A_30_P01020969 | Unknown | chr7:143759700-143760147_R | -2.29 |
| A_55_P2322120 | gb|BU939399 | 1700121I08Rik | -2.29 |
| A_55_P1999775 | ref|NM_144852|gb|AK043772|tc|TC1577911|riken|A830030P22 | Slc7a4 | -2.29 |
| A_55_P2282361 | gb|AK005014|tc|TC1634858|riken|1300015D01|nap|NAP018744-001 | 1300015D01Rik | -2.29 |
| A_55_P2379510 | gb|AK081638|riken|C130058A18|nap|NAP089826-1 | LOC628062 | -2.29 |
| A_55_P2184737 | ref|XM_001472312|gb|AK033958|tc|TC1585107|riken|9330128I06 | 6720489N17Rik | -2.29 |
| A_55_P2115906 | ref|NM_181680|ref|NM_181682|ens|ENSMUST00000076737|gb|AY314983 | Dsg1c | -2.29 |
| A_55_P2314316 | gb|CA491940 | 0610008F07Rik | -2.29 |
| A_55_P2093277 | gb|AF178753 | Olfr1077-ps1 | -2.29 |
| A_30_P01020518 | Unknown | chr9:78104935-78116974_F | -2.29 |
| A_55_P2233462 | gb|AK012274|tc|TC1604045|riken|2700022O18|nap|NAP091368-1 | 2700022O18Rik | -2.28 |
| A_55_P1957430 | ref|XM_001473316 | LOC100044910 | -2.28 |
| A_30_P01017530 | Unknown | chrX:50096566-50098135_R | -2.28 |
| A_66_P116299 | ref|NM_019663|ens|ENSMUST00000098651|gb|AK187989|gb|AK013753 | Pias1 | -2.28 |
| A_55_P2185905 | ref|NM_032002|ens|ENSMUST00000080140|ens|ENSMUST00000034861|ens|ENSMUST00000137675 | Nrg4 | -2.28 |
| A_55_P2033845 | ref|XM_919542|ref|XM_484215|nap|NAP111303-1 | Gm5445 | -2.28 |
| A_55_P2067798 | ref|NM_207543|ens|ENSMUST00000074132|gb|BC114347|gb|BC156709 | Vmn1r59 | -2.28 |
| A_30_P01030590 | Unknown | chr19:38392796-38398771_R | -2.28 |
| A_55_P2256601 | gb|BG068260 | AU022434 | -2.27 |
| A_55_P2011747 | ref|NM_178069|ens|ENSMUST00000023190|ens|ENSMUST00000089772|ens|ENSMUST00000143170 | Lsg1 | -2.27 |
| A_55_P2012537 | ref|XM_001479678|ref|XM_001480848 | Gm10847 | -2.27 |
| A_51_P331288 | ref|NM_009731|ens|ENSMUST00000007449|gb|AK147775|gb|AK002705 | Akr1b7 | -2.27 |
| A_55_P2114557 | ref|XM_001472110|ref|XM_001479325 | Gm3924 | -2.27 |
| A_55_P2128556 | ens|ENSMUST00000106832|gb|AK046571|tc|TC1590646|riken|B430104F01 | ENSMUST00000106832 | -2.27 |
| A_55_P2039712 | ref|NM_146112|ref|NM_001110212|ens|ENSMUST00000027475|ens|ENSMUST00000097662 | Gigyf2 | -2.27 |
| A_30_P01018244 | Unknown | chr8:70623390-70645212_R | -2.27 |
| A_30_P01022431 | Unknown | chr14:21437920-21464655_F | -2.27 |
| A_55_P2026458 | ref|NM_172866|ens|ENSMUST00000117140|ens|ENSMUST00000030190|ens|ENSMUST00000107886 | Rgp1 | -2.27 |
| A_55_P2409108 | gb|AY512943|gb|AY512936|tc|TC1591131|nap|NAP125237-1 | Gm16508 | -2.26 |
| A_55_P1998737 | ref|XM_001476791|ref|XM_001480800|gb|BB198284|tc|TC1658109 | LOC100046778 | -2.26 |
| A_30_P01018155 | Unknown | chr4:136856064-136875614_F | -2.26 |
| A_55_P2183168 | ref|XR_034569 | LOC100048406 | -2.26 |
| A_55_P1958744 | ens|ENSMUST00000108366|gb|AK039902|gb|AK033377|tc|TC1624781 | ENSMUST00000108366 | -2.26 |
| A_52_P102413 | ref|NM_145930|ens|ENSMUST00000046633|gb|AK045350|gb|AK143413 | AW549877 | -2.26 |
| A_66_P104221 | ref|XM_001478083|ref|XM_001479502|tc|TC1609340|nap|NAP071752-1 | Gm4122 | -2.26 |
| A_30_P01032400 | Unknown | chr11:33414450-33450750_F | -2.26 |
| A_55_P2061253 | ref|NM_138944|ens|ENSMUST00000034115|gb|AK084240|gb|AK087546 | Pou4f2 | -2.26 |
| A_55_P1993019 | ref|XM_918222 | LOC640972 | -2.26 |
| A_52_P135873 | ref|XM_001475453|tc|TC1574886|tc|TC1746951|tc|TC1695075 | Gm2744 | -2.26 |
| A_51_P397437 | ref|NM_183103|ens|ENSMUST00000119427|ens|ENSMUST00000055340|gb|AK007173 | Prss46 | -2.25 |
| A_55_P2242124 | gb|BG079244|tc|TC1660859|nap|NAP036689-1 | D15Ertd50e | -2.25 |
| A_30_P01017886 | Unknown | chr7:28588159-28617009_F | -2.25 |
| A_55_P2091120 | ref|NM_001142724|gb|AK016973|tc|TC1636164|riken|4933428M03 | Kbtbd12 | -2.25 |
| A_55_P2070009 | ref|NM_010660|ens|ENSMUST00000006961|ens|ENSMUST00000103131|gb|BC156547 | Krt10 | -2.25 |
| A_55_P1994992 | ref|XM_001479552|tc|TC1672211 | Klf9 | -2.25 |
| A_52_P671543 | ref|NM_008895|ens|ENSMUST00000020990|gb|AK030714|gb|AK133800 | Pomc | -2.25 |
| A_55_P2158324 | ref|XM_136686|ref|XM_910074 | Gm4958 | -2.25 |
| A_30_P01024849 | Unknown | chr2:168136389-168136560_R | -2.25 |
| A_52_P483280 | ref|NM_010685|ens|ENSMUST00000061755|gb|AK163933|gb|AK088804 | Lamp2 | -2.25 |
| A_51_P383755 | ens|ENSMUST00000032900|ref|XM_989766|ref|XM_001478810|gb|AK003497 | 1110006G14Rik | -2.24 |
| A_55_P2118095 | ens|ENSMUST00000101038|gb|AK040636|tc|TC1622629|riken|A430110O19 | ENSMUST00000101038 | -2.24 |
| A_55_P2285317 | gb|AK015880|tc|TC1606154|riken|4930524B17|nap|NAP074444-1 | 4930524B17Rik | -2.24 |
| A_55_P2019109 | nap|NAP111440-1|nap|NAP111442-1 | NAP111440-1 | -2.24 |
| A_55_P2019914 | ref|NM_001165997|ref|NM_029773|ref|NM_001165998|ens|ENSMUST00000114501 | Spopl | -2.24 |
| A_30_P01022302 | Unknown | chr9:56910620-56919667_F | -2.24 |
| A_51_P246092 | ref|NM_027074|ens|ENSMUST00000078770|gb|AK006335|gb|BC061241 | 1700025F22Rik | -2.24 |
| A_51_P502437 | ref|NM_009785|ens|ENSMUST00000022567|gb|AJ010949|tc|TC1585796 | Cacna2d3 | -2.24 |
| A_55_P2165028 | ens|ENSMUST00000091375|ref|XM_357274|ref|XM_906413|nap|NAP095932-001 | Rps24-ps2 | -2.24 |
| A_30_P01022758 | Unknown | chrX:146832315-146875680_R | -2.24 |
| A_30_P01030840 | Unknown | chr12:111287772-111333890_R | -2.24 |
| A_55_P2007636 | ens|ENSMUST00000121671|tc|TC1699244|tc|TC1644340 | ENSMUST00000121671 | -2.24 |
| A_55_P2056293 | ref|NM_153053|ens|ENSMUST00000076372|gb|AK047751|tc|TC1574620 | Sf3b4 | -2.24 |
| A_52_P151227 | ref|XM_001476605|gb|AK085785|tc|TC1622090|riken|D830009C02 | LOC100046158 | -2.23 |
| A_55_P2006385 | ref|NM_001037502|ens|ENSMUST00000099204|ens|ENSMUST00000121912|gb|BC141496 | Defb28 | -2.23 |
| A_55_P2056926 | ref|NM_198625|ens|ENSMUST00000052457|ref|XM_001477797|gb|AK052172 | Mtss1l | -2.23 |
| A_55_P2044717 | ens|ENSMUST00000055626 | ENSMUST00000055626 | -2.23 |
| A_66_P119550 | ens|ENSMUST00000045546|ref|XM_001476076|ref|XM_978766|tc|NP818202 | Olfr607 | -2.23 |
| A_55_P2038489 | ref|XM_001004831|ref|XM_001004836|ref|XM_984350 | Gm9205 | -2.23 |
| A_55_P2146655 | ref|XM_001472967|ref|XM_001475384 | Gm2262 | -2.23 |
| A_51_P454707 | ens|ENSMUST00000107750|gb|AK041359|tc|TC1587278|riken|A630005A06 | ENSMUST00000107750 | -2.23 |
| A_55_P1972882 | ref|XM_144122|ref|XM_112066|tc|TC1705606|tc|TC1734010 | E230028L10Rik | -2.23 |
| A_55_P1981465 | ref|XM_001478122 | LOC100047430 | -2.22 |
| A_55_P2013765 | ref|NM_023130|ref|NM_001139513|ref|NM_001139511|ref|NM_001139512 | Raly | -2.22 |
| A_55_P1971383 | ens|ENSMUST00000014072|ens|ENSMUST00000108187|ref|XM_001000510|ref|XM_906694 | ENSMUST00000014072 | -2.22 |
| A_55_P2042396 | ref|NM_028070|ens|ENSMUST00000041100|ens|ENSMUST00000100568|gb|AK008083 | Alkbh4 | -2.22 |
| A_55_P2368435 | gb|AK019609|riken|4930442G15|nap|NAP022380-001 | 4930442G15Rik | -2.22 |
| A_66_P118752 | ref|NM_145510|ens|ENSMUST00000047978|gb|AK155054|gb|AK087547 | Rabif | -2.22 |
| A_55_P2016490 | ref|NM_178363|gb|BC055465|nap|NAP005705-001 | Ylpm1 | -2.22 |
| A_55_P2410875 | gb|AK034163|gb|BB080880|riken|9330160M04|nap|NAP006579-002 | C030037D09Rik | -2.22 |
| A_55_P2247275 | gb|AK084148|tc|TC1613969|riken|D230003L03 | D330013E07Rik | -2.22 |
| A_55_P2240919 | gb|AK029486|tc|TC1626483|riken|4833447H21|nap|NAP088595-1 | A730032A03Rik | -2.22 |
| A_55_P2140913 | ref|NM_011720|ref|XM_001000215|gb|AK169459|tc|TC1736438 | Wnt8b | -2.22 |
| A_55_P2102838 | ref|XM_001475592|ref|XM_001476555 | Gm3073 | -2.22 |
| A_51_P368394 | ref|NM_019964|ens|ENSMUST00000061866|gb|AB028856|gb|AK006026 | Dnajb8 | -2.21 |
| A_55_P2048224 | ref|XM_987811 | LOC676159 | -2.21 |
| A_55_P2112230 | ref|NM_024229|ens|ENSMUST00000026129|gb|AK144544|gb|AK166319 | Pcyt2 | -2.21 |
| A_30_P01027969 | Unknown | chr16:4871449-4874336_R | -2.21 |
| A_55_P2148679 | ref|XM_001476582 | Gm14762 | -2.21 |
| A_55_P2414707 | gb|AK132490|riken|4732463B04 | 4732463B04Rik | -2.21 |
| A_30_P01022555 | Unknown | chr13:74712805-74729233_R | -2.21 |
| A_30_P01023316 | Unknown | chr4:99548675-99598875_R | -2.21 |
| A_30_P01027360 | Unknown | chr15:79286294-79286651_R | -2.21 |
| A_55_P2039038 | ref|NM_001081750|ens|ENSMUST00000063487|ens|ENSMUST00000111417|gb|AK165974 | Zfp664 | -2.21 |
| A_66_P134294 | ref|NM_011667|ens|ENSMUST00000096694|ens|ENSMUST00000115894|tc|TC1588561 | Ube1y1 | -2.21 |
| A_55_P2099952 | ref|NM_009799|ref|NM_001083957|ens|ENSMUST00000094365|ref|XM_994159 | Car1 | -2.21 |
| A_55_P2027693 | ens|ENSMUST00000066298 | ENSMUST00000066298 | -2.21 |
| A_51_P366680 | ref|NR_003243|ens|ENSMUST00000066116|gb|AK020359|tc|TC1594237 | Fate1 | -2.21 |
| A_55_P2135769 | ref|XM_984060|gb|AK076616|riken|4921536C22|nap|NAP119273-1 | Gm8122 | -2.21 |
| A_30_P01017795 | Unknown | chrX:98669962-98670424_F | -2.21 |
| A_55_P2142605 | ref|XM_001474988|ref|XM_001476951 | LOC100045825 | -2.21 |
| A_55_P2158821 | ens|ENSMUST00000097619|tc|TC1639537 | Gm1614 | -2.21 |
| A_51_P229002 | ref|NM_029777|ref|NM_001122685|ens|ENSMUST00000027322|gb|BC005518 | Rhbdd1 | -2.20 |
| A_55_P2263342 | ref|NR_015613|ref|NR_027909|gb|AK006510|riken|1700029M20 | 1700029M20Rik | -2.20 |
| A_55_P1999842 | ref|XM_001477263|ref|XM_001477856 | Gm3386 | -2.20 |
| A_55_P2178006 | ens|ENSMUST00000121426 | ENSMUST00000121426 | -2.20 |
| A_51_P281593 | ref|NM_028000|ens|ENSMUST00000139836|ens|ENSMUST00000068916|ens|ENSMUST00000124764 | Ppapdc1b | -2.20 |
| A_30_P01032914 | Unknown | chr1:55298349-55308374_R | -2.20 |
| A_30_P01026287 | Unknown | chr8:49911596-49912196_R | -2.20 |
| A_55_P1979477 | ens|ENSMUST00000067917 | ENSMUST00000067917 | -2.20 |
| A_55_P2086428 | ref|XM_001473465|ref|XM_906010|gb|BB429596|tc|TC1704851 | Gm4988 | -2.20 |
| A_52_P131372 | ref|XM_913504 | LOC633908 | -2.19 |
| A_51_P509229 | ref|NM_144925|ens|ENSMUST00000094053|gb|AK014218|tc|TC1696061 | Tnrc6a | -2.19 |
| A_30_P01029275 | Unknown | chr3:40966969-40976585_R | -2.19 |
| A_66_P123735 | ref|XR_034495|ref|XR_034802|gb|AK143547|gb|AK008913 | Gm7938 | -2.19 |
| A_55_P2149946 | ref|XM_001480139|ref|XM_001481173 | 2410021H03Rik | -2.19 |
| A_30_P01025563 | Unknown | chr12:110738120-110769295_F | -2.19 |
| A_55_P2041514 | ref|NM_023774|ens|ENSMUST00000118305|ens|ENSMUST00000062542|gb|AK016090 | 4930550L24Rik | -2.19 |
| A_30_P01023455 | Unknown | chr7:88668948-88678046_F | -2.19 |
| A_52_P93422 | ref|NM_172549|ens|ENSMUST00000092412|ens|ENSMUST00000001712|gb|BC057551 | Cabin1 | -2.19 |
| A_30_P01021047 | Unknown | chr8:124364048-124365705_F | -2.19 |
| A_52_P664506 | ref|NM_018811|gb|AK153642|gb|AK139526|gb|AF546701 | Abhd2 | -2.19 |
| A_30_P01025331 | Unknown | chr6:125330524-125410249_F | -2.19 |
| A_52_P208222 | ref|NM_001025568|ref|NM_001159952|ref|NM_001159960|ref|NM_001159957 | Pde1c | -2.19 |
| A_55_P2056106 | ref|XM_001476247 | Gm3293 | -2.19 |
| A_66_P140223 | ref|NM_198630|ens|ENSMUST00000056146|ref|XR_035468|ref|XR_034721 | 1810024B03Rik | -2.18 |
| A_55_P2178039 | ref|XM_976257 | LOC674665 | -2.18 |
| A_51_P399175 | ref|NM_027982|ens|ENSMUST00000002298|gb|BC117498|gb|BC121788 | Ppm1j | -2.18 |
| A_52_P251425 | ref|NM_029491|ens|ENSMUST00000097658|gb|AK083747|tc|TC1583380 | D030074E01Rik | -2.18 |
| A_30_P01020292 | Unknown | chr17:29380426-29380827_R | -2.18 |
| A_55_P1972997 | ref|NM_177039|ens|ENSMUST00000057465|ref|XM_001479089|gb|AK163065 | A530016L24Rik | -2.18 |
| A_52_P605812 | ref|NM_178595|ens|ENSMUST00000066352|gb|AK075833|gb|BC098218 | Ptrh1 | -2.18 |
| A_51_P167292 | ref|NM_009892|ens|ENSMUST00000063062|gb|AK137503|gb|AK154420 | Chi3l3 | -2.18 |
| A_30_P01032796 | Unknown | chr8:74431707-74442440_R | -2.18 |
| A_51_P309530 | ref|NM_025684|ens|ENSMUST00000067085|gb|AK017656|gb|BC132046 | Nepn | -2.18 |
| A_55_P2341025 | gb|BF139390 | Gm1420 | -2.18 |
| A_55_P2175767 | ref|NM_021329|ref|XM_001480603|ref|XR_034454|ref|XR_034984 | Rangrf | -2.18 |
| A_30_P01028050 | Unknown | chr6:148163559-148164253_F | -2.18 |
| A_55_P2070262 | ens|ENSMUST00000098384|gb|AK155210|tc|TC1735341|riken|F630208N05 | Gm10621 | -2.18 |
| A_51_P272066 | ref|NM_025929|ens|ENSMUST00000077004|gb|AK008365|gb|AK018552 | 2010109I03Rik | -2.18 |
| A_55_P1962329 | ref|NM_153110|ref|NM_001177551|ens|ENSMUST00000078409|ens|ENSMUST00000095293 | Trim61 | -2.17 |
| A_30_P01027477 | Unknown | chrX:92151384-92157256_R | -2.17 |
| A_55_P2153545 | ref|XR_031824|ref|XR_033009 | Gm7108 | -2.17 |
| A_55_P2194049 | gb|AK016816|tc|TC1600464|riken|4933415D12|nap|NAP073476-1 | 4933415D12Rik | -2.17 |
| A_55_P1979610 | ref|XM_001473292 | Gm2388 | -2.17 |
| A_51_P497661 | ref|NM_021320|gb|AF268066|gb|BC119182|gb|BC119184 | Ntn4 | -2.17 |
| A_55_P2091294 | ref|NM_001005247|ref|NM_001005248|ref|NM_001167864|ens|ENSMUST00000107649 | Hps5 | -2.17 |
| A_55_P2085761 | ref|NM_028500|ref|NM_029782|ens|ENSMUST00000109973|ens|ENSMUST00000109974 | Calr3 | -2.17 |
| A_52_P356119 | ref|NM_008316|ens|ENSMUST00000020683|ens|ENSMUST00000129115|gb|AK088403 | Hus1 | -2.17 |
| A_55_P2039349 | ref|NM_001080811|ens|ENSMUST00000109753|ref|XM_001473694|gb|BC082608 | Gm5840 | -2.17 |
| A_55_P2069659 | ref|NM_030728|ens|ENSMUST00000107286|ens|ENSMUST00000064174|gb|AB103331 | 9930013L23Rik | -2.16 |
| A_55_P2030194 | ref|NM_010505|ens|ENSMUST00000102804|ens|ENSMUST00000107135|ens|ENSMUST00000107136 | Ifna5 | -2.16 |
| A_51_P249559 | ref|NM_019457|ens|ENSMUST00000023006|gb|AF092208|gb|AK077124 | Lrrc6 | -2.16 |
| A_55_P2104422 | ref|NM_001005787|ens|ENSMUST00000030290|gb|BC037607|tc|TC1591890 | Inadl | -2.16 |
| A_30_P01022198 | Unknown | chr15:102026122-102033725_R | -2.16 |
| A_30_P01024416 | Unknown | chr4:145539725-145596000_R | -2.16 |
| A_55_P2326802 | gb|BG070114 | AU014876 | -2.16 |
| A_30_P01020029 | Unknown | chr13:99890903-99892577_R | -2.16 |
| A_66_P139116 | ref|NM_012053|ens|ENSMUST00000004072|gb|AK152063|gb|AK088263 | Rpl8 | -2.16 |
| A_55_P2037787 | ref|XM_001478340|ref|XM_001478221|ref|XM_001473642|ref|XM_001474087 | Gm3798 | -2.16 |
| A_51_P341336 | ref|NM_025778|ens|ENSMUST00000032321|gb|AK016670|tc|TC1585007 | Bcl2l14 | -2.16 |
| A_55_P2097393 | ref|NM_026132|ens|ENSMUST00000102897|gb|AY495589|gb|AK006683 | Txndc8 | -2.15 |
| A_52_P479961 | ref|XR_034032|ref|XR_035052|nap|NAP114245-1 | Gm14336 | -2.15 |
| A_51_P467430 | ens|ENSMUST00000106514|gb|Z83816|tc|NP046977 | ENSMUST00000106514 | -2.15 |
| A_55_P1980308 | ref|NM_019992|ens|ENSMUST00000031171|ens|ENSMUST00000113389|gb|BC145550 | Stap1 | -2.15 |
| A_55_P2180824 | ref|XR_032712|ref|XR_033399 | Gm7483 | -2.15 |
| A_51_P149257 | ref|NM_153104|ens|ENSMUST00000054173|gb|BC141532|gb|AJ457190 | Phospho1 | -2.15 |
| A_55_P2161804 | ref|XM_001480254|ref|XM_001480445 | Gm4530 | -2.15 |
| A_55_P1986123 | ref|NM_178653|ens|ENSMUST00000040538|ens|ENSMUST00000111126|ens|ENSMUST00000097455 | Sccpdh | -2.15 |
| A_55_P2182557 | ref|NM_183111|gb|AK016210|gb|BY715820|tc|TC1593953 | 4930563M21Rik | -2.15 |
| A_55_P1995473 | ref|NM_013641|ens|ENSMUST00000019608|gb|AK218198|gb|AK164746 | Ptger1 | -2.15 |
| A_51_P116609 | ref|NM_025687|ens|ENSMUST00000034568|gb|BC048458|gb|AF285582 | Tex12 | -2.15 |
| A_30_P01023664 | Unknown | chr3:117935228-118089771_R | -2.15 |
| A_55_P2060323 | ens|ENSMUST00000118559|tc|TC1678819 | ENSMUST00000118559 | -2.14 |
| A_55_P1961433 | ref|XM_001479314 | Gm4171 | -2.14 |
| A_30_P01026549 | Unknown | chrX:121032646-121038407_R | -2.14 |
| A_55_P2075048 | ref|NM_026195|ens|ENSMUST00000027384|ref|XM_001476884|gb|AK012074 | Atic | -2.14 |
| A_52_P2160 | ens|ENSMUST00000021742|gb|AK040674|riken|A530013C23|nap|NAP081120-1 | ENSMUST00000021742 | -2.14 |
| A_55_P2031611 | ref|XM_001002792|ref|XM_001476877|gb|AK008669|riken|2210008F06 | 2210008F06Rik | -2.14 |
| A_55_P1996181 | ref|NM_001103368 | Vmn2r80 | -2.14 |
| A_55_P2145908 | ref|NM_008831|ens|ENSMUST00000087858|ens|ENSMUST00000125172|gb|AK152933 | Phb | -2.14 |
| A_55_P2313658 | gb|BG066537 | C79777 | -2.14 |
| A_30_P01032499 | Unknown | chr8:85952719-85958369_F | -2.14 |
| A_30_P01030043 | Unknown | chr9:65647428-65677009_F | -2.14 |
| A_30_P01033244 | Unknown | chr10:110980223-110980667_R | -2.14 |
| A_66_P106148 | ref|NM_009018|gb|BC132028|gb|BC132032|gb|D64162 | Raet1c | -2.14 |
| A_55_P2141988 | ref|NM_001164312|ens|ENSMUST00000046662|gb|AK146137|riken|I730010H05 | Gm4847 | -2.13 |
| A_30_P01024317 | Unknown | chr9:71681897-71682498_F | -2.13 |
| A_55_P1989474 | ref|NM_145129|ens|ENSMUST00000114316|ens|ENSMUST00000034851|gb|AK080415 | Chrna3 | -2.13 |
| A_55_P1997195 | ref|XM_001475257 | LOC100045963 | -2.13 |
| A_51_P516741 | ref|NM_030709|ens|ENSMUST00000070390|gb|AB016230|gb|AB016229 | Tmprss5 | -2.13 |
| A_30_P01027141 | Unknown | chr13:66104025-66114075_R | -2.13 |
| A_52_P260555 | ref|NM_009509|ens|ENSMUST00000027366|gb|M98454|gb|AK027908 | Vil1 | -2.13 |
| A_55_P1978845 | ref|NM_203489|ref|NM_206868|ref|NM_001166712|ref|NM_001166713 | Vmn1r183 | -2.13 |
| A_30_P01024390 | Unknown | chr17:80133213-80144417_F | -2.13 |
| A_30_P01025081 | Unknown | chr9:96664617-96683617_F | -2.13 |
| A_55_P2085165 | ref|XR_030991|ref|XR_031840|ref|XR_032003 | Gm5951 | -2.12 |
| A_55_P2243092 | gb|BQ174124 | C030039E19Rik | -2.12 |
| A_30_P01022075 | Unknown | chr17:17481706-17482457_R | -2.12 |
| A_52_P21066 | ens|ENSMUST00000103327|gb|E10043|gb|Y13991|gb|AF030230 | ENSMUST00000103327 | -2.12 |
| A_55_P2001486 | ref|XM_001472776|ref|XM_001473802 | Gm2191 | -2.12 |
| A_30_P01029480 | Unknown | chr1:15834778-15843077_R | -2.12 |
| A_55_P2121794 | ref|NM_001005343|gb|AY591908|tc|TC1590798 | Sp9 | -2.12 |
| A_55_P2049226 | ref|NM_053156|ens|ENSMUST00000020965|ens|ENSMUST00000110917|gb|AK005893 | Allc | -2.12 |
| A_55_P2165439 | ref|NM_001174099|ens|ENSMUST00000107416|ens|ENSMUST00000007309|gb|X65506 | Krt36 | -2.12 |
| A_55_P2196417 | gb|AK035992|tc|TC1631023|riken|9630025F12|nap|NAP079017-1 | AK035992 | -2.12 |
| A_30_P01023189 | Unknown | chr15:66682293-66722977_F | -2.12 |
| A_51_P318830 | ref|NM_018803|ens|ENSMUST00000029441|gb|BC125634|gb|BC125632 | Syt10 | -2.12 |
| A_30_P01028603 | Unknown | chrX:6982891-7008528_R | -2.12 |
| A_66_P102110 | ref|XM_001474798 | LOC100045717 | -2.12 |
| A_55_P2162543 | ref|XM_001473966 | Gm2557 | -2.11 |
| A_30_P01026275 | Unknown | chr3:41325168-41332366_R | -2.11 |
| A_52_P610639 | ref|NM_021292|ens|ENSMUST00000114148|ens|ENSMUST00000114154|ens|ENSMUST00000031005 | Evc | -2.11 |
| A_30_P01022760 | Unknown | chr3:86827945-86886695_R | -2.11 |
| A_55_P1966155 | ref|NM_001012725|ens|ENSMUST00000094346|gb|BC141216|gb|AY541526 | Wfdc6b | -2.11 |
| A_51_P442118 | ref|NM_001039228|ens|ENSMUST00000051117|gb|AK030210|tc|TC1589941 | Gm6583 | -2.11 |
| A_55_P2049913 | ref|XM_001472048|ref|XM_001473969 | Gm12711 | -2.11 |
| A_51_P184484 | ref|NM_008607|ens|ENSMUST00000015394|gb|AK150728|gb|AK151626 | Mmp13 | -2.11 |
| A_55_P1988310 | ref|NM_153504|ens|ENSMUST00000107454|ens|ENSMUST00000079420|ens|ENSMUST00000107455 | Rnf183 | -2.11 |
| A_52_P515831 | ens|ENSMUST00000064097|gb|AK033101|tc|TC1598868|riken|8030423F21 | ENSMUST00000064097 | -2.11 |
| A_55_P2335713 | gb|AU018461 | D11Ertd712e | -2.11 |
| A_30_P01025700 | Unknown | chr13:108548345-108679004_F | -2.10 |
| A_55_P2015753 | ref|NM_027147|gb|AK214699|gb|AK050496|gb|AK009710 | Enho | -2.10 |
| A_55_P2143591 | ref|XM_001474915|ref|XM_001478319 | LOC100045775 | -2.10 |
| A_55_P2019670 | ref|NM_001163611|tc|TC1681795 | Nps | -2.10 |
| A_51_P225903 | ref|NM_173070|ens|ENSMUST00000062129|gb|AY158996|gb|BC107019 | Sprr4 | -2.10 |
| A_52_P333825 | ref|NM_207022|ens|ENSMUST00000062463|gb|BC104401|gb|BC104400 | Tas2r118 | -2.10 |
| A_30_P01033491 | Unknown | chrX:6790023-6896433_R | -2.10 |
| A_66_P105784 | ref|NM_009020|ens|ENSMUST00000111227|ens|ENSMUST00000044031|gb|AK089075 | Rag2 | -2.10 |
| A_55_P2271058 | gb|AF032130 | Dep1 | -2.10 |
| A_55_P2120025 | ref|XM_976298|ref|XM_986318|gb|DV058394 | Gm7596 | -2.10 |
| A_52_P480088 | ref|NM_025685|ens|ENSMUST00000036300|gb|AK003879|tc|TC1651469 | Col27a1 | -2.10 |
| A_55_P2141147 | ref|XM_001478650|ref|XM_001478742|gb|AK019813|riken|4930577H14 | 4930577H14Rik | -2.10 |
| A_52_P305230 | ref|NM_198610|ens|ENSMUST00000039331|gb|AK137439|gb|BC055811 | Igsf21 | -2.09 |
| A_55_P2105406 | ens|ENSMUST00000110138|gb|AK149250|tc|TC1660869|riken|A930001K06 | ENSMUST00000110138 | -2.09 |
| A_30_P01028356 | Unknown | chr8:86706637-86707004_R | -2.09 |
| A_51_P240269 | ref|NM_146261|ens|ENSMUST00000047852|gb|AK039695|gb|BC031748 | Fam199x | -2.09 |
| A_55_P2030721 | ref|NM_172756|ens|ENSMUST00000120725|ens|ENSMUST00000119976|ens|ENSMUST00000060324 | Ankle1 | -2.09 |
| A_30_P01023825 | Unknown | chr4:105035450-105059250_F | -2.09 |
| A_55_P2358399 | ref|NR_033213|gb|AK007172|tc|TC1664602|riken|1700111N16 | 1700111N16Rik | -2.09 |
| A_30_P01030089 | Unknown | chr15:92174226-92201201_R | -2.09 |
| A_55_P2125826 | ref|XR_031258|ref|XR_034324 | Gm7626 | -2.09 |
| A_55_P2050155 | ref|NM_001037712|ens|ENSMUST00000106903|ens|ENSMUST00000051640|ens|ENSMUST00000001965 | Kcnh6 | -2.09 |
| A_51_P346132 | ref|NM_023894|ens|ENSMUST00000089062|gb|DQ058646|gb|AF201698 | Rhox9 | -2.09 |
| A_55_P2024115 | ens|ENSMUST00000089106|ref|XM_001474256|ref|XM_001480088|gb|D86419 | ENSMUST00000089106 | -2.09 |
| A_52_P165654 | ref|NM_011485|gb|BC060970|gb|AK054470|tc|TC1586512 | Star | -2.08 |
| A_30_P01021058 | Unknown | chr2:77678672-77680993_F | -2.08 |
| A_66_P122660 | ref|NM_153524|ref|XM_884240|ref|XM_915262|gb|BC115741 | Mrgpra4 | -2.08 |
| A_55_P2019684 | ref|NM_138653|ens|ENSMUST00000107449|ens|ENSMUST00000030088|gb|BC139079 | Bspry | -2.08 |
| A_55_P1982514 | ref|XM_001473506|ref|XM_915075|nap|NAP028450-1|nap|NAP028447-1 | Gm14496 | -2.08 |
| A_30_P01018138 | Unknown | chr7:129294998-129295498_F | -2.08 |
| A_55_P1965456 | ref|NM_008262|ens|ENSMUST00000056006|gb|U95945|gb|BC024053 | Onecut1 | -2.08 |
| A_55_P2106454 | ens|ENSMUST00000107976|ens|ENSMUST00000107975|ens|ENSMUST00000069184|ref|XM_001472112 | ENSMUST00000107976 | -2.08 |
| A_66_P138662 | ref|NM_147065|ens|ENSMUST00000062719|tc|NP1221834|nap|NAP014987-001 | Olfr1390 | -2.08 |
| A_52_P330111 | ens|ENSMUST00000031618|gb|AK015903|tc|TC1773467|tc|TC1690712 | ENSMUST00000031618 | -2.08 |
| A_55_P2154467 | ref|XM_001480759|gb|AK052948|tc|TC1608847|riken|D930001M23 | LOC100048598 | -2.08 |
| A_55_P2161101 | ref|XM_001473291|gb|BC100510|gb|CN836674 | Gm2511 | -2.07 |
| A_55_P2156978 | ref|XM_001476190|ref|XM_001478704 | LOC100046429 | -2.07 |
| A_55_P2154684 | ref|NM_001174078|ref|NM_011417|ref|NM_001174079|ens|ENSMUST00000098948 | Smarca4 | -2.07 |
| A_55_P1995438 | ref|XR_034654|ref|XR_034694|ref|XR_031147 | Rpl23a-ps2 | -2.07 |
| A_55_P2012301 | ens|ENSMUST00000121661|tc|TC1712820 | 1110003E01Rik | -2.07 |
| A_51_P471659 | ref|NM_145684|ens|ENSMUST00000019051|gb|AK138743|gb|X99252 | Alox12e | -2.07 |
| A_30_P01030867 | Unknown | chr10:69831647-69831983_F | -2.07 |
| A_51_P140429 | ref|NM_026918|ens|ENSMUST00000051122|gb|AK007426|gb|BC031800 | Zg16 | -2.07 |
| A_55_P1974522 | ref|NM_175688|ens|ENSMUST00000062514|gb|AK041317|tc|TC1592691 | A530099J19Rik | -2.07 |
| A_55_P2105195 | ref|NM_031389|ens|ENSMUST00000121583|ens|ENSMUST00000037728|gb|AK166869 | Nlrp4c | -2.07 |
| A_51_P299964 | ref|NM_172901|gb|AK077105|gb|BC060958|tc|TC1586600 | 4933405O20Rik | -2.07 |
| A_55_P2198648 | gb|AK020016|tc|TC1624839|riken|5830420C07 | 5830420C07Rik | -2.06 |
| A_30_P01019395 | Unknown | chr7:137669441-137675722_F | -2.06 |
| A_51_P413302 | ref|NM_009417|ens|ENSMUST00000021005|gb|AK047843|gb|X60703 | Tpo | -2.06 |
| A_30_P01022388 | Unknown | chr17:21801262-21801932_F | -2.06 |
| A_55_P2044453 | ref|XM_001472317|ref|XM_001478173 | Gm2054 | -2.06 |
| A_55_P2161410 | ref|NM_009253|ens|ENSMUST00000055089|gb|BC053337|gb|BC011158 | Serpina3m | -2.06 |
| A_55_P1966690 | ref|NM_021282|ens|ENSMUST00000026552|gb|X62595|gb|L11650 | Cyp2e1 | -2.06 |
| A_30_P01025973 | Unknown | chr1:77819988-77820546_F | -2.06 |
| A_55_P2100949 | ens|ENSMUST00000060930|ref|XR_031282 | Ccdc158 | -2.06 |
| A_55_P2400101 | gb|AK016987|tc|TC1613174|riken|4933430A20|nap|NAP073627-1 | 4933430A20Rik | -2.06 |
| A_55_P2189743 | gb|BX527063|tc|TC1698248 | AA517023 | -2.06 |
| A_52_P297176 | ens|ENSMUST00000095448|gb|AK162363|gb|AK053925|gb|BC096647 | ENSMUST00000095448 | -2.06 |
| A_55_P1965821 | ref|NM_001081189|gb|BC147845|gb|BC147853|nap|NAP027219-1 | Uprt | -2.06 |
| A_55_P2003414 | ref|NM_023125|ref|NM_001102409|ens|ENSMUST00000039492|gb|D84415 | Kng1 | -2.06 |
| A_30_P01020808 | Unknown | chr18:3003075-3016150_F | -2.06 |
| A_55_P1966878 | ref|NM_027187|ens|ENSMUST00000109736|gb|AK146518|tc|TC1664398 | Rnaseh2a | -2.06 |
| A_55_P1976814 | ref|XM_001474628|ref|XM_001476102|tc|TC1679329 | Gm2753 | -2.06 |
| A_30_P01026976 | Unknown | chr17:84397891-84412748_F | -2.06 |
| A_51_P487690 | ref|NM_133871|ens|ENSMUST00000029671|gb|BC111882|gb|BC112402 | Ifi44 | -2.06 |
| A_51_P351166 | ref|NM_010866|ens|ENSMUST00000072514|gb|BC103613|gb|BC103618 | Myod1 | -2.05 |
| A_51_P255387 | ref|NM_026068|ens|ENSMUST00000021157|gb|AK013990|gb|AK005655 | Med31 | -2.05 |
| A_55_P1981479 | ref|NM_008326|ens|ENSMUST00000097271|ens|ENSMUST00000049519|gb|BC145957 | Irgm1 | -2.05 |
| A_55_P2030790 | ref|XM_001479036|nap|NAP059513-1 | LOC100047919 | -2.05 |
| A_30_P01023032 | Unknown | chr16:29711373-29712035_R | -2.05 |
| A_30_P01020423 | Unknown | chr9:89167172-89168980_F | -2.05 |
| A_30_P01027201 | Unknown | chrX:98671653-98672118_F | -2.05 |
| A_30_P01033389 | Unknown | chr4:109071198-109078714_F | -2.05 |
| A_52_P356698 | ref|NM_013665|ens|ENSMUST00000029422|gb|BC172634|gb|U67055 | Shox2 | -2.05 |
| A_30_P01024999 | Unknown | chr6:82608223-82669023_F | -2.05 |
| A_55_P2118856 | ens|ENSMUST00000072455|gb|AK015192|tc|TC1733120|riken|4930423O20 | ENSMUST00000072455 | -2.05 |
| A_55_P2055523 | ref|XM_001475214|ref|XM_001476097|ref|XR_033891|ref|XR_034000 | Gm3160 | -2.05 |
| A_55_P2315762 | gb|AK045275|tc|TC1647431|riken|B130054P17|nap|NAP083065-1 | AK045275 | -2.05 |
| A_30_P01023318 | Unknown | chr6:134846715-134847933_R | -2.05 |
| A_52_P299771 | ref|NM_007535|ens|ENSMUST00000066460|gb|BC120720|gb|BC132545 | Bcl2a1c | -2.05 |
| A_55_P2090265 | ens|ENSMUST00000093427|gb|BC144786|gb|BC145703|gb|BC144787 | Nwd1 | -2.05 |
| A_51_P515649 | ref|NM_053147|ens|ENSMUST00000097609|gb|AK143176|gb|AF326315 | Pcdhb22 | -2.05 |
| A_52_P501875 | ref|NM_001077684|ens|ENSMUST00000094942|gb|AK016309|gb|AK015894 | 4930578N16Rik | -2.05 |
| A_51_P295131 | ref|NM_011039|gb|AF254422|nap|NAP014244-001 | Pax7 | -2.05 |
| A_55_P2125144 | ens|ENSMUST00000121778|ens|ENSMUST00000154257|tc|TC1718914 | ENSMUST00000121778 | -2.04 |
| A_52_P52849 | ref|NM_018867|ens|ENSMUST00000033149|gb|AK019509|tc|TC1582529 | Cpxm2 | -2.04 |
| A_55_P1958270 | ens|ENSMUST00000109516 | Bcl11a | -2.04 |
| A_55_P1970224 | ref|XR_031694|ref|XR_031780 | Gm13176 | -2.04 |
| A_55_P1957932 | ens|ENSMUST00000020855|ens|ENSMUST00000108696|ref|XM_917005|ref|XM_894470 | 1700086D15Rik | -2.04 |
| A_55_P2050415 | ens|ENSMUST00000107297|ens|ENSMUST00000076755|ens|ENSMUST00000107298|ens|ENSMUST00000050758 | ENSMUST00000107297 | -2.04 |
| A_55_P2001966 | ens|ENSMUST00000086204|gb|AK044247|tc|TC1702898|riken|A930003C01 | ENSMUST00000086204 | -2.04 |
| A_55_P1975470 | ref|XM_001005301|ref|XM_984113 | 6720418B01Rik | -2.04 |
| A_55_P2137701 | ref|NM_001177767|ref|NM_009051|ref|XR_032301|ref|XR_032704 | Gm13138 | -2.04 |
| A_55_P2319730 | gb|AK019576|tc|TC1621540|riken|4930414F18|nap|NAP021780-001 | 4930414F18Rik | -2.04 |
| A_55_P2075631 | ref|NM_011394|ens|ENSMUST00000067786|ref|XM_001475083|ref|XM_001475299 | Slc20a2 | -2.04 |
| A_66_P132971 | ref|XM_001474031|ref|XM_001476405|gb|AK015234|tc|TC1618098 | 4930429E23Rik | -2.04 |
| A_30_P01027549 | Unknown | chr15:74320784-74321384_F | -2.04 |
| A_55_P1973326 | ref|XM_001476308 | Gm3165 | -2.04 |
| A_55_P1952086 | ens|ENSMUST00000107861|gb|AK162576|gb|AK038426|tc|TC1615387 | ENSMUST00000107861 | -2.03 |
| A_55_P1977633 | ref|NM_145836|ref|XM_001473734|gb|AK173282|gb|AK160059 | 6430527G18Rik | -2.03 |
| A_55_P1953788 | ref|NM_010583|ens|ENSMUST00000020664|ens|ENSMUST00000109237|gb|AK153613 | Itk | -2.03 |
| A_55_P2026894 | ref|XM_001477848|tc|TC1725171 | LOC100047306 | -2.03 |
| A_55_P1961444 | ref|NM_177568|ens|ENSMUST00000077829|ens|ENSMUST00000006415|ens|ENSMUST00000102524 | Plcb2 | -2.03 |
| A_55_P2124070 | ref|NM_001162864|ens|ENSMUST00000047034|gb|AK173279|gb|BC059249 | Ttbk1 | -2.03 |
| A_30_P01023221 | Unknown | chr7:114386535-114443910_F | -2.03 |
| A_55_P1952091 | ens|ENSMUST00000092780|gb|AK038512|riken|A230021I02|nap|NAP025462-001 | ENSMUST00000092780 | -2.03 |
| A_55_P1977003 | ref|XM_001475321|ref|XM_001479070|tc|TC1734175 | XM_001475321 | -2.03 |
| A_55_P1976943 | ref|NM_001029872|ref|NM_001177632|ens|ENSMUST00000033051|ens|ENSMUST00000098012 | Itgad | -2.03 |
| A_55_P2088223 | ref|NM_001163032|ref|NM_028052|ens|ENSMUST00000112656|ens|ENSMUST00000070323 | Synpr | -2.02 |
| A_30_P01027492 | Unknown | chr1:6181200-6204300_F | -2.02 |
| A_30_P01021962 | Unknown | chrX:121520904-121524132_F | -2.02 |
| A_30_P01032116 | Unknown | chr16:21794415-21815365_F | -2.02 |
| A_30_P01024112 | Unknown | chr11:18753941-18760118_R | -2.02 |
| A_55_P2115981 | ref|NM_031493|ref|NM_001111293|ref|NM_001045539|ens|ENSMUST00000088450 | Xlr5c | -2.02 |
| A_30_P01020459 | Unknown | chr15:99762797-99786472_R | -2.02 |
| A_55_P1975852 | ref|NM_001024626|ens|ENSMUST00000085664|ens|ENSMUST00000105313|gb|AK143655 | Zfp873 | -2.02 |
| A_55_P1980946 | ref|NM_027600|ens|ENSMUST00000028067|ens|ENSMUST00000062060|gb|AK014944 | 4921504E06Rik | -2.02 |
| A_55_P2147265 | ens|ENSMUST00000100438 | ENSMUST00000100438 | -2.02 |
| A_55_P2279852 | gb|BG069336 | AU019202 | -2.01 |
| A_30_P01024716 | Unknown | chr12:73789535-73799787_F | -2.01 |
| A_66_P140508 | ref|NM_001025244|ens|ENSMUST00000058761|gb|AK037252|tc|TC1588946 | Krtap31-2 | -2.01 |
| A_66_P105270 | ref|NM_010403|ens|ENSMUST00000028704|gb|AK149565|gb|BC018587 | Hao1 | -2.01 |
| A_55_P2014457 | ref|XM_914710 | LOC638627 | -2.01 |
| A_30_P01027964 | Unknown | chr1:182180931-182186106_F | -2.01 |
| A_30_P01028811 | Unknown | chr12:56607657-56613582_F | -2.01 |
| A_30_P01030128 | Unknown | chr11:82588507-82594501_F | -2.01 |
| A_55_P2114903 | ens|ENSMUST00000103657|ref|XM_001472767|ref|XM_001475259|gb|X83008 | ENSMUST00000103657 | -2.01 |
| A_30_P01030618 | Unknown | chr12:74135916-74142780_R | -2.01 |
| A_66_P108999 | ens|ENSMUST00000103553|gb|M71214|gb|Z48594|gb|Z48593 | ENSMUST00000103553 | -2.01 |
| A_55_P2013751 | ref|NM_207228|ens|ENSMUST00000114901|ens|ENSMUST00000151735|ens|ENSMUST00000088072 | Tsga10 | -2.01 |
| A_30_P01028558 | Unknown | chrX:35825525-35877700_R | -2.01 |
| A_55_P1975310 | ref|NM_001142750|ref|NM_153109|ens|ENSMUST00000072518|ens|ENSMUST00000073857 | Tgif2lx2 | -2.01 |
| A_55_P2351240 | gb|BU939189 | 4930403D09Rik | -2.01 |
| A_30_P01026767 | Unknown | chr1:71718767-71719614_F | -2.01 |
| A_55_P2413939 | gb|AK018576|gb|BB277633|tc|NP740480|riken|9030625G05 | 9030625G05Rik | -2.01 |
| A_66_P115187 | ref|NM_001007463|ens|ENSMUST00000107870|ens|ENSMUST00000139008|gb|AY769082 | Spag8 | -2.01 |
| A_51_P492595 | ref|NM_172518|ens|ENSMUST00000030757|gb|AK170202|gb|AK028867 | Fbxo42 | -2.01 |
| A_55_P1958697 | ref|NM_028234|ens|ENSMUST00000059644|ens|ENSMUST00000114884|gb|AK078210 | Rbm33 | -2.00 |
| A_30_P01018817 | Unknown | chr1:182808654-182815659_R | -2.00 |
| A_30_P01032566 | Unknown | chr1:122655019-122660142_F | -2.00 |
| A_30_P01024475 | Unknown | chr8:41941084-41947630_R | -2.00 |
| A_51_P262925 | ref|NM_027069|ens|ENSMUST00000031624|gb|BC147518|gb|BC147521 | 1700018F24Rik | -2.00 |
| A_52_P15461 | ref|NM_008357|ens|ENSMUST00000034148|gb|DQ083236|gb|AK155616 | Il15 | -2.00 |
| A_55_P2288690 | gb|CN840722 | 1700066J03Rik | -2.00 |
| A_66_P106611 | ref|NM_008137|ens|ENSMUST00000025602|gb|AK035320|gb|AK085444 | Gna14 | -2.00 |

**Table S7**: Genes up regulated ≥ 2 fold in the cerebral hemisphere of female pups from mothers having FA supplementation during gestation at 4 mg/kg in comparison to mothers at 0.4 mg/kg diet.

| **UniqueID** | **Accession** | **Symbol** | **Fold Change** |
| --- | --- | --- | --- |
| A_30_P01026348 | Unknown | chr15:60656586-60685333_R | 62.50 |
| A_55_P2195172 | gb|AK086332|tc|TC1616454|riken|D930021N14|nap|NAP087253-1 | D930021N14 | 38.46 |
| A_55_P2104071 | ref|NM_001012307|ref|NM_007848|ref|NM_007850|ref|NM_001167790 | Defa23 | 31.25 |
| A_55_P2013725 | ref|NM_001025240|ens|ENSMUST00000056023|gb|BC151154|gb|BC157897 | Gm5346 | 27.78 |
| A_55_P2273454 | gb|BG071831 | D7Ertd128e | 26.32 |
| A_30_P01021727 | Unknown | chr11:3521324-3539552_F | 25.64 |
| A_51_P225493 | ref|NM_027077|ens|ENSMUST00000016106|gb|AK006014|gb|BY706135 | 1700016C15Rik | 24.39 |
| A_55_P2081116 | ref|NM_001081120|ens|ENSMUST00000055257|ref|XM_001006406|ref|XM_001478908 | Fam89a | 22.73 |
| A_55_P1971928 | ref|NM_183253|ref|NM_207658|ens|ENSMUST00000076754|ens|ENSMUST00000098897 | Defa21 | 22.22 |
| A_55_P2332509 | gb|AK039916|tc|TC1601252|riken|A430028G04|nap|NAP025720-001 | A430028G04Rik | 21.28 |
| A_55_P1956147 | ref|NM_008081|ens|ENSMUST00000103158|gb|AK153938|tc|TC1651111 | B4galnt2 | 20.83 |
| A_51_P160713 | ref|NM_009654|ens|ENSMUST00000031314|gb|AJ457860|gb|AK050644 | Alb | 19.61 |
| A_55_P2058621 | ref|XM_917762|gb|AF303833|gb|AF023228|gb|AF023232 | LOC640701 | 19.23 |
| A_55_P2296044 | ref|NM_172795|ref|NM_001168521|ens|ENSMUST00000108287|ens|ENSMUST00000061174 | Sarm1 | 18.18 |
| A_30_P01022469 | Unknown | chr14:99752098-99752425_R | 17.86 |
| A_55_P2039027 | ref|NR_001581|ens|ENSMUST00000081735|ref|XR_034142|ref|XR_032605 | Speer6-ps1 | 17.54 |
| A_30_P01026703 | Unknown | chr1:161064402-161096518_F | 17.24 |
| A_55_P2126815 | ref|XM_001480184 | Gm4443 | 14.49 |
| A_66_P100182 | ref|NM_001011753|gb|BC139227|gb|BC139226|tc|TC1600211 | Olfr115 | 14.29 |
| A_55_P1955816 | ref|NM_172403|ens|ENSMUST00000108820|gb|BC058081|tc|TC1711419 | 2810021J22Rik | 13.33 |
| A_55_P2302888 | gb|BB209673|nap|NAP049488-1 | AU042410 | 13.33 |
| A_55_P2024948 | ref|NM_010461|ens|ENSMUST00000052650 | Hoxb8 | 13.33 |
| A_55_P2094868 | ref|NM_013825|ens|ENSMUST00000100079|ens|ENSMUST00000028362|ens|ENSMUST00000112533 | Ly75 | 12.99 |
| A_51_P198335 | ref|NM_008068|ref|NM_001099641|ens|ENSMUST00000020703|gb|AK137331 | Gabra6 | 12.82 |
| A_52_P1115773 | ref|XM_001479077|gb|AK042451|tc|TC1628017|riken|A630092P20 | LOC100047982 | 12.50 |
| A_55_P2142499 | ens|ENSMUST00000103502|ref|XM_912564|gb|FM179586|gb|EU568191 | LOC636979 | 11.49 |
| A_52_P547456 | ref|NM_026394|ens|ENSMUST00000047153|gb|AK003518|gb|BC060283 | Lce1f | 11.24 |
| A_55_P2195853 | gb|AK017555|tc|NP741073|riken|5730412P04|nap|NAP073025-1 | 5730412P04Rik | 10.99 |
| A_51_P406157 | ref|NM_054084|ens|ENSMUST00000032902|tc|TC1589573|nap|NAP025710-001 | Calcb | 10.64 |
| A_55_P2165834 | ref|NM_146797|ens|ENSMUST00000074221|gb|BC106810|tc|TC1599817 | Olfr1502 | 10.64 |
| A_30_P01028525 | Unknown | chr17:13220550-13236900_R | 10.20 |
| A_30_P01033131 | Unknown | chr9:67414463-67483185_F | 10.00 |
| A_30_P01029985 | Unknown | chr18:75131739-75144870_R | 9.80 |
| A_55_P2012894 | ref|NM_001033288|ens|ENSMUST00000040695|gb|AK137144|tc|TC1586893 | Gm106 | 9.43 |
| A_55_P2047431 | ref|NM_178257|ens|ENSMUST00000102546|gb|AY103454|gb|BC140972 | Il22ra1 | 9.35 |
| A_55_P2053459 | ref|NM_001161355|ref|NM_134249|ref|NM_001161356|ens|ENSMUST00000055102 | Timd2 | 9.35 |
| A_55_P2101696 | ref|NM_008141|ens|ENSMUST00000009252|ens|ENSMUST00000058669|gb|L10666 | Gnat2 | 9.17 |
| A_65_P07361 | gb|AK172895|gb|AK133220|tc|TC1601919|riken|4932418D21 | Trim14 | 9.01 |
| A_55_P2115350 | ref|XR_034837|ref|XR_031415 | Gm9529 | 8.77 |
| A_30_P01028184 | Unknown | chr18:69957420-69957776_F | 8.77 |
| A_55_P1956553 | ens|ENSMUST00000107605|ens|ENSMUST00000056442|ref|XM_001004722|ref|XM_001004719 | ENSMUST00000107605 | 8.47 |
| A_52_P799815 | ref|NM_001025606|ens|ENSMUST00000064347|gb|BC099490|tc|TC1585898 | Tmem171 | 8.40 |
| A_55_P2092204 | ref|NM_019516|ens|ENSMUST00000051105|ens|ENSMUST00000099729|ens|ENSMUST00000079902 | Lgals12 | 8.40 |
| A_30_P01020728 | Unknown | TSIX | 8.40 |
| A_55_P1985732 | ref|NM_013772|ens|ENSMUST00000105219|ens|ENSMUST00000105218|ens|ENSMUST00000090990 | Tcl1b3 | 8.33 |
| A_55_P2039865 | ens|ENSMUST00000100255|ref|XM_001474144|ref|XM_001475477|ref|XM_001475500 | LOC100041207 | 8.13 |
| A_55_P2128582 | ref|XM_001472379 | LOC100044090 | 8.13 |
| A_30_P01027196 | Unknown | chr1:173353316-173367449_F | 8.06 |
| A_30_P01027295 | Unknown | chr16:50752452-50753589_F | 8.06 |
| A_55_P1957401 | ref|NM_001037248|ens|ENSMUST00000098709|ens|ENSMUST00000098719|ens|ENSMUST00000098725 | Gm5156 | 7.94 |
| A_52_P321244 | ens|ENSMUST00000063230|gb|AK076988|tc|TC1593019|riken|4930595D20 | ENSMUST00000063230 | 7.94 |
| A_55_P2127015 | ref|NM_146392|ens|ENSMUST00000035250|gb|BC120861|gb|BC132043 | Olfr720 | 7.87 |
| A_55_P2114953 | ref|NM_011909|ens|ENSMUST00000032198|ref|XM_001480051|gb|AF069502 | Usp18 | 7.87 |
| A_55_P2106598 | ref|NM_001105254|ref|NM_178657|ens|ENSMUST00000110145|ens|ENSMUST00000095509 | Gm10436 | 7.87 |
| A_55_P2006510 | ref|XM_001473378|ref|XM_001475635|gb|AK053437|riken|E130018B18 | XM_001473378 | 7.75 |
| A_55_P1968799 | ref|NM_027157|ens|ENSMUST00000054532|gb|AK009782|tc|TC1587302 | Krtap1-5 | 7.69 |
| A_51_P310196 | ref|NM_011616|ens|ENSMUST00000033466|gb|X65453|tc|TC1594456 | Cd40lg | 7.69 |
| A_30_P01024529 | Unknown | chrX:50407969-50410370_R | 7.69 |
| A_30_P01021311 | Unknown | chr9:78107225-78118850_R | 7.69 |
| A_55_P2171847 | ref|XM_001479270|gb|AK014907|tc|TC1599894|riken|4921517D16 | LOC100047979 | 7.63 |
| A_55_P2281733 | gb|BG063235 | AA408396 | 7.58 |
| A_30_P01032839 | Unknown | chr4:146507925-146532650_F | 7.46 |
| A_55_P2334192 | gb|BQ552637 | AU022934 | 7.41 |
| A_30_P01026657 | Unknown | chr13:60508775-60534625_F | 7.35 |
| A_51_P173547 | ref|NR_015547|ens|ENSMUST00000044816|gb|AK015973|gb|AK006749 | 1700009J07Rik | 7.30 |
| A_55_P2352737 | gb|AK019999|tc|TC1616213|riken|5830406C21|nap|NAP074862-1 | 5830406C21Rik | 7.25 |
| A_55_P2034517 | ens|ENSMUST00000156066|ens|ENSMUST00000131423|ref|XM_001474905|gb|AK029684 | ENSMUST00000156066 | 7.25 |
| A_30_P01030233 | Unknown | chr9:56646768-56649068_R | 7.19 |
| A_55_P2229098 | gb|AK090054|tc|TC1610310|riken|G430080K10|nap|NAP072423-1 | LOC629206 | 7.14 |
| A_30_P01028407 | Unknown | chr9:53613193-53613871_F | 7.09 |
| A_30_P01020046 | Unknown | chr19:56580315-56592490_R | 7.04 |
| A_55_P2113853 | ens|ENSMUST00000075769 | ENSMUST00000075769 | 6.94 |
| A_30_P01033240 | Unknown | chr8:47813487-47824869_F | 6.90 |
| A_55_P2160905 | ref|XM_001003073|ref|XM_974325 | Gm9303 | 6.76 |
| A_55_P2022021 | ref|NM_001011819|ref|NM_146536|ens|ENSMUST00000078217|ens|ENSMUST00000082220 | Olfr312 | 6.71 |
| A_30_P01027289 | Unknown | chr17:13220550-13236900_F | 6.71 |
| A_30_P01019367 | Unknown | chr2:150008075-150101675_R | 6.71 |
| A_55_P2267696 | gb|AK134160|gb|BB031169|riken|5830462I19 | 5830462I19Rik | 6.71 |
| A_30_P01023284 | Unknown | chr19:61177675-61219875_F | 6.58 |
| A_30_P01023613 | Unknown | chr6:121036929-121037581_R | 6.54 |
| A_55_P2056619 | ref|XM_001479541|ref|XM_001480331|gb|BG973545 | LOC100044034 | 6.49 |
| A_55_P2285817 | gb|AK044558|riken|A930022K23 | 4932441J04Rik | 6.49 |
| A_66_P138406 | ref|NM_027218|ens|ENSMUST00000077228|gb|AY230260|gb|AK007794 | Clec4b1 | 6.49 |
| A_55_P2073930 | ref|XM_979023|ref|XM_981165|gb|AK135850|riken|7420426C01 | Gm7759 | 6.45 |
| A_55_P2078459 | ref|NM_010655|ens|ENSMUST00000018506|ens|ENSMUST00000145331|ref|XM_001473171 | Kpna2 | 6.45 |
| A_30_P01023967 | Unknown | chr1:42972297-42972716_R | 6.41 |
| A_66_P108910 | ens|ENSMUST00000114998|gb|AK087627|tc|TC1739667|riken|E230027M02 | ENSMUST00000114998 | 6.41 |
| A_55_P2157765 | ref|NM_001080712|ens|ENSMUST00000097849|gb|AK138880|riken|A430104B10 | Gm7534 | 6.37 |
| A_55_P2166232 | ref|XM_911001|ref|XM_976347 | Kdm4dl | 6.33 |
| A_55_P1970244 | ens|ENSMUST00000070418|gb|AK047716|tc|TC1594392|riken|C030016N17 | Dclk1 | 6.29 |
| A_30_P01019793 | Unknown | chr14:99753319-99753569_R | 6.17 |
| A_55_P1981649 | ref|XM_001475139 | Gm2929 | 6.17 |
| A_55_P2041145 | ref|XM_001481309 | 4930572O03Rik | 6.13 |
| A_30_P01031441 | Unknown | chr8:67542714-67561694_F | 6.13 |
| A_30_P01030551 | Unknown | chr18:47352028-47352681_R | 6.10 |
| A_55_P2129281 | ref|XM_001473377|ref|XM_001477450|gb|BB621217 | Gm15407 | 5.92 |
| A_55_P2118614 | ref|XM_915596|nap|NAP048689-1 | LOC639236 | 5.68 |
| A_30_P01028813 | Unknown | chr1:81854900-81906900_F | 5.68 |
| A_55_P2025630 | ref|XM_918521 | LOC641177 | 5.65 |
| A_51_P295582 | ref|NM_025530|ref|NM_001113562|ens|ENSMUST00000026199|ens|ENSMUST00000112047 | Cutc | 5.62 |
| A_55_P2140271 | ens|ENSMUST00000104981|gb|BG071895 | ENSMUST00000104981 | 5.59 |
| A_30_P01030146 | Unknown | chr4:123155515-123361565_R | 5.56 |
| A_51_P113906 | ref|NM_025467|ens|ENSMUST00000032128|gb|AK007451|gb|AK008986 | Gkn2 | 5.52 |
| A_55_P2087719 | ref|XM_979107 | Gm7762 | 5.52 |
| A_51_P184306 | ref|NM_026351|ens|ENSMUST00000053168|ens|ENSMUST00000134652|gb|AK016184 | Ttc39d | 5.52 |
| A_55_P2032985 | ref|NM_001159730|ref|NM_024458|ens|ENSMUST00000006164|gb|AK020798 | Pdc | 5.49 |
| A_55_P2074688 | ref|NM_013553|ens|ENSMUST00000100164|gb|D11329|tc|TC1647762 | Hoxc4 | 5.49 |
| A_55_P2216601 | gb|AK043303|tc|TC1602869|riken|A730082K24|nap|NAP020718-001 | A730082K24Rik | 5.41 |
| A_55_P2104204 | ref|NM_001167673|ens|ENSMUST00000098903|gb|AK136605|riken|9230005G05 | Pate-e | 5.38 |
| A_55_P2118586 | ref|NM_001164413|ref|NM_001164414|ref|NM_001164412|ens|ENSMUST00000114993 | Cnot4 | 5.32 |
| A_55_P2042451 | ref|NM_133251|gb|AB036708|tc|TC1658676|nap|NAP101870-1 | Vgll1 | 5.32 |
| A_30_P01027943 | Unknown | chr3:9401052-9415202_F | 5.32 |
| A_51_P511375 | ref|NM_011240|ens|ENSMUST00000003310|gb|AK133532|gb|BC030915 | Ranbp2 | 5.29 |
| A_52_P412457 | ref|NM_198958|ens|ENSMUST00000024565|ens|ENSMUST00000115800|gb|AY573240 | Nox3 | 5.29 |
| A_55_P1956718 | ref|NM_028238|ens|ENSMUST00000107256|ens|ENSMUST00000032778|gb|AK165682 | Rab38 | 5.24 |
| A_55_P2014932 | ref|XM_001472658|ref|XM_001472812|gb|AK133228|riken|4932419C06 | Gm2245 | 5.24 |
| A_52_P384690 | ref|NM_031384|ref|NM_001167997|ens|ENSMUST00000113716|ens|ENSMUST00000113718 | Tex11 | 5.18 |
| A_30_P01030135 | Unknown | chr6:34548561-34612216_F | 5.15 |
| A_55_P2038233 | ref|XM_913916|tc|TC1696749|nap|NAP096506-001 | Gm7228 | 5.15 |
| A_52_P625321 | ref|NM_144532|ens|ENSMUST00000025761|gb|AY039218|gb|BC029002 | Cabp4 | 5.13 |
| A_55_P2125521 | ref|NM_007966|ens|ENSMUST00000031787|gb|X54239|tc|TC1595533 | Evx1 | 5.13 |
| A_51_P460643 | ref|NM_001079869|ref|NM_010458|ref|NM_010468|ens|ENSMUST00000055334 | Hoxb3 | 5.08 |
| A_30_P01028502 | Unknown | chr9:24292762-24307562_R | 5.05 |
| A_55_P2090874 | ref|NM_011737|nap|NAP058716-1 | Ysk4 | 5.05 |
| A_66_P117729 | ref|NM_001081136|ens|ENSMUST00000096361|gb|BC147680|gb|BC147683 | Awat1 | 5.05 |
| A_30_P01028619 | Unknown | chr13:112207480-112213280_F | 5.03 |
| A_30_P01025069 | Unknown | chr7:88668435-88674440_F | 5.03 |
| A_55_P2056792 | ens|ENSMUST00000109450|gb|AK006874|tc|TC1623948|riken|1700063K20 | ENSMUST00000109450 | 5.03 |
| A_51_P313157 | ref|NM_133674|ens|ENSMUST00000031750|gb|AK220242|gb|BC025127 | Arhgef5 | 5.00 |
| A_30_P01029417 | Unknown | chr4:132897264-132910714_R | 5.00 |
| A_51_P388471 | ref|NM_027094|ens|ENSMUST00000022315|gb|AK006511|gb|BC049632 | Dydc1 | 5.00 |
| A_55_P1965169 | ens|ENSMUST00000098385|gb|AK086977|tc|TC1594587|riken|E030017D19 | ENSMUST00000098385 | 4.98 |
| A_55_P1994720 | ref|XM_001472146|ref|XM_001477745|gb|BC064714|tc|TC1628564 | Gm5141 | 4.95 |
| A_51_P264825 | ref|NM_008479|ens|ENSMUST00000032217|gb|BC120591|gb|X98113 | Lag3 | 4.95 |
| A_55_P2199917 | gb|AK135741|riken|7420409G12 | AU043392 | 4.93 |
| A_51_P406204 | ref|NM_146671|ens|ENSMUST00000080460|gb|BC127972|tc|NP830691 | Olfr822 | 4.90 |
| A_55_P2181296 | ref|NM_198659|ens|ENSMUST00000062306|gb|AK046955|tc|TC1589486 | B930007M17Rik | 4.88 |
| A_65_P19832 | ref|NM_013697|ens|ENSMUST00000075312|gb|AK050155|gb|AK018701 | Ttr | 4.88 |
| A_30_P01026663 | Unknown | chr5:92548501-92566401_F | 4.85 |
| A_55_P2055607 | ref|NM_008813|ens|ENSMUST00000039882|gb|AK164619|gb|AK088857 | Enpp1 | 4.85 |
| A_30_P01020354 | Unknown | chr1:64232564-64349617_R | 4.83 |
| A_55_P2391185 | gb|DV044254 | AI195381 | 4.83 |
| A_66_P117519 | ens|ENSMUST00000099292|ref|XR_035472|ref|XR_035475|gb|AK144672 | ENSMUST00000099292 | 4.81 |
| A_51_P137913 | ref|NM_182993|ens|ENSMUST00000085374|gb|AK159064|gb|BC028938 | Slc17a7 | 4.81 |
| A_51_P107959 | ref|XR_033917|ref|XR_034525|gb|AK006389|tc|TC1590263 | 1700026J12Rik | 4.81 |
| A_55_P2222904 | gb|AK047086|tc|TC1621597|riken|B930018H19|nap|NAP083493-1 | B930018H19 | 4.81 |
| A_30_P01033345 | Unknown | chr16:59557120-59602070_R | 4.78 |
| A_66_P130703 | ref|XM_896593|ref|XM_922311|tc|TC1695792 | B230218P12Rik | 4.78 |
| A_55_P2026675 | ens|ENSMUST00000060774|ref|XM_001473105|ref|XM_001475024|gb|AK046413 | ENSMUST00000060774 | 4.78 |
| A_30_P01022239 | Unknown | chr14:44146705-44149729_R | 4.76 |
| A_55_P2078815 | ref|NM_023500|ens|ENSMUST00000015486|gb|AY534248|gb|AF155511 | Xk | 4.76 |
| A_52_P338605 | ref|XR_032468|gb|AK029958|tc|TC1624517|riken|4932411C10 | Gm6639 | 4.74 |
| A_55_P1998702 | ref|XM_001473020 | Gm2276 | 4.74 |
| A_52_P57317 | ref|NM_183224|ens|ENSMUST00000069296|ens|ENSMUST00000139783|gb|AK078681 | Fam19a3 | 4.72 |
| A_30_P01017664 | Unknown | chr3:70581406-70614856_F | 4.69 |
| A_55_P2143366 | ref|XM_001474047|nap|NAP018839-001 | LOC100044075 | 4.69 |
| A_55_P2080183 | ens|ENSMUST00000098988|tc|TC1741071 | ENSMUST00000098988 | 4.65 |
| A_66_P108918 | ref|NM_001011836|ref|NM_146838|ens|ENSMUST00000081034|ens|ENSMUST00000079711 | Olfr1129 | 4.65 |
| A_55_P2075364 | ref|XM_001472141|ref|XM_001472908|ref|XM_001473097|ref|XM_001475486 | LOC100039041 | 4.63 |
| A_55_P2145601 | ref|NM_008474|ens|ENSMUST00000023720|gb|AY028607|gb|X65505 | Krt84 | 4.59 |
| A_52_P609868 | ref|NM_178759|ens|ENSMUST00000068877|gb|AK080212|gb|AK079787 | Timd4 | 4.59 |
| A_55_P2053439 | ref|NM_008412|ens|ENSMUST00000107316|ens|ENSMUST00000053107|gb|BC160201 | Ivl | 4.59 |
| A_51_P453197 | ens|ENSMUST00000062846|gb|AK006458|gb|AK077091|tc|NP357798 | 12-Sep | 4.52 |
| A_30_P01020100 | Unknown | chr2:116802549-116803286_R | 4.50 |
| A_55_P1991811 | ref|NM_001024230|ens|ENSMUST00000109212|ens|ENSMUST00000109210|gb|AK144287 | Gm5431 | 4.50 |
| A_55_P2041614 | ref|NM_001048176|ens|ENSMUST00000143974|ens|ENSMUST00000156731|gb|BC046474 | Cerkl | 4.50 |
| A_55_P2175056 | ref|XM_001475940|tc|TC1645953 | LOC100046053 | 4.50 |
| A_55_P2127482 | ens|ENSMUST00000033688|ens|ENSMUST00000062544|ens|ENSMUST00000127012|ens|ENSMUST00000061184 | ENSMUST00000033688 | 4.50 |
| A_51_P128075 | ref|NM_001163810|gb|AK005787|tc|TC1587596|riken|1700008P20 | 1700008P20Rik | 4.44 |
| A_55_P2069724 | ref|NM_001033239|ens|ENSMUST00000096090|gb|AK137702|tc|TC1585337 | Csta | 4.44 |
| A_55_P1968963 | ens|ENSMUST00000103480|nap|NAP100420-001 | ENSMUST00000103480 | 4.37 |
| A_55_P2163446 | ref|XR_002216|ref|XR_033797|nap|NAP062870-1 | Gm4973 | 4.37 |
| A_55_P2032698 | ref|XR_035092|ref|XR_035093|gb|AK050630|tc|TC1590086 | Gm4814 | 4.37 |
| A_55_P2126532 | ref|NM_146891|ens|ENSMUST00000099791|gb|BC145849|tc|TC1600043 | Olfr1225 | 4.35 |
| A_52_P460791 | ref|NM_008918|ens|ENSMUST00000017460|gb|BC101946|gb|BC100676 | Ppy | 4.33 |
| A_30_P01019130 | Unknown | chr9:118060025-118085050_R | 4.31 |
| A_52_P253567 | ref|NM_013821|ens|ENSMUST00000029463|gb|AB109387|gb|AK142267 | Hsd3b6 | 4.31 |
| A_55_P1963590 | ens|ENSMUST00000112745|gb|CO809669|tc|TC1770112 | ENSMUST00000112745 | 4.29 |
| A_55_P2055247 | ref|NM_205783|ens|ENSMUST00000099589|gb|BC120615|tc|TC1654128 | Chrm5 | 4.29 |
| A_55_P2306794 | gb|AK037657|gb|BB400609|tc|TC1618995|riken|A130034L04 | 9030417H13Rik | 4.29 |
| A_30_P01032440 | Unknown | chr17:29383810-29384205_F | 4.27 |
| A_30_P01030549 | Unknown | chr8:12278818-12280657_F | 4.27 |
| A_55_P1957669 | ref|NM_030734|ens|ENSMUST00000110763|ens|ENSMUST00000038948|gb|AF318068 | Defb5 | 4.26 |
| A_51_P125205 | ref|NM_007472|ens|ENSMUST00000004774|gb|AK157333|gb|AK081886 | Aqp1 | 4.26 |
| A_55_P2108165 | ref|NM_001164289|gb|EU155100|gb|EU155105|gb|EU155103 | Gm6907 | 4.26 |
| A_52_P541161 | ref|NM_022881|ens|ENSMUST00000027603|gb|AK036407|gb|BC099387 | Rgs18 | 4.26 |
| A_55_P2027852 | ref|NR_033527|ens|ENSMUST00000098949|ens|ENSMUST00000127460|ens|ENSMUST00000069762 | Ccl25 | 4.24 |
| A_52_P15377 | ref|NM_011719|ens|ENSMUST00000018630|gb|AK052708|tc|TC1642958 | Wnt9b | 4.22 |
| A_55_P2088075 | ref|XM_987199|tc|TC1626843 | LOC676066 | 4.22 |
| A_30_P01033577 | Unknown | chr3:145269449-145298239_R | 4.20 |
| A_55_P2144140 | ens|ENSMUST00000100734|ref|XM_001477259|ref|XM_001478669|gb|AK133080 | Gm10386 | 4.18 |
| A_55_P1969176 | ref|XM_001473558|ref|XM_001479812 | B130016D09Rik | 4.18 |
| A_55_P2321979 | gb|BG070544 | AI314760 | 4.18 |
| A_30_P01022470 | Unknown | chr8:74439200-74441363_F | 4.17 |
| A_51_P109097 | ref|NM_146835|ens|ENSMUST00000031086|gb|BC130246|tc|NP646758 | Olfr109 | 4.15 |
| A_30_P01026327 | Unknown | chr2:119412770-119435110_R | 4.15 |
| A_55_P2274837 | gb|AK049102|riken|C230099C02|nap|NAP019778-001 | A830029E22Rik | 4.13 |
| A_30_P01024506 | Unknown | chr14:43418592-43428090_F | 4.13 |
| A_55_P2074085 | ref|NM_001077410|ref|NM_212486|ens|ENSMUST00000078223|gb|AK155985 | Gimap8 | 4.13 |
| A_55_P2188862 | gb|CX239135 | C030034E14Rik | 4.12 |
| A_55_P1987675 | ref|XM_001472024|ref|XM_001472350 | Gm2001 | 4.12 |
| A_55_P2164055 | ref|NM_001110506|ens|ENSMUST00000032468|gb|BC060267|tc|TC1584269 | BC060267 | 4.10 |
| A_55_P2104343 | ens|ENSMUST00000097807|ref|XM_887803|ref|XM_001476667|gb|AK131727 | Gm6420 | 4.10 |
| A_55_P2148056 | ref|XR_030499|ref|XR_030515 | Gm7341 | 4.05 |
| A_55_P2302750 | gb|C79829 | C80120 | 4.05 |
| A_55_P2177351 | ref|NM_008066|ens|ENSMUST00000000572|ref|XM_001478553|gb|AK039055 | Gabra2 | 4.02 |
| A_55_P2065601 | ref|NM_177704|ens|ENSMUST00000086165|ens|ENSMUST00000067529|gb|BC150158 | Sytl5 | 4.02 |
| A_55_P2066672 | ref|NM_177813|ens|ENSMUST00000060526|gb|AK083193|tc|TC1592453 | Gm5087 | 4.02 |
| A_30_P01030821 | Unknown | chr12:12876192-12918398_R | 4.00 |
| A_55_P2234964 | gb|AI987986 | AI987986 | 4.00 |
| A_55_P2098499 | ref|NM_001038015|ens|ENSMUST00000031117|gb|AK220275|gb|AK168516 | Gnpda2 | 4.00 |
| A_52_P686785 | ref|NM_053247|ens|ENSMUST00000033050|gb|AK004182|gb|AK004726 | Lyve1 | 3.98 |
| A_51_P443314 | ref|NM_029294|gb|AK077203|gb|BC109355|tc|TC1585939 | Prps1l1 | 3.95 |
| A_55_P2316041 | gb|BC050972|gb|BU961845|tc|TC1662429|tc|TC1679757 | BC050972 | 3.95 |
| A_55_P2091592 | ref|NM_001168356|ref|NM_134253|ens|ENSMUST00000137250|ens|ENSMUST00000125515 | Bnipl | 3.94 |
| A_55_P1997132 | ref|XM_001475349 | LOC100044017 | 3.94 |
| A_30_P01028022 | Unknown | chr4:53217935-53230433_R | 3.92 |
| A_51_P405912 | ref|NM_144799|ens|ENSMUST00000032376|gb|AK159946|gb|AK075847 | Lmcd1 | 3.92 |
| A_52_P201531 | ref|NM_173018|ens|ENSMUST00000136740|ens|ENSMUST00000135298|ens|ENSMUST00000128341 | Myo9a | 3.92 |
| A_55_P2155264 | ref|XM_001480028|ref|XM_001475728 | Gm7147 | 3.91 |
| A_30_P01019103 | Unknown | chr12:53708656-53729799_F | 3.91 |
| A_30_P01032443 | Unknown | chr5:121759042-121789992_F | 3.89 |
| A_52_P194971 | ref|NM_010460|ens|ENSMUST00000049352|gb|X06762|tc|TC1585082 | Hoxb7 | 3.89 |
| A_51_P334155 | ref|NM_001163385|ref|NM_001163386|ens|ENSMUST00000012186|gb|AK016318 | 4930579F01Rik | 3.89 |
| A_55_P2053132 | ens|ENSMUST00000110746|ref|XR_033869|gb|AK042354|gb|BB240998 | ENSMUST00000110746 | 3.88 |
| A_51_P325254 | ens|ENSMUST00000117489|gb|BC002139|gb|AK134431|tc|TC1584465 | ENSMUST00000117489 | 3.88 |
| A_66_P105288 | ref|NM_007475|ens|ENSMUST00000086519|gb|AK009767|gb|AK010267 | Rplp0 | 3.88 |
| A_55_P2063076 | ref|XM_001477502 | LOC100047144 | 3.86 |
| A_55_P2179151 | ens|ENSMUST00000100658|gb|AK157412|riken|F830215A15 | ENSMUST00000100658 | 3.86 |
| A_51_P254855 | ref|NM_011198|ens|ENSMUST00000035065|gb|AK149820|gb|M64291 | Ptgs2 | 3.83 |
| A_55_P2111394 | ens|ENSMUST00000032132|gb|AB041550|tc|TC1592892|nap|NAP018765-001 | Ccdc48 | 3.83 |
| A_55_P2182517 | gb|BC058422|tc|TC1602367|nap|NAP000001-041|nap|NAP113262-1 | BC058422 | 3.82 |
| A_55_P2341358 | gb|AK133035|riken|4930558C16 | 4930452L02Rik | 3.82 |
| A_52_P43150 | ref|NM_007519|ens|ENSMUST00000043056|gb|BC012683|gb|AK017923 | Baat | 3.82 |
| A_55_P2366358 | gb|AK086457|riken|D930030E17|nap|NAP087407-1 | 4930444F02Rik | 3.82 |
| A_55_P2106725 | ref|NM_001008429|ens|ENSMUST00000045152|gb|BC139171|gb|BC139172 | Taar3 | 3.80 |
| A_51_P171075 | ref|NM_009969|ens|ENSMUST00000019060|gb|X02333|gb|X03221 | Csf2 | 3.77 |
| A_30_P01032224 | Unknown | chr8:123571640-123607481_F | 3.77 |
| A_51_P423290 | ref|NM_027613|ref|NM_001163507|ens|ENSMUST00000067803|ens|ENSMUST00000129603 | Mmrn1 | 3.76 |
| A_52_P257041 | ref|NM_153561|ens|ENSMUST00000099130|ens|ENSMUST00000052645|ens|ENSMUST00000108118 | Nudt6 | 3.76 |
| A_55_P2111980 | ref|NM_008295|ens|ENSMUST00000044094|gb|L41519|gb|BC012715 | Hsd3b5 | 3.76 |
| A_30_P01033228 | Unknown | chr9:88575075-88611650_R | 3.75 |
| A_55_P2399208 | gb|AK172666|riken|F830226J21 | 4930472D12Rik | 3.73 |
| A_66_P117254 | ens|ENSMUST00000070942|ref|XR_035386|gb|AK041266|tc|TC1593837 | ENSMUST00000070942 | 3.73 |
| A_51_P283344 | ref|NM_025956|ens|ENSMUST00000022398|gb|AK005866|gb|BC026534 | 1700011H14Rik | 3.73 |
| A_55_P2026950 | ens|ENSMUST00000101381|tc|TC1706519 | ENSMUST00000101381 | 3.70 |
| A_55_P1966634 | ref|NM_001102578|ref|NM_001105187|nap|NAP092187-001|nap|NAP113640-1 | Vmn2r75 | 3.70 |
| A_30_P01017640 | Unknown | chr11:76427968-76435706_R | 3.70 |
| A_55_P2017054 | ens|ENSMUST00000103487|nap|NAP013673-001 | ENSMUST00000103487 | 3.69 |
| A_55_P2147706 | ref|NM_001011835|ens|ENSMUST00000099837|tc|NP1398925|nap|NAP112949-1 | Olfr1162 | 3.69 |
| A_55_P2147260 | ref|NM_177816|ens|ENSMUST00000096000|gb|AX776199|gb|BC138964 | Sh2d4b | 3.69 |
| A_55_P1969575 | ens|ENSMUST00000098926|gb|AK144276|riken|G630005L12 | ENSMUST00000098926 | 3.68 |
| A_55_P2148185 | ref|XM_977642|tc|TC1704642|tc|TC1677461 | Gm7677 | 3.68 |
| A_55_P2007878 | ref|NM_146042|ref|NM_001170643|ens|ENSMUST00000110111|ens|ENSMUST00000068891 | Rnf144b | 3.66 |
| A_55_P2117119 | ref|NM_028889|ens|ENSMUST00000118687|ens|ENSMUST00000027472|gb|AK171846 | Efhd1 | 3.65 |
| A_55_P1961799 | ref|XM_001476682|ref|XM_001476875|gb|BY708968 | Gm3235 | 3.62 |
| A_30_P01025900 | Unknown | chr8:24329249-24329541_F | 3.62 |
| A_55_P2002975 | ref|NM_205795|ens|ENSMUST00000094388|gb|BC107202|gb|BC107203 | Mrgprb4 | 3.61 |
| A_55_P2075120 | ens|ENSMUST00000066157|gb|AK046715|tc|TC1595872|riken|B430320O11 | ENSMUST00000066157 | 3.61 |
| A_55_P2397211 | ref|NM_028317|ens|ENSMUST00000142856|ens|ENSMUST00000066885|ens|ENSMUST00000091420 | 2810030E01Rik | 3.61 |
| A_55_P2009767 | ens|ENSMUST00000068453|gb|AK078977|tc|TC1589278|riken|9130604K18 | ENSMUST00000068453 | 3.58 |
| A_55_P1999274 | ref|XM_001474757 | LOC100045690 | 3.58 |
| A_66_P140973 | ref|NM_001033799|ens|ENSMUST00000101419|gb|AK132827|tc|TC1593990 | 4930428D18Rik | 3.58 |
| A_30_P01026777 | Unknown | chr8:19959345-20020379_R | 3.57 |
| A_30_P01030148 | Unknown | chr6:31233512-31241062_F | 3.57 |
| A_51_P378336 | ref|NM_172687|ens|ENSMUST00000029909|gb|AK028680|gb|AK142287 | Coq3 | 3.56 |
| A_55_P2121511 | ref|XM_001475060|ref|XM_001477832|gb|AK079063|riken|9330119I17 | AI849053 | 3.56 |
| A_30_P01019407 | Unknown | chr6:148173524-148174740_F | 3.56 |
| A_30_P01021557 | Unknown | chr13:66888650-66902600_R | 3.56 |
| A_55_P2057972 | ens|ENSMUST00000092214|gb|DQ025533 | ENSMUST00000092214 | 3.56 |
| A_55_P2045777 | ref|XM_001475033|ref|XM_001478809|nap|NAP095730-001 | Gm7902 | 3.55 |
| A_55_P2074762 | ref|NM_010702|ens|ENSMUST00000109886|ens|ENSMUST00000062806|gb|AB009688 | Lect2 | 3.53 |
| A_55_P1989389 | ref|XM_001479289|nap|NAP063229-1 | LOC100047984 | 3.53 |
| A_55_P1970893 | ref|NM_146750|ens|ENSMUST00000098153|gb|BC119379|nap|NAP098302-001 | Olfr689 | 3.53 |
| A_55_P1958976 | ref|NM_001004157|ens|ENSMUST00000118243|ens|ENSMUST00000071964|ens|ENSMUST00000042808 | Scarf1 | 3.52 |
| A_55_P2170673 | ens|ENSMUST00000074699|gb|BC021366|tc|TC1657344|tc|TC1600762 | ENSMUST00000074699 | 3.52 |
| A_51_P351413 | ref|NM_028521|ens|ENSMUST00000028494|ens|ENSMUST00000112266|gb|BC025612 | Phospho2 | 3.52 |
| A_30_P01028351 | Unknown | chr14:77258025-77278750_F | 3.52 |
| A_30_P01028618 | Unknown | chr12:89448550-89461843_F | 3.50 |
| A_51_P284503 | ref|XM_897249|ref|XM_925070|gb|AK004025|tc|TC1694027 | Krtap22-2 | 3.50 |
| A_51_P100625 | ref|NM_133996|ens|ENSMUST00000105239|ens|ENSMUST00000060782|gb|AK083289 | Apon | 3.47 |
| A_30_P01027307 | Unknown | chr4:33369310-33369871_R | 3.47 |
| A_30_P01018005 | Unknown | chr15:76193801-76194225_F | 3.46 |
| A_55_P2182372 | ref|XM_915096|ref|XM_976192 | Gm7254 | 3.46 |
| A_55_P2036693 | ref|NM_011611|ref|NM_170704|ref|NM_170703|ref|NM_170702 | Cd40 | 3.46 |
| A_30_P01031430 | Unknown | chr1:71939741-71947891_R | 3.46 |
| A_30_P01017642 | Unknown | chr18:6144775-6202105_R | 3.46 |
| A_52_P40293 | ref|NM_013547|ref|NR_027778|ens|ENSMUST00000023519|gb|AK002584 | Hgd | 3.45 |
| A_55_P2395018 | ref|NM_001128146|ref|NM_001128145|gb|EF624462|gb|EF624463 | 5830411N06Rik | 3.44 |
| A_55_P2163541 | ref|NM_177872|ref|NM_001081401|ens|ENSMUST00000061427 | Adamts3 | 3.42 |
| A_30_P01018823 | Unknown | chr7:30645026-30667034_R | 3.42 |
| A_30_P01031601 | Unknown | chr1:121971906-121988306_R | 3.42 |
| A_30_P01019249 | Unknown | chr3:68867193-68876128_F | 3.41 |
| A_52_P388780 | ref|NM_001164166|gb|AK160151|tc|TC1669462|riken|1700054H16 | Pom121l12 | 3.41 |
| A_30_P01025431 | Unknown | chr19:44653017-44654777_F | 3.40 |
| A_52_P582318 | ref|XM_001476862|ref|XM_001478622|gb|AK006595|gb|AK076925 | 1700034H15Rik | 3.39 |
| A_30_P01023688 | Unknown | chr2:94008913-94019798_R | 3.39 |
| A_55_P1964247 | ref|XM_001473949 | LOC100045264 | 3.39 |
| A_55_P2150471 | ref|XM_001473879|ref|XM_001476765 | XM_001473879 | 3.39 |
| A_30_P01025940 | Unknown | chr2:174982600-175005825_R | 3.38 |
| A_30_P01027865 | Unknown | chr12:72103832-72116875_R | 3.38 |
| A_55_P2050962 | ref|XM_001479668 | LOC100048168 | 3.38 |
| A_52_P318584 | ref|NM_183104|ens|ENSMUST00000070831|gb|AK016488|gb|BB013503 | 4931429L15Rik | 3.38 |
| A_30_P01020219 | Unknown | chr15:43363046-43364763_F | 3.36 |
| A_66_P132193 | ref|XM_001001008|ref|XM_986694|gb|AK029404|tc|TC1622658 | Gm536 | 3.36 |
| A_51_P246627 | ref|NM_028547|ens|ENSMUST00000061019|gb|AK030188|gb|AK006866 | Kif2b | 3.36 |
| A_52_P5190 | ref|NM_172258|ens|ENSMUST00000108877|ens|ENSMUST00000069816|ens|ENSMUST00000020502 | Slc36a3 | 3.34 |
| A_55_P2052066 | ref|XM_001473267|ref|XM_001472348 | Gm2082 | 3.34 |
| A_55_P2115832 | ref|NM_011253|ref|NM_001166384|ens|ENSMUST00000100360|ens|ENSMUST00000092052 | Rbmy1a1 | 3.33 |
| A_55_P1957163 | ref|XM_001473071|ref|XM_001475011|tc|TC1643136 | Gm2297 | 3.32 |
| A_55_P2232023 | gb|BB756663 | AI448005 | 3.31 |
| A_66_P127223 | ref|NR_003635|ens|ENSMUST00000068874|gb|AK016585|tc|TC1590600 | 4933400A11Rik | 3.30 |
| A_55_P2064014 | ref|NM_028671|ens|ENSMUST00000114835|ref|XM_001478102|gb|AK015916 | Fam122c | 3.30 |
| A_55_P1953753 | ref|NM_007726|ens|ENSMUST00000084736|ens|ENSMUST00000057188|gb|AK136514 | Cnr1 | 3.30 |
| A_55_P2164265 | ref|NM_026206|ens|ENSMUST00000072943|gb|AF525157|gb|AK014436 | Prl7c1 | 3.29 |
| A_30_P01025511 | Unknown | chr4:149445201-149445902_F | 3.29 |
| A_55_P2134992 | ref|XR_035365|ref|XR_035428|gb|AK155420|gb|AK169472 | Golgb1 | 3.28 |
| A_30_P01029785 | Unknown | chr15:59619971-59626514_R | 3.28 |
| A_51_P513460 | ref|NM_015780|ens|ENSMUST00000023965|gb|BC111870|gb|M29008 | Cfhr1 | 3.27 |
| A_52_P413502 | ref|NM_025542|ens|ENSMUST00000029005|gb|AK053258|gb|AK077823 | 2410001C21Rik | 3.26 |
| A_55_P1979242 | ref|NM_134110|ens|ENSMUST00000113971|ens|ENSMUST00000047383|gb|AK008619 | Kcne2 | 3.25 |
| A_55_P1976237 | ref|NM_008299|ens|ENSMUST00000115975|ens|ENSMUST00000119972|gb|AK005690 | Dnajb3 | 3.25 |
| A_55_P2057741 | ens|ENSMUST00000066183|ref|XR_035354|ref|XR_035397|gb|AK005254 | ENSMUST00000066183 | 3.25 |
| A_30_P01029282 | Unknown | chr2:176908707-176991202_R | 3.24 |
| A_55_P1998115 | ref|NM_008455|ens|ENSMUST00000026907|ens|ENSMUST00000116473|gb|M58588 | Klkb1 | 3.24 |
| A_55_P2059105 | ens|ENSMUST00000110230|ens|ENSMUST00000100659|gb|BC099610|tc|TC1707632 | ENSMUST00000110230 | 3.24 |
| A_30_P01032778 | Unknown | chr3:35782698-35789748_F | 3.23 |
| A_55_P2117760 | ens|ENSMUST00000105012|ref|XR_031864|gb|BC115856 | ENSMUST00000105012 | 3.23 |
| A_52_P533265 | ref|NM_177711|gb|AK077018|tc|TC1589904|riken|4932411G14 | 4932411G14Rik | 3.23 |
| A_30_P01020931 | Unknown | chr7:69024136-69131056_R | 3.22 |
| A_55_P1991244 | ref|NM_001081424|ens|ENSMUST00000084830|gb|AK033068|tc|TC1618142 | Chrna10 | 3.22 |
| A_55_P2404658 | gb|AK040843|gb|FJ422280|tc|TC1706471|riken|A530029P05 | LOC100216343 | 3.22 |
| A_55_P2108708 | ref|NM_020574|ens|ENSMUST00000049333|ref|XM_001472865|gb|AK132773 | Kcne3 | 3.22 |
| A_55_P2118436 | ref|XM_974183|gb|X03052|gb|AK153910|riken|E430004M04 | LOC674370 | 3.22 |
| A_52_P262338 | ref|NR_033492|gb|AK031340|gb|AV319627|tc|TC1600449 | 4931406H21Rik | 3.21 |
| A_30_P01031346 | Unknown | chr1:36094831-36095159_R | 3.19 |
| A_30_P01032964 | Unknown | chr16:40552116-40552742_F | 3.19 |
| A_66_P126242 | ref|NM_146711|ens|ENSMUST00000078936|gb|BC125464|gb|BC125468 | Olfr43 | 3.19 |
| A_30_P01033188 | Unknown | chr19:54011403-54018978_R | 3.19 |
| A_52_P199633 | ref|NM_199146|ref|NM_001167828|ens|ENSMUST00000141063|ens|ENSMUST00000033211 | Trim79 | 3.18 |
| A_55_P2186757 | ref|NM_001011736|ens|ENSMUST00000074125|ens|ENSMUST00000086788|gb|BC150839 | Olfr205 | 3.18 |
| A_52_P62617 | ens|ENSMUST00000065159|gb|AK028012|gb|BB609792|tc|TC1673422 | ENSMUST00000065159 | 3.18 |
| A_55_P2031841 | ref|XM_001480868 | Gm4620 | 3.17 |
| A_30_P01024219 | Unknown | chr4:59093960-59122541_R | 3.16 |
| A_55_P2005512 | ref|NM_146451|ens|ENSMUST00000056727|gb|BC145676|tc|TC1599883 | Olfr164 | 3.16 |
| A_52_P669682 | ref|XM_001001994|ref|XR_035612|nap|NAP102285-1|nap|NAP112859-1 | Gm7225 | 3.16 |
| A_55_P1965278 | ref|NM_027591|ref|NM_001038616|ref|NM_029378|ens|ENSMUST00000033686 | Dmrtc1a | 3.16 |
| A_51_P245090 | ref|NM_016689|ens|ENSMUST00000055327|gb|AF104416|gb|AK160929 | Aqp3 | 3.16 |
| A_51_P310821 | ref|NM_010453|ens|ENSMUST00000048794|gb|X16840|gb|M36604 | Hoxa5 | 3.13 |
| A_55_P2007210 | ref|NM_177213|ens|ENSMUST00000121265|ens|ENSMUST00000076272|gb|AY243471 | Abca15 | 3.13 |
| A_55_P2245317 | gb|AK131745|riken|1700061E17 | 1700061E17Rik | 3.13 |
| A_55_P2360356 | gb|AK016599|tc|NP741622|riken|4933401D09|nap|NAP073396-1 | 4933401D09Rik | 3.12 |
| A_55_P2342444 | gb|AK084672|tc|TC1736600|riken|D330027L19|nap|NAP086335-1 | D330022K07Rik | 3.12 |
| A_66_P135601 | ref|XM_001473655|ref|XM_001477485 | Gm8420 | 3.12 |
| A_55_P1989321 | ref|NM_009121|ens|ENSMUST00000026318|ens|ENSMUST00000112551|ref|XM_485586 | Sat1 | 3.12 |
| A_52_P518997 | ref|NM_010139|ens|ENSMUST00000006614|gb|AK144202|gb|AK137704 | Epha2 | 3.11 |
| A_51_P222359 | ref|NM_133918|ens|ENSMUST00000031055|gb|AK140388|gb|AK155486 | Emilin1 | 3.11 |
| A_55_P2003596 | ref|XM_001472384|tc|TC1681662 | LOC100048880 | 3.11 |
| A_52_P264790 | ref|NM_027924|gb|AF335583|gb|AK003359|tc|TC1593939 | Pdgfd | 3.11 |
| A_55_P2131805 | ref|NR_033542|ens|ENSMUST00000098523|gb|AK135478|riken|6820427K09 | Gm10636 | 3.10 |
| A_55_P2155161 | nap|NAP013642-001 | NAP013642-001 | 3.10 |
| A_30_P01022059 | Unknown | chr3:27848923-27856325_F | 3.09 |
| A_51_P480241 | ref|NM_007921|ref|NM_001163131|ens|ENSMUST00000003135|gb|BC145380 | Elf3 | 3.08 |
| A_55_P1981210 | ref|NM_028730|ens|ENSMUST00000120066|ens|ENSMUST00000118234|ens|ENSMUST00000088561 | Pex26 | 3.08 |
| A_52_P144263 | ref|NM_020296|ref|NM_001141931|ref|NM_001141932|ens|ENSMUST00000074547 | Rbms1 | 3.07 |
| A_55_P2178094 | ref|XM_001474967|ref|XM_001476894 | Gm3484 | 3.06 |
| A_55_P2135341 | ref|NM_029686|ens|ENSMUST00000109093|gb|AY164484|tc|TC1685057 | Pkd1l2 | 3.06 |
| A_55_P2028305 | ref|NM_001167757|ref|NM_029202|ens|ENSMUST00000115396|ens|ENSMUST00000031489 | Ankrd7 | 3.06 |
| A_55_P1953371 | ref|XR_032428|ref|XR_033790 | Vmn1r-ps20 | 3.06 |
| A_30_P01018811 | Unknown | chr13:98270713-98272269_F | 3.06 |
| A_66_P132070 | ref|NM_175456|ens|ENSMUST00000054742|gb|AK081696|gb|AK045186 | Abra | 3.06 |
| A_52_P216226 | ref|NM_008555|gb|BC131637|gb|BC131638|tc|TC1618198 | Masp1 | 3.05 |
| A_51_P205215 | ref|NM_027551|ens|ENSMUST00000027533|gb|AK086025|gb|AK014532 | Klhl30 | 3.05 |
| A_55_P2068184 | ref|XM_001473044|ref|XM_001473253|tc|TC1615311 | XM_001473044 | 3.05 |
| A_55_P2179271 | ref|NM_029286|ens|ENSMUST00000044781|ens|ENSMUST00000030386|ens|ENSMUST00000063642 | Ccdc30 | 3.05 |
| A_55_P2095133 | ref|NM_080451|ens|ENSMUST00000106427|ens|ENSMUST00000106426|ens|ENSMUST00000139160 | Synpo2 | 3.05 |
| A_30_P01033083 | Unknown | chr18:84747776-84748219_F | 3.05 |
| A_30_P01031108 | Unknown | chr9:65651848-65652920_R | 3.04 |
| A_66_P101732 | ref|NM_009936|ens|ENSMUST00000103059|gb|BC030945|gb|AF349718 | Col9a3 | 3.03 |
| A_55_P2083454 | ens|ENSMUST00000100346|gb|AK131846|riken|1810048O14 | ENSMUST00000100346 | 3.02 |
| A_55_P2163428 | ref|NM_008021|ens|ENSMUST00000112148|ens|ENSMUST00000073316|gb|AK215575 | Foxm1 | 3.02 |
| A_30_P01019690 | Unknown | chr7:140017838-140017923_F | 3.02 |
| A_55_P1988433 | ref|NM_139308|ens|ENSMUST00000069099|ens|ENSMUST00000110375|ens|ENSMUST00000028845 | Stard7 | 3.02 |
| A_55_P2136686 | ens|ENSMUST00000109054|gb|AK164563|riken|D330045G02 | Gm14443 | 3.00 |
| A_51_P389156 | ref|NM_054052|ref|NM_001159407|ref|NM_001159408|ens|ENSMUST00000119468 | B3gnt5 | 3.00 |
| A_30_P01023937 | Unknown | chr9:27148989-27155714_F | 3.00 |
| A_30_P01017562 | Unknown | chr1:44922628-44923224_F | 2.99 |
| A_30_P01017727 | Unknown | chr12:4579206-4583562_R | 2.99 |
| A_55_P2361731 | gb|BU848224|tc|TC1678489 | AW047481 | 2.99 |
| A_55_P2217548 | gb|AK014899|riken|4921515G04|nap|NAP073252-1 | 4921515G04Rik | 2.98 |
| A_55_P1994746 | ref|XM_001480359|gb|AK016152|riken|4930556N09|nap|NAP074610-1 | 4930556N09Rik | 2.98 |
| A_55_P1970900 | ref|NM_001033498|ens|ENSMUST00000098661|ens|ENSMUST00000114056|gb|BC138948 | Gramd2 | 2.98 |
| A_55_P2075200 | ref|NM_023141|ens|ENSMUST00000122242|ens|ENSMUST00000079625|ref|XM_001479238 | Tor3a | 2.97 |
| A_52_P200925 | ref|XR_031141|ref|XR_033570|nap|NAP061138-1|nap|NAP111052-1 | Gm8864 | 2.96 |
| A_51_P450888 | ref|NM_022982|ens|ENSMUST00000059589|gb|AF283462|gb|BC052317 | Rtn4r | 2.96 |
| A_55_P2108422 | ref|XM_001472519 | Gm12134 | 2.95 |
| A_55_P2110758 | ref|NM_177083|ens|ENSMUST00000125174|gb|AK133242|gb|AK170621 | B430306N03Rik | 2.95 |
| A_52_P129428 | ref|NM_020510|gb|AK048420|gb|AK135363|gb|AF206322 | Fzd2 | 2.94 |
| A_51_P480861 | ref|NM_021453|ens|ENSMUST00000025647|gb|AF240776|gb|BC119523 | Pga5 | 2.94 |
| A_55_P2182187 | ref|NM_009719|ens|ENSMUST00000050103|gb|AK008017|tc|TC1592863 | Neurog3 | 2.94 |
| A_52_P348847 | ens|ENSMUST00000107875|gb|AK012293|tc|TC1602742|riken|2700027C09 | ENSMUST00000107875 | 2.93 |
| A_55_P1981030 | ens|ENSMUST00000114743|tc|TC1655950 | ENSMUST00000114743 | 2.93 |
| A_30_P01023301 | Unknown | chr4:146889506-146890173_F | 2.93 |
| A_30_P01031630 | Unknown | chr1:23334791-23335367_F | 2.93 |
| A_52_P635105 | ens|ENSMUST00000093819|gb|AK034716|tc|TC1584634|riken|9430028F23 | ENSMUST00000093819 | 2.93 |
| A_51_P147361 | ens|ENSMUST00000058713|gb|AK054382|gb|AK135843|gb|BC099944 | ENSMUST00000058713 | 2.92 |
| A_55_P2079806 | ref|NM_146773|ens|ENSMUST00000072914|gb|BC130241|tc|TC1642234 | Olfr508 | 2.92 |
| A_52_P588378 | ens|ENSMUST00000068905|gb|AB041801|gb|AK043501|tc|TC1592454 | ENSMUST00000068905 | 2.92 |
| A_52_P358651 | ref|NM_027454|ref|NM_173212|ens|ENSMUST00000060943|ens|ENSMUST00000079463 | Chrnb3 | 2.92 |
| A_30_P01030396 | Unknown | chr4:131903928-131909578_R | 2.92 |
| A_55_P2073692 | ens|ENSMUST00000096145|tc|TC1574445 | ENSMUST00000096145 | 2.92 |
| A_30_P01026733 | Unknown | chr6:95418306-95424131_R | 2.91 |
| A_55_P2060604 | ref|NM_001025085|ref|XM_001475800|ref|XM_001476364|ref|XM_001476557 | Gm5797 | 2.91 |
| A_30_P01020493 | Unknown | chr14:44151048-44156924_R | 2.91 |
| A_30_P01019073 | Unknown | chr2:173420987-173480087_F | 2.91 |
| A_55_P2179206 | ref|NM_027394|ens|ENSMUST00000034986|gb|AK049229|tc|TC1585477 | Ube2cbp | 2.91 |
| A_55_P1962400 | ref|NM_001039701|ref|NM_031167|ref|NM_001159562|ens|ENSMUST00000114486 | Il1rn | 2.90 |
| A_55_P2133963 | ref|NM_146760|ens|ENSMUST00000050599|gb|BC160235|nap|NAP100458-001 | Olfr672 | 2.89 |
| A_55_P1988065 | ref|XM_001477121|ref|XM_001477576|gb|AK144329|tc|TC1675562 | Gm3546 | 2.89 |
| A_55_P1965732 | ref|XM_001000855|ref|XM_994273 | E130314M14Rik | 2.89 |
| A_55_P2026913 | ref|NM_001166843|ref|NM_001166721 | Vmn1r169 | 2.88 |
| A_30_P01027374 | Unknown | chr15:85476512-85498105_F | 2.88 |
| A_66_P103510 | ref|XM_887818|ref|XM_915476|nap|NAP024664-001 | Gm6422 | 2.88 |
| A_55_P2140328 | ref|XM_887930|ref|XM_907093 | Gm6434 | 2.87 |
| A_52_P295104 | ref|NM_183259|ens|ENSMUST00000052422|ref|XM_001478797|ref|XM_001478793 | 2210020M01Rik | 2.87 |
| A_55_P2384671 | gb|AK033123|gb|BB065047|riken|8030441M13|nap|NAP124541-1 | AK033123 | 2.87 |
| A_55_P2085531 | ref|NM_146643|ens|ENSMUST00000099843|tc|TC1600160|nap|NAP021220-001 | Olfr1155 | 2.87 |
| A_66_P132491 | ens|ENSMUST00000100683|gb|AK148391|tc|TC1645642|riken|G370126I09 | ENSMUST00000100683 | 2.86 |
| A_55_P2085240 | ref|XM_001472390|ref|XM_001474636 | LOC100044102 | 2.86 |
| A_52_P6524 | ref|NM_001099277|ens|ENSMUST00000108509|gb|BC157962|gb|DQ864732 | Zfp541 | 2.86 |
| A_55_P1954388 | ref|NM_001105185|ref|XM_913272|nap|NAP025578-001 | Vmn2r72-ps | 2.85 |
| A_30_P01030303 | Unknown | chr4:153321746-153324612_R | 2.85 |
| A_51_P170562 | ref|NM_027904|gb|BC025836|gb|BC081550|gb|BC054470 | Cpn2 | 2.85 |
| A_55_P1982329 | ref|XM_001004541|ref|XM_991889 | Gm9422 | 2.85 |
| A_55_P2382741 | gb|AA473072|tc|TC1680442 | AI314604 | 2.84 |
| A_30_P01030212 | Unknown | chr12:85252300-85253597_R | 2.84 |
| A_51_P168630 | ref|NM_010635|ens|ENSMUST00000067060|gb|BC114978|gb|AK158037 | Klf1 | 2.84 |
| A_30_P01019497 | Unknown | chr7:48853209-48886010_R | 2.84 |
| A_55_P2007339 | ens|ENSMUST00000105022|nap|NAP068828-1 | ENSMUST00000105022 | 2.84 |
| A_66_P136257 | ref|NM_172829|ens|ENSMUST00000086878|gb|BC125586|gb|BC138142 | St6gal2 | 2.84 |
| A_55_P2097121 | ens|ENSMUST00000117775|ens|ENSMUST00000121097|gb|BC094385 | ENSMUST00000117775 | 2.84 |
| A_55_P2084458 | ens|ENSMUST00000098707|gb|AK166451|tc|TC1760443|riken|G930023J15 | ENSMUST00000098707 | 2.83 |
| A_51_P162955 | ref|NM_177920|ens|ENSMUST00000033626|ens|ENSMUST00000060824|gb|BC132100 | Serpina7 | 2.83 |
| A_52_P599789 | ref|NM_001033267|ens|ENSMUST00000093909|ens|ENSMUST00000134182|gb|BC150976 | Qrich2 | 2.83 |
| A_55_P1969431 | ref|NM_001163628|ref|NM_133688|ens|ENSMUST00000111719|ens|ENSMUST00000111723 | Lyrm5 | 2.83 |
| A_55_P2074371 | ref|NM_145833|ens|ENSMUST00000051674|gb|AF521097|nap|NAP098592-001 | Lin28a | 2.82 |
| A_51_P153557 | ref|NM_175199|ens|ENSMUST00000066285|gb|AB093239|gb|AK141925 | Hspa12a | 2.82 |
| A_51_P188574 | ref|NM_001033535|gb|BC118003|gb|BC119769|gb|AK036953 | Tnfaip8l3 | 2.82 |
| A_51_P471911 | ens|ENSMUST00000028621|gb|AK014837|tc|TC1592958|riken|4921507L20 | ENSMUST00000028621 | 2.82 |
| A_55_P2277970 | ref|NM_001033668|ens|ENSMUST00000048603|gb|AK052631|gb|AK046346 | Dnahc1 | 2.82 |
| A_52_P495372 | ref|NM_153796|ens|ENSMUST00000026227|gb|AK146244|gb|AK054159 | Peo1 | 2.82 |
| A_55_P2349617 | gb|BG068075 | AU021880 | 2.82 |
| A_55_P2167406 | ens|ENSMUST00000041281|gb|AK167780|gb|BC080201|tc|TC1622125 | ENSMUST00000041281 | 2.82 |
| A_30_P01024696 | Unknown | chr17:10517466-10522172_F | 2.82 |
| A_55_P2420694 | gb|AK136555|riken|9130211K07 | AV074028 | 2.82 |
| A_52_P321140 | ref|NM_007843|ens|ENSMUST00000051017|gb|BC024380|gb|AK142544 | Defb1 | 2.82 |
| A_55_P1971448 | ref|NM_009226|ens|ENSMUST00000002551|ref|XM_001475517|ref|XM_001481165 | Snrpd1 | 2.81 |
| A_55_P2063033 | ens|ENSMUST00000106078|ens|ENSMUST00000133439|ens|ENSMUST00000147572|tc|TC1703408 | ENSMUST00000106078 | 2.81 |
| A_55_P1991451 | ref|XM_001478201 | Gm3845 | 2.81 |
| A_30_P01021602 | Unknown | chr15:60731651-60732079_R | 2.81 |
| A_55_P2107374 | ref|NM_025777|ens|ENSMUST00000028656|ens|ENSMUST00000099463|ref|XM_001480386 | Duoxa2 | 2.81 |
| A_55_P2052416 | ref|NM_001134383|ref|NM_001134384|ens|ENSMUST00000101151|ens|ENSMUST00000043863 | Iqsec1 | 2.80 |
| A_30_P01028595 | Unknown | chr18:90750100-90772025_F | 2.80 |
| A_51_P482322 | ref|NM_134235|ens|ENSMUST00000072044|gb|BC141439|tc|NP496230 | Vmn1r210 | 2.80 |
| A_52_P373666 | ref|NM_001081217|ens|ENSMUST00000041778|gb|AK038182|tc|TC1598461 | Zfp174 | 2.79 |
| A_55_P2000102 | ref|NM_025964|ens|ENSMUST00000114079|ens|ENSMUST00000053469|gb|AK033578 | Fam119a | 2.79 |
| A_55_P2413598 | ref|NM_001080813|ref|NM_029423|ens|ENSMUST00000054212|ens|ENSMUST00000033878 | Rab11fip1 | 2.79 |
| A_51_P436342 | ref|NM_008268|ens|ENSMUST00000049272|gb|M26283|gb|AK133512 | Hoxb5 | 2.79 |
| A_51_P202331 | ref|NM_011126|ens|ENSMUST00000028985|gb|U69172|gb|BC054375 | Plunc | 2.79 |
| A_30_P01033075 | Unknown | chr4:8351625-8358785_F | 2.79 |
| A_30_P01021484 | Unknown | lincP21 | 2.79 |
| A_55_P2184889 | ref|XM_001476948 | LOC100046866 | 2.79 |
| A_55_P1997305 | ref|XM_001478763|ref|XM_917116|nap|NAP063237-1 | Fam55a | 2.79 |
| A_55_P2028365 | ref|NM_001170788|ref|NM_001170789|ref|NM_001033371|ens|ENSMUST00000109355 | Lrrc36 | 2.79 |
| A_66_P116896 | ref|XM_001003948|ref|XM_001472902|gb|AK019818|gb|CN837231 | 4930578N18Rik | 2.79 |
| A_55_P2038183 | ref|NM_173767|ens|ENSMUST00000106620|ens|ENSMUST00000060351|ens|ENSMUST00000117543 | Insc | 2.78 |
| A_30_P01017464 | Unknown | chrX:20539200-20563428_F | 2.78 |
| A_52_P577748 | ref|NM_134152|ens|ENSMUST00000025601|gb|AK077085|gb|BC026563 | Lpxn | 2.78 |
| A_55_P2262743 | gb|CK332063|tc|TC1604837|nap|NAP024947-001 | Gm671 | 2.77 |
| A_55_P2180996 | ref|NM_182929|gb|BC115536|gb|AY326953|gb|AK043152 | Rims3 | 2.77 |
| A_55_P2101040 | ref|NM_028737|ens|ENSMUST00000013497|gb|AK030125|tc|TC1591832 | 4931406B18Rik | 2.77 |
| A_55_P1979103 | ref|NM_175528|ens|ENSMUST00000039008|gb|AK139744|gb|AK054277 | E330009J07Rik | 2.77 |
| A_55_P2130338 | ref|NM_146660|ens|ENSMUST00000099853|nap|NAP022286-001 | Olfr1135 | 2.77 |
| A_30_P01018734 | Unknown | chr6:86477309-86537554_R | 2.76 |
| A_51_P328631 | ref|NM_001081137|ens|ENSMUST00000094190|gb|AK008441|gb|EU937530 | Sis | 2.76 |
| A_55_P2128501 | ref|NM_031170|ens|ENSMUST00000023952|gb|M22831|gb|AK145777 | Krt8 | 2.76 |
| A_51_P450984 | ref|NM_146722|ens|ENSMUST00000060693|gb|BC127973|gb|DR065598 | Olfr429 | 2.76 |
| A_55_P1965432 | ref|XM_001477692|ref|XM_001475949 | LOC100047229 | 2.75 |
| A_55_P2080633 | ref|NM_175428|ref|NM_001081684|ref|NM_001081685|ens|ENSMUST00000113734 | Zfp295 | 2.75 |
| A_30_P01029986 | Unknown | chr1:94611439-94613779_R | 2.75 |
| A_30_P01029701 | Unknown | chr17:84895250-84908450_F | 2.75 |
| A_55_P2119263 | ref|XM_001472162 | Gm2019 | 2.75 |
| A_55_P2150442 | ref|XR_031823 | LOC675054 | 2.75 |
| A_30_P01029904 | Unknown | chr12:53669634-53672010_F | 2.75 |
| A_30_P01029915 | Unknown | chr13:98479793-98481177_R | 2.75 |
| A_55_P2051787 | ref|NM_015739|gb|AF547996|tc|TC1599006 | Gbx1 | 2.75 |
| A_55_P2002275 | ref|XM_622848|ref|XM_912024|gb|AY989873|gb|BC040514 | H2-T3-like | 2.75 |
| A_55_P2136612 | ref|XR_034610 | LOC637578 | 2.74 |
| A_55_P2156761 | ref|NM_029736|ens|ENSMUST00000093416|ens|ENSMUST00000034111|ens|ENSMUST00000109899 | Slc10a7 | 2.74 |
| A_55_P2005433 | ref|XM_001479248|ref|XM_001481094|tc|TC1725173|nap|NAP041609-1 | LOC100047570 | 2.74 |
| A_52_P16873 | ref|NM_178785|ens|ENSMUST00000137458|ens|ENSMUST00000135618|ens|ENSMUST00000136375 | Rasal3 | 2.74 |
| A_30_P01033015 | Unknown | chr1:108657255-108667405_F | 2.73 |
| A_30_P01023551 | Unknown | chr4:89088538-89130181_F | 2.73 |
| A_55_P2029915 | ref|NM_146346|ens|ENSMUST00000074250|ens|ENSMUST00000108465|gb|BC120574 | Olfr397 | 2.73 |
| A_52_P109304 | ref|NM_013763|gb|AK132366|gb|BC039273|gb|BC048185 | Tbl2 | 2.73 |
| A_66_P131896 | ref|NM_207155|ens|ENSMUST00000113639|ens|ENSMUST00000073636|gb|BC148220 | Olfr117 | 2.72 |
| A_55_P2048808 | ens|ENSMUST00000100090|gb|AK133126|tc|TC1594428|riken|4930588K23 | ENSMUST00000100090 | 2.72 |
| A_52_P163660 | ref|NM_025720|ens|ENSMUST00000092699|gb|AK003615|gb|AK014641 | Krtap3-2 | 2.72 |
| A_66_P117204 | ens|ENSMUST00000068507|gb|AK041383|gb|AK041468|tc|TC1588741 | ENSMUST00000068507 | 2.72 |
| A_52_P490071 | ref|NM_146550|ens|ENSMUST00000091986|gb|BC120749|gb|BC120751 | Olfr810 | 2.72 |
| A_30_P01028027 | Unknown | chr18:38776580-38841080_R | 2.72 |
| A_51_P229893 | ref|NM_021445|ens|ENSMUST00000021890|gb|AF223401|gb|AK005518 | Cts6 | 2.72 |
| A_55_P2041095 | ref|NM_011382|ens|ENSMUST00000043208|ens|ENSMUST00000110487|gb|D50416 | Six4 | 2.72 |
| A_55_P2078470 | ref|NM_001168369|ref|NM_029292|ens|ENSMUST00000148234|ens|ENSMUST00000026912 | 1700008F21Rik | 2.72 |
| A_55_P2164890 | ref|NM_029086|ens|ENSMUST00000151903|gb|AK153576|gb|AK044073 | 5830415F09Rik | 2.71 |
| A_55_P1988795 | ref|NM_019811|ens|ENSMUST00000065973|ens|ENSMUST00000109658|ens|ENSMUST00000133654 | Acss2 | 2.71 |
| A_52_P24631 | ref|NM_018745|ref|NM_001102458|ens|ENSMUST00000129589|ens|ENSMUST00000110329 | Azin1 | 2.71 |
| A_55_P2010758 | ref|NM_008723|ref|NR_002702|ens|ENSMUST00000070215|ref|XM_916150 | Npm3 | 2.71 |
| A_51_P140042 | ref|NM_029428|ens|ENSMUST00000098361|gb|AK015063|gb|BC137980 | Adad2 | 2.71 |
| A_52_P204035 | ref|XM_001480761|ref|XM_001473619|gb|AK016784|tc|TC1735769 | 4933412A08Rik | 2.71 |
| A_55_P1963639 | ref|NM_026716|ens|ENSMUST00000115981|ens|ENSMUST00000115982|ens|ENSMUST00000119059 | Sycn | 2.70 |
| A_55_P2404823 | gb|AK007074|tc|TC1694534|riken|1700095A21|nap|NAP120456-1 | 1700095A21Rik | 2.70 |
| A_52_P579517 | ref|NM_178379|ens|ENSMUST00000049091|gb|AK156865|gb|AK172718 | Cox10 | 2.70 |
| A_55_P2028680 | ref|NM_029367|ens|ENSMUST00000103223|ens|ENSMUST00000065468|ens|ENSMUST00000103222 | Spaca3 | 2.70 |
| A_52_P662244 | ref|NM_001012310|ens|ENSMUST00000057026|gb|AK149369|gb|BC042739 | AI132487 | 2.70 |
| A_55_P1980796 | ref|NM_008367|ens|ENSMUST00000028111|gb|AK088594|gb|AF054581 | Il2ra | 2.70 |
| A_55_P1987827 | ref|XM_889589|ref|XM_909359|nap|NAP060691-1 | Gm6545 | 2.70 |
| A_51_P145948 | ref|NM_144946|gb|AK081325|gb|AF448840|gb|BC051145 | Neto1 | 2.70 |
| A_55_P2170409 | ref|XM_914584 | LOC638531 | 2.70 |
| A_55_P1995512 | ref|NM_175408|ens|ENSMUST00000095987|gb|AK053212|gb|BC096053 | Tmem139 | 2.70 |
| A_55_P2019776 | ref|NM_001033275|ens|ENSMUST00000057896|ens|ENSMUST00000049484|ref|XR_031318 | Gxylt1 | 2.70 |
| A_52_P505827 | ref|NM_024195|ens|ENSMUST00000113151|ens|ENSMUST00000050631|ref|XR_001999 | Cyb5r4 | 2.70 |
| A_51_P353735 | ref|NM_019468|ens|ENSMUST00000119338|ens|ENSMUST00000143521|gb|BC120827 | G6pd2 | 2.70 |
| A_55_P2138627 | ens|ENSMUST00000103486|ref|XM_984967|ref|XR_031667|ref|XR_034099 | LOC675759 | 2.70 |
| A_51_P497870 | ref|NM_026489|ens|ENSMUST00000107154|ens|ENSMUST00000090797|gb|AK163995 | Hormad1 | 2.69 |
| A_52_P333648 | ref|NM_027033|ref|NM_145692|ens|ENSMUST00000130102|ens|ENSMUST00000124874 | Lrrc67 | 2.69 |
| A_52_P956363 | ens|ENSMUST00000067077|gb|AK085249|tc|TC1589430|tc|TC1639890 | ENSMUST00000067077 | 2.69 |
| A_51_P372393 | ref|NM_009641|ens|ENSMUST00000028955|gb|AF113707|gb|BC129965 | Angpt4 | 2.68 |
| A_55_P2004184 | tc|TC1629580 | TC1629580 | 2.68 |
| A_55_P2100355 | ref|NM_011326|ens|ENSMUST00000000221|gb|AF112187|gb|AK143995 | Scnn1g | 2.68 |
| A_51_P391495 | ref|NM_008893|ref|NM_001164057|ens|ENSMUST00000025752|gb|AK153081 | Pola2 | 2.68 |
| A_55_P2141796 | ref|XM_893895|ref|XM_912438 | Serpine3 | 2.68 |
| A_55_P2427150 | gb|AK049386|tc|TC1624397|riken|C430002N11|nap|NAP077045-1 | C430002N11Rik | 2.68 |
| A_55_P2158227 | ref|NM_009640|ens|ENSMUST00000110296|ens|ENSMUST00000022921|gb|AK088439 | Angpt1 | 2.68 |
| A_52_P412574 | ref|NM_183131|ens|ENSMUST00000061695|gb|AK019625|gb|BC048622 | 4930451I11Rik | 2.67 |
| A_51_P454008 | ref|NM_008489|ens|ENSMUST00000016168|gb|X99347|gb|BC004795 | Lbp | 2.67 |
| A_55_P2293146 | gb|AK016046|tc|TC1603612|riken|4930545H06|nap|NAP074465-1 | 4930545H06Rik | 2.67 |
| A_55_P2360800 | gb|AK048775|tc|TC1685458|riken|C230060E24|nap|NAP084759-1 | C230060E24 | 2.67 |
| A_30_P01031910 | Unknown | chr12:89479525-89503525_F | 2.67 |
| A_30_P01024860 | Unknown | chr6:82585089-82585332_R | 2.66 |
| A_51_P157524 | ref|NM_148952|ens|ENSMUST00000015003|gb|AK157028|gb|AK008398 | E2f4 | 2.66 |
| A_30_P01017973 | Unknown | chr4:124091663-124128538_R | 2.66 |
| A_52_P466799 | ref|NM_011884|ens|ENSMUST00000029942|ens|ENSMUST00000108153|gb|AK160706 | Rngtt | 2.65 |
| A_55_P1978735 | ref|NM_028834|ens|ENSMUST00000110094|gb|AK008687|riken|2210009G21 | 2210009G21Rik | 2.65 |
| A_30_P01027831 | Unknown | chr12:89448550-89461843_R | 2.65 |
| A_55_P1973156 | ens|ENSMUST00000099317|gb|AK157102|riken|F830201J05 | ENSMUST00000099317 | 2.65 |
| A_66_P126254 | ref|NM_013527|ens|ENSMUST00000037313|gb|AF525752|gb|BC156128 | Gdf7 | 2.65 |
| A_55_P2073592 | ref|NM_026278|ens|ENSMUST00000066451|ens|ENSMUST00000110380|ens|ENSMUST00000110381 | Lrp2bp | 2.65 |
| A_52_P631356 | ref|NM_177025|ref|NM_027225|ens|ENSMUST00000090894|ens|ENSMUST00000155082 | Cobll1 | 2.65 |
| A_30_P01028894 | Unknown | chr4:146233300-146260825_R | 2.65 |
| A_52_P113700 | ref|NM_027324|gb|AK076205|gb|AF325260|tc|TC1572564 | Sfxn1 | 2.65 |
| A_55_P1975718 | ref|NM_001142775|ens|ENSMUST00000106568|gb|AK132639|gb|EU099306 | Skint7 | 2.64 |
| A_55_P1988985 | ref|NM_001166712|ref|NM_001166737|ref|NM_001166713|ref|NM_001166714 | Vmn1r151 | 2.64 |
| A_55_P2146590 | ref|NM_026931|ens|ENSMUST00000052622|gb|AK007441|gb|BC016562 | 1810011O10Rik | 2.64 |
| A_30_P01029038 | Unknown | chrX:120324027-120326909_R | 2.64 |
| A_52_P432570 | ref|NM_175343|ref|NM_001136240|ref|NM_172264|ens|ENSMUST00000118917 | Chdh | 2.63 |
| A_30_P01028950 | Unknown | chr12:111322030-111323387_F | 2.63 |
| A_30_P01033366 | Unknown | chr16:32388843-32395264_R | 2.63 |
| A_55_P2041803 | ens|ENSMUST00000070887|tc|TC1699976 | ENSMUST00000070887 | 2.63 |
| A_51_P176042 | ref|NM_013631|ref|NM_001099779|ens|ENSMUST00000047111|gb|BC152542 | Pklr | 2.63 |
| A_55_P2180091 | ref|NM_027413|ens|ENSMUST00000032775|gb|AK008713|gb|AK050154 | Lrrc28 | 2.62 |
| A_55_P2394624 | gb|AK015648|tc|TC1599637|riken|4930488N15|nap|NAP074337-1 | 4930488N15Rik | 2.62 |
| A_55_P2185618 | ref|NM_175470|ens|ENSMUST00000062028|ref|XM_001478540|ref|XM_001480906 | Gpr61 | 2.62 |
| A_52_P6130 | ref|NM_011105|ens|ENSMUST00000064370|gb|BC156299|gb|BC157024 | Pkdrej | 2.62 |
| A_30_P01019174 | Unknown | chr11:16834711-16851125_R | 2.62 |
| A_30_P01031429 | Unknown | chr5:52581905-52637135_F | 2.62 |
| A_52_P304947 | ref|NM_028131|ens|ENSMUST00000034205|gb|AK012097|gb|BC027119 | Cenpn | 2.62 |
| A_55_P2105200 | ref|NM_172405|ens|ENSMUST00000117364|ens|ENSMUST00000153302|ens|ENSMUST00000044535 | Fam175a | 2.62 |
| A_52_P150988 | ref|NM_029582|ens|ENSMUST00000115818|ens|ENSMUST00000155221|ens|ENSMUST00000147884 | Txndc11 | 2.62 |
| A_30_P01019940 | Unknown | chr14:27176059-27202735_F | 2.62 |
| A_30_P01026018 | Unknown | chr9:30805020-30810645_F | 2.62 |
| A_55_P2193661 | gb|AK081630|tc|TC1628596|riken|C130057D09|nap|NAP084231-1 | C130057D09Rik | 2.62 |
| A_55_P2102624 | ref|NM_001113401|ref|NM_001113405|ref|NM_134111|ens|ENSMUST00000023537 | Eaf2 | 2.62 |
| A_52_P434279 | ens|ENSMUST00000118707|ens|ENSMUST00000117093|gb|BC024834|gb|AK016205 | ENSMUST00000118707 | 2.62 |
| A_52_P23379 | ref|NM_144804|ens|ENSMUST00000028595|gb|AK162086|gb|AK162218 | Depdc7 | 2.62 |
| A_55_P2160712 | ens|ENSMUST00000051301|ref|XM_001475407|ref|XM_001475437|ref|XM_001475421 | ENSMUST00000051301 | 2.61 |
| A_51_P433789 | ref|NM_207531|ens|ENSMUST00000042101|gb|AK053177|gb|BC023499 | Agr3 | 2.61 |
| A_52_P498086 | ref|NM_011355|ens|ENSMUST00000002180|gb|X17463|gb|AK154945 | Sfpi1 | 2.61 |
| A_30_P01026834 | Unknown | chr11:55315017-55322092_R | 2.60 |
| A_55_P2136121 | ref|NM_145448|ens|ENSMUST00000110070|ens|ENSMUST00000110068|ens|ENSMUST00000110073 | 9030617O03Rik | 2.60 |
| A_55_P2020736 | ref|NM_144939|ens|ENSMUST00000113296|ens|ENSMUST00000024034|gb|AK077209 | Frs3 | 2.60 |
| A_51_P211765 | ref|NM_145495|ens|ENSMUST00000025818|gb|BC011277|tc|TC1577567 | Rin1 | 2.60 |
| A_51_P399614 | ref|NM_023322|ens|ENSMUST00000031632|gb|BC141244|gb|AK012630 | Zkscan14 | 2.60 |
| A_55_P2138386 | ref|NM_010558|ens|ENSMUST00000048605|gb|X04601|gb|X06270 | Il5 | 2.60 |
| A_55_P2293668 | gb|AI266897 | AI132709 | 2.60 |
| A_55_P2060672 | ref|NM_010452|ens|ENSMUST00000118397|ens|ENSMUST00000048882|ens|ENSMUST00000114434 | Hoxa3 | 2.58 |
| A_52_P223224 | ens|ENSMUST00000119025|gb|AK049355|gb|BB815111|tc|TC1592855 | ENSMUST00000119025 | 2.58 |
| A_52_P72162 | ens|ENSMUST00000099963|ens|ENSMUST00000132967|ref|XM_141020|ref|XM_913669 | ENSMUST00000099963 | 2.58 |
| A_66_P110111 | ref|XR_001556|gb|AK081008|riken|B930020H05|nap|NAP083544-1 | Gm5636 | 2.58 |
| A_52_P1036735 | ref|NM_001004158|ens|ENSMUST00000066558|gb|AK076969|gb|BC099536 | Gm5142 | 2.58 |
| A_66_P139675 | ens|ENSMUST00000103300|gb|M11858|gb|M26418|gb|D12900 | ENSMUST00000103300 | 2.58 |
| A_55_P2066173 | ens|ENSMUST00000103421|ens|ENSMUST00000103420|ens|ENSMUST00000103419|ens|ENSMUST00000103416 | ENSMUST00000103421 | 2.58 |
| A_55_P1998319 | ref|XR_031396 | LOC100045098 | 2.57 |
| A_55_P2185757 | ens|ENSMUST00000107873 | ENSMUST00000107873 | 2.57 |
| A_30_P01030765 | Unknown | chr8:67542714-67561694_R | 2.57 |
| A_55_P2111782 | ref|XM_001480354 | Gm13134 | 2.57 |
| A_55_P2091496 | ref|NM_139218|ens|ENSMUST00000049644|ens|ENSMUST00000123429|ref|XM_909724 | Dppa3 | 2.56 |
| A_52_P671722 | ref|NM_001142631|ens|ENSMUST00000124001|gb|AK077138|tc|TC1589715 | Spdya | 2.56 |
| A_55_P1953894 | ref|NM_030026|ens|ENSMUST00000022148|ens|ENSMUST00000091326|gb|AK143233 | Mccc2 | 2.56 |
| A_55_P2018254 | ens|ENSMUST00000094315 | ENSMUST00000094315 | 2.56 |
| A_55_P1972436 | ref|NM_178666|ens|ENSMUST00000105515|ens|ENSMUST00000056097|gb|AK088136 | Themis | 2.56 |
| A_55_P2140745 | ref|XM_135380 | LOC235882 | 2.56 |
| A_51_P137452 | ref|NM_013809|ens|ENSMUST00000040944|gb|AK014160|gb|L81171 | Cyp2g1 | 2.56 |
| A_52_P141628 | ref|NM_011397|ens|ENSMUST00000025212|gb|AK085499|gb|AK143959 | Slc23a1 | 2.56 |
| A_55_P2282904 | gb|AK015707|riken|4930505M18|nap|NAP025391-001 | 4930505M18Rik | 2.56 |
| A_55_P2052062 | ref|NM_010818|ens|ENSMUST00000023341|gb|AK148308|gb|BC051984 | Cd200 | 2.56 |
| A_30_P01032480 | Unknown | chrX:135882029-135892277_R | 2.56 |
| A_30_P01026628 | Unknown | chr2:154235540-154248551_R | 2.56 |
| A_55_P2004248 | ref|NM_133237|ens|ENSMUST00000096554|gb|AK156319|gb|AK200324 | Apcdd1 | 2.55 |
| A_55_P2185143 | ens|ENSMUST00000111181|ens|ENSMUST00000044972|ens|ENSMUST00000111182|gb|AK090036 | ENSMUST00000111181 | 2.55 |
| A_55_P2309100 | ref|NM_024291|ens|ENSMUST00000039390|gb|AK047076|gb|AJ293727 | Ky | 2.55 |
| A_55_P2108109 | ref|NM_010205|ref|NM_001166361|ref|NM_001166362|ref|NM_001166363 | Fgf8 | 2.55 |
| A_55_P2040689 | ref|NM_175098|ens|ENSMUST00000095933|ens|ENSMUST00000055559|ens|ENSMUST00000114491 | Ccdc126 | 2.55 |
| A_30_P01032330 | Unknown | chr10:60473030-60535340_F | 2.54 |
| A_66_P114540 | ens|ENSMUST00000063891|gb|AK079848|tc|TC1618761|riken|A430092C21 | ENSMUST00000063891 | 2.54 |
| A_55_P2080481 | ref|XM_978031|ref|XM_985197|nap|NAP058854-1 | Gm8214 | 2.54 |
| A_55_P1999232 | ref|NM_001037752|ens|ENSMUST00000081235|ens|ENSMUST00000109834|gb|AY591384 | Defb45 | 2.54 |
| A_55_P2039532 | ref|NM_011040|ens|ENSMUST00000102940|ens|ENSMUST00000028355|gb|AK050717 | Pax8 | 2.54 |
| A_55_P1965911 | ref|XM_919447|ref|XM_905579|tc|TC1589582 | LOC632209 | 2.54 |
| A_55_P2130885 | ref|NM_008552|ens|ENSMUST00000089015|ref|XM_001480461|ref|XM_001480458 | Mas1 | 2.54 |
| A_52_P68223 | ref|XM_001475660|ref|XM_001477324|gb|AK016866|riken|4933421O10 | 4933421O10Rik | 2.54 |
| A_30_P01024386 | Unknown | chr18:47254152-47254844_F | 2.54 |
| A_55_P1985446 | ref|XM_884524 | Mrgprb13 | 2.54 |
| A_30_P01027049 | Unknown | chr13:28760289-28977218_R | 2.54 |
| A_55_P2357342 | gb|AK005634|tc|TC1591746|riken|1700003F17|nap|NAP012180-001 | 1700003F17Rik | 2.54 |
| A_55_P2094272 | ref|XM_001479176|gb|AK133045|riken|4930560G13 | Gm9694 | 2.53 |
| A_30_P01032194 | Unknown | chr13:94201024-94201496_R | 2.53 |
| A_55_P2363652 | gb|AK019498|riken|4632415I09|nap|NAP121284-001 | AK019498 | 2.53 |
| A_55_P2073035 | ref|XM_983279|ref|XM_983444|gb|AK007033|tc|TC1719123 | 1700091H14Rik | 2.53 |
| A_30_P01023313 | Unknown | chr1:162965309-162970704_F | 2.53 |
| A_51_P160913 | ref|NM_008209|ens|ENSMUST00000027744|gb|AK137171|gb|AF010448 | Mr1 | 2.53 |
| A_55_P1992515 | ref|NM_146319|ens|ENSMUST00000079142|gb|BC132575|gb|BC145712 | Olfr727 | 2.53 |
| A_55_P1972872 | ref|NM_001005858|ens|ENSMUST00000099507|ens|ENSMUST00000076249|ref|XM_001000862 | I830012O16Rik | 2.53 |
| A_30_P01028884 | Unknown | chr11:87872568-87880978_R | 2.53 |
| A_52_P125636 | ref|NM_020597|ens|ENSMUST00000022464|ens|ENSMUST00000130397|gb|BC140208 | Msmb | 2.53 |
| A_55_P2049025 | ens|ENSMUST00000093546|ref|XM_487321|nap|NAP021113-001 | Gm5670 | 2.53 |
| A_51_P100327 | ref|NM_013683|ref|NM_001161730|ens|ENSMUST00000041633|gb|S66882 | Tap1 | 2.52 |
| A_66_P122828 | ref|NM_030047|ens|ENSMUST00000070216|gb|AK133249|gb|AY842249 | 4930458L03Rik | 2.52 |
| A_55_P2168271 | ens|ENSMUST00000099718|gb|AK135041|tc|TC1591153|riken|6430553K19 | ENSMUST00000099718 | 2.52 |
| A_30_P01020507 | Unknown | chr6:6499224-6499832_R | 2.52 |
| A_55_P2054445 | ref|NM_013489|ens|ENSMUST00000042302|ens|ENSMUST00000136479|ens|ENSMUST00000155802 | Cd84 | 2.52 |
| A_55_P2332401 | gb|BF143845 | Ss18 | 2.52 |
| A_55_P2041924 | ref|NM_029373|ens|ENSMUST00000031798|gb|AK006332|tc|TC1590343 | 1700025E21Rik | 2.52 |
| A_52_P404302 | ref|NM_172854|gb|AK220202|gb|BC137871|gb|AK035313 | Olfml2a | 2.52 |
| A_51_P306933 | ref|NM_019404|ens|ENSMUST00000033765|gb|AJ310753|tc|TC1590212 | Avpr2 | 2.51 |
| A_55_P2154228 | ref|NM_178113|ens|ENSMUST00000086198|ens|ENSMUST00000073127|gb|DQ340802 | Ncapd3 | 2.51 |
| A_55_P2127699 | ref|NM_011089|ref|NM_011093|ref|NM_008848|ens|ENSMUST00000071671 | Pira2 | 2.51 |
| A_55_P2046760 | ref|NM_009493|ref|NM_001105063|ref|NM_001105156|ref|NM_001105152 | Vmn2r42 | 2.51 |
| A_51_P478678 | ens|ENSMUST00000111720|gb|AK012888|tc|TC1719761|tc|TC1595920 | ENSMUST00000111720 | 2.51 |
| A_55_P2043612 | ref|NM_001033281|ens|ENSMUST00000091900|ens|ENSMUST00000154557|ens|ENSMUST00000115398 | Prdm6 | 2.51 |
| A_51_P232207 | ref|NM_008269|ens|ENSMUST00000000704|gb|X56461|gb|BC016893 | Hoxb6 | 2.51 |
| A_66_P122433 | ref|NM_029107|ens|ENSMUST00000111154|gb|AK029492|gb|AK133272 | 4930417G10Rik | 2.51 |
| A_66_P121924 | ref|XM_001475470|ref|XM_001476872|gb|AK039441|riken|A330043C09 | A330043C09Rik | 2.51 |
| A_55_P1996971 | ens|ENSMUST00000100395|gb|AK138658|riken|A330094G03 | ENSMUST00000100395 | 2.51 |
| A_30_P01024360 | Unknown | chr18:34557000-34567050_R | 2.51 |
| A_55_P2064457 | ref|NM_001038655|ref|NM_010319|ens|ENSMUST00000118465|ens|ENSMUST00000118233 | Gng7 | 2.51 |
| A_55_P1959056 | ref|NM_148932|ens|ENSMUST00000111171|ens|ENSMUST00000065638|gb|AK172982 | Pom121 | 2.51 |
| A_55_P1975360 | ens|ENSMUST00000105895|tc|TC1622389|nap|NAP107316-1 | ENSMUST00000105895 | 2.51 |
| A_51_P256747 | ref|NM_173029|ens|ENSMUST00000027852|ens|ENSMUST00000111439|ens|ENSMUST00000111440 | Adcy10 | 2.50 |
| A_55_P2035838 | ens|ENSMUST00000106841|tc|TC1624361 | ENSMUST00000106841 | 2.50 |
| A_55_P2163897 | ref|XM_001477254|ref|XM_001478958|nap|NAP058456-1 | Gm3088 | 2.50 |
| A_55_P2021735 | ref|XR_031843|ref|XR_034927|nap|NAP068857-1 | Tsga10ip | 2.49 |
| A_30_P01025084 | Unknown | chr3:146014132-146014545_R | 2.49 |
| A_55_P2112605 | ref|NM_001105184|nap|NAP029872-1|nap|NAP000001-079 | Vmn2r71 | 2.49 |
| A_55_P2088401 | ref|NM_010399|ref|NM_010397|ens|ENSMUST00000080015|ens|ENSMUST00000077960 | H2-T9 | 2.49 |
| A_52_P248343 | ref|NM_153789|ref|NM_181043|ens|ENSMUST00000038275|gb|AY434449 | Mylip | 2.49 |
| A_55_P1977345 | ref|NM_001011807|ens|ENSMUST00000078517|nap|NAP114044-1 | Olfr191 | 2.49 |
| A_55_P2021603 | ens|ENSMUST00000103608|ens|ENSMUST00000103635|ens|ENSMUST00000103669|ref|XM_001478402 | ENSMUST00000103608 | 2.49 |
| A_55_P2065475 | ref|NM_146851|ens|ENSMUST00000078447|tc|TC1600230|nap|NAP098334-001 | Olfr295 | 2.49 |
| A_51_P241995 | ref|NM_016919|ens|ENSMUST00000004201|gb|AF176645|tc|TC1583902 | Col5a3 | 2.49 |
| A_55_P2175857 | ens|ENSMUST00000100929|gb|AK136714|riken|9230028M13 | ENSMUST00000100929 | 2.49 |
| A_52_P413394 | ref|NM_011148|ens|ENSMUST00000031359|gb|AF023458|gb|AK010833 | Ppef2 | 2.48 |
| A_55_P2067301 | ref|NM_028984|ens|ENSMUST00000110028|ens|ENSMUST00000110027|ens|ENSMUST00000028910 | 8430406I07Rik | 2.48 |
| A_30_P01028032 | Unknown | chrX:122623597-122630826_F | 2.48 |
| A_52_P193611 | ref|NM_181422|ens|ENSMUST00000042026|gb|AK046772|gb|AF271381 | Pkd2l1 | 2.48 |
| A_55_P2319815 | gb|CO038890 | AW552889 | 2.48 |
| A_30_P01022731 | Unknown | chr8:60132300-60146830_R | 2.48 |
| A_55_P1978666 | ref|NM_176998|ref|NM_178765|ref|NM_001032727|ens|ENSMUST00000090057 | Sybu | 2.48 |
| A_30_P01030140 | Unknown | chr3:30099759-30133959_F | 2.48 |
| A_55_P2311855 | gb|EL606049 | Gm10509 | 2.48 |
| A_55_P2000062 | ref|NM_008390|ref|NM_001159396|ens|ENSMUST00000108920|ens|ENSMUST00000019043 | Irf1 | 2.48 |
| A_55_P1964648 | ref|NM_001037719|ref|NM_177584|ens|ENSMUST00000102802|ens|ENSMUST00000063654 | Btla | 2.47 |
| A_55_P2153740 | ref|XM_001471975|ref|XM_001472093|ref|XM_001479034|gb|AK085535 | Gm1981 | 2.47 |
| A_55_P2001290 | ref|NM_009454|ens|ENSMUST00000028398|ens|ENSMUST00000121433|gb|AK215206 | Ube2e3 | 2.47 |
| A_51_P205326 | ref|NM_177743|ens|ENSMUST00000098269|gb|BC113767|gb|AK134876 | Fam198a | 2.47 |
| A_66_P104815 | ref|NM_007899|ens|ENSMUST00000131376|ens|ENSMUST00000123143|ens|ENSMUST00000029753 | Ecm1 | 2.47 |
| A_30_P01023749 | Unknown | chr17:32044823-32045974_R | 2.46 |
| A_55_P2060198 | ens|ENSMUST00000064573|gb|AK082849|tc|TC1594206|riken|C330042E05 | ENSMUST00000064573 | 2.46 |
| A_52_P42069 | ref|NM_021381|ens|ENSMUST00000050887|gb|AK156734|gb|AK134279 | Prokr1 | 2.46 |
| A_51_P399265 | ref|NM_001168602|ens|ENSMUST00000085886|ref|XM_898397|gb|AJ245454 | Smok3a | 2.46 |
| A_55_P2082539 | ref|NM_134198|ens|ENSMUST00000079633|gb|BC127026|tc|NP496264 | Vmn1r234 | 2.46 |
| A_52_P410765 | ref|NM_011352|ens|ENSMUST00000043059|gb|AK156123|gb|AK019344 | Sema7a | 2.46 |
| A_66_P121312 | ref|NM_001011529|ens|ENSMUST00000099767|gb|BC147159|gb|BC147160 | Olfr1251 | 2.46 |
| A_30_P01032259 | Unknown | chr13:104591573-104599048_R | 2.45 |
| A_30_P01018831 | Unknown | chr4:3746573-3746891_F | 2.45 |
| A_55_P2362955 | gb|AK077069|tc|TC1616582|riken|4933400C23|nap|NAP089216-1 | 4933400C23 | 2.45 |
| A_30_P01025636 | Unknown | NRON | 2.44 |
| A_30_P01023733 | Unknown | chr5:120269342-120279542_F | 2.44 |
| A_30_P01029751 | Unknown | chr3:18521534-18522194_R | 2.44 |
| A_55_P2163857 | ref|NM_001146007|ref|NM_175677|ens|ENSMUST00000059037|gb|AK146437 | 9230105E10Rik | 2.44 |
| A_52_P20906 | ref|NM_011658|ens|ENSMUST00000049089|gb|M63650|gb|BC033434 | Twist1 | 2.44 |
| A_55_P1963434 | ref|NM_008150|ens|ENSMUST00000033450|ens|ENSMUST00000114860|ref|XR_033476 | Gpc4 | 2.44 |
| A_55_P2090652 | ref|XM_001480606|ref|XM_001480206 | LOC100048594 | 2.44 |
| A_51_P347467 | ref|NM_028276|ens|ENSMUST00000114979|ens|ENSMUST00000080713|gb|AK134348 | Utp14a | 2.44 |
| A_66_P122883 | ref|NM_008803|gb|BC125578|gb|BC132145 | Pde8a | 2.44 |
| A_55_P2218483 | gb|AK043413|tc|TC1625045|riken|A730093L10 | A730093L10Rik | 2.44 |
| A_55_P1963006 | Unknown | A_55_P1963006 | 2.44 |
| A_55_P2100655 | ref|NM_030145|ens|ENSMUST00000073284|ens|ENSMUST00000051867|ref|XM_001476756 | Lsm6 | 2.44 |
| A_52_P432881 | ref|NM_030556|ens|ENSMUST00000045560|gb|AK098112|gb|AK079566 | Slc19a3 | 2.43 |
| A_55_P2243566 | gb|AK036466|riken|9830113G23|nap|NAP117508-1 | 9430076G02Rik | 2.43 |
| A_55_P2184189 | ref|XR_031347|ref|XR_032778|gb|BC003730|gb|BC076609 | Ncf2-rs | 2.43 |
| A_30_P01028352 | Unknown | chr4:55465247-55494547_F | 2.43 |
| A_55_P2094064 | ref|XR_031919|ref|XR_031025 | LOC674844 | 2.43 |
| A_55_P2069989 | ref|NM_153128|ens|ENSMUST00000027725|ens|ENSMUST00000116528|ens|ENSMUST00000112232 | Klhl12 | 2.43 |
| A_52_P130686 | ref|NM_147101|ens|ENSMUST00000098227|gb|BC100691|gb|BC100692 | Olfr549 | 2.43 |
| A_55_P2121761 | ref|XM_896571|ref|XM_924215 | Gm6249 | 2.43 |
| A_66_P135702 | ref|NM_007941|ens|ENSMUST00000100680|ens|ENSMUST00000031378|ref|XM_916036 | Stx2 | 2.43 |
| A_55_P2006300 | ref|NM_010661|ens|ENSMUST00000017741|tc|TC1578522|nap|NAP009850-001 | Krt12 | 2.43 |
| A_51_P264634 | ref|NM_009261|gb|AK082675|tc|TC1604065|riken|C230082I21 | Strbp | 2.43 |
| A_55_P2109882 | ref|XM_001480479|ref|XM_001480475|ref|XM_001481202|ref|XM_001481201 | Gm4716 | 2.43 |
| A_55_P2070976 | ref|XM_893647|ref|XM_909790|nap|NAP021429-001 | Gm6923 | 2.42 |
| A_52_P628127 | ref|NM_001085491|ens|ENSMUST00000106043|gb|AK018907|gb|AK018987 | 1700125D06Rik | 2.42 |
| A_30_P01021088 | Unknown | chr13:26649529-26838004_R | 2.42 |
| A_55_P2037743 | ref|XM_001474868|ref|XM_001479326|gb|AK077204|gb|AV263240 | LOC100047072 | 2.42 |
| A_51_P161308 | ref|NM_013667|ens|ENSMUST00000046959|gb|BC069911|gb|BC015250 | Slc22a2 | 2.42 |
| A_51_P231557 | ref|XM_887943|ref|XM_910329|gb|AK033061|gb|AK080657 | 7530428D23Rik | 2.42 |
| A_51_P185693 | ref|NM_031197|ens|ENSMUST00000029240|gb|AK005068|gb|AK149532 | Slc2a2 | 2.42 |
| A_51_P190254 | ens|ENSMUST00000019268|gb|AK129084|gb|AK031998|tc|TC1600655 | Scrn1 | 2.42 |
| A_52_P483336 | ref|NM_007641|ens|ENSMUST00000025576|gb|AK137469|gb|BC028322 | Ms4a1 | 2.42 |
| A_30_P01020544 | Unknown | chr1:87412059-87419385_F | 2.42 |
| A_52_P109270 | ref|NM_173379|ens|ENSMUST00000039990|gb|AJ430350|tc|TC1585868 | Leprel1 | 2.41 |
| A_55_P2042277 | ref|NM_001081251|ens|ENSMUST00000112094|ens|ENSMUST00000022474|ens|ENSMUST00000112092 | Pbrm1 | 2.41 |
| A_30_P01028295 | Unknown | chr14:78957377-78963514_F | 2.40 |
| A_51_P286034 | ref|NM_134033|ens|ENSMUST00000020776|gb|BC018601|gb|BC034558 | Ccdc117 | 2.40 |
| A_52_P540488 | ref|NM_001012309|ens|ENSMUST00000102494|tc|TC1579286|nap|NAP034446-1 | Ccdc55 | 2.40 |
| A_51_P418820 | ref|NM_009335|ref|NM_001159696|ens|ENSMUST00000099058|ens|ENSMUST00000030391 | Tcfap2c | 2.40 |
| A_51_P385351 | ref|NM_145394|ens|ENSMUST00000039197|gb|AK180307|gb|BC025548 | Slc44a3 | 2.40 |
| A_55_P1974243 | ref|NM_138942 | Dbh | 2.40 |
| A_55_P2018017 | ref|NM_009425|ens|ENSMUST00000046383|gb|AK039433|gb|AK157633 | Tnfsf10 | 2.40 |
| A_55_P2062171 | ens|ENSMUST00000106049|ens|ENSMUST00000026555|gb|AK005883|gb|AB067773 | Odf3 | 2.40 |
| A_55_P2062004 | tc|TC1773384 | TC1773384 | 2.40 |
| A_55_P2413458 | ref|NM_023755|ens|ENSMUST00000027629|gb|AK143960|gb|BC116663 | Tcfcp2l1 | 2.39 |
| A_51_P107722 | ens|ENSMUST00000061643|gb|AK011970|tc|TC1648870|riken|2610303G11 | ENSMUST00000061643 | 2.39 |
| A_55_P1967168 | ref|NM_172668|ens|ENSMUST00000111341|ens|ENSMUST00000028689|gb|BC132240 | Lrp4 | 2.39 |
| A_55_P2065424 | ref|NM_009217|ref|NM_001042606|ens|ENSMUST00000067591|ens|ENSMUST00000106630 | Sstr2 | 2.39 |
| A_30_P01030772 | Unknown | chr17:38132217-38157667_R | 2.39 |
| A_30_P01027687 | Unknown | chr6:40699474-40719122_F | 2.39 |
| A_30_P01022983 | Unknown | chr1:138518674-138521227_R | 2.39 |
| A_52_P420357 | ref|NM_053079|ens|ENSMUST00000088386|gb|AK136428|gb|AF205540 | Slc15a1 | 2.39 |
| A_55_P1997479 | ref|NM_173770|ens|ENSMUST00000052501|gb|AK046504|tc|TC1592437 | Fam69c | 2.39 |
| A_30_P01025011 | Unknown | chr6:117827499-117851478_F | 2.38 |
| A_51_P101460 | ref|NM_023842|ens|ENSMUST00000068162|ens|ENSMUST00000124830|ens|ENSMUST00000127906 | Dsp | 2.38 |
| A_52_P486234 | ens|ENSMUST00000108216|gb|AK033497|gb|AK031520|gb|BB040069 | ENSMUST00000108216 | 2.38 |
| A_30_P01023806 | Unknown | chr6:23105525-23118900_F | 2.38 |
| A_51_P176265 | gb|BC025876|tc|TC1603459 | Trip11 | 2.37 |
| A_55_P2123207 | ens|ENSMUST00000064334|ens|ENSMUST00000150042|gb|AK085283|gb|BC090640 | ENSMUST00000064334 | 2.37 |
| A_52_P15300 | ref|NM_134032|ens|ENSMUST00000100523|gb|BC019414|tc|TC1594062 | Hoxb2 | 2.37 |
| A_55_P2178568 | ref|NM_133715|ens|ENSMUST00000041385|ens|ENSMUST00000107024|gb|AK046763 | Arhgap27 | 2.37 |
| A_52_P104562 | ref|NM_001037918|gb|BC019519 | Lipt1 | 2.37 |
| A_52_P169082 | ref|NM_021294|ens|ENSMUST00000040806|gb|AK006528|gb|S83465 | Dbil5 | 2.36 |
| A_55_P2174863 | ens|ENSMUST00000091994|ref|XM_001474481|ref|XM_621234|nap|NAP108747-1 | ENSMUST00000091994 | 2.36 |
| A_55_P2104382 | ref|XM_001473716|ref|XM_001480235 | Gm4456 | 2.36 |
| A_55_P1987740 | ref|NM_018751|ens|ENSMUST00000024738|ens|ENSMUST00000110788|ref|XM_001471993 | Sult1c1 | 2.36 |
| A_55_P2078320 | ref|XM_001473306|ref|XM_001473616 | LOC100044943 | 2.36 |
| A_55_P2002287 | ref|NM_010836|ens|ENSMUST00000106084|ens|ENSMUST00000055353|gb|AB221640 | Msx3 | 2.36 |
| A_52_P747383 | ens|ENSMUST00000068927|gb|AK039675|tc|TC1608353|riken|A330084C13 | ENSMUST00000068927 | 2.36 |
| A_55_P1959615 | ens|ENSMUST00000098770|gb|AK149124|riken|7120485K02 | ENSMUST00000098770 | 2.36 |
| A_55_P2066017 | ref|XM_001478769|ref|XM_001476337 | Gm4015 | 2.36 |
| A_30_P01027593 | Unknown | chr7:69075022-69127242_R | 2.36 |
| A_30_P01025499 | Unknown | chr3:84610558-84611133_R | 2.36 |
| A_55_P2157530 | ref|XM_001479304|ref|XM_001479970 | Gm4360 | 2.36 |
| A_30_P01019862 | Unknown | chr7:68849401-68958266_R | 2.36 |
| A_55_P2019620 | ref|NM_001104543|ref|NM_001102581|ref|NM_001104549|ref|NM_001104550 | Vmn2r94 | 2.36 |
| A_66_P109978 | ens|ENSMUST00000117450|gb|AK014659|gb|AV248510|riken|4833407C03 | ENSMUST00000117450 | 2.35 |
| A_55_P2002553 | ref|NM_172675|ref|NM_001102423|ref|NM_001102424|ref|NM_001102425 | Stx16 | 2.35 |
| A_55_P2164492 | ens|ENSMUST00000099138|gb|AK152815|gb|AK150964|riken|I830086L09 | ENSMUST00000099138 | 2.35 |
| A_55_P1992199 | ref|NM_054058|ens|ENSMUST00000076677|tc|NP062293|nap|NAP102704-1 | Psg20 | 2.35 |
| A_55_P2042247 | ref|NM_177690|ens|ENSMUST00000045022|gb|DQ066881|gb|AK054264 | Nlrp2 | 2.35 |
| A_55_P2017804 | ref|NM_009942|ens|ENSMUST00000081180|ref|XM_001475417|gb|AK135174 | Cox5b | 2.35 |
| A_51_P405375 | ref|NM_175358|ens|ENSMUST00000042070|gb|AK029137|gb|AK132304 | Zdhhc15 | 2.35 |
| A_30_P01027765 | Unknown | chr18:38477934-38478445_F | 2.35 |
| A_55_P2150612 | ref|NM_080453|ref|NM_172797|ens|ENSMUST00000119346|ens|ENSMUST00000100710 | Mmp28 | 2.35 |
| A_51_P114722 | ref|NM_019545|ens|ENSMUST00000029464|gb|AF272947|gb|AK018684 | Hao2 | 2.35 |
| A_55_P2087399 | ref|NM_029381|ens|ENSMUST00000109729|ens|ENSMUST00000146107|ens|ENSMUST00000012355 | Tex22 | 2.35 |
| A_55_P1997434 | ref|NM_001177418|ref|NM_001177422|ref|NM_001177424|ref|NM_001177429 | Gm13698 | 2.35 |
| A_51_P190979 | ref|NM_146650|ens|ENSMUST00000099833|gb|BC051250|tc|TC1599773 | Olfr1166 | 2.35 |
| A_30_P01032953 | Unknown | chr10:24269317-24270145_R | 2.35 |
| A_55_P2120737 | ens|ENSMUST00000097572|gb|AK133141|riken|4930598K17 | ENSMUST00000097572 | 2.35 |
| A_55_P2002190 | ref|XM_001476196 | Gm3280 | 2.35 |
| A_30_P01030429 | Unknown | chr14:67585234-67596823_F | 2.35 |
| A_55_P1995205 | ref|NM_011623|ens|ENSMUST00000068031|gb|D12513|gb|AK033321 | Top2a | 2.34 |
| A_55_P2041363 | ref|XM_001474880|tc|TC1623546 | LOC100045760 | 2.34 |
| A_55_P2124472 | ref|XM_001472941 | Gm2254 | 2.34 |
| A_55_P2120308 | ref|NM_007578|ens|ENSMUST00000121390|ens|ENSMUST00000038122|gb|AK147288 | Cacna1a | 2.34 |
| A_55_P2409336 | gb|AK016536|tc|TC1656003|riken|4932429P19|nap|NAP023741-001 | 4932429P19Rik | 2.34 |
| A_55_P2154119 | ref|XM_001474348|ref|XM_001476031 | Gm3228 | 2.34 |
| A_55_P1985925 | ens|ENSMUST00000098987|gb|AK142629|riken|D730019K09 | ENSMUST00000098987 | 2.34 |
| A_30_P01031981 | Unknown | chr9:78372153-78372834_F | 2.34 |
| A_55_P1957178 | ref|XM_001477863|ref|XM_001474010|gb|AK016056|riken|4930547H16 | 4930547H16Rik | 2.34 |
| A_55_P2330215 | gb|AK085325|tc|TC1599717|riken|D630011P15|nap|NAP118828-1 | A730090N16Rik | 2.34 |
| A_55_P2131213 | ref|NM_001085506|ens|ENSMUST00000088481|gb|AK136064|tc|TC1680248 | Magea10 | 2.34 |
| A_55_P2077628 | ens|ENSMUST00000106148|gb|AK180254|tc|NP1282615|tc|TC1602529 | ENSMUST00000106148 | 2.34 |
| A_51_P161054 | ref|NM_021359|ref|NM_001159564|ens|ENSMUST00000028348|ens|ENSMUST00000059888 | Itgb6 | 2.34 |
| A_55_P2052048 | ref|NM_028300|ens|ENSMUST00000151197|ens|ENSMUST00000000171|ens|ENSMUST00000132187 | Pih1d2 | 2.34 |
| A_55_P2063376 | ref|NM_013458|ens|ENSMUST00000032068|ens|ENSMUST00000032069|tc|TC1584489 | Add2 | 2.33 |
| A_30_P01026592 | Unknown | chr2:67834535-67834914_F | 2.33 |
| A_30_P01031410 | Unknown | chr12:33808493-33829143_F | 2.33 |
| A_55_P2086770 | ref|NM_001040695|tc|TC1667482 | Uevld | 2.33 |
| A_55_P1973120 | ref|NR_033566|ens|ENSMUST00000097812|gb|AK131719|gb|AV042767 | AI507597 | 2.33 |
| A_55_P2127223 | ens|ENSMUST00000056170|gb|AK076716|tc|TC1593373|riken|4930420A19 | ENSMUST00000056170 | 2.33 |
| A_55_P1988048 | ref|NM_001163522|ref|NM_016885|ens|ENSMUST00000119475|ens|ENSMUST00000067877 | Emcn | 2.33 |
| A_66_P109220 | ens|ENSMUST00000111548|ens|ENSMUST00000111547|gb|AK077292|gb|AK160463 | ENSMUST00000111548 | 2.33 |
| A_55_P2292470 | gb|AK016796|riken|4933412O06|nap|NAP073473-1 | 4933412O06Rik | 2.33 |
| A_51_P282616 | ref|NM_001081025|ens|ENSMUST00000023501|gb|AK077038|tc|TC1603418 | 4932425I24Rik | 2.33 |
| A_55_P2370394 | ref|NM_012024|ens|ENSMUST00000021447|ens|ENSMUST00000101303|gb|AK133824 | Ppp2r5e | 2.32 |
| A_30_P01024278 | Unknown | chr12:32781477-32808567_R | 2.32 |
| A_55_P2077535 | ref|NM_175311|ref|NM_001177901|ens|ENSMUST00000087992|ens|ENSMUST00000114590 | Zfp513 | 2.32 |
| A_55_P2150851 | ens|ENSMUST00000044682|ens|ENSMUST00000064236|gb|AK081075|tc|TC1593053 | ENSMUST00000044682 | 2.32 |
| A_30_P01018619 | Unknown | chr13:67806242-67830110_R | 2.32 |
| A_55_P2333797 | gb|AK015575|riken|4930474H20|nap|NAP074284-1 | 4930474H20Rik | 2.32 |
| A_30_P01027021 | Unknown | chr5:104936417-104984892_F | 2.31 |
| A_66_P114501 | ref|NM_026025|ens|ENSMUST00000076070|gb|AK170136|gb|AK012852 | Zcrb1 | 2.31 |
| A_30_P01028478 | Unknown | chr12:76771424-76772158_F | 2.31 |
| A_51_P226542 | ref|NM_173450|ens|ENSMUST00000028796|gb|AK044767|gb|BB488470 | Rpusd2 | 2.31 |
| A_51_P219483 | ref|NM_001024619|ref|NM_001168541|ref|NM_001168540|ref|NM_001168539 | Tsku | 2.31 |
| A_51_P403814 | ref|NM_001166067|ens|ENSMUST00000039212|ens|ENSMUST00000113899|gb|AK049248 | Slc4a5 | 2.31 |
| A_55_P1989787 | ens|ENSMUST00000053859|nap|NAP059701-1 | ENSMUST00000053859 | 2.31 |
| A_55_P2154659 | ref|NM_023651|ens|ENSMUST00000020523|gb|AK031710|tc|TC1671980 | Pex13 | 2.31 |
| A_55_P2007372 | ref|XR_032342|ref|XR_033710|tc|TC1630516 | Gm15550 | 2.31 |
| A_52_P439887 | ens|ENSMUST00000053799 | ENSMUST00000053799 | 2.31 |
| A_55_P1976142 | ens|ENSMUST00000098321 | ENSMUST00000098321 | 2.30 |
| A_55_P2247021 | ref|NM_029317|ens|ENSMUST00000085422|ens|ENSMUST00000107897|gb|AK006268 | 1700023D19Rik | 2.30 |
| A_55_P2155372 | ref|NM_001081284|ens|ENSMUST00000107071|nap|NAP069072-1 | Gm12695 | 2.30 |
| A_30_P01026665 | Unknown | chr13:23519843-23522887_R | 2.30 |
| A_55_P2054455 | ref|NM_025510|ens|ENSMUST00000072719|ens|ENSMUST00000116364|gb|AK009142 | 2310004I24Rik | 2.30 |
| A_30_P01019432 | Unknown | chr2:67410825-67411407_F | 2.30 |
| A_30_P01022714 | Unknown | chr9:35112668-35113346_F | 2.30 |
| A_66_P129671 | ens|ENSMUST00000094956|gb|AK154974|gb|AK089333|tc|TC1590210 | ENSMUST00000094956 | 2.30 |
| A_55_P2219174 | gb|AK008222|tc|TC1609250|riken|2010013B24|nap|NAP090868-1 | 2010013B24Rik | 2.30 |
| A_52_P561772 | ens|ENSMUST00000047643|nap|NAP013487-001 | ENSMUST00000047643 | 2.30 |
| A_55_P2033308 | ref|NM_011380|ens|ENSMUST00000024947|gb|D83147|gb|BC068021 | Six2 | 2.30 |
| A_30_P01027682 | Unknown | chr16:21305700-21306107_F | 2.30 |
| A_55_P2078116 | ens|ENSMUST00000056178 | ENSMUST00000056178 | 2.29 |
| A_55_P2104835 | ens|ENSMUST00000077457|ens|ENSMUST00000031429|gb|AK162820|riken|A230004D21 | ENSMUST00000077457 | 2.29 |
| A_55_P1976849 | ref|NR_015593|gb|AK051169|gb|AK051771|gb|AK051495 | D130009I18Rik | 2.29 |
| A_30_P01025209 | Unknown | chr16:31607863-31608726_F | 2.29 |
| A_30_P01023611 | Unknown | chr9:121845920-121858120_R | 2.29 |
| A_30_P01028758 | Unknown | chr3:121627545-121639470_F | 2.29 |
| A_52_P273847 | ref|NM_029061|ens|ENSMUST00000108747|ens|ENSMUST00000108748|ens|ENSMUST00000095158 | Ccdc7 | 2.28 |
| A_55_P2081942 | ref|XM_001478032 | LOC100047273 | 2.28 |
| A_66_P109708 | ref|NM_019450|ens|ENSMUST00000028361|gb|AK224931|gb|AK224929 | Il1f6 | 2.28 |
| A_52_P681016 | ens|ENSMUST00000099348|gb|AY344585|tc|NP835894 | ENSMUST00000099348 | 2.28 |
| A_55_P2020026 | ref|XM_001479264|ref|XM_001472276 | 4930439D14Rik | 2.28 |
| A_52_P32864 | ref|NM_026662|ens|ENSMUST00000026839|gb|BC024942|gb|AK029690 | Prps2 | 2.28 |
| A_51_P104569 | ref|NM_146505|ens|ENSMUST00000050807|gb|BC125353|gb|BC132239 | Olfr148 | 2.28 |
| A_55_P1994132 | ens|ENSMUST00000110832|gb|AK148454|tc|TC1618977|riken|C820004G13 | Tmem184a | 2.28 |
| A_30_P01031765 | Unknown | chr17:17143325-17181500_R | 2.28 |
| A_55_P2215680 | gb|AK133241|riken|4932420L07 | Gm15612 | 2.28 |
| A_51_P369675 | ref|NM_146841|ens|ENSMUST00000048265|ens|ENSMUST00000106884|ens|ENSMUST00000106885 | Olfr617 | 2.28 |
| A_51_P169327 | ref|NM_175147|ens|ENSMUST00000074232|gb|AK006555|gb|AY134667 | Etd | 2.28 |
| A_55_P2067538 | ref|NM_133193|ens|ENSMUST00000095020|gb|BC117797|gb|AF284433 | Il1rl2 | 2.27 |
| A_30_P01029324 | Unknown | chr19:53517903-53540753_R | 2.27 |
| A_55_P2029721 | ref|XR_032962|gb|AK016831|tc|TC1619507|riken|4933416E14 | 4933416E14Rik | 2.27 |
| A_66_P120205 | ens|ENSMUST00000084298|gb|AK082664|gb|AK137318|tc|TC1587526 | ENSMUST00000084298 | 2.27 |
| A_55_P2095311 | ens|ENSMUST00000023246|ref|XM_001475753|ref|XM_909927|tc|NP063235 | ENSMUST00000023246 | 2.27 |
| A_55_P1975535 | ref|NM_026532|ref|NR_033574|ens|ENSMUST00000008594|ref|XM_001003873 | Nutf2 | 2.27 |
| A_55_P2008556 | ref|XM_001477085|ref|XM_981806|ref|XM_001477829|ref|XM_001477405 | LOC100041834 | 2.27 |
| A_55_P2345825 | gb|AK005964|tc|TC1607537|riken|1700013M08|nap|NAP090572-1 | 1700013M08Rik | 2.27 |
| A_55_P2045446 | ref|NM_001045518|ens|ENSMUST00000098546|gb|BC120577|gb|BC120579 | Fam83b | 2.27 |
| A_55_P2278775 | gb|AK050221|riken|C730027O05|nap|NAP025675-001 | 9130016M20Rik | 2.27 |
| A_51_P466685 | ref|NM_175206|gb|AK052175|gb|AK084845|tc|TC1654975 | Fbxl22 | 2.27 |
| A_52_P615375 | ref|NM_178218|ens|ENSMUST00000108817|gb|AK077568|gb|AK087537 | Hist3h2a | 2.26 |
| A_30_P01024008 | Unknown | chr1:167802183-167805411_F | 2.26 |
| A_52_P506452 | ens|ENSMUST00000052843|gb|AK031395|tc|TC1594760|riken|6030422M02 | ENSMUST00000052843 | 2.26 |
| A_55_P2043882 | ref|NM_012035|ens|ENSMUST00000109871|ens|ENSMUST00000022023|gb|AK135153 | Trpc7 | 2.26 |
| A_55_P2009116 | ref|NR_033558|ens|ENSMUST00000099888|gb|AK158018|riken|F930017D23 | F830002L21Rik | 2.26 |
| A_51_P116651 | ref|NM_019759|ens|ENSMUST00000027861|gb|AF143374|gb|BC046420 | Dpt | 2.26 |
| A_51_P126563 | ref|NM_008769|ens|ENSMUST00000049910|gb|M17030|gb|AK050930 | Otc | 2.26 |
| A_51_P448203 | ref|NM_013493|ref|NM_001109745|ref|NM_001109746|ens|ENSMUST00000032138 | Cnbp | 2.26 |
| A_30_P01033125 | Unknown | chr8:67550064-67560987_R | 2.26 |
| A_51_P488991 | ref|NM_178644|ens|ENSMUST00000034512|gb|AK150279|gb|BC034836 | Oaf | 2.26 |
| A_55_P2136249 | ens|ENSMUST00000108547|nap|NAP061506-1 | ENSMUST00000108547 | 2.26 |
| A_55_P1988703 | ref|NM_146896|ens|ENSMUST00000076438|nap|NAP006888-002 | Olfr1203 | 2.26 |
| A_55_P2119377 | ref|NM_177155|ens|ENSMUST00000050385|gb|AK040915|riken|A530044P19 | Klri2 | 2.26 |
| A_30_P01029466 | Unknown | chrX:121493684-121500532_F | 2.26 |
| A_55_P2083624 | ref|NM_016852|ens|ENSMUST00000106446|ens|ENSMUST00000074628|gb|AK170474 | Wbp2 | 2.26 |
| A_52_P258338 | ref|NM_178061|gb|AK039841|gb|BC020028|gb|AK155217 | Mobkl2b | 2.25 |
| A_55_P2370931 | gb|CK334688|nap|NAP049195-1 | AU017674 | 2.25 |
| A_51_P123262 | ref|NM_029530|ens|ENSMUST00000057503|gb|AK014127|gb|AK018222 | 6330527O06Rik | 2.25 |
| A_55_P2075909 | ref|NM_001101579|nap|NAP062304-1 | Vmn1r12 | 2.25 |
| A_55_P2363040 | gb|AK005727|riken|1700007J24 | 1700007J24Rik | 2.25 |
| A_55_P2064272 | ref|XM_001478621 | Gm9545 | 2.25 |
| A_55_P2325663 | ref|NM_001164201|gb|AK160253|tc|TC1682009|riken|4930550L11 | Lass3 | 2.25 |
| A_51_P463003 | ref|NM_021367|ref|NR_033206|ens|ENSMUST00000025237|gb|AF232937 | Tslp | 2.25 |
| A_55_P2022946 | ref|NM_013627|ens|ENSMUST00000111086|ens|ENSMUST00000111084|ens|ENSMUST00000123063 | Pax6 | 2.25 |
| A_55_P2383897 | gb|AK047163|tc|TC1607868|riken|B930030E13|nap|NAP083567-1 | 8030453O22Rik | 2.25 |
| A_30_P01030283 | Unknown | chr1:153685487-153710962_R | 2.25 |
| A_55_P2285397 | gb|AK016003|riken|4930539N22|nap|NAP019234-001 | 4930539N22Rik | 2.25 |
| A_55_P1960857 | ref|NM_172752|ens|ENSMUST00000098788|ens|ENSMUST00000130011|ens|ENSMUST00000135336 | Sorbs2 | 2.24 |
| A_55_P1974892 | ref|NM_013915|ref|NM_001012330|ens|ENSMUST00000094276|ens|ENSMUST00000077225 | Zfp238 | 2.24 |
| A_55_P2079475 | ref|XM_001475610 | LOC100046119 | 2.24 |
| A_55_P1964585 | ref|XR_032445|ref|XR_033132|ref|XR_031874|ref|XR_032834 | LOC100041111 | 2.24 |
| A_55_P2071283 | ref|XM_001474514|ref|XM_001477208 | Gm3374 | 2.24 |
| A_66_P126640 | ref|NM_007592|ens|ENSMUST00000098290|ref|XM_001473032|gb|AK079310 | Car8 | 2.24 |
| A_30_P01021006 | Unknown | chr8:10886200-10905925_R | 2.24 |
| A_55_P2109927 | ens|ENSMUST00000020837|gb|AK053237|gb|AK053033|tc|TC1586557 | ENSMUST00000020837 | 2.24 |
| A_66_P135192 | ref|NM_001100451|ens|ENSMUST00000085177|gb|AK081923|gb|AK173208 | Msl2 | 2.24 |
| A_55_P2087944 | ref|XM_001472867|ref|XM_001472887 | Gm15452 | 2.24 |
| A_66_P128079 | ref|XR_004919|ref|XR_034809|gb|AK166854|gb|AK167120 | Gm5604 | 2.24 |
| A_55_P1990909 | ref|XM_001475915|nap|NAP066544-1 | LOC100046270 | 2.24 |
| A_55_P1986174 | ref|NM_178752|ens|ENSMUST00000062915|ref|XR_004799|gb|AK166345 | D330012F22Rik | 2.24 |
| A_30_P01022515 | Unknown | chr8:74473445-74496948_R | 2.24 |
| A_30_P01026381 | Unknown | chr14:33975934-33998694_R | 2.23 |
| A_55_P2081169 | ref|XR_032934|ref|XR_032789 | Gm4754 | 2.23 |
| A_55_P2069995 | ref|XR_032158|ref|XR_034034 | Gm15707 | 2.23 |
| A_51_P507051 | ref|NM_021560|ens|ENSMUST00000026120|gb|AK047201|gb|AK076228 | Bhlhe22 | 2.23 |
| A_52_P311853 | ref|NM_030143|ens|ENSMUST00000053855|gb|AK029362|gb|AK053333 | Ddit4l | 2.23 |
| A_55_P2122614 | ref|NM_001104566|nap|NAP101499-1 | Vmn2r104 | 2.23 |
| A_55_P1978271 | ens|ENSMUST00000070754|gb|AK076898|tc|TC1592649|riken|4930543A14 | ENSMUST00000070754 | 2.23 |
| A_51_P107053 | ref|NM_198623|ens|ENSMUST00000057254|gb|AK077072|gb|BC100419 | Ubqln3 | 2.23 |
| A_55_P2013177 | ref|NM_172724|ref|NM_181066|ens|ENSMUST00000100505|ens|ENSMUST00000064662 | AA881470 | 2.23 |
| A_55_P2006747 | ref|NM_028657|gb|BC118620|gb|BC119790|gb|BC038699 | F630110N24Rik | 2.23 |
| A_55_P2258467 | gb|AK076537|tc|TC1622457|tc|TC1708727|riken|4833445I07 | 4833445I07Rik | 2.23 |
| A_55_P1970763 | ref|NM_001024932|ens|ENSMUST00000110977|ens|ENSMUST00000085889|ref|XM_001479060 | Pilrb2 | 2.23 |
| A_55_P2454239 | ref|NM_001122992|ref|NM_020273|gb|AK049572|gb|AK044554 | Gmeb1 | 2.23 |
| A_51_P231768 | ref|NM_173187|ref|NM_029349|ens|ENSMUST00000086721|ens|ENSMUST00000039173 | 2310035C23Rik | 2.23 |
| A_55_P1952684 | ref|XM_001479939 | LOC100048289 | 2.23 |
| A_55_P1954608 | ref|NM_001037923|ens|ENSMUST00000099075|gb|AK137452|tc|TC1584986 | Lekr1 | 2.22 |
| A_55_P2077173 | ref|XM_001476322 | Gm6876 | 2.22 |
| A_55_P2099510 | ref|XM_001475618|ref|XM_001476720|gb|CF617977 | Gm2985 | 2.22 |
| A_55_P1977792 | ref|NM_008034|ens|ENSMUST00000001882|ens|ENSMUST00000106985|ens|ENSMUST00000106983 | Folr1 | 2.22 |
| A_55_P2003634 | ens|ENSMUST00000120605|tc|TC1654081|tc|TC1713381 | ENSMUST00000120605 | 2.22 |
| A_30_P01022956 | Unknown | chr13:70745306-70837331_R | 2.22 |
| A_55_P2086646 | ref|XR_035255|ref|XR_035287 | B230317F23Rik | 2.22 |
| A_55_P2358147 | gb|AK007349|tc|TC1737521|riken|1810006J02|nap|NAP090346-1 | 1810006J02Rik | 2.22 |
| A_55_P2001510 | ref|XM_001473743|ref|XM_001473771|ref|XM_001479920|ref|XM_001479927 | Gm2451 | 2.22 |
| A_55_P2062826 | ens|ENSMUST00000101442|gb|BC103789|tc|TC1586722 | ENSMUST00000101442 | 2.22 |
| A_51_P111532 | ref|NM_177823|ens|ENSMUST00000048656|ens|ENSMUST00000151620|gb|AK169696 | Ubash3a | 2.22 |
| A_55_P2011570 | ref|XM_980754 | Gm7881 | 2.22 |
| A_55_P2083014 | ens|ENSMUST00000103281|gb|FM179715|gb|FM179559|gb|GU599459 | ENSMUST00000103281 | 2.22 |
| A_55_P2112937 | ref|NM_146578|ens|ENSMUST00000111589|ens|ENSMUST00000062360|gb|AK041245 | Olfr1033 | 2.22 |
| A_55_P2193229 | gb|AK039367|tc|TC1607096|riken|A330032P22|nap|NAP080444-1 | A330032P22Rik | 2.22 |
| A_66_P137943 | ref|NM_026486|ens|ENSMUST00000100706|ref|XR_034387|gb|AK211913 | Tctn2 | 2.22 |
| A_52_P615247 | ens|ENSMUST00000111365|ens|ENSMUST00000113384|ens|ENSMUST00000113396|ens|ENSMUST00000096862 | ENSMUST00000111365 | 2.21 |
| A_55_P1953201 | ref|NM_146887|ens|ENSMUST00000080094|tc|TC1627217|nap|NAP022124-001 | Olfr1301 | 2.21 |
| A_51_P249848 | ref|NM_007883|ens|ENSMUST00000059787|gb|AB072269|gb|AJ000328 | Dsg2 | 2.21 |
| A_55_P2025645 | ref|XM_918515 | LOC641172 | 2.21 |
| A_51_P521090 | ens|ENSMUST00000044964|gb|AK144870|gb|AK008838|tc|TC1612220 | ENSMUST00000044964 | 2.21 |
| A_30_P01018551 | Unknown | chr1:115829055-115854155_R | 2.21 |
| A_55_P1996973 | ref|NM_029000|ref|NM_001039160|ens|ENSMUST00000006667|ens|ENSMUST00000106766 | Gvin1 | 2.21 |
| A_55_P1952230 | ref|NR_027658|ref|NR_027659|gb|BY735202|tc|TC1608542 | Gm8883 | 2.21 |
| A_55_P2311258 | gb|AK010467|tc|NP744695|riken|2410012E07|nap|NAP092043-1 | 2410012E07Rik | 2.21 |
| A_30_P01028945 | Unknown | chr13:114111261-114117957_F | 2.21 |
| A_66_P140224 | ens|ENSMUST00000119133|gb|AK006881|tc|TC1678225|riken|1700064H15 | ENSMUST00000119133 | 2.21 |
| A_55_P2062558 | ens|ENSMUST00000101131|gb|AK141725|riken|D030044C16 | ENSMUST00000101131 | 2.21 |
| A_30_P01029280 | Unknown | chr2:165780133-165800663_R | 2.21 |
| A_66_P100325 | ref|XM_001473412|ref|XM_001475672|gb|AK006052|gb|BU935594 | 1700016P04Rik | 2.20 |
| A_51_P512591 | ref|NM_172395|ens|ENSMUST00000053872|gb|AK028573|gb|AK004859 | Cdc42se1 | 2.20 |
| A_55_P2170564 | ref|NM_001004194|ens|ENSMUST00000076470|ens|ENSMUST00000036813|gb|AY596197 | Nlrp4e | 2.20 |
| A_30_P01030022 | Unknown | chr12:20620869-21028045_F | 2.20 |
| A_30_P01023504 | Unknown | chr12:21584725-21595050_F | 2.20 |
| A_30_P01031304 | Unknown | chr11:48603904-48611829_R | 2.20 |
| A_30_P01031470 | Unknown | chr6:31037606-31037912_R | 2.20 |
| A_55_P2070199 | ref|NM_173069|ref|NM_001037919|ref|NM_027650|ens|ENSMUST00000076500 | Speer2 | 2.20 |
| A_51_P166740 | ref|NM_008427|ens|ENSMUST00000057801|gb|S71382|gb|BC137594 | Kcnj4 | 2.20 |
| A_55_P2076333 | ref|NM_008665|ref|NM_001171680|ref|NM_001171616|ref|NM_001171615 | Myt1 | 2.20 |
| A_55_P1983544 | ref|NM_028876|ens|ENSMUST00000002837|ref|XM_001476475|ref|XM_001476494 | Tmed5 | 2.20 |
| A_51_P106397 | ref|NM_029239|ref|NM_001171004|ref|NM_001171005|ens|ENSMUST00000118768 | Prkd3 | 2.19 |
| A_55_P2105988 | ens|ENSMUST00000114775|gb|AK161955|riken|6430561E11 | ENSMUST00000114775 | 2.19 |
| A_55_P2080372 | ref|XM_001474438|ref|XM_001474423|ref|XM_620863|gb|AK157637 | Gm2749 | 2.19 |
| A_55_P2032423 | ref|XM_001477808 | LOC100047290 | 2.19 |
| A_51_P137604 | ref|NM_007995|ens|ENSMUST00000028307|gb|CT010383|gb|AB222271 | Fcna | 2.19 |
| A_52_P596592 | ref|NM_146240|ens|ENSMUST00000055355|gb|AK033464|gb|AK086855 | Rassf9 | 2.19 |
| A_51_P349402 | ref|NM_183185|ens|ENSMUST00000040667|gb|AK053018|tc|TC1592118 | Zfp300 | 2.19 |
| A_55_P2122125 | ref|NM_001005570|ens|ENSMUST00000088687|ref|XM_001474685|gb|BC153156 | Olfr707 | 2.19 |
| A_30_P01027300 | Unknown | chr17:84355587-84356863_F | 2.19 |
| A_30_P01031840 | Unknown | chrX:146827709-146913398_R | 2.19 |
| A_30_P01022620 | Unknown | chr3:27837825-27880925_R | 2.19 |
| A_52_P73498 | ens|ENSMUST00000118771|gb|AK077037|tc|TC1765984|tc|TC1580151 | ENSMUST00000118771 | 2.19 |
| A_55_P2009351 | ref|NM_025370|ens|ENSMUST00000021938|ens|ENSMUST00000099434|gb|AK006589 | 1110018J18Rik | 2.19 |
| A_55_P2153990 | ref|NM_001177439|ens|ENSMUST00000099140|ref|XR_035603|nap|NAP014076-001 | Gm1332 | 2.19 |
| A_55_P2129182 | ref|XM_001478114|gb|AK016337|gb|BY715978|tc|TC1607940 | 4930580E04Rik | 2.19 |
| A_55_P2295762 | gb|AK017040|riken|4933433G15|nap|NAP073624-1 | 4933433G15Rik | 2.19 |
| A_30_P01018573 | Unknown | chr3:127170996-127201096_R | 2.18 |
| A_51_P314991 | ref|NM_146798|ens|ENSMUST00000086061|gb|BC119221|gb|BC119219 | Olfr878 | 2.18 |
| A_30_P01023219 | Unknown | chr8:15580278-15583962_R | 2.18 |
| A_51_P510900 | ref|NM_026460|ens|ENSMUST00000039047|gb|AK007347|gb|AK007510 | Serpini2 | 2.18 |
| A_66_P102318 | ref|XR_034975|ref|XR_034986|nap|NAP060966-1 | Gm14131 | 2.18 |
| A_52_P519653 | ref|NM_147219|ens|ENSMUST00000043961|gb|AK138604|gb|AB097675 | Abca5 | 2.18 |
| A_52_P234910 | ref|XM_001472223|ref|XM_001474806|nap|NAP061240-1 | Gm15032 | 2.18 |
| A_51_P504735 | ref|NM_146791|ens|ENSMUST00000099770|gb|BC150718|nap|NAP110779-1 | Olfr1248 | 2.18 |
| A_30_P01023660 | Unknown | chr3:85589154-85621215_R | 2.18 |
| A_55_P1963175 | ref|NM_145835|ens|ENSMUST00000118215|ens|ENSMUST00000124020|ens|ENSMUST00000034969 | Lctl | 2.18 |
| A_55_P1991763 | ref|NM_001039373|ens|ENSMUST00000114081|ens|ENSMUST00000033542|ens|ENSMUST00000114083 | Mtcp1 | 2.18 |
| A_51_P247184 | ref|NM_008728|ref|NM_001039181|ens|ENSMUST00000066529|gb|AK046712 | Npr3 | 2.18 |
| A_55_P2459006 | ref|NM_177351|ens|ENSMUST00000039742|gb|AK085527|gb|AK162425 | Agphd1 | 2.18 |
| A_55_P2007016 | ref|NR_033707|ref|XM_001479616|ref|XM_909462|ref|XM_001479610 | Zscan4a | 2.18 |
| A_51_P286748 | ref|NM_011356|ens|ENSMUST00000028389|gb|AK019093|gb|U68058 | Frzb | 2.17 |
| A_30_P01031851 | Unknown | chr3:35782698-35789932_R | 2.17 |
| A_30_P01023245 | Unknown | chr15:61870759-61938796_F | 2.17 |
| A_55_P2087533 | ens|ENSMUST00000097321|gb|AK136747|riken|9230114N20 | ENSMUST00000097321 | 2.17 |
| A_55_P2078133 | ref|XR_031167|ref|XR_034215 | Gm7289 | 2.17 |
| A_55_P1993404 | ref|XR_030640|ref|XR_032318|nap|NAP111477-1 | Gm6335 | 2.17 |
| A_30_P01019674 | Unknown | chr7:57115122-57121735_R | 2.17 |
| A_55_P2133795 | ref|NM_008712|ens|ENSMUST00000111939|ens|ENSMUST00000111935|gb|D14552 | Nos1 | 2.17 |
| A_55_P2070494 | ref|NM_001105057|ens|ENSMUST00000085506|tc|TC1601866|nap|NAP098598-001 | Vmn2r60 | 2.17 |
| A_55_P2314396 | gb|AK014921|tc|NP742686|riken|4921518J05|nap|NAP124305-1 | 4921518J05Rik | 2.17 |
| A_55_P2170851 | ref|XM_001474833|ref|XM_001476366|gb|AK015798|gb|AV266265 | 4930515L03Rik | 2.17 |
| A_66_P111035 | ref|NM_001113416|ens|ENSMUST00000027632|gb|AK040354|gb|BC011476 | Epb4.1l5 | 2.17 |
| A_66_P113868 | ref|NM_007665|ref|NM_001037809|ens|ENSMUST00000080797|gb|X06340 | Cdh3 | 2.16 |
| A_51_P217282 | ref|NM_001081193|ens|ENSMUST00000053671|ens|ENSMUST00000105260|ens|ENSMUST00000119093 | Lemd3 | 2.16 |
| A_66_P126915 | ref|XM_001479256|ref|XM_001481095|gb|AK021326|riken|D730003K21 | D730003K21Rik | 2.16 |
| A_30_P01033656 | Unknown | chr4:146017200-146029700_F | 2.16 |
| A_55_P2173601 | ens|ENSMUST00000097884|tc|TC1652560 | ENSMUST00000097884 | 2.16 |
| A_55_P2180046 | ref|XM_001478512 | Gm10904 | 2.16 |
| A_30_P01029750 | Unknown | chr7:4827340-4863315_F | 2.16 |
| A_51_P116431 | ens|ENSMUST00000062118|gb|AK052446|tc|TC1580144|riken|D430024F16 | Pigv | 2.16 |
| A_55_P1973583 | ref|NM_001110252|ref|NM_008281|ens|ENSMUST00000108102|ens|ENSMUST00000039435 | Hpn | 2.16 |
| A_30_P01018582 | Unknown | chr12:52944867-52971992_R | 2.16 |
| A_30_P01028035 | Unknown | chrX:148720661-148763177_F | 2.16 |
| A_66_P111858 | ref|XR_034330 | LOC100048070 | 2.16 |
| A_55_P2009783 | ref|NM_026377|ens|ENSMUST00000099353|ens|ENSMUST00000099354|ens|ENSMUST00000099352 | 6330577E15Rik | 2.16 |
| A_30_P01026045 | Unknown | chr4:3406175-3443875_R | 2.16 |
| A_55_P1966508 | ref|NM_010465|ens|ENSMUST00000001711|gb|X16511|gb|X16510 | Hoxc6 | 2.16 |
| A_30_P01028146 | Unknown | chr18:67435075-67447150_F | 2.16 |
| A_55_P2298158 | gb|BB618401 | D8Ertd620e | 2.16 |
| A_55_P2070118 | ref|XM_001476431|ref|XM_001477441 | LOC100046569 | 2.16 |
| A_52_P69558 | ref|NR_033577|gb|BC052510|tc|TC1647934|nap|NAP112277-1 | Gm8221 | 2.15 |
| A_55_P2259248 | gb|AK043585|tc|TC1612200|riken|A830010I12|nap|NAP082500-1 | AI835086 | 2.15 |
| A_52_P557395 | ens|ENSMUST00000060705|gb|AK037190|tc|TC1590382|riken|A030007L22 | ENSMUST00000060705 | 2.15 |
| A_55_P2176150 | ref|NM_001040137|ref|NM_001040136|ens|ENSMUST00000033178|gb|AK006727 | Pdzd9 | 2.15 |
| A_30_P01029827 | Unknown | chr18:36470130-36489110_F | 2.15 |
| A_55_P2115225 | ref|NM_007986|ens|ENSMUST00000000402|ens|ENSMUST00000102732|gb|BC019190 | Fap | 2.15 |
| A_55_P2005883 | ref|NM_001033469|ens|ENSMUST00000098837|gb|AK139562|tc|TC1589496 | Foxr1 | 2.15 |
| A_55_P2270437 | gb|AK029922|tc|TC1718586|riken|4931441A13|nap|NAP124538-1 | 4930528J11Rik | 2.15 |
| A_55_P2085756 | ref|NM_146474|ref|NM_146794|ens|ENSMUST00000111509|ens|ENSMUST00000077785 | Olfr1261 | 2.15 |
| A_55_P2025687 | ref|NM_080457|ens|ENSMUST00000058568|ens|ENSMUST00000115119|ens|ENSMUST00000135753 | Muc4 | 2.15 |
| A_55_P2029895 | ref|XR_033265 | LOC676291 | 2.14 |
| A_52_P408736 | ens|ENSMUST00000063318|gb|AK085398|tc|TC1623955|riken|D630022A07 | Slc16a7 | 2.14 |
| A_55_P2184200 | ref|XM_001472543|ref|XM_001476595 | Gm3410 | 2.14 |
| A_51_P439403 | ref|NM_011059|ens|ENSMUST00000026378|gb|AB013848|gb|AK037182 | Padi1 | 2.14 |
| A_30_P01020643 | Unknown | chr9:7214754-7225700_R | 2.14 |
| A_52_P447284 | ref|NM_172469|ens|ENSMUST00000023670|gb|AK077599|gb|BC075706 | Clic6 | 2.14 |
| A_55_P2108216 | ref|XM_001472666|ref|XM_001476710 | Gm3437 | 2.14 |
| A_55_P2000574 | ref|NM_001033340|ens|ENSMUST00000097290|gb|BC147419|gb|BC147420 | Lrrc30 | 2.14 |
| A_51_P289889 | ref|NM_033525|ref|NM_001029836|ens|ENSMUST00000093971|ens|ENSMUST00000117164 | Npnt | 2.14 |
| A_51_P264695 | ref|NM_016669|ens|ENSMUST00000033198|gb|AK143116|gb|AK138422 | Crym | 2.14 |
| A_55_P2064466 | ref|XM_001477040|ref|XM_001472746 | Gm13539 | 2.14 |
| A_52_P178963 | ref|NM_145709|ref|NM_145708|ref|NM_145707|ens|ENSMUST00000098802 | Obox5 | 2.14 |
| A_30_P01030989 | Unknown | chr12:7530807-7866572_R | 2.13 |
| A_55_P2147301 | Unknown | A_55_P2147301 | 2.13 |
| A_55_P2055050 | ref|XM_001476789|ref|XM_001477129|gb|AK016061|riken|4930547M16 | 4930547M16Rik | 2.13 |
| A_30_P01022967 | Unknown | chr4:63736566-63744848_R | 2.13 |
| A_52_P204809 | ref|NM_053138|ens|ENSMUST00000056915|gb|AK082435|gb|BB387231 | Pcdhb13 | 2.13 |
| A_51_P117903 | ens|ENSMUST00000103467|ens|ENSMUST00000103463|gb|EU568223|gb|U37848 | LOC630322 | 2.13 |
| A_52_P63022 | ref|XM_485924|ref|XM_916641|gb|AK137863|gb|BC063263 | Zfp141 | 2.13 |
| A_55_P2117656 | ref|NM_181545|ref|NM_001167743|ens|ENSMUST00000108152|ens|ENSMUST00000038141 | Slfn8 | 2.13 |
| A_55_P2149756 | ens|ENSMUST00000070439 | ENSMUST00000070439 | 2.13 |
| A_30_P01028847 | Unknown | chr13:22110479-22116629_F | 2.13 |
| A_55_P2140903 | ref|NR_002871|tc|TC1748951 | Vax2os2 | 2.12 |
| A_55_P2164413 | ref|NM_148953|gb|BC127245|gb|AF403044|gb|BC127246 | Asb16 | 2.12 |
| A_30_P01029713 | Unknown | chr15:38205154-38206196_F | 2.12 |
| A_55_P2137927 | ref|NM_001136055|ref|NM_007656|ens|ENSMUST00000111257|ens|ENSMUST00000028644 | Cd82 | 2.12 |
| A_66_P108986 | gb|AK054090|riken|E230017H14|nap|NAP017763-001 | Hoxd11 | 2.12 |
| A_30_P01018489 | Unknown | chr15:92667158-93020682_R | 2.12 |
| A_55_P1973373 | ens|ENSMUST00000109647|ens|ENSMUST00000093308|gb|AK079096|tc|TC1591691 | ENSMUST00000109647 | 2.12 |
| A_51_P243094 | ref|NM_053221|ens|ENSMUST00000089419|gb|BC125372|tc|TC1599879 | Vmn1r42 | 2.12 |
| A_30_P01020672 | Unknown | chrX:18734106-18744367_R | 2.12 |
| A_52_P404341 | ref|NM_019911|gb|AB476989|gb|AB476990|gb|BC018390 | Tdo2 | 2.12 |
| A_55_P2223710 | gb|BC068229|tc|TC1600661 | 2700054A10Rik | 2.11 |
| A_55_P2060238 | ref|NM_145467|ens|ENSMUST00000038582|ens|ENSMUST00000100289|gb|AK029068 | Itgbl1 | 2.11 |
| A_55_P2175977 | ref|NM_001105067|ref|NM_001105062|ref|NM_001105065|ref|NM_001105070 | Vmn2r35 | 2.11 |
| A_55_P2065325 | ref|NM_028014|ens|ENSMUST00000103034|ens|ENSMUST00000103033|ens|ENSMUST00000093912 | 2310067B10Rik | 2.11 |
| A_55_P1963570 | ref|NM_146331|ens|ENSMUST00000080329|gb|BC148448|gb|BC153039 | Olfr954 | 2.11 |
| A_55_P1979929 | ref|NM_028243|ens|ENSMUST00000076052|ref|XR_034467|gb|AK051677 | Prcp | 2.11 |
| A_55_P2275437 | gb|AK002860|gb|BY703358|tc|TC1635334|riken|0610040B09 | 0610040B09Rik | 2.11 |
| A_55_P2089577 | ref|XM_001476475|ref|XM_001476494|gb|AK172643|riken|F830223E18 | LOC100046567 | 2.11 |
| A_55_P1962693 | ref|NM_001122954|ref|NM_011110|ens|ENSMUST00000102513|ens|ENSMUST00000102512 | Pla2g5 | 2.11 |
| A_55_P2136000 | ref|XM_001478524|ref|XM_001478634 | Gm3936 | 2.11 |
| A_51_P239214 | ref|NM_001113367|ref|NM_029267|ens|ENSMUST00000114423|ens|ENSMUST00000087585 | Boll | 2.11 |
| A_52_P543869 | ref|NM_029031|ens|ENSMUST00000006105|gb|AK171027|gb|BC054721 | Shpk | 2.11 |
| A_55_P2157549 | ens|ENSMUST00000103488|nap|NAP013673-001 | ENSMUST00000103488 | 2.11 |
| A_52_P63855 | ref|NM_199422|ens|ENSMUST00000079286|gb|AY465109|tc|TC1592055 | S100a7a | 2.11 |
| A_55_P2129658 | ref|NM_008542|ens|ENSMUST00000041029|gb|BC047280|gb|AK046602 | Smad6 | 2.11 |
| A_30_P01024639 | Unknown | chr6:28870220-28870692_R | 2.11 |
| A_55_P1983853 | ref|NM_009808|ens|ENSMUST00000027009|ref|XM_001471642|gb|AK030309 | Casp12 | 2.11 |
| A_55_P2174836 | ref|NM_013703|ens|ENSMUST00000025866|gb|L33417|gb|U06670 | Vldlr | 2.11 |
| A_30_P01021501 | Unknown | chr2:150072436-150074859_F | 2.11 |
[truncated: 16,019 more chars]
